# Sequana coverage: detection and characterization of genomic variations using running median and mixture models

**DOI:** 10.1093/gigascience/giy110

**Published:** 2018-09-06

**Authors:** Dimitri Desvillechabrol, Christiane Bouchier, Sean Kennedy, Thomas Cokelaer

**Affiliations:** 1 Institut Pasteur – Pole Biomics – 25-28 Rue du Docteur Roux, 75015 Paris, France; 2 Institut Pasteur – Bioinformatics and Biostatistics Hub – C3BI, USR 3756 IP CNRS – Paris, France

**Keywords:** genome coverage, sequencing depth, running median, Sequana, NGS, Python, Snakemake, CNV

## Abstract

**Background:**

In addition to mapping quality information, the *Genome coverage* contains valuable biological information such as the presence of repetitive regions, deleted genes, or copy number variations (CNVs). It is essential to take into consideration atypical regions, trends (e.g., origin of replication), or known and unknown biases that influence coverage. It is also important that reported events have robust statistics (e.g. *z*-score) associated with their detections as well as precise location.

**Results:**

We provide a stand-alone application, sequana_coverage, that reports genomic regions of interest (ROIs) that are significantly over- or underrepresented in high-throughput sequencing data. Significance is associated with the events as well as characteristics such as length of the regions. The algorithm first detrends the data using an efficient running median algorithm. It then estimates the distribution of the normalized genome coverage with a Gaussian mixture model. Finally, a *z*-score statistic is assigned to each base position and used to separate the central distribution from the ROIs (i.e., under- and overcovered regions). A double thresholds mechanism is used to cluster the genomic ROIs. HTML reports provide a summary with interactive visual representations of the genomic ROIs with standard plots and metrics. Genomic variations such as single-nucleotide variants or CNVs can be effectively identified at the same time.

## Background

Sequencing technologies allow researchers to investigate a wide range of genomic questions [1], covering research fields such as the expression of genes (transcriptomics) [2], the discovery of somatic mutations, or the sequencing of complete genomes of cancer samples, to name a few [3, 4]. The emergence of second-generation sequencing, which is also known as next-generation sequencing, or NGS hereafter, has dramatically reduced the sequencing cost. This breakthrough multiplied the number of genomic analyses undertaken by research laboratories but also yielded vast amounts of data. Consequently, NGS analysis pipelines require efficient algorithms and scalable visualization tools to process this data and to interpret the results.

Raw data generated by NGS experiments are usually stored in the form of sequencing reads (hereafter simply called reads). A read stores the information about a DNA fragment and also an error probability vector for each base. Read lengths vary from 35 to 300 bases for current short-read approaches [1] to several tens of thousands of bases possible with long-read technologies such as Pacific Biosciences [5, 6] or Oxford Nanopore [7].

**Figure 1:**
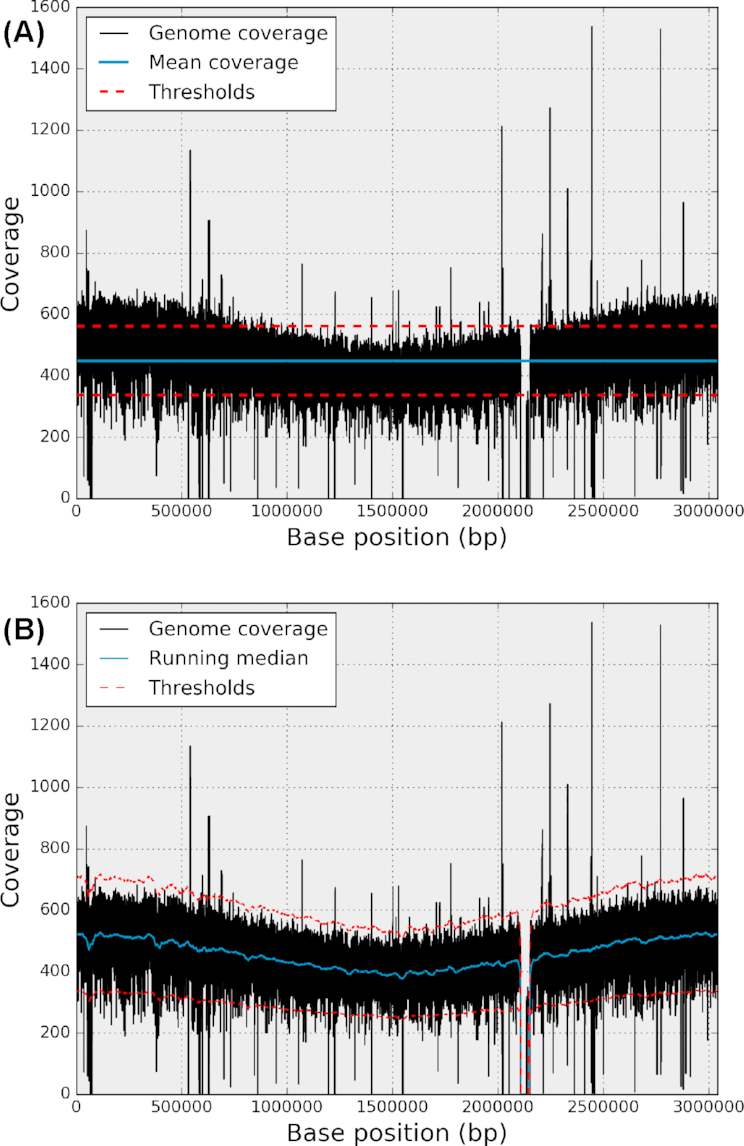
Example of a genome coverage series (in black in both panels). The genome coverage corresponds to the bacteria test case (see text). It contains a deleted region (around 2.2 Mbp) and various under- and overcovered regions (from 100 bp to several Kbp). Although the sequencing depth is about 500X, there is a nonlinear trend from 500X on both ends to 400X in the middle of the genome. The top panel shows the sequencing depth (blue horizontal line) and two arbitrary fixed thresholds (dashed red lines) at 400X and 500. Due to the nonlinear trend, the fixed thresholds lead to an increase of type I and type II errors. On the contrary, in the bottom figure, the trend is estimated using a RM (red line), and adaptive lower and upper thresholds (dashed red lines) can be derived.

After trimming steps (quality, adapter removal), most high-throughput sequencing (HTS) experiments will require mapping the reads onto a genome of reference [8]. If no reference is available, a *de novo* genome assembly can be performed [9]. In both cases, reads can be mapped back on the reference taking into account their quality. We define the *genome coverage* as the number of reads mapped to a specific position within the reference genome. The theoretical distribution of the genome coverage has been thoroughly studied following the seminal work of the Lander-Waterman model [10, 11]. A common metric used to characterize the genome coverage is the sequencing depth [12], which is the empirical average of the genome coverage. It may also be called depth of coverage (DOC), fold coverage, read depth, or confusingly, depth or coverage. The sequencing depth unit is denoted X. An example of genome coverage with a sequencing depth of about 450X is shown in Fig. 1. Another useful metric is the breadth of coverage (BOC), which is the proportion of the intended genome reference covered by at least one read.

The required sequencing depth depends on the experimental application. For instance, to detect human genome mutations, single-nucleotide polymorphisms (SNPs), and rearrangements, a 30 to 50X depth is recommended [1, 13] in order to distinguish between sequencing errors and true SNPs. In contrast, the detection of rarely expressed genes in transcriptomics experiments often requires greater sequencing depth. However, greater sequencing depth is not always desirable. Indeed, in addition to a higher cost, ultradeep sequencing (large sequencing depth in excess of 1,000X) may be an issue for a *de novo* genome assembly [14].

The Lander-Waterman model provides a good theoretical estimate of the required sequencing depth to guarantee that all nucleotides are covered at least *N* times. This is, however, a theoretical estimate that does not take into account technical and biological limitations; some regions being difficult to efficiently map (e.g., repetitive DNA) or containing compositional biases (e.g., GC bias [15]). Furthermore, the genome coverage itself may contain a nonconstant trend along the genome due to the impact of the origin of replication. Finally, some regions may be deleted or duplicated. The genome coverage example shown in Fig. 1 shows these different features.

While the sequencing depth and other metrics (e.g., BOC) provide a quick understanding about the quality of sequencing and mapping, the genome coverage can also be analyzed to identify genomic variations such as single-nucleotide variations (SNVs) or copy number variations (CNVs) [16–17].

In order to detect genomic regions of interest (ROIs) based on genome coverage, a simple and fast approach might be to set two arbitrary thresholds bounding the sequencing depth. However, there are two major drawbacks with this approach. First, as shown in Fig. 1 (top panel) and Notebook 4 in [18], with a fixed threshold, one may detect numerous false signals (type I errors) or fail to detect real events (type II errors). An adaptive threshold that follows the trend of the genome coverage is thus required. Furthermore, a fixed threshold is arbitrary, and so the detected events lack a robust means of assigning significance. A more robust alternative is to estimate the genome coverage profile histogram [19] from which a *z*-score statistic can be used to identify outliers more precisely. Due to a number of known and unknown biases, one should still normalize the data [16]. There are a number of different methods for detecting the ROIs. For example, for CNV detection, numerous techniques are used [17], such as the mean-shift technique [20] or bias correction followed by application of a complex statistical model [16].

Here, we describe a novel approach that can be used to efficiently detect various types of genomic ROIs. The algorithm does not target any specific type of genomic variations but instead systematically reports all positions (with a *z*-score) that have depth departing from the overall distribution. The algorithm normalizes the genome coverage using a running median (RM) and then calculates a robust statistic (*z*-score) for each base position based on the parameter estimation of the underlying distribution. This allows us to obtain robust and nonconstant thresholds at each genome position. Various types of clustering or filtering can then be implemented to focus on specific categories of variations.

In the Data Description section, we describe the datasets used throughout as test-case examples. In the Methods section, we describe the RM used to detrend the genome coverage, the statistical methods used to characterize the central distribution from which outliers can be identified, and a double thresholds method proposed to cluster the ROIs. Finally, in the Applications section, we describe the standalone application, sequana_coverage, and potential applications for HTS-dependent research projects, including CNV detection.

## Data Description

Three test cases of genome coverage are presented here, covering representative organisms and sequencing depths. The genome coverage datasets are in BED (Browser Extensible Data) format, a tabulated file containing the coverage, reference (e.g., chromosome number, contig), and position on the reference. BED files can be created from binary alignment (BAM) files (mapped reads) using bedtools [21], in particular, the genomecov tool.

We first considered bacteria from a study of methicillin-resistant *Staphylococcus aureus* [22]. One circular chromosome of 3 *Mbp* is present. The sequencing depth is 450X and the genome coverage exhibits a nonconstant trend along the genome (see Fig. 1). This pattern, often observed in rapidly growing bacteria, is the result of an unsynchronized population where genome replication occurs bidirectionally from a single origin of replication [23, 24]. The proportion of outliers (see Table 1) is about 2.5% of the total bases. The original datasets (Illumina sequencing reads, paired-end, 100 bp) are available at the European Nucleotide Archive (ENA) [25] under study accession number PRJEB2076 (ERR036019). The accession number of the reference is FN433596.

The second organism is a virus with a sequencing depth of 1000X [26]. A circular plasmid containing the virus chromosome is 19,795 bp long. About 13% of the genome coverage contains large or low coverage regions (outliers). It also contains two large undercovered regions (one partially undercovered and one region that is not covered at all), as shown in the Notebook 1 of [18]). The accession number of the reference is JB409847.

The third test case is a fungus (*Schizosaccharomyces pombe*) [27]. The genome coverage has a sequencing depth of 105 X. It has three noncircular chromosomes of 5.5 Mbp, 4.5 Mbp, and 2.5 Mbp. The references from ENA are CU329670.1, CU329671.1, and CU329672.1. Although we will look at the first chromosome only (1.5% of outliers), the tools presented hereafter handle circular chromosomes and multiple chromosomes. See examples in Notebook 3 of [18].

We provide the three genome coverage data files in BED format on Synapse [28, 29]. See Availability of supporting data and materials, at the end of the article, for more details.

In addition to these three cases, we also use a population composed of six *S. aureus* isolates from [16] (Supplementary Data), which is used to measure the efficiency of our algorithm against two dedicated CNVs detection tools: CNOGpro [16] and CNVnator [20].

## Methods

### Detrending the genome coverage

The genome coverage function is denoted *C*(*b*) where *b* is the base (nucleotide) position on the genome of reference. The genome coverage and reference lengths are denoted *N*. For simplicity, we drop the parentheses and refer to the genome coverage as *C_b_*. The empirical sequencing depth (average of genome coverage) is denoted $\delta = \overline{C}_b$. Ideally, *C_b_* is made of a continuous homogeneous central region. In practice, however, this may be interrupted by a succession of under- and overcovered regions; these are the genomic ROIs that we want to detect.

A naive classifier consists of setting two fixed thresholds, δ^−^ and δ^+^, whereby low and high ROIs are defined as $C^-_b=C_b\le \delta ^-$ and $C^+_b=C_b\ge \delta ^+$, respectively. If $C^0_b$ denotes the remaining data such that $\delta ^- \lt C_b^0 \lt \delta ^+$, then the genome coverage can be written as $C_b = \lbrace C^0_b, C^+_b, C^-_b\rbrace$.

**Figure 2:**
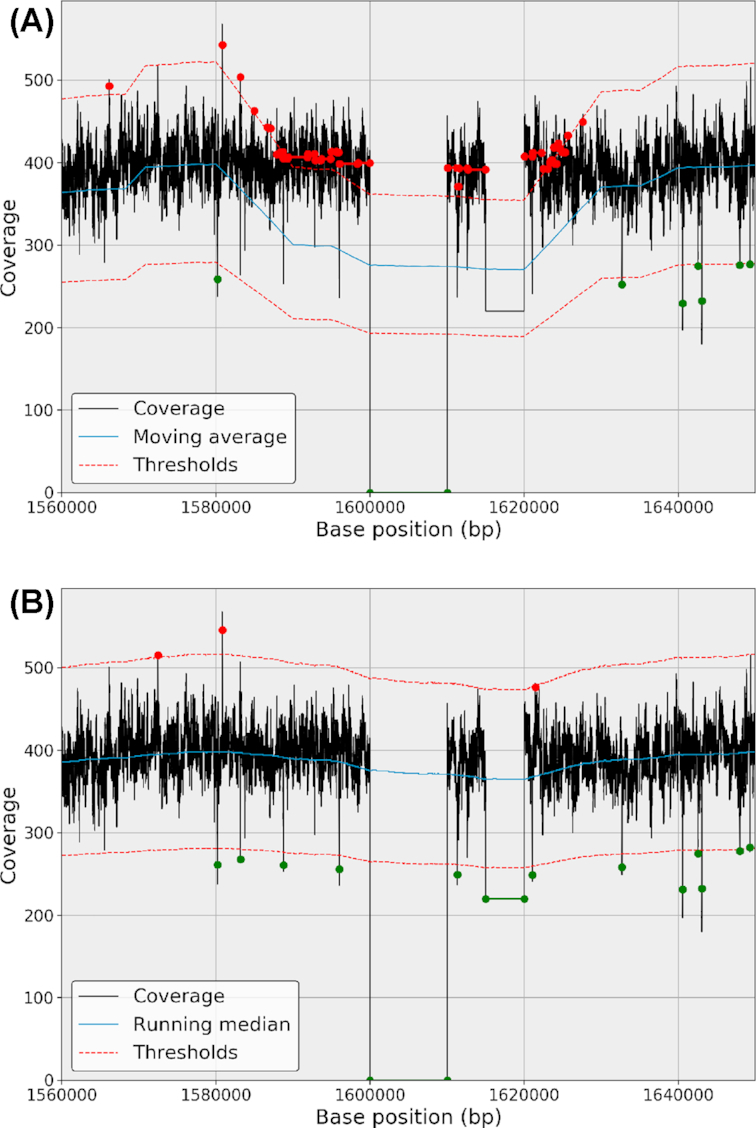
Moving average (top panel) and RM (bottom panel) behaviors in the presence of outliers (here, a deleted region in the center followed by a depleted region). In both cases, the window parameter is set to 40,000 bases. The presence of the deleted and depleted regions shows how the moving average (blue line, top panel) can be shifted compared to the RM (blue line, bottom panel). In the top panel, the thresholds (red lines) are also shifted and consequently the depleted region (position 1,620,000) is not detected. In addition, the rate of false detection increases (red dots). On the contrary, the RM has a better behavior with fewer false detections and the ability to detect the depleted region.

The advantage of the fixed-thresholds method is that it is conceptually simple and computationally inexpensive. However, there are two major drawbacks. First, as shown in Fig. 1A, false negatives and false positives will increase as soon as there is a nonconstant trend present in the data. It may be a low-frequency trend as shown here, but high-frequency trends are also present (see, e.g., Fig. 2). Also of importance is that an arbitrary choice of threshold(s) is unsatisfactory from a statistical point of view since we cannot associate any level of significance to a genomic region.

In order to account for a possible trend in the genome coverage series (and remove it), a standard method consists of dividing the series by a representative alternative such as its moving average (MA) or RM.

The MA is computed at each position, *b*, as the average of *W* data points around that position and defined as follows:

(1) \begin{equation*}
\rm {MA}_W(b) = \frac{1}{W}\sum _{i=-V}^{V} C(b+i),
\end{equation*}

where *W* is the length of the moving window (odd number) and *V* = (*W* − 1)/2. Note that the first and last *V* values are undefined. However, in the case of circular DNA (e.g., viral or bacterial genomes), the first and last *V* points are defined since *C_b_* is now a circular series.

Similarly, the RM is computed at each position, *b*, as the median of *W* data points around that position:

(2) \begin{equation*}
\rm {RM}_W(b) = median(\lbrace C(b-V), .., C(b+V)\rbrace ),
\end{equation*}

where *W* and *V* are defined as before and the median function is defined as the middle point of the sample set (half of the data are below the median and half are above). A mathematical expression of the median and running median are given in the Appendix (Eq. 8).

The mean estimator is commonly used to estimate the central tendency of a sample; nevertheless, it should be avoided in the presence of extraneous outliers, which are common in NGS genome coverage series (see, e.g., Fig. 1). Figure 2 shows the impact of outliers when using an MA or a running mean. We will use the running median (RM) only and define the normalized genome coverage as follows:

(3) \begin{equation*}
\widetilde{C}_b = \frac{C_b}{RM_W(b)}.
\end{equation*}

We will use the tilde symbol for all metrics associated with the normalized genome coverage, $\widetilde{C}_b$. For instance, $\widetilde{C}_b=\lbrace \widetilde{C}^0_b, \widetilde{C}^+_b, \widetilde{C}^-_b\rbrace$.

The RM is used in various research fields, in particular, in spectral analysis [30] to estimate the noise floor while ignoring biases due to narrow frequency bands (e.g., [31]). Here, the goal is to avoid narrow peaks but also to be insensitive to long deleted regions. This can be a major issue in NGS as the RM estimator complexity is a function of the window length. Indeed, the RM algorithm involves the sorting of a sample of length *W* at each position of the genome. So, the RM estimator must be efficient and scalable. This is not an issue in spectral analysis and most fields where RMs are used but is a bottleneck for NGS analysis where *W* is large. As explained in the Appendix, the complexity of the sorting part is in *O*(*n*^2^) in the worst case but, as with the MA, one can take advantage of the rolling window and the fact that the previous block is already sorted. We opted for the very efficient Pandas [32] implementation (see Appendix for details). In our implementation, both the MA and RM have the ability to account for circular DNA data, which are essential for handling a circular series.

**Figure 3:**
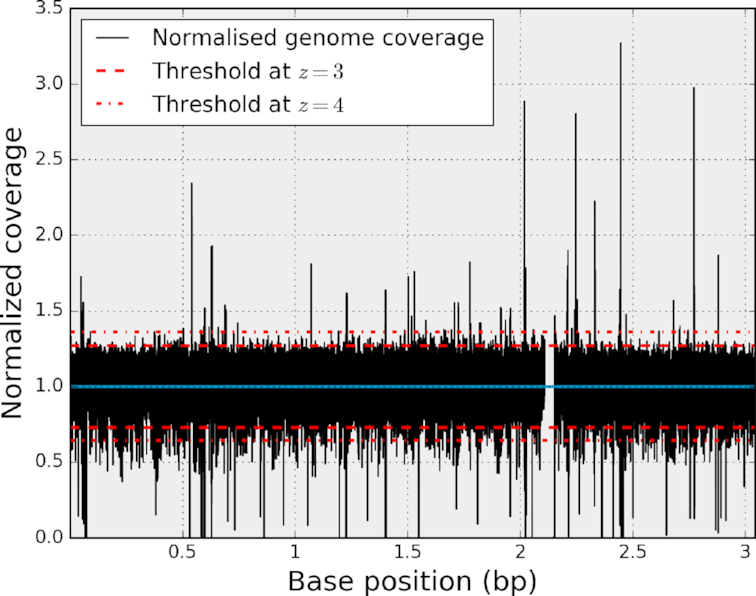
Normalized genome coverage $\widetilde{C}_b$ (bacteria test case). The outliers present in the original genome coverage *C_b_* (see Fig. 1) are still present as well as the deleted regions. The distribution is now centered on unity (blue line). Since the distribution is normalized, constant thresholds can be used (dashed lines).

If we normalize the genome coverage from the bacteria example (Fig. 1), we obtain the results shown in Fig. 3. Finally, note that with the genome coverage being discrete, the RM is also discrete as well as the normalized genome coverage. The discreteness will become more pronounced as sequencing depth decreases.

Hereafter, we will discuss the impact of the *W* parameter on the detection of genomic ROIs and how to set its value.

### Parameter estimation of the central distribution and adaptive thresholds in the original space

In the ideal case of randomly distributed reads across the genome, the number of reads covering each base follows a Poisson distribution [10]. This distribution is discrete and has one parameter that corresponds to the sequencing depth (mean of the distribution). Yet, the Poisson distribution is often too narrow [19], as can be observed in the three test cases considered. This is generally due to biological overdispersion. In order to account for overdispersion, the Poisson parameter can be distributed according to a second distribution. For instance, when the Poisson parameter is distributed according to a Gamma distribution, we obtain a negative binomial, which has two shape parameters [19].

A Poisson distribution with a large mean parameter approximates a normal distribution, even though, technically speaking, it is not (discrete vs continuous and one parameter vs two). Yet, for δ ≫ 1, we can assume that the *C_b_* distribution exhibits a Gaussian distribution denoted $\mathcal {N}(\mu , \sigma ^2)$ hereafter where μ is the average of the genome coverage (δ in an ideal case) and σ is its standard deviation. What about the normalized genome coverage $\widetilde{C}_b$? It is a ratio distribution where the numerator follows $\mathcal {N}(\mu , \sigma ^2)$ distribution while the denominator’s distribution is that of the RM. We can see empirically that for large δ and small *W* parameter, the distribution of the RM follows a Gaussian distribution, while for large *W* or small δ, the RM tends to be discrete and the distribution may depart from a Gaussian distribution (see Notebook 7 of [18]). Even if we knew the RM distribution, the ratio distribution is only known for two Gaussian distributions *X* and *Y* (Cauchy distribution) and when (i) the two distributions are centered on zero, which is not the case, and (ii) when they are independent, which is also not the case. Furthermore, the scenario we considered (ideal distribution, δ ≫ 1) is too restrictive since we are interested in identifying outliers in real data and may encounter cases where δ is small (for which *C_b_* follows a negative binomial, not a Gaussian distribution). So, we envisage a solution based on a mixture model as described hereafter.

Genome coverage is a mix of distributions. Consider, for instance, the presence of many CNVs, each with a different copy number (CN; either depletion or duplication). The overall distribution here would be very difficult to model analytically. Therefore, the assumption and our goal are to fit a known distribution on the central distribution so as to establish *z*-scores on the remaining data.

Our first hypothesis is that $\widetilde{C}_b$ can be decomposed into a central distribution, $\widetilde{C}^0_b$, and a set of outliers, $\widetilde{C}^1_b = \lbrace \widetilde{C}^+_b, \widetilde{C}^-_b\rbrace$ where the central distribution is predominant: $\left|\widetilde{C}^0_b\right| \gt \left|\widetilde{C}^1_b\right|$ (vertical bars indicate the cardinality of the sets).

**Figure 4:**
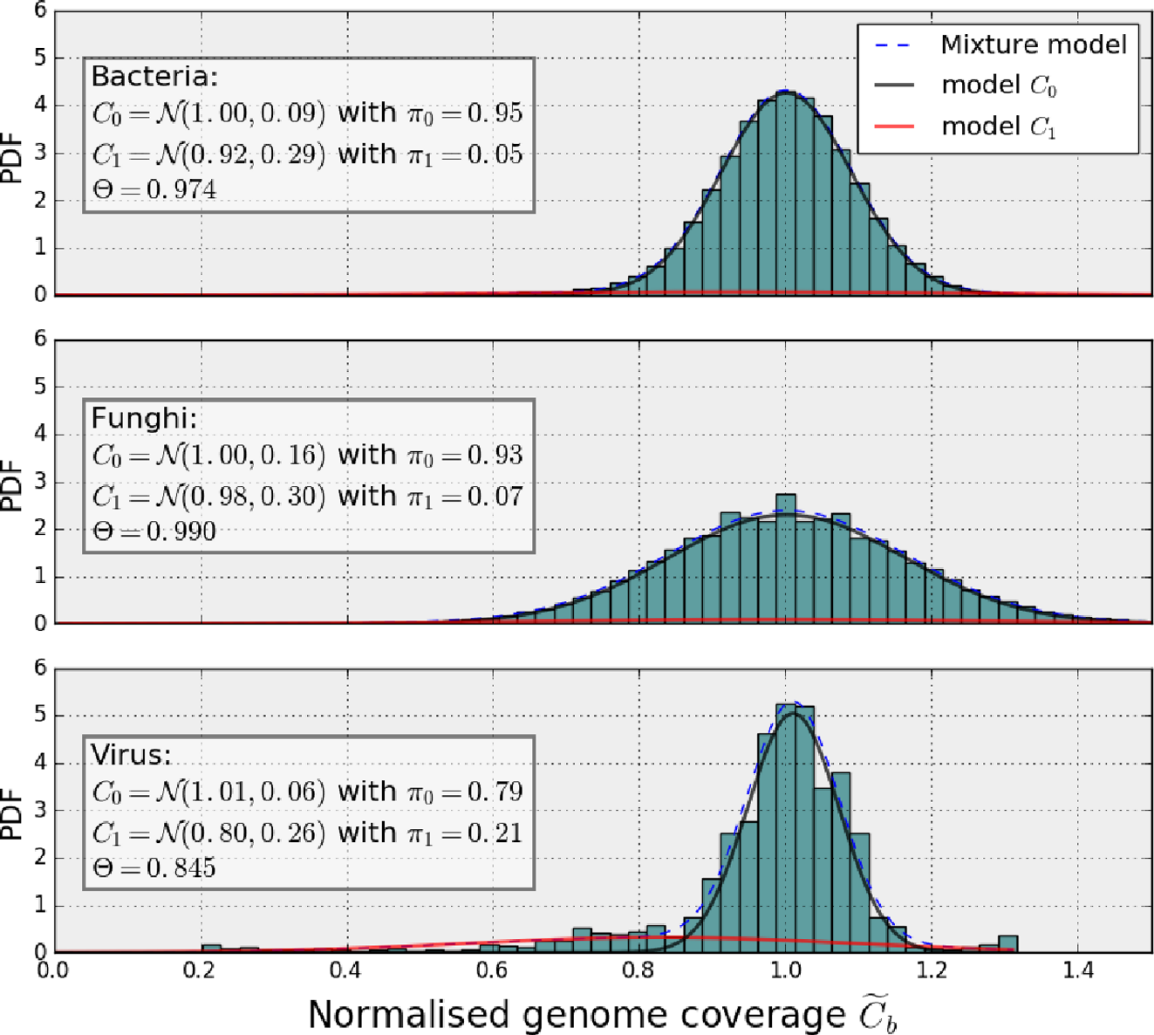
Probability density functions (PDFs) of the normalized genome coverage function concerning the three test cases. The distributions were fitted with Gaussian mixture models with *k* = 2 models. The first model (black line) fits the central distribution’s PDF, and the second model (red line close to *y* = 0) fits the outliers’ PDF. The dashed line (close to the black lines) indicates the mixture distribution. In each panel, we report the parameters of the two Gaussian distributions, the proportions π_0_, π_1_, and the Θ parameter introduced in the text that gives the centralness of the data for each test case.

Our second hypothesis is that the mixture model that represents $\widetilde{C}_b$ is a Gaussian mixture model of *k* = 2 models only: $\widetilde{C}^0_b \sim \mathcal {N}(\widetilde{\mu }_0, \widetilde{\sigma }_0^2)$ and $\widetilde{C}^1_b \sim \mathcal {N}(\widetilde{\mu }_1, \widetilde{\sigma }_1^2)$. The central distribution $\widetilde{C}^0_b$ exhibits a clear Gaussian distribution both on simulated data (see Notebook 7 in [18]) and on real data (see the three examples in Fig. 4). The second model is used to identify outliers (below or above the central distribution). The parameters of the second model are not used in defining the central distribution and so have little impact on detection.

Similar to the method deployed in [19] to identify a mixture model of negative binomials (on raw genome coverage), we will use an expectation maximization (EM) [33] method to estimate the parameters $\widetilde{\mu }_{0,1}$ and $\widetilde{\sigma }_{0,1}$ (on the normalized genome coverage).

The EM algorithm is an iterative method that alternates between two steps: an expectation step that creates a function for the expectation of the log-likelihood using the current estimate of the parameters and a minimization step that computes parameters maximizing the expected log-likelihood found in the first step. The likelihood function and the maximum likelihood estimate can be derived analytically in the context of Gaussian distributions. Note that in addition to the means and standard deviations, the mixture parameters also need to be estimated. These are denoted $\widetilde{\pi }_0$ and $\widetilde{\pi }_1$. The EM algorithm is standard and can be found in various scientific libraries. Note, however, that the normalized genome coverage may contain zeros in the presence of deleted regions, and the estimation of the mixture model should ignore them.

We have applied the EM algorithm on the normalized genome coverage vector on various real NGS datasets, including the three test cases in Fig. 4. The EM retrieves the parameters of the central distribution (in particular, $\tilde{\mu }_0=1$) and the outliers. Note that the choice of the RM parameter, *W*, does not significantly affect the parameter estimation. In each case, the mean of the central distribution is very close to unity. The standard deviation varies significantly and is a function of the sequencing depth only (since the outliers are now incorporated in $C_b^1$). Finally, we can confirm that the proportion of outliers is small compared to the central distributions by inspection of parameters π_0_ and π_1_: $\widetilde{\pi }_0 \gt \gt \widetilde{\pi }_1$.

Once we have identified the parameters of the central distribution $\widetilde{C}_0$, we can assign statistics for $\widetilde{C}_b$ in terms of *z*-score:

(4) \begin{equation*}
z(b) = \frac{\widetilde{C}(b)-\widetilde{\mu }_0}{\widetilde{\sigma }_0}.
\end{equation*}

Since the *z*-score corresponds to a normal distribution, we can now set a threshold in terms of tolerance interval within which a specified proportion of the genome coverage falls. For instance, with a threshold of 3, we know from the normal distribution that 99.97% of the sample lies in the range –3 and +3. The exact mathematical value is given by the complementary error function, erfc(*x*), where $x=n/\sqrt{2}$. Note that for *n* = 3, 4, and 5, the tolerance interval is 99.73%, 99.993%, and 99.999942%, respectively. Thus, for a genome of 1 Mbp, by pure chance, we should obtain about 2,700, 70, and 1 outlier(s), respectively.

If we now replace $\widetilde{C}_b$ in Eq. 4 using its expression from Eq. 3, we can express the original genome coverage as a function of the RM, the *z*-score, and the parameters of the central distribution:

(5) \begin{equation*}
C(b) = \left( \widetilde{\mu }_0 + z(b) \widetilde{\sigma }_0 \right)RM_W(b).
\end{equation*}

We can now set a fixed threshold *z*(*b*) = ±*n* in the normalized space. This is much easier to manipulate. Moreover, we can derive a variable threshold in the original space that is function of the genome position:

(6) \begin{equation*}
\tilde{\delta }^\pm (b) = \left( \widetilde{\mu }_0 \pm n^\pm \times \widetilde{\sigma }_0 \right)RM_W(b).
\end{equation*}

Examples of variable upper and lower threshold functions are shown in Figs. 1 and 2 (red dashed lines). This manipulation results in a robust statistical estimate of the presence of outliers in the genome coverage. The *z*-score, computed earlier, provides a precise level of confidence.

Using the normalization presented above, we can define the *centralness* as one minus the proportion of outliers contained in the genome coverage:

(7) \begin{equation*}
\Theta _n = 1 - \frac{\left| \widetilde{C}^1_b \right|}{\left|\widetilde{C}_b\right|} = 1-\frac{\left| \widetilde{C}^1_b \right|}{G},
\end{equation*}

where *G* is the length of the genome and vertical bars indicate the cardinality. This necessarily depends on how the threshold *n* is set in the normalized space. In the case of an ideal Gaussian distribution and *n* = 3, the centralness should equal the tolerance interval of a normal distribution $\mathcal {N}(0,1)$ that is the error function, $\textrm{erf}(n/\sqrt{2})$. The centralness equals unity when there are no outliers, i.e., *n* → ∞. Finally, note that the centralness is meaningless for values below 0.5 (meaning that the central distribution is not central!). As shown in Table 1, Θ_3_ equals 0.974, 0.99, and 0.86 in the three cases considered (bacteria, fungus, and virus). So the proportion of outliers in the virus case is higher than in the two other test cases, which is not obvious at first glance given the very different lengths of the genome considered.

**Table 1:** Metrics derived from the genome coverage of the three test cases considered—bacteria, fungus, and virus).

Metric	Bacteria	Fungus	Virus
Genome length	3 Mbp	5.5 Mbp	19,795 bp
BOC	0.985	1.0	0.966
Mean δ	447.8	105.49	931.3
Median δ	453	105	988
σ	84.1	19.9	237.2
CV	0.19	0.19	0.25
*W*	5,001/(20,001)	5,001/(20,001)	5,001
$\widetilde{\mu }_0$	1.000/(1.001)	1.002/(1.002)	1.011
$\widetilde{\sigma }_0$	0.073/(0.073)	0.162/(0.158)	0.069
Θ_4_	0.957/(0.960)	0.986/(0.985)	0.868

The top part of the table contains metrics derived from the genome coverage only, while the bottom part contains metrics derived from the normalized genome coverage. All metrics are defined in the text; BOC stands for breadth of coverage, δ for sequencing depth, and CV for coefficient of variation. The standard deviation is denoted σ. In the bacteria and fungus cases, the running window W is set to 5,001 or 20,001, while for the virus we used 5,001 only. The parameters of the central distribution,$\widetilde{\mu }_0$ and $\widetilde{\sigma }_0$ , and the centralness, Θ4, are reported. Proportion of outliers (1 – Θ4) are about 4.5%, 1.5%, and 13% for the bacteria, fungus, and virus, respectively.

Finally, it is important to note that the *z*-scores assigned to each position on the genome coverage are stable with respect to the *W* parameter. Indeed, as shown in Notebook 7 of [18], the mean and standard deviation of the distribution of the normalized genome coverage $\widetilde{C}$ are not affected by the parameter *W*.

### Genomic ROIs

**Figure 5:**
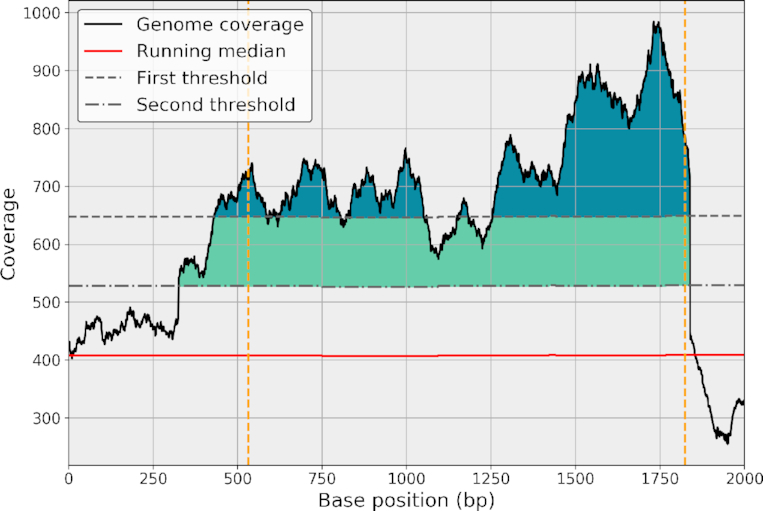
Example of a genomic ROI clustered using a double-threshold method. The genome coverage (black line) and its RM (red) on a short genome location of 2 Kbp. The first threshold (top dashed gray line) alone identifies many short ROIs (dark blue areas). Using a second threshold (bottom dashed gray line), the short ROIs are clustered and identified as a single ROI (colored areas). Yellow vertical lines indicate the beginning and end of the cluster.

Starting from the normalized genome coverage, $\widetilde{C}$, we estimate the parameters of the central distribution. This allows us to set a *z*-score on each genome position. All values above the threshold *n*^+^ are stored into a subset of events denoted $\widetilde{C}_b^+$, and all values below the threshold *n*^−^ are stored into $\widetilde{C}_b^-$. The selected data can be made of continuous or noncontinuous regions. The number of events can be quite large for low thresholds (e.g., for *n*^+^ = 2.5, the bacteria has 35  Kbp such events). However, many positions belong to the same event (i.e., same cluster). Consider the short genomic region in Fig. 5, which is made of 2,000 base positions. It contains five different regions that cross the threshold *n*^+^. Ideally, the five events should be clustered together. To do so, we proceed with a double-threshold approach [31] where a second fixed threshold *m*^+^ is defined as *m*^+^ = α^+^*n*^+^ where α^+^ ≤1 and usually set to 1/2.

In the normalized space, the double-threshold method works as follows. We scan the entire genome coverage vector starting from the first position *b* = 0. As soon as a per-base coverage value crosses the threshold *m*^+^, a new cluster starts. We then accumulate the following bases until the per-base coverage crosses *m* + again (going down). If the maximum of the cluster is above the first threshold, *n*^+^, then the cluster is classified as an ROI . The process carries on until the end of the vector is reached. We repeat this classification for the lower case (with *m*^−^ = α^−^*n*^−^). This method dramatically reduces the number of short ROIs. Finally, we can characterize each region with various metrics such as the length of the region, maximum coverage, or mean coverage. If consecutive data points were independent, we could also report a *z*-score for large events (probability that an event of length N crosses a predefined threshold). Instead, for simplicity, we report the mean and max *z*-score of the event only.

### Impact of the RM window parameter

In order to estimate correctly the general trend of the genome coverage, the RM should cancel out the impact of long deleted, duplicated, or depleted regions. Because the median takes the middle point of a segment as its estimate, the parameter *W* should be set to 2*N* where *N* is the longest atypical genomic region present in the data. For instance, an expected CNV region with a length of 50,000 would imply setting *W* = 100,000 so that the genome coverage trend remains appropriate (see Notebook 6 in [18] for a counter example). Since such regions are not known in advance, *W* should be as large as possible so as to avoid the presence of any long regions that depart from the central distribution. Yet, over-increasing *W* may have undesired effects. For instance, in the extreme case where *W* is set to the full genome length, one would obtain the same value all along the genome (the sequencing depth itself). This could lead to an increase of false detections or missed detections. By default, we recommend setting *W* to 20,000. Indeed, below this value, it seems that there is a slight increase in marginal false detections, while for values in the range *W* = 20,000 to 500,000, the list of ROIs is similar (see Notebook 6 in [18]). As mentioned above, the impact of the *W* parameter on the *z*-scores is marginal, so one can safely change it from 20,000 to 100,000. A strategy could be to run two analysis: one with *W* = 20,000 to list the short events and one with very large *W* for longer events.

## Applications

### Stand-alone and computational time

Although the algorithm described here is quite simple *per se*, each of the three steps requires optimization in order to handle HTS datasets. We provide an implementation within the Sequana project [34], which is a Python library that also provides HTS pipelines based on the workflow management system called Snakemake [35] (Makefile-like with a Python syntax). Stand-alone applications are provided, including sequana_coverage. In addition to the algorithm described above, the stand-alone application has several additional features as explained hereafter. The input file can be either a BAM or a BED file [21] encoded as a three-column tab-delimited file (chromosome, position, coverage). Consider this command:

sequana_coverage --input virus.bed -w 4001 -o

**Figure 6:**
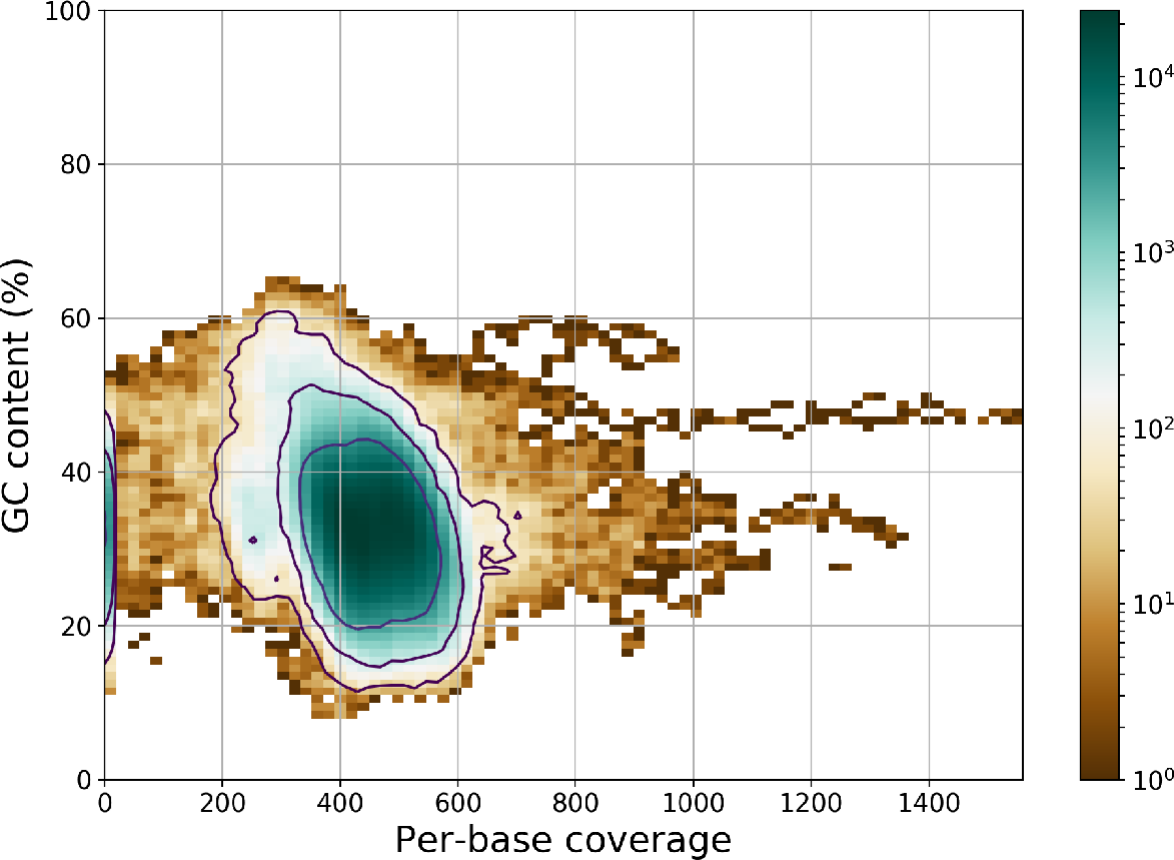
Two-dimensional histogram of the GC content vs coverage available in the HTML reports. The data used correspond to the bacteria test case. We can quickly see that the mean coverage is around 450, the mean GC is around 30%, there is part of the genome coverage with zero coverage (left hand side blue line), and there are low and high ROIs with coverage up to 1,500X that would possibly require more investigations. Be aware of the logarithmic scale; most of the data are indeed centered in the blue area. The brown outliers represent less than a few percentages of the data.

The -*o* option indicates that the input is a circular DNA molecule. The RM window can be tuned using the -*w* option. Several chromosomes may be present (e.g., fungus case). By default, all chromosomes are analyzed, but users can select a specific one using the -*c* option. Other useful options are the ability to change the thresholds on the *z*-score, cluster close ROIs, and analyze the data by chunks (useful for large eukaryote genomes). An additional feature is the ability to download a reference genome (given its ENA [25] accession number). This is achieved internally using BioServices [36], which can switch between the ENA and National Center for Biotechnology Information web services to download the data automatically. Regions of lower genome coverage are sometimes related to repeated content or unusual GC content [37]. Using the reference, we provide a GC content vs coverage plot in the report as shown in Fig. 6. GenBank annotations can also be downloaded to annotate ROIs.

The output is a directory that contains (for each contig/chromosome): an HTML report, a summary file (JSON format), and a comma-separated values (CSV) file with detected ROIs. In addition, we provide a multiQC report [38] via a plugin available in the Sequana library. The multiQC report contains a summary of the mapping metrics, including the DOC and BOC metrics, the number of ROIs, and the centralness (defined in this manuscript). The CSV file is structured with one ROI per row, including information such as the location, length, mean *z*-score, max *z*-score, and mean coverage. In the individual HTML reports, JavaScript plots are provided together with the ROIs for quick inspection (not available for genomes >5 Mbp).

Finally, the stand-alone application is designed to be scalable. The virus genome is analyzed in a few seconds, while the 5-Mbp bacteria genome is analyzed in about 1 minute on a standard computer including analysis and HTML reports (Python implementation). Although the stand-alone was initially designed for bacterial genomes (genome could fit in memory), we extended the functionality so that larger genomes could also be analyzed. In particular, we looked at human genome used in [20]. Although the algorithm is not designed for this lower DOC (around 5X), as the central distribution does not follow a Gaussian distribution, the genome coverage can still be analyzed. Thresholds were increased (from 4 to 6) to avoid an abundance of false detections. The 3.5-Gb genome could be analyzed in a couple of hours (see the conclusion section for details) on a single core. This required adding an option called *binning* that merges data before analysis. Similar to the CNVnator implementation, this reduces the breakpoint accuracy and prevents the tool from identifying short events.

### Example: viral genome characterization

**Figure 7:**
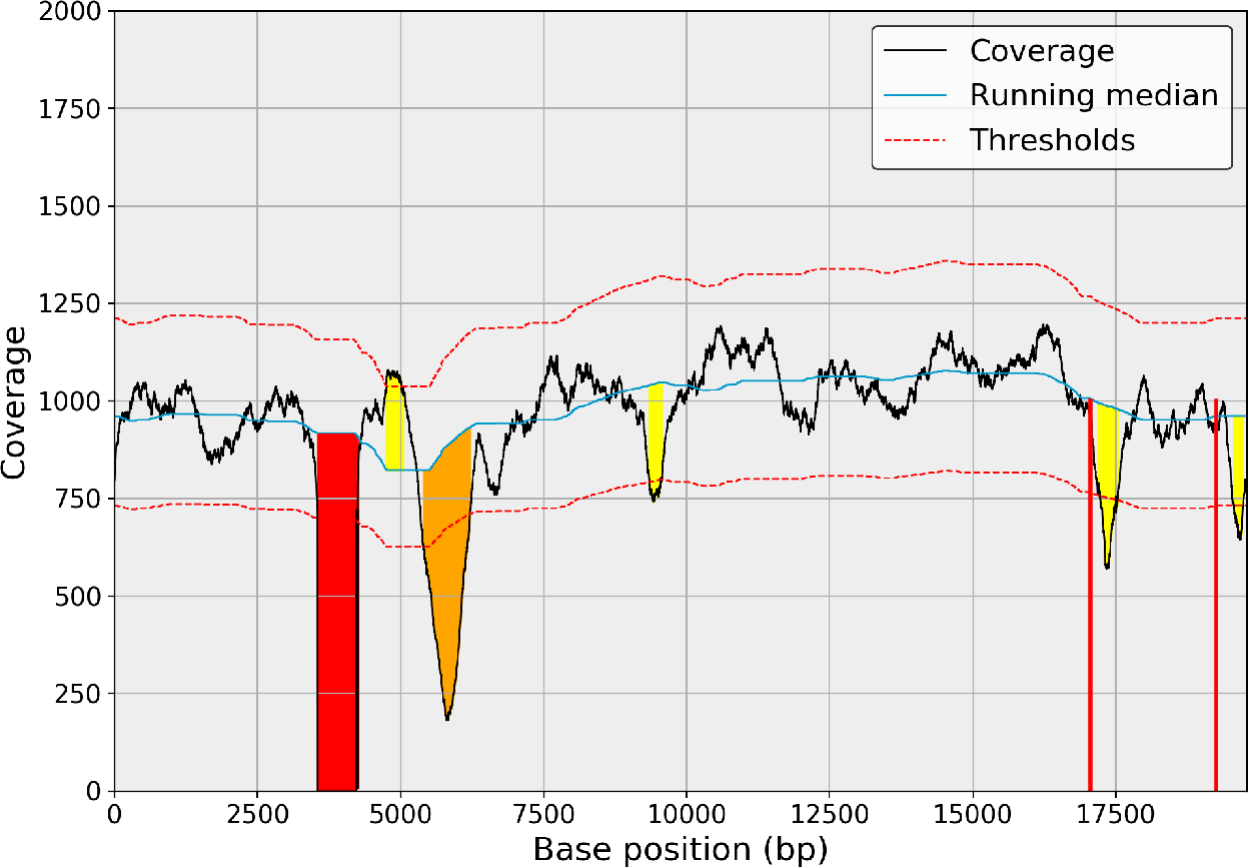
ROIs detected with sequana_coverage. The *W* window parameter was set to 4,000 bases, and circularity was taken into account. We identify nine events: eight depleted regions and one enriched region. The enriched region could be considered as a false positive that appears like a detection due to the presence of two flanking deleted regions. The color code is as follows: red for max *z*-score above 12, orange for max *z*-score between 8 and 12, and yellow for max *z*-score between 4 and 8; gray is for the false-positive event. Using a larger window (e.g., 5,000), the RM would be smoother between the two long deleted events (on the right-hand side); therefore, the false positive would no longer be detected while keeping the eight depleted regions in the list of ROIs.

In this section we illustrate the usage of sequana_coverage on the viral test case (described in the Data Description section). This 18-kb-long genome contains three SNVs (coverage of zero) of length 3, 1, and 1 base with two of them separated by only two bases; two deleted events (700 and 800 bases long); and three short depleted regions with a low signal-to-noise ratio (see Fig. 7 for a visual representation). When running sequana_coverage, the default window parameter is set to 20,000 bases for genomes longer than 100,000 bases. Otherwise, the default value of *W* is set to a fifth of the genome length. Here, it means *W* ∼ 4,000. Taking into account the circularity of the genome, we obtain the results shown in Fig. 7 and Table 2, where nine ROIs are found distributed into eight depleted regions and one enriched region. We emphasize the *z*-score using the following color code: red, orange, and yellow for large, intermediate, and small values, respectively. Table 2 lists the lengths of the ROIs as well as their starting positions. The second ROI (enriched region) can be considered as a false positive, but the eight depleted regions can be considered as true positives. The false positive is due to the presence of two depleted regions that bias the RM estimation and can be avoided by increasing the *W* parameter. For instance, with *W* = 5,000, the enriched region is not detected while keeping the eight depleted regions.

**Table 2:** List of ROIs detected by sequana_coverage, CNVnator, and CNOGpro tools

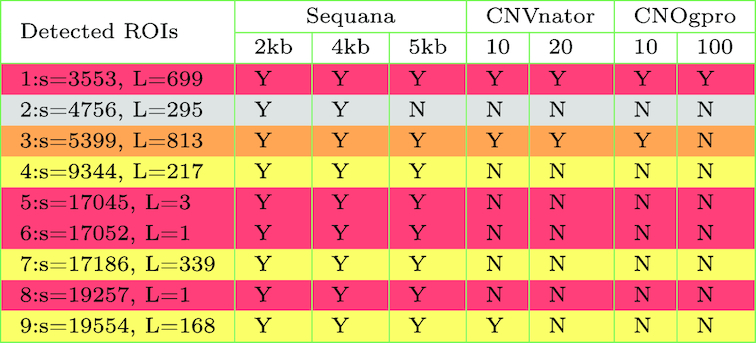

The sequana columns include three analyses with a window parameter W set to 2, 4, and 5 kb. The 4-kb column corresponds to the results shown in Fig. 7. The CNVnator columns include two analyses with a bin parameter set to 10 and 20. The CNOGpro columns include two analyses with a bin parameter set to 10 and 100. The color code is the same as in Fig. 7: red, orange, and yellow for significant, intermediate, and small z-scores. Note that CNVnator and CNOGpro tools have no false-positive results (ROI 2 is not detected). However, none of the ROIs 4 to 9 (short ones) are detected. For each event, we also indicate the starting position (s) and length (L) of the events reported by sequana_coverage.

For comparison, we used CNVnator and CNOGpro tools. Although they are dedicated to the search for CNVs, we were expecting to detect at least the long deleted events (ROIs 1 and 3). As summarized in Table 2, with a bin parameter of 10 or 20, CNVnator detects the two CNV-like events with lengths similar to what is reported by sequana_coverage. No other events were detected (none of the short ones). We obtained similar results with a bin set to 5 (optimal, as explained hereafter), but there are also two short false positives. CNOGpro tool detects the ROI 1, but the ROI 3 is either missed or only partially detected (see Notebook 10 [45]for details). So, despite a marginal false positive, sequana_coverage is able to detect the eight depleted ROIs with sensible length estimation. The results are also robust with respect to the window parameter *W*.

### CNV detection

In extending the functionality of sequana_coverage to include larger genomes, we also explored its ability to detect CNVs.

CNV detection methods can be categorized into five different strategies depending on the input data: paired-end mapping, split-read, read depth (i.e., genome coverage), *de novo* assembly, and combinations of these approaches. Among the numerous tools based on the genome coverage reported in [17], we choose CNVnator [20], which is able to detect CNVs in various sizes ranging from a few hundred bases to mega-bases. CNVnator can also handle whole genome datasets and exhibits a good precision at detecting breakpoints. We also considered a more recent tool, CNOGpro [16], which is dedicated to prokaryotic whole genome sequencing data. As stated in [17], none of the various tools have been able to detect the full spectrum of all types of CNVs with high sensitivity and specificity. To increase the performance in detecting CNVs and reduce false positives, a combinatorial approach could take advantage of the different methods.

**Figure 8:**
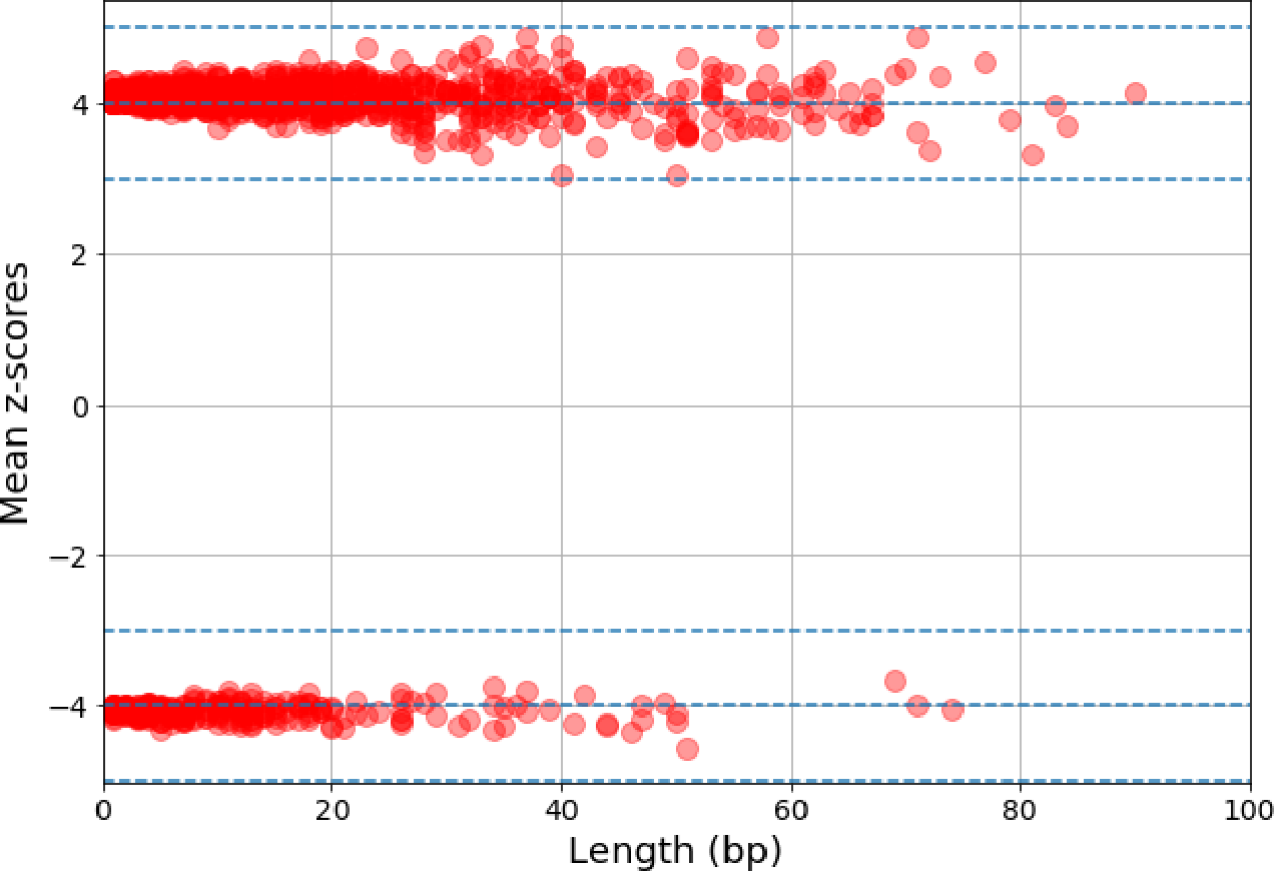
Distribution of ROIs found by analyzing 100 simulated genome coverage data from *Staphylococcus aureus* at a depth of 100X. No features such as CNVs were injected. We plot the mean *z*-score of each ROIs vs its length (in bases). No events have *z*-score above 5 or below -5. All ROI lengths are below 100 bases. For each simulation, an average of 17 ROIs are found.

We first examined the false-positive rate of sequana_coverage on simulated data. Technical details can be found in the Notebook 5 [18]. Simulated paired-end data were used to create 100 genome coverage data for *S. aureus*, each one having a depth of 100X. The number of ROIs detected with sequana_coverage is 17.5 on average (standard deviation of 6). The 1,750 ROIs are plotted in Fig. 8, showing their mean *z*-scores vs lengths. We observed that no *z*-score are above 5 (in absolute value). However, the sizes of the ROIs vary widely, up to 80 bases. Such events are not caused by genuine features in the genome (e.g., high GC, repeats). Indeed, across the 100 independent lists of ROIs, the longest events do not appear at the same location on the reference. They are therefore real false positives. Consequently, in the context of CNVs detection, one should ignore events with mean *z*-score below 5 and length below 100 bases.

We then studied the sensitivity of sequana_coverage by injecting three types of CNVs into the simulated data. First, we deleted 30 nonoverlapping regions (length between 1,000 and 8,000). We achieved a 100% sensitivity. Indeed, all deleted regions were reported with starting and ending position accuracies below 5 bases. Second, we duplicated 80 nonoverlapping regions (same length as above), with a CN = 2. Again, we have a 100% sensitivity with accuracies below 5 bases. The sequana_coverage stores a value called *log2_ratio* for each ROI. This value corresponds to the ratio of the mean coverage and mean RM for that ROI and is equivalent to the CN. The average CN reported for the 80 injected CNVs is 1.96 ± 0.04. Third, we injected a mix of 80 depleted and duplicated events (same length as above) at a coverage of 150X (CN = 1.5) or 50X (CN = 0.5). The 80 events are found with a slightly reduced accuracy (still below 20 bases). The CN reported for duplicated and deleted events is 1.49 ± 0.023 and 0.5 ± 0.026, respectively. The simulated data indicate that the algorithm can detect short CNVs (from 1,000 to 8,000) with high sensitivity and accurate estimate of CN and location. If we set the threshold to a mean *z*-score of 5 and discard events with length below 100 bases, there are no false-positive detections.

**Figure 9:**
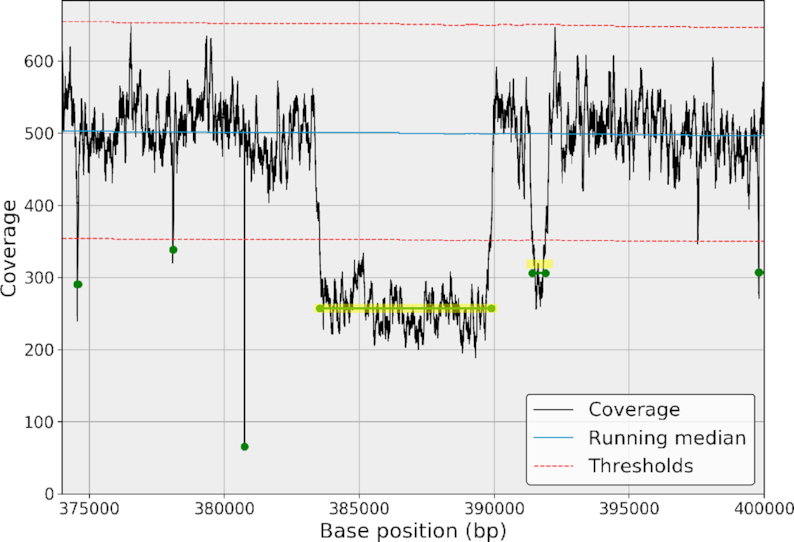
Detection of a depleted region (CN 0.5). CNVnator (thick yellow segments) and sequana_coverage (thin green segments and dots) identifies the 6,300 long event with the correct location and similar CN (based on the mean of the data). The sequana_coverage identifies the other depleted region of about 500 bases at position 392,000. CNVnator's ability to detect that event depends on the bin parameter: missed for a value of 1 or 100, found for a value of 6. All short events (few bases long) are missed by CNVnator. Conversely, CNVnator is able to identify very long CNV regions up to mega-bases.

For a comparison with published tools using real data, we examined the *S. aureus* case used in [20]. We ran sequana_coverage and CNVnator on the 3-Mbp genome. CNVnator has a parameter called *bin*, which is essentially used to define the breakpoint resolution accuracy. We used bin parameters of 1, 6, and 100 (default) where 6 was chosen as the optimal bin size for the sequencing depth considered (500X). Here, we refer to the instructions found in [20] that led to an empirical equation bin = 2,500/DOC (see also Notebook 8 [18]). All results can be found in Notebook 9 in [18]. The number of events reported by CNVnator are 207, 72, and 13 for bin = 1, 6, and 100, respectively. With sequana_coverage, *W* was set to 40,000 bases. The number of reported events is about 600 events (quite stable with respect to the*W* parameter). Only 211 events have a size larger than 10 bases and a mean *z*-score above 5 (47 events have a size larger than 100 bases and a mean *z*-score above 5). All events reported by CNVnator with a bin = 6 or 100 are also detected by sequana_coverage with the same breakpoint resolution. The additional CNVnator events, obtained with bin = 1, are mostly false positives (see Notebook 9 [18] for examples). Visual inspection of events reported by sequana_coverage, but not found by CNVnator, show that they are close to the threshold and appear to be real events (see example in Fig. 9). In terms of computational time, sequana_coverage takes 1 minute to process this 3-Mbp genome, irrespective of *W*, while CNVnator takes about 25 minutes, 5 minutes, and 40 seconds for bin = 1, 6, and 100, respectively.

**Figure 10:**
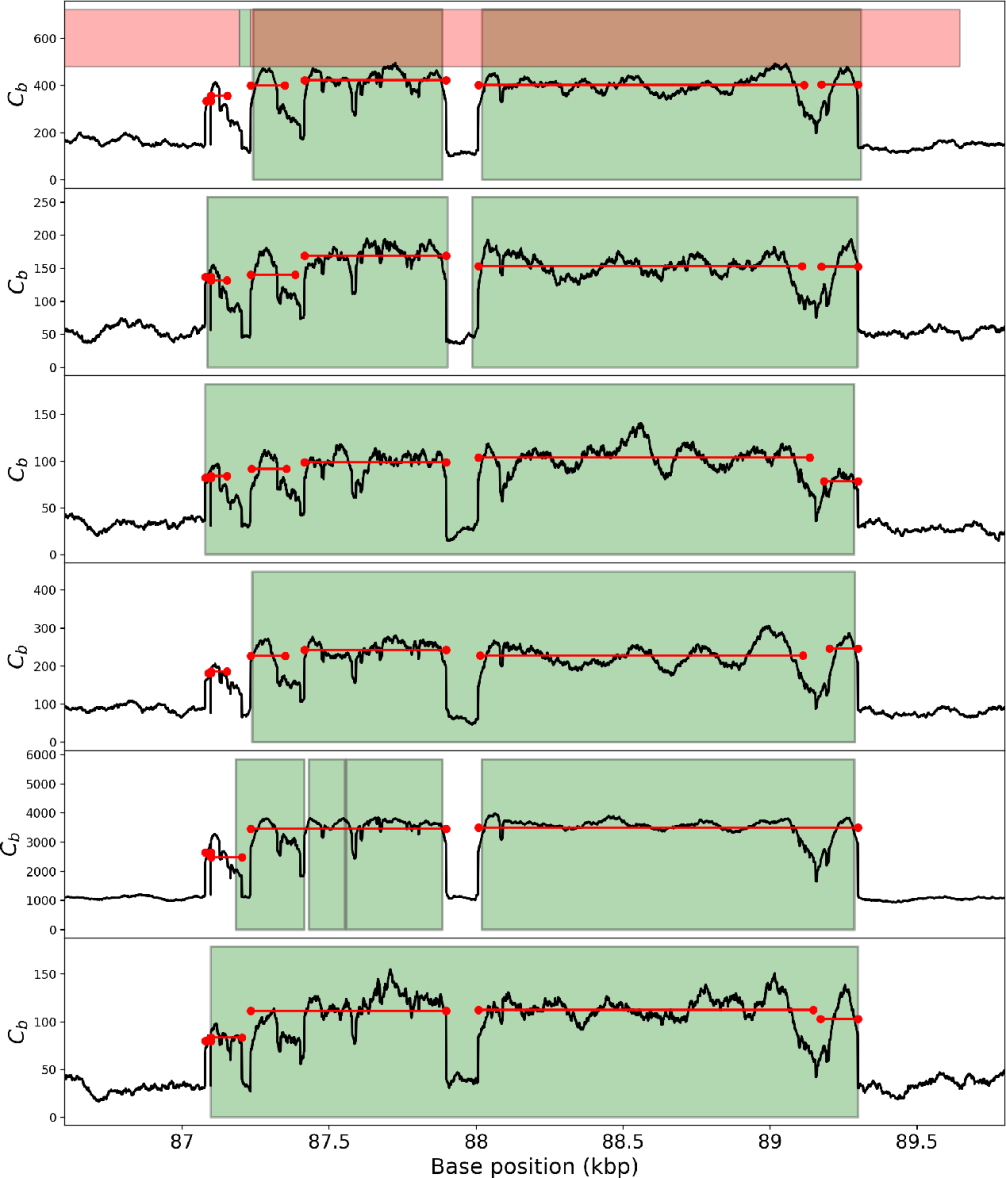
Detection and segmentation of complex events in a population sample. We focus on the region between positions 86,500 and 90,000. We analyze the data (black lines) with sequana_coverage (horizontal red lines) and CNVnator (green areas). We also report the results of CNOGpro (red areas in the top panel only). CNOGpro detects the complex event as a single event with poor breakpoint resolution (end location is offset by 300 bases); see text for an explanation. CNVnator detects one event in three isolates, two events in two isolates) and four events in one isolate (fifth row); the gap in the middle of the genomic region considered is missed in 50% of the cases; breakpoint resolution is high. sequana_coverage reports four to six events; the breakpoint resolution is high; the event in the middle is systematically ignored, as it should be, given its length is about 100 bases.

**Figure 11:**
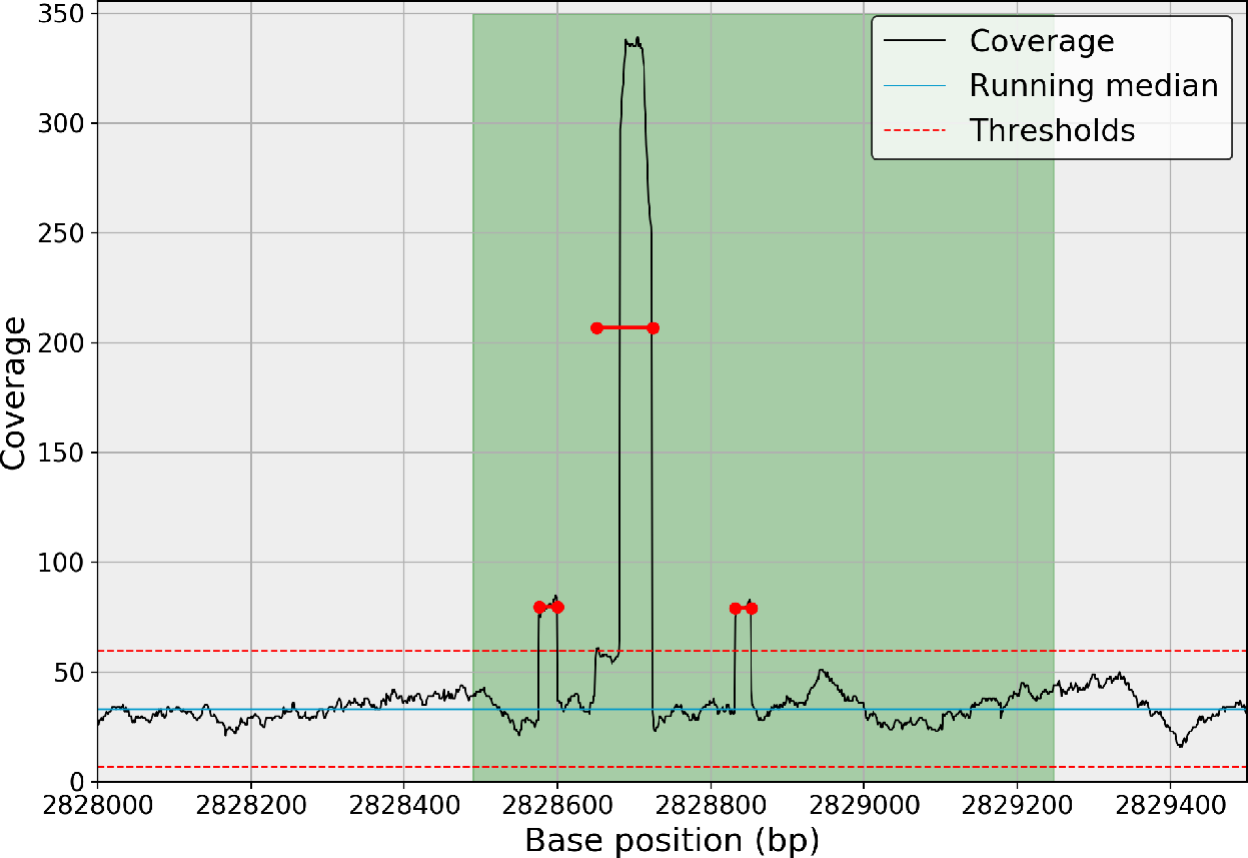
Narrow event made of a strong central peak (CN = 10) and two secondary weak peaks (CN = 2.5). The three peaks can be identified visually in the six isolates. In this plot, we only show the isolate ERR316404. The algorithm designed in sequana_coverage detects the main peak (with CN = 5) and the secondary peaks with CN = 2.5 (red segments). Note that in the six isolates the main peak is always detected, while the secondary peaks are detected in 66% of the cases (8 peaks out of 12). CNVnator does not detect those events in any of the six isolates, probably because the length of those events (irrespective of their strength) are too short. CNOGpro detects one event, shown here as the green area with a CN = 2 for the overall event.

Then, we looked at a population of six isolates of *S. aureus* used in [16]. The six datasets have a wide range of sequencing depth: 165, 61, 36, 94, 1,100, and 34, for the isolate ERR043367, ERR043371, ERR073375, ERR043379, ERR14216, and ERR316404, respectively. We compared the results provided in the supplementary data of [16] with those obtained by running sequana_coverage and CNVnator. In CNOGpro’s supplementary, the authors report 43 CNVs with various CNs. After visual inspection, we believe that seven are false positives and the remaining are confirmed by sequana_coverage. It is important to note that, unlike CNVnator and sequana_coverage, which rely on the data to find the breakpoint of the ROIs, CNOGpro breakpoints are based on annotation and individual genes (or intergenic segments) assuming that duplications and deletions work at the gene level. In Fig. 10, we show an example of a 2-kb-long event present in the six isolates. CNOGpro found this event (same gene position) in the six isolates, similar to CNVnator and sequana_coverage. However, the location of the event reported by CNOGpro is not as precise as the two other tools because it is influenced by the *a priori* knowledge of the gene starting and ending positions. For the same reason, several narrow events found in the same intergenic segment will be averaged together, whereas sequana_coverage reports the events individually, as demonstrated in Fig. 11.

We also ran CNVnator, with its bin parameter set to the optimal value (see above). The detected events found by CNVnator and sequana_coverage (*W* = 40 000) are generally consistent in location and CN. Both tools have a very good breakpoint accuracy, as shown in Fig. 10, with the main difference being that sequana_coverage splits events with a gap in between. Again, CNVnator is optimized to detect long CNV events and may miss narrower events, even if those events have large variations, as shown in Fig. 11.

## Conclusions

The method presented here provides a robust statistical framework to detect under- and overcovered genomic regions that are then further annotated (e.g., length, mean coverage, maximum *z*-score). Although robust, the method is straightforward and can be summarized in three main steps: (1) detrending of genome coverage series using an RM, (2) parameter estimation of the central distribution of the normalized genome coverage series using an EM approach (for a Gaussian mixture model), and (3) clustering and characterization of the outliers as genomic ROI using a double-threshold clustering method.

We underlined the value of the RM algorithm as compared to an MA while emphasizing the practical impact of the RM algorithm complexity. We used an efficient RM algorithm (see Supplementary Materials), which is of paramount importance in the context of HTS analysis. In our implementation, we can take into account the circularity of the molecules as well as multichromosome organisms.

We implemented the method described here within the stand-alone application sequana_coverage, which also provides HTML reports with a summary of the genomic ROIs detected. The HTML reports provide easy visual inspection of genome coverage, a list of genomic ROIs, and statistics such as the centralness, a metric that encompasses the preponderance of the central distribution with respect to the outliers.

We presented test cases with relatively large sequencing depth (30X to 1,000X), although we believe that the algorithm can be used for sequencing depths as low as 10X. A natural extension to this work is to consider sequencing depths below 10X by using a mixture of binomial models instead of Gaussian models.

One obvious application of the algorithm presented is the systematic identification of SNVs or CNVs in a single sample or population of samples. We have shown that sequana_coverage is competitive with dedicated tools such as CNOGpro and CNVnator. We believe that sequana_coverage could be used in a combinatorial approach with existing tools to complement and complete the toolkit of CNV detection.

sequana_coverage is also relatively fast. Viral and bacterial genomes can be analyzed in less than 1 minute. For larger Eukaryotic genomes (human), once the individual BED files are created for each chromosome, the analysis of the 24 human chromosome files should take less than 2 hours (1.5 hours on an HPC cluster using only one core and 1 hour on a DELL Latitude with an SSD hard disk using only one core). The longest chromosome (chr1), with 250 Mb, is analyzed in about 5-6 minutes. A Snakemake [35] pipeline was also recently implemented within Sequana [34] (named Coverage), allowing each chromosome to be analyzed independently. Using 24 cores, we could analyze the 24 chromosomes in about 7 to 8 minutes, which is basically the time needed to analyze the longest chromosome. A graphical interface using Sequanix [39] (a Snakemake graphical user interface) is also available, making the configuration of the parameters and execution of the analysis on a cluster accessible.

With additional features such as the ability to annotate the ROIs with GenBank files and the identification of repeated regions, we believe that the stand-alone application sequana_coverage will help researchers in deciphering the information contained in the genome coverage.

## Availability of source code

- Project name: Sequana (sequana_coverage standalone), version 0.7.0
- Project home page: http://sequana.readthedocs.org
- Operating system(s): Platform independent
- Programming language: Python 3
- Containers: Sequana is available on Bioconda channel [40, 41], and we also provide a Singularity container [42] (version 0.7.0). See http://sequana.readthedocs.org for details.
- License: BSD 3-clause Revised License

## Availability of supporting data

The datasets supporting the results as well as additional files used to created them are available within a Synapse project [29]. More specifically, the BED files mentioned in the Data Description section corresponding to the virus, bacteria, and fungus are available under: doi:10.7303/syn10638370.1 (JB409847.filtered.bed), doi:10.7303/syn10638494.1 (JB409847.filtered.bed), and doi:10.7303/syn10638487.1 (S_pombe.filtered.bed), respectively. In addition, we provide the genome reference used in Fig. 6 (doi:10.7303/syn10638477.1). The datasets are also available on a GitHub repository [18] together with a notebook that reproduces the figures. Finally, note that the BED files can be recreated using the original FastQ files available on doi:10.7303/syn10638358. We also provide recipes to create the BED files from the FastQ files as notebooks in [18]. All notebooks mentioned are available in [18]. Snapshots of the code are also available in the *GigaScience* GigaDB database [43].

## Abbreviations

BAM: binary alignment map; BED: Browser Extensible Data; BOC: breadth of coverage; CN: copy number; CNV: copy number variation; DOC: depth of coverage; EM: expectation maximization; ENA: European Nucleotide Archive; HTS: high-throughput sequencing; MA: moving average; NGS: next-generation sequencing; RM: running median; ROI: regions of interest; SNP: single-nucleotide polymorphism; SNV: single-nucleotide variation.

## Competing interests

The authors declare that have no competing interests.

## Funding

This work has been supported by the France Génomique Consortium (ANR 10-INBS-09-08).

## Author contributions

D.D. and T.C. conceived the study. D.D and T.C. implemented the software. C.B. provided the data. T.C. did the CNV studies. D.D. and T.C. contributed to the initial writing. D.D., T.C., C.B., and S.K. contributed to the final manuscript. All authors contributed to writing and revision and approved the submission.

## Supplementary Material

GIGA-D-17-00238_(Original_Submission).pdf

GIGA-D-17-00238_Revision_1.pdf

GIGA-D-17-00238_Revision_2.pdf

GIGA-D-17-00238_Revision_3.pdf

Response_to_Reviewer_Comments_Original_Submission.pdf

Response_to_Reviewer_Comments_Revision_1.pdf

Response_to_Reviewer_Comments_Revision_2.pdf

Reviewer_1_Report_(Original_Submission) -- Wibowo Arindrarto, M.Sc. 10/16/2018 Reviewed

Reviewer_2_Report_(Original_Submission) -- Rob Patro 10/18/2018 Reviewed

Reviewer_3_Report_(Original_Submission) -- Ian Sudbery 10/25/2017 Reviewed

Reviewer_3_Report_Revision_1 -- Ian Sudbery 3/27/2018 Reviewed

## Figures and Tables

**Figure 12:**
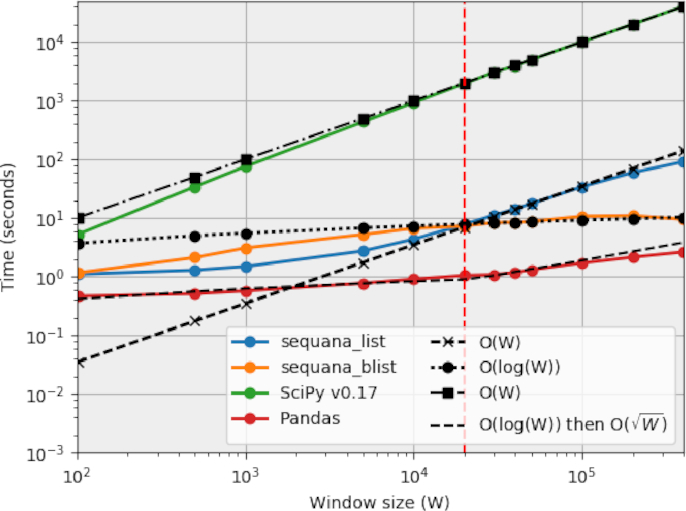
Computational cost of RM algorithms as a function of the window size parameter *W* (for *N* = 1*e*6). Four variants are considered: SciPy [45] implementation (function medfilt v0.17), Pandas [32], and two Python variants available in Sequana based on a list or blist data containers (see text for details). The SciPy variant has a $\mathcal {O}(W)$ complexity irrespective of the *W* value. For low *W* values (*W* <20,000), the two Python variants have $\mathcal {O}(log(W))$ complexity. For larger *W* values, the blist keeps its $\mathcal {O}(log(W))$ complexity while the list container follows a $\mathcal {O}(W)$ complexity. Pandas complexity is less clear with a O(W) for *W* <20,000 and $\mathcal {O}(log(W))$ otherwise. The fastest implementation is clearly the Pandas one, even for large *W* values.

## Appendix: Running median implementation

The mean is a measure of the central tendency of a population. It is not a robust estimator in the presence of large extraneous outliers in the population. In such a situation, it is preferable to consider a truncated mean or a *median* estimator. The median is the middle point of a sample set in which half the numbers are above the median and half are below. More formally, let us consider a sample *s*[*i*], *i* = 1, .., *n* and *S*[*i*], the sequence obtained by sorting *s*[*i*] in ascending order (ordering of equal elements is not important here). Then, the median is defined as

(8) \begin{equation*}
\nu = \rm {median}(\left\lbrace s[1], s[2],..,s[n]\right\rbrace ) = \left\lbrace \begin{array}{rcc}S\left[\frac{n+1}{2}\right] &n\,\,\rm {odd}, \\
\frac{S\left[n/2\right] + S\left[n/2+1\right]}{2} &n\,\,\rm {even}. \end{array}\right.
\end{equation*}

Let us now consider a series *X*(*k*) where *k* = 1, .., *N*. Then, the *running median* (RM) of *X*(*k*) is defined as the sequence $\nu (k)=\rm {median}(\lbrace X(k), X(k+1), .., X(k+W)\rbrace )$, *k* = *W*/2, .., *N* − *W*/2 where *W* is a window size defined by the user and the application. The first *W*/2 and last *W*/2 values are undefined, so we should have *W* ≪ *N*.

Since we perform a sorting of an array of *W* elements at *N* positions, the complexity of the RM is *N* times the complexity of the sorting algorithm. If *W* and *N* are small (e.g., removal of narrow lines in power spectral density in addition to the overall smoothing of time or frequency series [31]), a naive quick-sort algorithm ($\mathcal {O}(W^2)$ in the worst case scenario) may be used. However, better algorithms do exist and can be decreased to $\mathcal {O}(\sqrt{W})$ in the worst case, as implemented in [44]. Yet, in NGS applications, *N* could easily reach several million, and *W* may need to be set to large values up to 50,000 (e.g., to identify long deleted regions).

Instead of computing the median at each position, *k*, a more efficient solution consists of re-using the sorted block at *k* − 1 and maintaining the block as sorted as new elements are added. Indeed, one only needs to insert the next sample into the sorted block and delete the earliest sample from the sorted block. A standard Python module named *bisect* provides an efficient insertion in sorted data (keeping the data sorted). The complexity of this sorting algorithm is $\mathcal {O}(\log W)$.

So far, we have neglected the cost of the insertion and deletion steps, which is not negligible. For instance, in Python language, one of the most common data structures is the *list*. It is a dynamically sized array (i.e., insertion and deletion of an item from the beginning or middle of the list requires moving most of the list in memory), and the look-up, insertion, and deletion have a $\mathcal {O}(n)$ complexity. So, the RM is actually dominated by the slow *O*(*n*) insertion and deletion steps. A better data structure is available thanks to the *blist* package; it is based on a so-called B-tree, which is a self-balancing tree data structure that keeps data sorted. The blist allows searches, sequential access, insertions, and deletions in $\mathcal {O}(\log n)$ (see https://pypi.python.org/pypi/blist/ for details).

Based on materials from http://code.activestate.com/recipes/576930/, we have implemented these two variants of RM functions in Python available in the Sequana [34] library. We also considered established numerical analysis tools from the SciPy [45] and Pandas [32] libraries. We finally compare the four implementations in terms of computation time and complexity, as shown in Fig. 12. It appears that the Pandas implementation is the fastest. For *W* >20,000 up to 200,000, our implementation is 2 to 3 order of magnitude faster than the SciPy version but 4 to 5 times slower than Pandas. We should emphasize the fact that the SciPy function has additional features since it is available for N-dimensional datasets, whereas we restrict ourselves to 1-D datasets. In Sequana, the two variants only differ in the data structure being used to hold the data (list vs blist). Figure 12 shows the difference between the list and blist data structures that is marginal for low *W* values, while for large values asymptotic behaviors are reached, showing the interest of the blist over the list choice. We also see that our implementation with blist has a lower complexity than the Pandas implementation. However, for the range considered, Pandas is always the fastest choice.
