# Supplementary material for: Sequana coverage: detection and characterization of genomic variations using running median and mixture models
Source: Gigascience. 2018 Sep 6;7(12):giy110. doi: 10.1093/gigascience/giy110 (PMC6275460; doi:10.1093/gigascience/giy110)
Supplement: GIGA-D-17-00238_(Original_Submission).pdf [file giy110_giga-d-17-00238_(original_submission).pdf]

GigaScience

Sequana Coverage: Automatic Detection and Characterization of Low and High Genome Coverage Regions.

--Manuscript Draft--

**Manuscript Number:**	GIGA-D-17-00238
**Full Title:**	Sequana Coverage: Automatic Detection and Characterization of Low and High Genome Coverage Regions.
**Article Type:**	Technical Note
**Funding Information:**	Agence Nationale de la Recherche (ANR10-INBS-09-08) — Mr Dimitri Desvillechabrol
**Abstract:**	Background: Genome coverage contains valuable biological information like the presence of repetitive regions or deleted genes. Yet, researchers lack robust tools that account for these non-constant regions and trends in the data. As a consequence, these biologically relevant events have no statistics (e.g. z-score) associated with their detections.<br>Results: We provide a standalone application -- sequana_coverage -- that reports genomic regions of interest (ROI) that are significantly over- or under-represented in NGS sequencing data. Significance is associated with the events as well as characteristics such as length of the regions. The algorithm first detrends the data using an efficient running median algorithm. It then estimates the distribution of the normalized genome coverage with a Gaussian mixture model. Finally, a z-score statistics is assigned to each base position and used to separate the central distribution from the ROIs (i.e., under- and over-covered regions). A double thresholds mechanism is also used to cluster the genomic ROIs. HTML reports provide a summary with interactive= visual representations of the genomic ROIs and standard plots and metrics.
**Corresponding Author:**	Thomas Cokelaer, Ph. D.<br>Institut Pasteur<br>Paris, FRANCE
**Corresponding Author Secondary Information:**	
**Corresponding Author's Institution:**	Institut Pasteur
**Corresponding Author's Secondary Institution:**	
**First Author:**	Dimitri Desvillechabrol
**First Author Secondary Information:**	
**Order of Authors:**	Dimitri Desvillechabrol
	Christiane Bouchier, Ph. D.
	Sean Kennedy, Ph. D.
	Thomas Cokelaer, Ph. D.
**Order of Authors Secondary Information:**	
**Opposed Reviewers:**	
**Additional Information:**	
**Question**	**Response**
Are you submitting this manuscript to a special series or article collection?	No
**Experimental design and statistics**	Yes

Powered by Editorial Manager® and ProduXion Manager® from Aries Systems Corporation

Full details of the experimental design and statistical methods used should be given in the Methods section, as detailed in our Minimum Standards Reporting Checklist. Information essential to interpreting the data presented should be made available in the figure legends.<br><br>Have you included all the information requested in your manuscript?	
**Resources**<br><br>A description of all resources used, including antibodies, cell lines, animals and software tools, with enough information to allow them to be uniquely identified, should be included in the Methods section. Authors are strongly encouraged to cite Research Resource Identifiers (RRIDs) for antibodies, model organisms and tools, where possible.<br><br>Have you included the information requested as detailed in our Minimum Standards Reporting Checklist?	Yes
**Availability of data and materials**<br><br>All datasets and code on which the conclusions of the paper rely must be either included in your submission or deposited in publicly available repositories (where available and ethically appropriate), referencing such data using a unique identifier in the references and in the "Availability of Data and Materials" section of your manuscript.<br><br>Have you have met the above requirement as detailed in our Minimum Standards Reporting Checklist?	Yes

Manuscript

Click here to download Manuscript sequence-coverage-giga.pdf

GigaScience, 2017, 1–10

doi: xx.xxxx/xxxx
Manuscript in Preparation
Technical Note

TECHNICAL NOTE

Sequana Coverage: Automatic Detection and Characterization of Low and High Genome Coverage Regions.

Dimitri Desvillechabrol[1†], Christiane Bouchier[1], Sean Kennedy[1] and Thomas Cokelaer[1,2,†,*]

[1]Institut Pasteur – Pole Biomics – Paris, France and [2]Institut Pasteur – Bioinformatics and Biostatistics Hub – C3BI, USR 3756 IP CNRS – Paris, France

[†]equal contributions
[*]corresponding author
Emails: ddesvillechabrol@gmail.com, christiane.bouchier@pasteur.fr, sean.kennedy@pasteur.fr, thomas.cokelaer@pasteur.fr

Abstract

Background: *Genome coverage* contains valuable biological information like the presence of repetitive regions or deleted genes. Yet, researchers lack robust tools that account for these non-constant regions and trends in the data. As a consequence, these biologically relevant events have no statistics (e.g. z-score) associated with their detections.
Results: We provide a standalone application – *sequana_coverage* – that reports genomic regions of interest (ROI) that are significantly over- or under-represented in NGS sequencing data. Significance is associated with the events as well as characteristics such as length of the regions. The algorithm first detrends the data using an efficient running median algorithm. It then estimates the distribution of the normalized genome coverage with a Gaussian mixture model. Finally, a *z*-score statistics is assigned to each base position and used to separate the central distribution from the ROIs (*i.e.*, under- and over-covered regions). A double thresholds mechanism is also used to cluster the genomic ROIs. HTML reports provide a summary with interactive visual representations of the genomic ROIs and standard plots and metrics.

Key words: genome coverage, sequencing depth, running median, double thresholds clustering, Sequana, NGS, Python

Background

Sequencing technologies allow researchers to investigate a wide range of genomic questions [1], covering research fields such as the expression of genes (transcriptomics) [2], the discovery of somatic mutations, or the sequencing of complete genomes of cancer samples to name a few examples [3, 4]. The emergence of the second generation sequencing, which is also known as Next-Generation Sequencing or NGS hereafter, has dramatically reduced the sequencing cost. This breakthrough multiplied the number of genomic analyses undertaken by research laboratories but also yielded vast amount of data. Consequently, NGS analysis pipelines require efficient algorithms and scalable visualization tools to process this data and to interpret the results.

Raw data generated by NGS experiments are usually stored in the form of *sequencing reads* (hereafter simply called reads). A read stores the information about a DNA fragment and also an error probability vector for each base. Read lengths vary from 35-300 bases for current short-read approaches [1] to several tens of thousands of bases possible with long-read technologies such as Pacific Biosciences [5, 6] or Oxford Nanopore [7].

After trimming steps (quality, adapter removal), most NGS experiments will require mapping the reads onto a genome of reference [8]. If no reference is available, a *de-novo* genome assembly can be performed [9]. In both cases, reads can be

Compiled on: September 21, 2017.
Draft manuscript prepared by the author.

Key Points

- Genome coverage is used to assess the mapping quality of sequencing reads onto a genome reference.
- Biological effects (e.g., origin of replication, repetitive sequence) should be taken into account.
- We propose an algorithm to automatically detect low and highly covered regions of interest (ROI) with a robust statistics using an efficient running median and mixture Gaussian model.
- We provide a standalone application called **sequana_coverage** – available in the **Sequana** [27] project.
- From a BAM or BED file, HTML reports are created with the genome coverage, ROIs, standard metrics (e.g., sequencing depth), coverage versus GC content plot, genbank annotations, etc.
- Javascript visualization ease the introspection of the ROIs and genome coverage.

mapped back on the reference taking into account their quality. We define the *genome coverage* as the number of reads mapped to a specific position within the reference genome. The theoretical distribution of the genome coverage has been thoroughly studied following the seminal work of Lander-Waterman model [10, 11]. A common metric used to characterize the genome coverage is the *sequencing depth*: the empirical average of the genome coverage. It may also be called depth of coverage (DOC) or fold coverage, or confusingly, *depth* or *coverage*. The sequencing depth unit is denoted X. An example of a genome coverage with a sequencing depth of about 450 X is shown in Figure 1. Another useful metric is the breadth of coverage (BOC): the proportion of the intended genome reference covered by at least some sequencing depth.

The required sequencing depth depends on the experimental application. For instance, to detect human genome mutations, single-nucleotide polymorphisms (SNPs), and rearrangements, a 50 X depth is recommended [1] to be able to distinguish between sequencing errors and true SNPs. In contrast, the detection of rarely expressed genes in transcriptomics experiments often requires greater sequencing depth. However, greater sequencing depth is not always desirable. Indeed, in addition to a higher cost, ultra-deep sequencing (large sequencing depth in excess of 1000 X) may be an issue for a *de-novo* genome assembly [12].

The Lander-Waterman model also provides a good theoretical estimate of the required redundancy to guarantee that for instance all nucleotides are covered at least N times. This is, however, a theoretical estimate that does not take into account technological and biological limitations: some regions are indeed difficult to efficiently map (e.g., repetitive DNA). Furthermore, the genome coverage may also contain a non-constant trend or additional sequence not present in the reference. The genome coverage example in Figure 1 demonstrates these different features.

While the sequencing depth metric provides a quick understanding about the quality of the mapping, the genome coverage can be further used to identify regions that are significantly under- or over-covered. Hereafter, these regions of interest (ROI) are denoted low-ROIs and high-ROIs, respectively.

In order to detect low and high-ROIs, a simple and fast approach consists in setting two arbitrary thresholds bounding the sequencing depth. There are two major drawbacks with this approach. First, as shown in Figure 1A, with a fixed threshold, one may detect numerous false signals (type I errors) or fail to detect real events (type II errors). Secondly, the threshold is fixed manually and lacks a robust statistics. An alternative is to estimate the genome coverage profile histogram [13] from which a z-score statistics can be used to identify outliers more precisely. Yet, since the genome coverage may contain low and high frequency fluctuations, the statistics will also suffer from Type I and II errors.

In this paper, we describe an approach that first estimates the genome coverage trend using a running median. It can be employed to normalize the genome coverage vector and calculate a robust statistic (z-score) for each base position. This allows us to obtain robust low and high thresholds at each base position.

In the Data Description section, we describe the data sets used throughout the paper as test-case examples. In the Methods section, we describe (i) the running median used to detrend the genome coverage and (ii) the statistical method used to characterize the central distribution from which outliers can be identified and (iii) a double threshold method proposed to cluster the ROIs. Finally, in the Applications section, we describe the standalone application, *sequana_coverage*, and potential applications for NGS research projects.

Data Description

Three test-cases of genome coverage are presented here, covering representative organisms and sequencing depths. The genome coverage data sets are in BED (Browser Extensible Data) format, a tabulated file containing the coverage, reference (e.g., chromosome number, contig) and position on the reference. BED files can be created using `bedtools` [14], in particular the `genomecov` tool.

We first considered a bacteria from a study of methicillin resistant *Staphilococcus aureus* [15]. One circular chromosome of 3 *Mbp* is present. The sequencing depth is 450 X and the genome coverage exhibits a non-constant trend along the genome (see Figure 1). This pattern, often observed in rapidly growing bacteria, is the result of an unsynchronized population where genome replication occurs bi-directionally from a single origin of replication [16, 17]. The proportion of outliers (see Section Building a statistics for a formal definition; see Table 1) is about 2.5 % of the total bases. The original data sets (Illumina sequencing reads, paired-end, 100 bp) are available at the European Nucleotide Archive (ENA) [18] under study accession number PRJEB2076 (ERR036019). The accession number of the reference is FN433596.

The second organism is a virus with a sequencing depth of 1000 X [19]. A circular plasmid, containing the virus chromosome, is 19 795 bp-long. About 13% of the genome coverage contains large or low coverage regions (outliers). It also contains two large under-covered regions (one partially under-covered and one region that is not covered at all) as shown in Figure 2. The accession number of the reference is JB409847.

The third test case is the fungus (*Schizosaccharomyces pombe*) [20]. The genome coverage has a sequencing depth of 105 *X*. It has three non-circular chromosomes of 5.5 Mbp, 4.5 Mbp and 2.5 Mbp. The references from ENA are CU329670.1, CU329671.1 and CU329672.1 (X54421.1). Although we will look at the first chromosome only (1.5% of outliers), the tools presented hereafter handles circular chromosomes and multiple

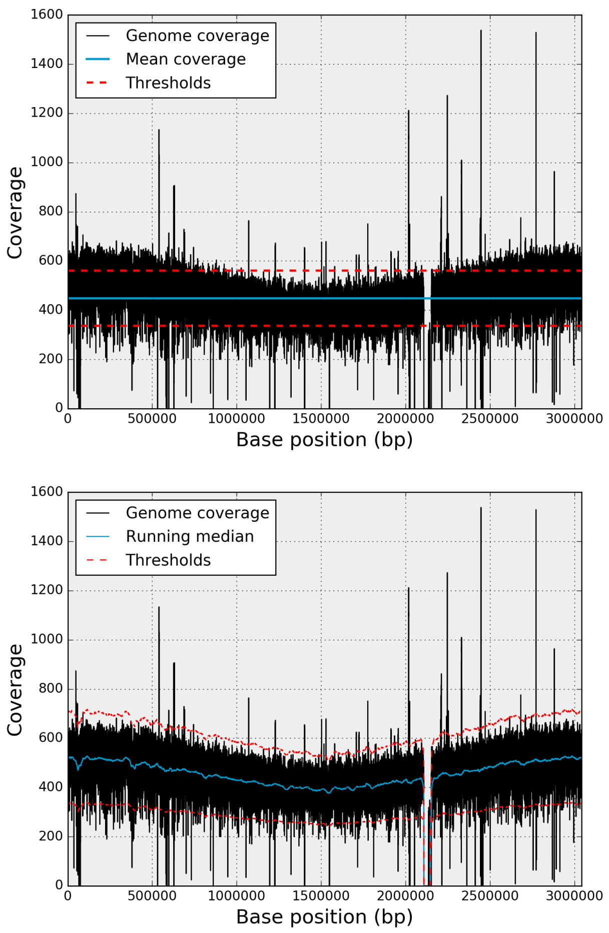

Figure 1. Example of a genome coverage series (in black in both panels). The genome coverage corresponds to the bacteria test case (see text). It contains a deleted region (around 2.2 Mbp) and various under- and over-covered regions (from 100 bp to several Kbp). Although the sequencing depth is about 500 X, there is non-linear trend from 500 X on both ends to 400 X in the middle of the genome. The top panel shows the sequencing depth (blue horizontal line) and two arbitrary fixed thresholds (dashed red lines) at 400 X and 500 X. Due to the non-linear trend, the fixed thresholds lead to an increase of Type I and Type II errors. On the contrary, in the bottom figure, the trend is estimated using a running median (red line) and adaptive lower and upper thresholds (dashed red lines) can be derived.

chromosomes.

We provide the 3 genome coverage data files in BED format on Synapse [21, 22]. See Section Availability of supporting data and materials for more details.

Methods

Detrending the genome coverage

The genome coverage function is denoted $C(b)$ where b is the base (nucleotide) position on the genome of reference. The genome coverage and reference lengths are denoted N. For simplicity, we drop the parentheses and refer to the genome coverage as C_b. The empirical sequencing depth (average of genome coverage) is denoted $\delta = \overline{C_b}$. Ideally, C_b is made of a continuous homogeneous central region. In practice, however, this may be interrupted by a succession of under- and over-covered regions: the genomic ROIs that we want to detect.

A naive classifier consists in setting two fixed thresholds δ^- and δ^+ whereby low and high ROIs are defined as $C_b^- = C_b \leq \delta^-$ and $C_b^+ = C_b \geq \delta^+$, respectively. If C_b^0 denotes the remaining data such that $\delta^- < C_b^0 < \delta^+$, then the genome coverage can be written as $C_b = \{C_b^0, C_b^+, C_b^-\}$.

The advantage of the fixed-thresholds method is that it is conceptually simple and computationally inexpensive. However, there are two major drawbacks manifest. First, as shown in Figure.1-A, false negatives and false positives will increase as soon as there is a non-constant trend present in the data. It may be a low frequency trend as shown here but high frequency trend are also present (see e.g., Fig 2). Also of importance is that an arbitrary choice of threshold(s) is unsatisfactory from a statistical point of view since we cannot associate any level of significance to a genomic region.

In order to account for a possible trend in the genome coverage series (and remove it), a standard method consists in dividing the series by a representative alternative such as its moving average or running median.

The moving average (MA) is computed at each position, b, as the average of W data points around that position and defined as follows:

$$\mathrm{MA_W(b)} = \frac{1}{\mathrm{W}} \sum_{\mathrm{i=-V}}^{\mathrm{V}} \mathrm{C(b+i)}, \quad (1)$$

where W is the length of the moving window (odd number) and $V = (W-1)/2$. Note that the first and last V values are undefined. However, in the case of circular DNA (e.g., virus case), then the first and last V points are defined since C_b is now a circular series.

Similarly, the running median (RM) is computed at each position, b, as the median of W data points around that position:

$$\mathrm{RM_W(b)} = \mathrm{median}(\{\mathrm{C(b-V)}, .., \mathrm{C(b+V)}\}), \quad (2)$$

where W and V are defined as before and the median function is defined as the middle point of the sample set (half of the data is below the median and half is above). A mathematical expression of the median and running median are given in the Appendix section (Eq. 8).

The mean estimator is commonly used to estimate the central tendency of a sample, nevertheless it should be avoided in the presence of extraneous outliers, which are common in NGS genome coverage series (see e.g., Figure 1). Figure 2 shows the impact of outliers when using a moving average or a running mean. We will use the running median only and define the normalized genome coverage as follows:

$$\widetilde{C}_b = \frac{C_b}{RM_W(b)}. \quad (3)$$

We will use the tilde symbol for all metrics associated with the normalized genome coverage, $\widetilde{C}_b$. For instance, $\widetilde{C}_b = \{\widetilde{C}_b^0, \widetilde{C}_b^+, \widetilde{C}_b^-\}$.

The running median is used in various research fields, in particular in spectral analysis [23] to estimate the noise floor while ignoring biases due to narrow frequency bands (e.g., [24]). Here, the goal is to avoid narrow peaks but also to be insensitive to long deleted regions. This can be a major issue in NGS as the running median estimator complexity is a function of the window length. Indeed the running median algorithm involves the sorting of a sample of length W at each position of the genome. So, the running median estimator must be efficient and scalable. This is not an issue in spectral analysis and most fields where running median are used but is a bottleneck for NGS analysis where W is large. As explained in the Appendix section, the complexity of the sorting part is in $O(n^2)$ in the worst case but similarly to the moving average,

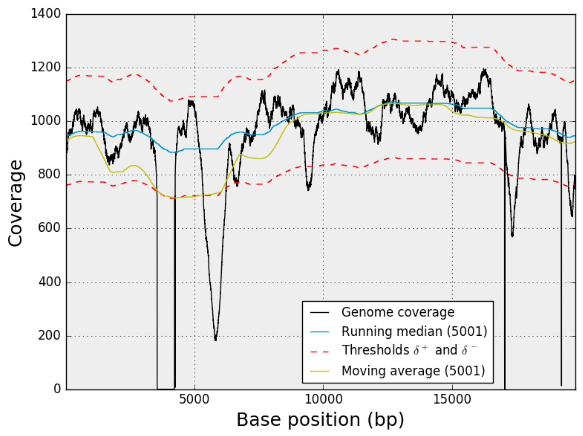

Figure 2. Comparison of the running median and moving average estimators (virus case). The sequencing depth is 930 X and the genome coverage has a deleted region situated around b = 4 000 as well as an under-covered region at b = 6 000. The moving average is less robust to outliers or deleted regions. For instance, the region around b = 5 000 is biased due to the presence of a deleted region, which increases the rate of false alarms.

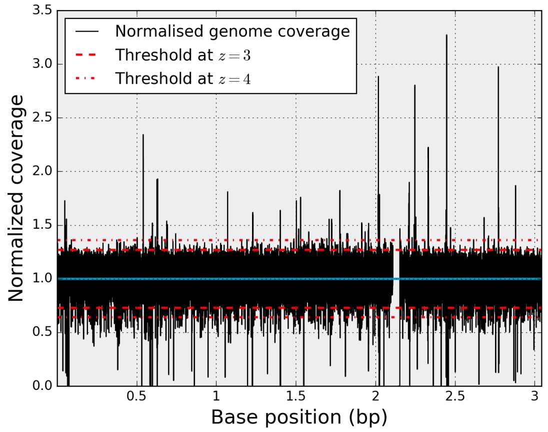

Figure 3. Normalized genome coverage $\widetilde{C}_b$ (bacteria test case). The outliers present in the original genome coverage C_b (see Figure 1) are still present as well as the deleted regions. The distribution is now centred around unity (blue line). Since the distribution is normalized, constant thresholds can be used (dashed lines). See section for details.

one can take advantage of the rolling window and the fact that the previous block is already sorted. We opted for the very efficient Pandas [25] implementation (See Appendix for details). In our implementation, both the moving average and running median have the ability to account for circular DNA data, which is essential to handle circular series.

If we normalize the genome coverage from the bacteria example (Figure 1), we obtain the results shown in Figure 3. Finally, note that the genome coverage being discrete, the running median is also discrete as well as the normalized genome coverage. The discreteness will become more pronounced as sequencing depth decreases.

Building a statistics

Since the reads are randomly generated (uniform distribution over the genome), when reads are mapped to the genome, the per-base coverage follows a Poisson distribution. It is discrete and has one parameter that corresponds to the sequencing depth (mean of the distribution). Yet, the Poisson distribution is often too narrow, as can be observed in the three test cases considered. This is due to biological over-dispersion. In order to account for over dispersion, the Poisson parameter can be distributed according to a second distribution. For instance when the Poisson parameter is distributed according to a Gamma distribution, we obtain a negative binomial, which has two shape parameters [13]. For large sequencing depth, we can approximate the negative binomial or Poisson distributions with a Gaussian distribution. We will use the mathematical notation $\mathcal{N}(\mu, \sigma^2)$ hereafter where μ is the average of the genome coverage (δ in an ideal case) and σ is its standard deviation.

Let us start with an ideal scenario where (i) there is no outliers, (ii) the running median window W is fixed and (iii) $\delta \gg 1$. The latter means that C_b distribution exhibits a Gaussian distribution $\sim \mathcal{N}(\mu, \sigma^2)$. Can we derive the distribution of the normalized genome coverage $\widetilde{C}_b$ knowing that it is a ratio distribution? By definition, the numerator follows a $\mathcal{N}(\mu, \sigma^2)$ distribution while the denominator's distribution is the running median's distribution. The latter is generally not known, especially in the case of large W. Even if we knew the running median distribution, the ratio distribution is only known for two Gaussian distributions X and Y (Cauchy distribution) when (i) the two distributions are centred around zero, which is not the case, and (ii) when they are independent, which is also not the case. Further, the scenario we considered (no outliers, W fixed, $\delta \gg 1$) is too restrictive since we are interested in identifying outliers and may encountered cases where δ is small (for which C_b follows a negative binomial, not a Gaussian distribution).

Our first hypothesis is that $\widetilde{C}_b$ can be decomposed into a central distribution, $\widetilde{C}_b^0$, and a set of outliers, $\widetilde{C}_b^1 = \{\widetilde{C}_b^+, \widetilde{C}_b^-\}$ where the central distribution is predominant: $\left|\widetilde{C}_b^0\right| > \left|\widetilde{C}_b^1\right|$, and where vertical bars indicate the cardinality of the sets.

Our second hypothesis is that the mixture model that represents $\widetilde{C}_b$ is a Gaussian mixture model of $k = 2$ models: $\widetilde{C}_b^0 \sim \mathcal{N}(\widetilde{\mu}_0, \widetilde{\sigma}_0^2)$ and $\widetilde{C}_b^1 \sim \mathcal{N}(\widetilde{\mu}_1, \widetilde{\sigma}_1^2)$. The Gaussianity hypothesis about the central distribution, $\widetilde{C}_b^0$ is valid as long as the raw sequencing depth is large (*i.e.*, at least 10 X). The Gaussianity of the outliers may be questioned, especially for the low-sampling case. However, in the context of a null hypothesis where the central distribution represents the background and the outliers the signal to detect, we can consider that the outliers population is a mix of samples and that we are in the limit of the central theorem. Similarly to the method deployed in [13] to identify a mixture model of negative binomials (on the raw genome coverage), we will use an Expectation Minimization (EM) [26] method to estimate the parameters $\widetilde{\mu}_{0,1}$ and $\widetilde{\sigma}_{0,1}$ (on the normalised genome coverage).

The EM algorithm is an iterative method that alternates between two steps: (i) an Expectation step that creates a function for the expectation of the log-likelihood using the current estimate of the parameters, and (ii) a Minimization step that computes parameters maximizing the expected log-likelihood found in the first step. The likelihood function and the maximum likelihood estimate (MLE) can be derived analytically in the context of Gaussian distributions. Note that in addition to the means and standard deviations, the mixture parameters also need to be estimated. These are denoted $\widetilde{\pi}_0$ and $\widetilde{\pi}_1$. The EM algorithm is standard and can be found in various scientific libraries. Note, however, that the normalized genome coverage may contain zeros in the presence of deleted regions and the estimation of the mixture model should ignore them.

We have applied the EM algorithm on the normalized genome coverage vector on various real NGS data sets including the three test cases Figure 4. The EM retrieves the parameters of the central distribution (in particular $\widetilde{\mu}_0 = 1$) and the outliers.

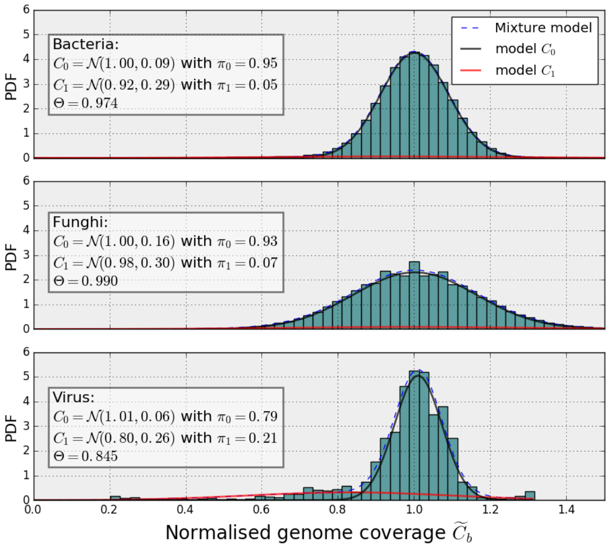

Figure 4. Probability density functions (PDFs) of the normalized genome coverage function concerning the three test cases. The distributions were fitted with a Gaussian mixture models with $k = 2$ models. The first model (black line) fits the central distribution's PDF and the second model (red line close to $y = 0$) fits the outliers' PDF. The dashed lines (close to the black lines) indicates the mixture distribution. In each panel, we report the parameters of the two Gaussian distributions, the proportions π_0, π_1 and the Θ parameter introduced in the text that gives the centralness of the data for each test cases.

Note that the choice of the running median parameter, W, does not significantly affect the parameter estimation. In each case, the mean of the central distribution is very close to unity. The standard deviation varies significantly and is a function of the sequencing depth only (since the outliers are now incorporated in C_b^1). Finally, we can confirm that the proportion of outliers is small as compared to the central distributions by inspection of parameters π_0 and π_1: $\widetilde{\pi}_0 >> \widetilde{\pi}_1$.

Once we have identified the parameters of the central distribution $\widetilde{C}_0$, we can assign statistics for $\widetilde{C}_b$ in terms of z-score:

$$z(b) = \frac{\widetilde{C}(b) - \widetilde{\mu}_0}{\widetilde{\sigma}_0}. \quad (4)$$

Since the z-score corresponds to a normal distribution, we can now set a threshold in terms of tolerance interval within which a specified proportion of the genome coverage falls. For instance, with a threshold of 3, we know from the normal distribution that 99.97% of the sample lies in the range -3 and +3. The exact mathematical value is given by the complementary error function, erfc(x), where $x = n/\sqrt{2}$. Note that for $n = 3$, 4 and 5, the tolerance interval is 99.73%, 99.993% and 99.999942%, respectively. Thus, for a genome of 1 Mbp, by pure chance we should obtain about 2700, 70 and 1 outlier(s), respectively.

If we now replace $\widetilde{C}_b$ in Eq.4 using its expression from Eq. 3, we can express the original genome coverage as a function of the running median, the z-score and the parameters of the central distribution:

$$C(b) = \left(\widetilde{\mu}_0 + z(b)\widetilde{\sigma}_0\right) RM_W(b). \quad (5)$$

We can now set a fixed threshold $z(b) = \pm n$ in the normalized space. This is much easier to manipulate. Moreover, we can derive a variable threshold in the original space that is function of the genome position:

$$\tilde{\delta}^{\pm}(b) = \left(\widetilde{\mu}_0 \pm n^{\pm} \times \widetilde{\sigma}_0\right) RM_W(b). \quad (6)$$

Examples of variable upper and lower threshold functions are shown in Figure 1 and Figure 2 (red dashed lines). This manipulation results in a robust statistical estimate of the presence of outliers in the genome coverage. The z-score, computed earlier, provides a precise level of confidence.

Using the normalization presented above, we can define the **centralness** as one minus the proportion of outliers contained in the genome coverage:

$$\Theta_n = 1 - \frac{\left|\widetilde{C}_b^1\right|}{\left|\widetilde{C}_b\right|} = 1 - \frac{\left|\widetilde{C}_b^1\right|}{G}, \quad (7)$$

where G is the length of the genome, and vertical bars indicate the cardinality. This necessarily depends on how the threshold n is set in the normalized space. In the case of an ideal Gaussian distribution and $n = 3$, the centralness should equal the tolerance interval of a normal distribution $\mathcal{N}(0, 1)$ that is the error function, erf($n/\sqrt{2}$). The centralness equals unity when there are no outliers *i.e.*, $n \to \infty$. Finally, note that the centralness is meaningless for values below 0.5 (meaning that the central distribution is not central!). As shown in Table 1, Θ_3 equals 0.974, 0.99 and 0.86 in the three cases considered (bacteria, fungus, virus). So the proportion of outliers in the virus case is higher than in the two other test cases, which is not obvious at first glance given the very different lengths of the genome considered.

Genomic ROIs

Let us now consider the sub-set of outliers $\widetilde{C}_b^+$. From the previous section, it is defined by positions that are above the fixed threshold n^+ in the normalized space; it is a list of continuous or non-continuous positions; the list may be quite extensive for low threshold (e.g., for $n^+ = 2.5$, the bacteria has 35 Kbp such positions). However, many positions belong the same event (*i.e.*, same cluster). Considering the short genome region in Figure 5, which is made of 2000 base positions. It contains 5 different regions that cross the threshold n^+. However, only one is well above. Ideally, the 5 events should be clustered together. To do so, we proceed with a double-threshold approach [24] where a second fixed threshold m^+ is defined as $m^+ = \alpha^+ n^+$ where $\alpha^+ \leq 1$ and usually set to 1/2.

In the normalized space, the double threshold method works as follows; We scan the entire genome coverage vector starting from the first position $b = 0$. As soon as a per-base coverage value crosses the threshold m^+, a new cluster starts. We then accumulate following bases until the per-base coverage crosses $m+$ again (going down). If the maximum of the cluster is above the first threshold, n^+, then the cluster is classified as a region of interest. The process carries on until the end of the vector is reached. We repeat this classification for the lower case (with $m^- = \alpha^- n^-$). This method dramatically reduces the number of short ROIs. Finally, we can characterize each region with various metrics such as the length of the region, maximum coverage, mean coverage, mean and maximum z-scores.

Applications

Although the algorithm described is quite simple *per se*, each of the three steps need to be optimised to handle NGS data sets. We provide an implementation within the `Sequana` project [27]. `Sequana` is a Python library that provides NGS pipelines in the form of *snakefiles* based on the workflow management sys-

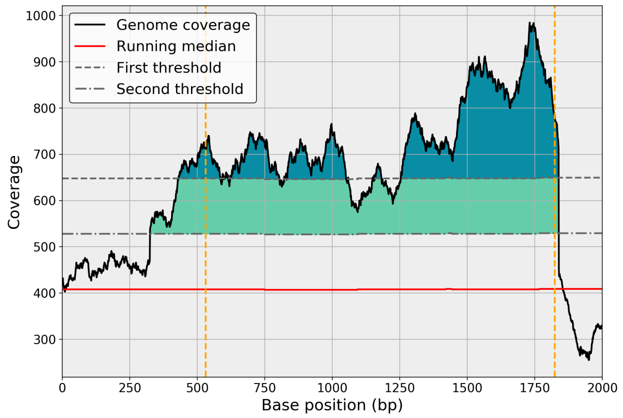

Figure 5. Example of a genomic region of interest (ROI) clustered using a double threshold method. The genome coverage (black line) and its running median (red) on a short genome location of 2kbp. The first threshold (top dashed gray line) alone identifies many short ROIs (dark blue areas). Using a second threshold (bottom dashed gray line), the short ROIs are clustered and identified as a single ROI (coloured areas). Yellow vertical lines indicates the beginning and end of the cluster.

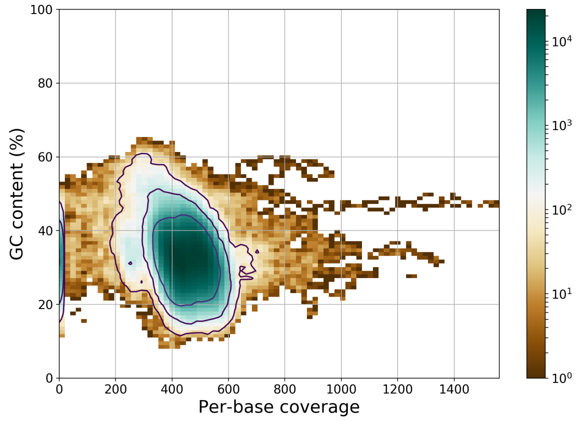

Figure 6. 2-dimensional histogram of the GC content versus coverage available in the HTML reports. The data used correspond to the bacteria test case. We can quickly see that (i) the mean coverage is around 450, (ii) the mean GC is around 30 % (iii) there are part of the genome coverage with zero coverage (left hand side blue line), (iv) there are low and high ROI with coverage up to 1500X that would possibly require more investigations. Be aware of the logarithmic scale: most of the data is indeed centered in the blue area and the brown outliers represent less than a few percents of the data.

Metric	Bacteria	Fungus	Virus
Genome length	3 Mbp	5.5Mbp	19795
BOC	0.985	1.0	0.966
mean δ	447.8	105.49	931.3
median δ	453	105	988
σ	84.1	19.9	237.2
CV	0.19	0.19	0.25
W	5001 / (20001)	5001 / (20001)	5001
$\widetilde{\mu}_0$	1.000 / (1.001)	1.002 / (1.002)	1.011
$\widetilde{\sigma}_0$	0.073 / (0.073)	0.158 / (0.161)	0.069
Θ_3	0.976 / (0.967)	0.986 / (0.986)	0.868

Table 1. Metrics derived from the genome coverage of the three test cases considered (Bacteria, Fungus, Virus). The top part of the table contains metrics derived from the genome coverage only, while the bottom part contains metrics derived from the normalized genome coverage, $\widetilde{C}_b$. All metrics are defined in the text; BOC stands for breadth of coverage, δ for sequencing depth, CV for coefficient of variation. The standard deviation is denoted σ. In the bacteria and fungus cases, the running window W is set to 5 001 or 20 001 while for the virus we used 5 001 only. The parameters of the central distribution, $\widetilde{\mu}_0$ and $\widetilde{\sigma}_0$ and the *centralness*, Θ_3 are reported. Proportion of outliers (1-Θ_3) are 2.5, 1.5 and 14% for the bacteria, fungus and virus, respectively.

tem called Snakemake [28] (Makefile-like with a Python syntax). `Sequana` also provides a Python library with re-usable blocks. Moreover, we provide independent standalone applications. One of them is called `sequana_coverage`; it includes the different features related to genome coverage exposed in this paper. The standalone `sequana_coverage` provides a self-explanatory help and below we demonstrate how to generate an HTML report from a BED file. The BED file format is a data structure that stores the genome coverage information [14] (3-columns tab-delimited file).

```
sequana_coverage --input virus.bed -w 4001 -o
```

Several chromosomes may be present (e.g., fungus case). By default, the first chromosome is used but one can provide the chromosome number using the *-c* option. The *-o* option indicates that the input is made of a circular DNA. The running median window can be tuned using *-w* option. Full details are available using *--help*. An HTML report is created by default in the `./report` directory. In the case of regions or genomes larger than 0.5 Mbp, independent `JavaScript` pages are created for each 0.5 Mbp-long region. This was done to optimize browsing and analysis of larger data sets. A list of genomic regions are available as HTML tables but also as downloadable CSV files. An additional feature is the ability to download a reference genome (given its ENA [18] accession number). This is achieved internally using BioServices [29] that can switch between the ENA or NCBI web services to download the data automatically. This is particularly useful to further compare the genome coverage with other characteristics (e.g., the GC content of the reference). Finally, the standalone application is designed to be scalable: the virus case takes a few seconds while the 5 Mbp bacteria case takes about one minute on a standard computer including analysis and HTML reports (Python implementation).

Here is a non exhaustive list of applications followed by a few illustrative examples.

- Quickly check the quality of the mapping. This can be performed visually in the HTML reports. However, one can also use the statistics provided such as the **centralness** metric. For instance, the reported value of 0.873 (below unity) clearly indicates an issue in the Virus test-case example where 13% of the genome being is under-covered. This metric can also be used to directly compare the quality of different mappings.
- Associate a statistic (z-score) on each value of the genome coverage. Again, because of the statistics used, one can compare different mapping strategies more precisely.
- Automatic and robust detection of all under or over covered ROIs. The CSV files provided may be used for further classification using machine learning tools. For instance the width of the ROIs and/or the maximum amplitude may be used as features to characterise the anomalies in the genome coverage.
- Effect of the GC content on the coverage. Regions of lower genome coverage are sometimes related to repeated content or unusual GC content [30]. We provide a GC content versus coverage plot in the report as shown in Figure 6, which can be used to detect this effect.
- Annotation of the ROIs, if an annotated data file is provided (genbank). Again, this can be downloaded via the standalone application. The interest of the annotations is the

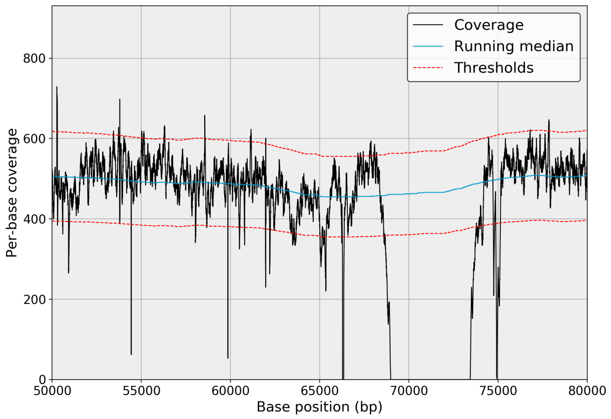

Figure 7. Focus on the bacteria test-case genome coverage. We show the genome coverage in the range 50000–85000 base position. This range contains a few instances of anomalies: short drop of coverage at position 54431, 59852, 61955, 66278, 74731, long deleted area (68809–68809+4789) and short excess of coverage at position 53787 and 58552. If those anomalies can be explained will have more confidence in the mapping that looks suspicious at first glance. It appears that the long deleted region is a repeated one (IS431), amongst the 5 short drops the last four are known CDS regions. As or the excess, the second one is a CDS as well.

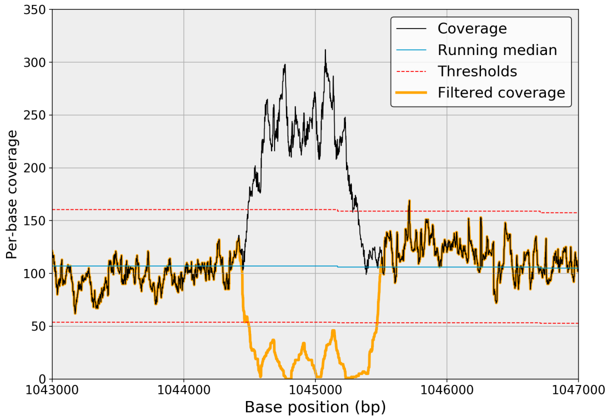

Figure 8. Identification of repeated regions (from highly covered regions). We can concatenate a filtered genome coverage in the BED file (as the fourth column) and compare the unfiltered and filtered genome coverage. Here, a over-represented ROI is detected above the threshold. Using the filtered genome coverage (green line), it appears that the mapping is of poor quality indicating a possibly of repeated regions.

ability to associate an ROI with a known annotation as illustrated in Figure 7. This allows researchers to be more confident in interpreting NGS data. For instance, a deleted or poorly covered regions may be a within an annotated repeated region.

- Identification of repeated regions. In addition to the genbank annotations that may be useful to understand anomalies in the coverage, one can also take advantage of the mapping quality information. Indeed, the BED file may contain two coverage vectors. The first one contains the standard, unfiltered, mapping data that we have address thus far. The second one is the filtered coverage where reads with poor quality (as given by tools such as BWA [8]) have been removed. With this technique, one might associate anomalous over-represented ROIs with poor quality mapping (See Figure 8) or associate regions that looks normal with annotations or repeated regions (See Figure 9).

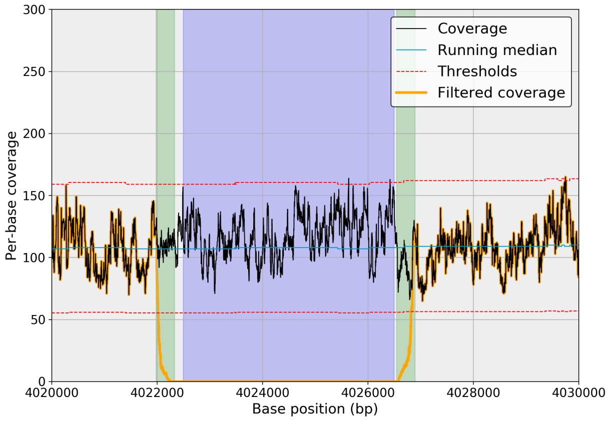

Figure 9. Same as Figure 8. Here, the unfiltered coverage (black line) appears normal. However, using the filtered coverage (orange), a large regions with poor mapping is revealed. It appears that this 4 000 bp regions (blue) corresponds to a retro-transposable element flanked by two repeated regions (light green), which is confirmed by the Genbank annotations.

Conclusion

The genome coverage along a reference contains valuable information and deserves to be part of an NGS toolkit (e.g., quality control of an alignment before a variant detection pipeline). Yet, it is too often summarised by its sequencing depth even though the raw data usually contains a wide spectrum of features such as deleted regions, low frequency trends, non-homogeneous central distribution, repeated regions, . . .

The method presented in this paper provides a robust statistical framework to detect under and over-covered genomic regions that can be further characterized with basic statistics (length, mean coverage, maximum z-score, . . .). Although robust, the method remains simple and can be summarized in three main steps: (1) detrending of genome coverage series using a running median (ii) parameter estimation of the central distribution of the normalized genome coverage series using an EM approach (for a Gaussian mixture model), (iii) clustering and characterization of the outliers as genomic regions of interest (ROI).

We underlined the value of the running median algorithm as compared to a moving average while emphasizing the practical impact of the running median algorithm complexity. Indeed, an efficient implementation is of paramount importance in the context of NGS analysis. In addition, circular series and multi-chromosome organisms should be handled. We wrap the algorithm within the standalone application `sequana_coverage`, which also provides HTML reports with a summary of the genomic regions of interest. The HTML reports provide visual introspection of the genome coverage, list of genomic ROIs and statistics such as the centralness, a metric that encompasses the preponderance of the central distribution with respect to the outliers.

Although we presented test cases with relatively large sequencing depth (100X to a thousand), it is based on a robust statistics and practical cases down to 30X were studied with success. We believe that the algorithm can be used to sequencing depth as low as 10X. Below 10X, a Gaussian distribution hypothesises is not valid anymore and the z-score values are less precise. A natural extension to this work is to consider low sequencing depths below 10X.

With additional features such as the ability to annotate the ROIs with genbank files and the identification of repeated regions, we believe that the standalone application `sequana_coverage` will help researchers in deciphering the infor-

mation contained in the genome coverage. Finally, notebooks, examples and code available in Sequana [27] should be helpful for integration in other libraries.

Availability of source code

- Project name: Sequana (sequana_coverage standalone)
- Project home page: http://sequana.readthedocs.org
- Operating system(s): Platform independent
- Programming language: Python 3
- Containers: Sequana is available on Bioconda channel [35, 36] and we also provide a Singularity container [37]. See http://sequana.readthedocs.org for details.
- License: BSD 3-clause Revised License

Availability of supporting data and materials

The data sets supporting the results as well as additional files used to created them are available within a Synapse project [22]. More specifically, the BED files mentioned in Section Data Description corresponding to the virus, bacteria and fungus are available under: doi:10.7303/syn10638370.1 (JB409847.filtered.bed), doi:10.7303/syn10638494.1 (JB409847.filtered.bed) and doi:10.7303/syn10638487.1 (S_pombe.filtered.bed), respectively. In addition, we provide the genome reference used in Figure 6 (doi:10.7303/syn10638477.1) and the genbank used in Figure 9 (doi:10.7303/syn10638480.1). The data sets are also available on a Github repository [38] together with a notebook that reproduces the figures. Finally, note that the BED files can be recreated using the original FastQ files available on doi:10.7303/syn10638358. We also provide recipes to create the BED files from the FastQ files as notebooks in [38].

Declaration

List of abbreviations

- BAM: Binary Alignment Map, the binary version of the Sequence Alignment Map (SAM) format.
- BED: Browser Extensible Data
- BOC: Breadth of Coverage
- DOC: Depth of Coverage
- CV: Coefficient of Variation
- EM: Expectation Minimization
- MA: Moving Average
- MLE: Maximum Likelihood Estimate
- RM: Running Median
- ROI: Regions of Interest, samples within a data set identified for a particular purpose.
- SNP: Single Nucleotide Polymorphisms

Competing Interests

All authors have no conflicts of interest to this manuscript.

Funding

This work has been supported by France Génomique consortium ANR10-INBS-09-08.

Author's Contributions

D.D. and T.C. conceived the study. D.D and T.C. implemented the software. C.B. provided the data. D.D. and T.C. contributed to the initial writing. C.B and S.K contributed to the final manuscript. All authors contributed to writing and revision and approved the submission.

Acknowledgements

We are grateful to Nicolas Escriou (Institut Pasteur) for providing the FastQ and reference of the Virus test case. We are also grateful to Benoit Arcangioli (Institut Pasteur) and Serge Gangloff (Institut Pasteur) for providing the FastQ files and reference of the *S. Pombe* test case.

References

1. Goodwin, S., *et al.* (2016) Coming of age: ten years of next-generation sequencing technologies. *Nature Reviews Genetics*, **17**(6), 333-351.
2. Wang, Z. *et al.* (2009) RNA-Seq: a revolutionary tool for transcriptomics. *Nature reviews genetics*, **10** (1), 57-63.
3. Meyerson, M. *et al.* (2010) Advances in understanding cancer genomes through second-generation sequencing. *Nature Reviews Genetics*, **11**(10), 685-696.
4. Iorio, F. *et al.* (2016) A Landscape of Pharmacogenomic Interactions in Cancer. *Cell*, **166**(3), 740-754.
5. Eid, J. *et al.* (2009) Real-time DNA sequencing from single polymerase molecules. *Science* **323**, (5910) 133-138.
6. Lee, H. *et al.* (2004) Error correction and assembly complexity of single molecule sequencing reads. *BioRxiv*, 006395.
7. Eisenstein, M. (2012) Oxford Nanopore announcement sets sequencing sector abuzz *Nat. Biotechnology* **30**(4), 295-296
8. Li, H. (2013) Aligning sequence reads, clone sequences and assembly contigs with BWA-MEM. *arXiv preprint* arXiv:1303.3997.
9. Bankevich, A. *et al.* (2012) SPAdes: a New genome assembly algorithm and its applications to single-cell sequencing. *J. Comput. Biol.* **19**(5): 455-477.
10. Lander, E.S. and Waterman, M.S. (1988) Genomic mapping by fingerprinting random clones: a mathematical analysis. *Genomics*, **2**(3), 231-239.
11. Wendl, M.C. and Barbazuk, W.B. (2005) Extension of Lander-Waterman theory for sequencing filtered DNA libraries. *BMC Bioinformatics*, **6**(1):245
12. Mirebrahim, H. *et al.* (2015) De novo meta-assembly of ultra-deep sequencing data. *Bioinformatics*, **31**(12), i9-i16.
13. Lindner, M.S. *et al.* (2013) Analyzing genome coverage profiles with applications to quality control in metagenomics. *Bioinformatics*, **29**(10) 1260-1267.
14. Quinlan, A.R. and Hall, I.M., (2010). BEDTools: a flexible suite of utilities for comparing genomic features. Bioinformatics. **26**, 6, pp. 841-842. http://bedtools.readthedocs.io
15. Tong, S.Y. *et al.* (2015) Genome sequencing defines phylogeny and spread of methicillin-resistant Staphylococcus aureus in a high transmission setting. *Genome Res.*, **25**(1), 111-118.
16. Bremer, H. Churchward, G (1977) An examination of the Cooper-Helmstetter theory of DNA replication in bacteria and its underlying assumptions. *Journal of Theoretical Biology*, **69**(4): 645-654
17. Prescott, D.M. and Kuempel, P.L., (1972) Bidirectional

replication of the chromosome in Escherichia coli. *Proceedings of the National Academy of Sciences*, **69**(10): 2842-2845.

18. European Nucleotide Archive (ENA). http://www.ebi.ac.uk/ENA. Accessed 8 Sept 2017.
19. Combredet, C. *et al.* (2003), A molecularly cloned Schwarz strain of measles virus vaccine induces strong immune responses in macaques and transgenic mice. *J. Virol.*, **77**(21): 11546-11554
20. Wood, V. *et al.*, (2002) The genome sequence of Schizosaccharomyces pombe. *Nature* **415**(6874), 871-880.
21. Sages's Synapse platform https://www.synapse.org. Accessed 8 Sept 2017.
22. Supporting materials on Synapse project page (BEDs, FastQs, Genome references and genbanks). http://dx.doi.org/doi:10.7303/syn10638358. Accessed 8 Sept 2017.
23. Percival, D.B. and Walden, A.T. (1993) Spectral analysis for physical applications. Cambridge University Press.
24. Balasubramanian, R. *et al.* (2005) GEO 600 online detector characterization system. *Classical Quant. Grav.*, **22**(23), 4973-4986.
25. McKinney, W. Data Structures for Statistical Computing in Python, Proceedings of the 9th Python in Science Conference, 51-56 (2010)
26. Dempster, A.P. and Laird,N.M., and Rubin,D.B. (1977). Maximum likelihood from incomplete data via the EM algorithm. *Journal of the royal statistical society. Series B (methodological)* **39**(1) 1-38.
27. Cokelaer, T. and Desvillechabrol, D. and Legendre, R. and Cardon, M. (2017) Sequana: a Set of Snakemake NGS pipelines. *The Journal of Open Source Software*, **2**, 16 https://doi.org/10.21105/joss.00352. Accessed 8 Sept 2017.
28. Köster, J., and Rahmann, S. (2012). Snakemake - a scalable bioinformatics workflow engine. Bioinformatics, **28**(19), 2520-2522.
29. Cokelaer, T. *et al.*(2013). BioServices: a common Python package to access biological Web Services programmatically. Bioinformatics, **29**(24), 3241-3242.
30. Dohm, J.C. and Lottaz, C. and Borodina, T. and Himmelbauer, H. (2008) Substantial biases in ultra-short read data sets from high-throughput DNA sequencing. *Nucleic Acids Res.* **36**(16): e105
31. Mohanty, S.D. (2002). Median based line tracker (MBLT): model independent and transient preserving line removal from interferometric data. *Class. Quantum Grav.*, **19**(7): 1513-1519.
32. Jones, E. and Oliphant, T. and Peterson, P. et al. (2001) SciPy: Open source scientific tools for Python.
33. Mokry, M. *et al* (2010) Accurate SNP and mutation detection by targeted custom microarray-based genomic enrichment of short-fragment sequencing libraries. *Nucleic Acids Res.* **38**(10) e116
34. Sims, D. *et al.* (2014) Sequencing depth and coverage: key considerations in genomic analyses. *Nature Reviews Genetics*, **15**(2), 121-132.
35. Conda: Package, dependency and environment management for any language. https://conda.io/docs. Accessed 8 Sept 2017.
36. Bioconda is a channel for the conda package manager specializing in bioinformatics software. http://bioconda.github.io/. Accessed 8 Sept 2017.
37. Kurtzer, G.M and Sochat, V. and Bauer, M.W. (2017) Singularity: Scientific containers for mobility of compute. PLoS One. **12**(5).
38. The Sequana resources GitHub repository. https://github.com/sequana/resources. Accessed 8 Sept 2017.

Appendix

Running median implementation

The mean is a measure of the central tendency of a population. It is not a robust estimator in the presence of large extraneous outliers in the population. In such a situation, it is preferable to consider a truncated mean or a *median* estimator. The median is the middle point of a sample set in which half the numbers are above the median and half are below. More formally, let us consider a sample $s[i], i = 1, .., n$ and $S[i]$ the sequence obtained by sorting $s[i]$ in ascending order (ordering of equal elements is not important here). Then, the median is defined as

$$\nu = \text{median}(\{s[1], s[2], .., s[n]\}) = \begin{cases} S\left[\frac{n+1}{2}\right] & n \text{ odd}, \\ \frac{S[n/2]+S[n/2+1]}{2} & n \text{ even}. \end{cases} \quad (8)$$

Let us now consider a series $X(k)$ where $k = 1, .., N$. Then, the *running median* of $X(k)$ is defined as the sequence $\nu(k) = \text{median}(\{X(k), X(k+1), .., X(k+W)\})$, $k = W/2, .., N - W/2$ where W is a window size defined by the user and the application. The first $W/2$ and last $W/2$ values are undefined so we should have $W \ll N$.

Since we perform a sorting of an array of W elements at N positions, the complexity of the running median is N times the complexity of the sorting algorithm. If W and N are small (e.g., removal of narrow lines in power spectral density in addition to the overall smoothing of time or frequency series [24]), a naive quick-sort algorithm ($\mathcal{O}(W^2)$ in the worst case scenario) may be used. However, better algorithms do exist and can be decreased to $\mathcal{O}(\sqrt{W})$ in the worst case as implemented in [31]. Yet, in NGS applications, N could easily reach several millions and W may need to be set to large values up to 50,000 (e.g., to identify long deleted regions).

Instead of computing the median at each position, k, a more efficient solution consists in re-using the sorted block at $k - 1$, and to maintain the block sorted as new elements are added. Indeed, one only needs to *insert* the next sample into the sorted block and *delete* the earliest sample from the sorted block. A standard Python module named *bisect* provides an efficient insertion in sorted data (keeping the data sorted). The complexity of this sorting algorithm is $\mathcal{O}(\log W)$.

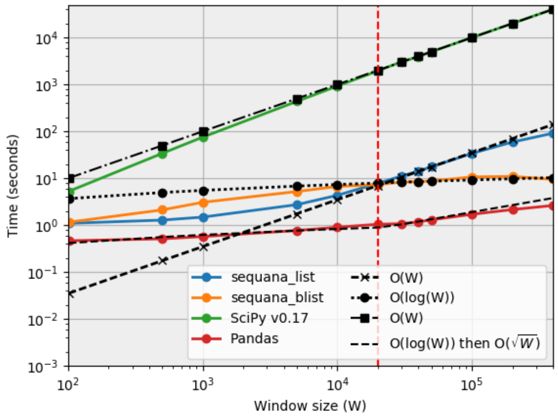

Figure 10. Computational cost of running median algorithms as a function of the window size parameter W (for N = 1e6). Four variants are considered: SciPy [32] implementation (function medfilt v0.17), Pandas [25] and 2 Python variants available in `Sequana` based on a `list` or `blist` data containers (see text for details). The `SciPy` variant has a $\mathcal{O}(W)$ complexity irrespective of the W value. For low W values (W < 20 000), the two Python variants have $\mathcal{O}(log(W))$ complexity. For larger W values, the blist keeps its $\mathcal{O}(log(W))$ complexity while the list container follows a $\mathcal{O}(W)$ complexity. Pandas complexity is less clear with a O(W) for W < 20 000 and $\mathcal{O}(log(W))$ otherwise. The fastest implementation is clearly the Pandas one even for large W values.

So far, we have neglected the cost of the insertion and deletion steps, which is not negligible. For instance, in Python language, one of the most common data structure is the *list*. It is a dynamically-sized array (i.e., insertion and deletion of an item from the beginning or middle of the list requires to move most of the list in memory) and the look-up, insertion and deletion have a $\mathcal{O}(n)$ complexity. So the running median is actually dominated by the slow $O(n)$ insertion and deletion steps. A better data structure is available thanks to the *blist* package; it is based on a so-called B-tree, which is a self-balancing tree data structure that keeps data sorted. The blist allows searches, sequential access, insertions, and deletions in $\mathcal{O}(\log n)$ (see https://pypi.python.org/pypi/blist/ for details).

Based on materials from http://code.activestate.com/recipes/576930/, we have implemented these two variants of running median functions in Python available in **Sequana** [27] library. We also considered established numerical analysis tools from the `SciPy` [32] and Pandas [25] libraries. We finally compare the four implementations in terms of computation time and complexity as shown in Figure 10. It appears that the Pandas implementation is the fastest. For $W > 20,000$ up to 200,000, our implementation is 2-3 order of magnitude faster than the `SciPy` version but 4-5 times slower than Pandas. We should emphasize the fact that the `SciPy` function has additional features since it is available for N-dimensional data sets whereas we restrict ourselves to 1-D data sets. In `Sequana`, the two variants only differ in the data structure being used to hold the data (list versus `blist`). The Figure 10 shows the difference between the list and blist data structures that is marginal for low W values while for large values asymptotic behaviours are reached showing the interest of the blist over the list choice. We also see that our implementation with blist has a lower complexity than the Pandas implementation. However, for the range considered Pandas is always the fastest choice.
