# Supplementary material for: Sequana coverage: detection and characterization of genomic variations using running median and mixture models
Source: Gigascience. 2018 Sep 6;7(12):giy110. doi: 10.1093/gigascience/giy110 (PMC6275460; doi:10.1093/gigascience/giy110)
Supplement: GIGA-D-17-00238_Revision_1.pdf [file giy110_giga-d-17-00238_revision_1.pdf]

# GigaScience


## Sequana Coverage: Detection and Characterization of Genomic Variations using Running Median and Mixture Models

--Manuscript Draft--

| | |
|---|---|
| **Manuscript Number:** | GIGA-D-17-00238R1 |
| **Full Title:** | Sequana Coverage: Detection and Characterization of Genomic Variations using Running Median and Mixture Models |
| **Article Type:** | Technical Note |
| **Funding Information:** | Agence Nationale de la Recherche (ANR10-INBS-09-08) — Mr Dimitri Desvillechabrol |
| **Abstract:** | Background: In addition to mapping quality information, Genome coverage contains valuable biological information like the presence of repetitive regions, deleted genes or copy number variations. It is essential to take into consideration atypical regions, trends (e.g., origin of replication) or known and unknown biases that influence coverage. It is also important that reported events have robust statistics (e.g. z-score) associated with their detections as well as precise location<br><br>Results: We provide a standalone application -- sequana_coverage -- that reports genomic regions of interest (ROIs) which are significantly over- or under-represented in HTS sequencing data. Significance is associated with the events as well as characteristics such as length of the regions. The algorithm first detrends the data using an efficient running median algorithm. It then estimates the distribution of the normalized genome coverage with a Gaussian mixture model. Finally, a z-score statistic is assigned to each base position and used to separate the central distribution from the ROIs (i.e., under- and over-covered regions). A double thresholds mechanism is used to cluster the genomic ROIs. HTML reports provide a summary with interactive visual representations of the genomic ROIs with standard plots and metrics. Genomic variations such as single nucleotide variants (SNVs) or copy number variations (CNVs) can be effectively identified at the same time. |
| **Corresponding Author:** | Thomas Cokelaer, Ph. D.<br>Institut Pasteur<br>Paris, FRANCE |
| **Corresponding Author Secondary Information:** | |
| **Corresponding Author's Institution:** | Institut Pasteur |
| **Corresponding Author's Secondary Institution:** | |
| **First Author:** | Dimitri Desvillechabrol |
| **First Author Secondary Information:** | |
| **Order of Authors:** | Dimitri Desvillechabrol |
| | Christiane Bouchier, Ph. D. |
| | Sean Kennedy, Ph. D. |
| | Thomas Cokelaer, Ph. D. |
| **Order of Authors Secondary Information:** | |
| **Response to Reviewers:** | Dear editor,<br><br>Please find here below the changes made to the manuscript and supplementary data as well as the answers to the reviewers' comments. In the submission, we also provide a file called main_annotated.pdf with changes highlighted. |

Powered by Editorial Manager® and ProduXion Manager® from Aries Systems Corporation

The main differences concern the section “Application” that was rewritten to include a discussion on CNV detection. We also added a sub section about the “impact of the running median window parameter”, figure 2 was replaced with 2 different panels and a different example. Other changes are shown in the main_annotated.pdf file. Note also that we changed the title from

Sequana Coverage: Automatic Detection and Characterization of Low and High Genome Coverage Regions.

To

Sequana Coverage: Detection and Characterization of Genomic Variations using Running Median and Mixture Models.

Best regards

Thomas Cokelaer on behalf of the authors

REVIEWER 1
==============================================================

QUESTION

The authors presented `sequana_coverage` as a tool that can be used to automatically detect low/high-coverage regions of interest (ROI) on genome sequencing data. The main reason for detecting these regions (parsing from the submission) is to detect > potentially interesting biological features and also to assess the quality of mapping to a reference genome. The submission is quite clear in its scope and the authors should be commended for making the source code open and installation of the too available via a publicly-accessible repository. The code, though not explicitly shown here, is readable and not difficult to understand. It is also continuously tested, which puts it above a large number of published scientific tools in terms of code quality. However, there are some concerns and questions that I came across while reviewing the manuscript, which I hope can be clarified and/or explained by the authors in order to improve the quality of the submission.

Major concerns:

The authors highlighted the dangers of using static coverage boundary across the genome and uses it as the rationale of developing the methods described in the submission (last 4 paragraphs of the background section). However, there are two important use cases here that should be better distinguished: the use of coverage for assessing quality metrics or for detecting biologically interesting features. Since the authors mentioned Type 1 and 2 errors, one has the impression that it is the latter use of coverage that is being discussed. Unfortunately, this does not make for a convincing rationale for developing the tools since published methods that do use coverage for detecting features (for example, copy number variation detection or simple structural variation detection) do not employ fixed coverage boundary to do so but instead rely on complex statistical methods tailor-made for the problem at hand [for example, see Brynildsrud et al. 2015 [https://www.ncbi.nlm.nih.gov/pubmed/25644268]). Could the authors comment on this?

ANSWER:

We originally designed sequana_coverage to quickly access standard metrics such as depth of coverage (DOC) or breadth of coverage (BOC) and also visualise the coverage for viral and bacterial genomes. We also wanted a quick and efficient means to visualise and detect atypical significant events that occur in the depth of coverage signal. The goal here was to detect potentially interesting biological features including deleted genes but also much shorter features.

“one has the impression that it is the latter use of coverage that is being

discussed." : Indeed the manuscript, we focused on the second aspect, which should be clear from the background section that has been edited accordingly.

"this does not make for a convincing rationale for developing the tools since published methods that do use coverage for detecting features ... do not employ fixed coverage boundary…. " We use fixed threshold as an example to emphasize the DOC biases that exist in replicating bacteria genomes. We then provide a solution (running median). We then complement our approach with robust statistical method.

QUESTION

One application I was hoping to see explained (or at least compared to) is the detection of copy number variation. There, the detection of features are similarly based on sequencing coverage and no pre-set coverage values need to be set for detection. Zare, et al. (2017) [https://bmcbioinformatics.biomedcentral.com/articles/10.1186/s12859-017-1705-x] calls this the read-count based method for detecting CNVs. Seeing that CNVs also represent interesting biological features, an omission of this discussion makes the submission feel incomplete.

ANWSER:

Our tool was not designed to specifically detect CNVs, however the reviewer's comments make it clear that this would improve our tool and broaden its interest. To that end, we added a section addressing CNV detection. sequana_coverage is sown to be competitive with existing tools such as in Brynildsrud et al., 2015 or Abyzov et al 2011 (CNVnator). This was not mentioned in the original manuscript and we have updated the code and the manuscript to emphasize this capability.

In particular, we have compared sequana_coverage with results found in the supplementary results from Brynildsrud et al.
Our comparison shows that we can retrieve the properties of the CNVs (position, length, copy number); we can detect the same CNVs (>1000 bp) and could also detect short features (1 to 1000 bp), which were not reported in Brynildsrud et al.

As an example, we show in the following image that the CNV at position 2,874,000 is detected with the exact position and length (+- 1 base; horizontal colored segments) whereas CNOGpro split the event into 5 parts and reports starting and ending position with large offset. See for example figure here:

https://tinyurl.com/y7jy5agt

The intention of the manuscript was not, and is not, to claim that sequana_coverage is better or worst than CNOGpro or other such tools. This use of sequana_coverage illustrates that this potentially interesting biological feature, CNVs, can be detected and brought to the attention of the biologist. As such, sequana_coverage is competitive with respect to CNOGpro.

We have also looked at another tool called CNVnator. On the same species and test example as above, we found that CNVnator and sequana_coverage identify the same CNVs. In terms of calculation time, sequana_coverage was as fast (faster for viral and bacterial) as CNVnator.

We also compared sequana_coverage and cnvnator on a human genome (NA12878 from the 1000 genomes project) as described in the notebook available here:

https://github.com/sequana/resources/blob/master/coverage/comparison_cnvnator_human/human_case.ipynb

A full comparison of the two tools is not within the scope of this manuscript

given the complexity of the human genome. Yet, recognizing the potential interest of the community,

we updated sequana_coverage and demonstrate we can analyse such data with a reasonable memory footprint (3Go; CNVnator requires 6.5Go). We subsequently analyzed the 24 human chromosomes with both tools. CNVnator took 5.5 hours on a single CPU and could analyse the same data in a an hour on a dual-core. We found that sequana_coverage required about 20 hours to complete. We decided to provide a simple Snakemake (https://snakemake.readthedocs.io/en/stable/) pipeline to analyse the chromosome in parallel on a cluster (24 chromosome files using 24 CPUs). With this configuration, the analysis took 1 hour.

Additional features are complement existing CNV detection tools because:

- It can also detect short features thanks to the running median.
- The underlying algorithm is simple (running median + normalisation, + mixture model) but is nevertheless based on solid statistical considerations
- HTML reports are provided, which is very convenient in the context of viral and bacterial genomes
- Multiqc report are now available to compare different contig statistics
- Snakemake pipeline is provided to analyse several chromosomes in parallel
- The entire code is in Python and researchers can built on top of it as demonstrated n the numerous notebooks that have been provided in this open resource page on github: https://github.com/sequana/resources/tree/master/coverage

In summary, we have improved the manuscript (and sequana_coverage) significantly (application section) by adding a dedicated section on CNVs detection.

QUESTION

It would make for a stronger submission if the features detected using `sequana_coverage` and how they would look like using a naive setting of coverage depths is discussed. The bacteria test case explained further in Figure 7, while interesting, does not > really convince the readers on the need of this tool as the changes in coverages seem to be visible even without using the tool (i.e. just by plotting the raw coverages). In figures 8-9 it seems to be the case as well that detection of the ROI can be done by > using only the mapping quality track.

ANWSER:

The bacteria test case example of the figure 7 (previous version) has been removed. Instead, the new figure 2 (a virus example) is more convincing:

https://tinyurl.com/yc6a75js
https://tinyurl.com/y884rjwl

In the region shown, we have a deleted event (15,000 bases) followed by a depleted event (5,000 bases) with a copy number of 0.5 separated by 5,000 bases. This better emphasizes the inability of a moving average to detect the depleted event. The red/green dots indicate detected events: there are many false positives in the left panel, while the running median (right) has a much better performance.

Figure 8 and 9 have been removed and replaced with examples related to CNV detections.

We are convinced of the utility of using a running median. As space in the text is limited, we provide a notebook that explains the negative impact of the constant threshold in the presence of replication or non-constant depth of coverage along the genome (it also contains examples that motivates the use of a running median instead of moving average):

Powered by Editorial Manager® and ProduXion Manager® from Aries Systems Corporation

https://github.com/sequana/resources/blob/master/coverage/running_median_motivation/running_median.ipynb

QUESTION

What are the characteristics of the features that can be detected using this tool. For example, are they between a specific size range (in relation to, for example, the window size and/or the read length). This is not completely clear from the submission, and while some methods of correlating the detected features to known biological features are presented, one wonders if such detection can be made with simpler methods.

ANWSER

Sequana_coverage can detect any events from 1 base to W/2 bases. There was some confusion as to how W is set, and it’s optimal value. We have clarified the text to explain the impact of the window length parameter, W, adding a section called ‘Impact of the running median parameter’. We have also provided a notebook with examples and figures:

https://github.com/sequana/resources/tree/master/coverage/window_impact

In summary, in order to detect (and avoid the impact) of a deleted or duplicated event of length N, one should use a window parameter W > 2N . This is independent of the genome size. However, if you have a small genome (e.g. virus), then the window size must be less than this genome size, of course. In such situation, we would recommend a fifth of the genome size.

The impact of the W parameter on z-score is marginal. One should not set W to large values (e.g., 500,000) otherwise the trend will not be correctly estimated. We also recommend a W>20,000 ; below this there is a slight increase of false positives (see image below and notebooks https://github.com/sequana/resources/tree/master/coverage/window_impact).

https://tinyurl.com/y888n8jd

So, we can detect events from 1 base to W / 2 bases. The drawback of using larger windows are (i) slower computation, although the implementation in place is very efficient so this is marginal, (ii) too much smoothing will decrease the detection power if the signal is very noisy.

We have changed the text to mention those different aspects.

One final point concerns the reviewer’s allusion to ‘simpler methods.’ We feel that sequana_converage takes into account the biases and variability inherent in both biological systems as well as HTS data. Indeed part of our motivation is to accurately account for significant variations in DOC in sequencing data that may or may not be biologically relevant. These are not simple systems and thus require analysis with tools adapted to the task.

Minor concerns:

QUESTION

Although the tool here is presented as a standalone tool, installing it requires installation of other tools and pipelines in the authors' sequana` toolkit. It would be more suitable if the tool is also packaged as its own, truly standalone package.

ANSWER

The easiest and most robust way to use sequana_coverage is to use the singularity solution. Singularity tool (http://singularity.lbl.gov/) can be installed in minutes.

We provide sequana (version 0.7.0) as an image on Singularity hub https://www.singularity-hub.org/collections/114) .

Although the image is 1.7Gb it contains all the tools required. It does not require any compilation and is reproducible. We are not aware of a simpler solution that would provide an executable that would work locally and on a cluster.

We also provide sequana version 0.7.0 on bioconda (https://www.biorxiv.org/content/early/2017/10/21/207092) , in review in Nature Method. and on Pypi website for python developers.

Everything is documented on sequana.readthedocs.io in particular, please see http://sequana.readthedocs.io/en/master/installation.html#singularity

The reason we do not provide sequana_coverage as a standalone is because of manpower: we decided to keep the coverage tool within the sequana library to ease the development and debugging of the tool and allows us to be more responsive. We have every intention of maintaining this tool in the future, and this structure is most conducive with this goal.

QUESTION

The version of the `sequana_coverage` tool used for producing the plots is unfortunately not mentioned clearly. Could the authors clarify this?

ANWSER

The version used in the manuscript and notebooks is 0.7.0, which is now also written in the manuscript.

QUESTION

The EM algorithm to which the author refers in the statistical section, should be expanded as Expectation Maximization instead of Expectation Minimization. This is how it was presented in the referred Dempster et al. (1977) paper.

ANSWER

Thank you for catching this oversight. It has been corrected.

QUESTION

The rationale on choosing the running median window size is not completely clear. Are there any relations between this value and, for example, the read length or the reference genome size?

ANSWER

As mentioned above, in order to detect a deleted or duplicated event of length N, one should use a window parameter W such that W > 2N , which is independent of the the genome size, coverage or read length.

Could the author comment on the suitability of using `sequana_coverage` for other species such as humans, mice, and/or some plant species?

ANSWER

We have used sequana_coverage for viruses, bacteria and fungi. However, in the notebook

https://github.com/sequana/resources/tree/master/coverage/comparison_cnvnator_human

we also demonstrate that it can be used for humans. We have now updated the code that allows the analysis of a human genome in an hour (+1 hour to convert BAM to BED files), which is competitive with dedicated tools such as CNVnator (5-6 hours).

Powered by Editorial Manager® and ProduXion Manager® from Aries Systems Corporation

QUESTION

Page 2, line 27-30. The authors wrote: "For instance, to detect human genome mutations, single-nucleotide polymorphisms (SNPs), and rearrangements, a 50 X depth is recommended [1] to be able to distinguish between sequencing errors and true SNPs." Could the authors clarify which table and/or statements present in the cited article that contains this recommendation?

ANSWER

This refers to reference 15 (Ajay et al., 2011) of the reference 1. We changed the text to add the missing reference. We now refer to a range 30-50X instead of 50X since this really depends on the application (SNPs, SNV) and technologies.

REVIEWER2
=================================================================

In this manuscript, Desvillechabrol et al. introduce seqana_coverage, a method and tool for estimating statistically significant deviations in genome coverage data and providing an automated method to detect genomic regions of interest (ROI). The overall algorithm method seems sound, and consists of de-trending the data using a running-median filter, followed by learning foreground (center) and background (outlier) distributions, as well as their maximum likelihood mixture proportions in the sample. Finally, given the Gaussianity assumptions of the model, they define a z-score that can be computed for each base in the genome, which can aid in discovering loci that exhibit statistically surprising high or low coverage. I generally find the manuscript convincing, and the tool looks as though it will be useful. However, I have a couple of high-level questions about the proposed solution and the manuscript.

Questions / comments ----

There is not much discussion in the current manuscript on the effect of W, the window length used in the running-median de-trending algorithm. Do the authors have a recommended procedure for setting W? It would seem that the choice of W will set a "scale" for outliers, and while the approach would generally be robust to small changes in W, larger changes could highlight different regions of interest (this, of course, can be viewed as a feature rather than a problem). However, for someone wishing to use the tool, a recommendation on how to set W, or what values make sense for discovering different types of regions of interest, would be useful.

ANSWER

We have clarified the text to explain the impact of the window length parameter, W, adding a section called ‘Impact of the running median parameter’. We have also provided a notebook with examples and figures:

https://github.com/sequana/resources/tree/master/coverage/window_impact

In summary, in order to detect (and avoid the impact) of a deleted or duplicated event of length N, one should use a window parameter W > 2N . This is independent of the genome size. However, if you have a small genome (e.g. virus), then the window size must be less than this genome size of course. In such situation, we would recommend a fifth of the genome size.

The impact of the W parameter on z-score is marginal. One should not set W to large values (e.g., 500,000) otherwise we may not estimate the trend correctly. We also recommend a W>20,000 ; below, there is a slight increase of false positives (see image below and notebooks https://github.com/sequana/resources/tree/master/coverage/window_impact).

https://tinyurl.com/y888n8jd

Powered by Editorial Manager® and ProduXion Manager® from Aries Systems Corporation

So, we can detect events from 1 base to W / 2 bases. The drawbacks of using larger windows are (i) slower computation, although the implementation in place is very efficient so this is marginal, (ii) too much smoothing which decreases detection power if the signal is very noisy.

We have changed the text to clarify those different aspects.

QUESTION

Related to the above, one potential effect of the de-trending that is performed in seqana_coverage is that outliers appear to generally be high-frequency effects. Would the proposed approach be applicable if one wished to discover low-frequency effects (i.e. very large ROIs)? For example, one might expect a dip in coverage in difficult-to-sequence regions or in regions with highly-repetitive arrays of elements. Would it be possible to detect such large-scale regions using the proposed approach, or do they not classify as outliers under the assumed definition?

ANWSER

Sequana_coverage can detect any events from 1 base to W/2 bases. We generally set W to 20,000 but it can be increased safely to 50,000, or even larger values. For instance, when the goal is really on the detection of large events, such as those to be found in human genome, we used values of 250,000 or even 500,000. We then compared to CNVnator, a CNV dedicated tool, as shown in:

https://github.com/sequana/resources/tree/master/coverage/comparison_cnvnator_human

Certainly a much more detailed investigation would be needed to fully characterize and compare the behaviour of sequana_coverage algorithm on this kind of data. This type of analysis of human CNV detection is out of scope with the current manuscript; we indeed focused on bacterial genome at first. Nevertheless, it looks promising both in terms of computational time and overlap with CNVnator. Larger values for W would not be recommended since the running median would not follow the trend closely, especially in the presence of long deleted regions, which are common in human genomes

QUESTION

I'm not certain I understand, or completely agree with, the argument that the outlier distribution can safely be assumed as Gaussian. Specifically, for the CLT to apply, we must be taking the (normalized) sum of a large number of *independent* random variables. However, it's not immediately clear to me why the outlier samples are independent --- e.g., changes due to repeated elements may be be correlated. Also, given the definition of the outlier samples (those with considerably higher or lower coverage than their local, de-trended window), it's not completely clear why this distribution may not be e.g., bimodal. Perhaps the assumption of normality is simply a computational convenience, as it allows closed-form update rules for the mixture model being applied. But perhaps this assumption could be addressed empirically (e.g., by looking at a goodness-of-fit test of the outlier samples to a normal distribution with the inferred mean and standard deviation).

ANSWER

Our assumption of normality of the distributions that compose the normalised coverage is empirical. Moreover, as the reviewer stated in his comment/question, this is also a convenient choice to allow us to fit a mixture of Gaussian models. However, low depth of coverage does not exhibit a Gaussian distribution, and we do not recommend to use sequana_coverage for coverage below 10X.

As for the possible bimodal distributions of outliers on each side of the central distribution; this is indeed a possibility. However we are not concerned by the

Powered by Editorial Manager® and ProduXion Manager® from Aries Systems Corporation

outliers at this stage, as the most important aspect is the statistical properties of the central distribution. This is why we use k=2 in the mixture models and why we assume the central distribution to be predominant.

Our hypotheses is that the central distribution is Gaussian, which looks reasonable in the cases considered. For instance, in the case of a simulated data set, the mapped data is normalised and exhibits a central distribution that is gaussian (see link to figure below).

https://tinyurl.com/y9ues5t4

QUESTION

It would be nice if the authors could provide a couple of examples demonstrating the underlying biological cause of some of the outliers identified by their algorithm. Specifically, the algorithm is demonstrated on 3 real datasets, and outliers are predicted. It would be a nice addition to the manuscript to select a few extreme outliers and discuss what gave rise to them.

ANSWER

We have updated the manuscript, and now show examples from data from CNOGpro (6 strains of Staphylococcus) to emphasize the ability of sequana_coverage to identify CNVs with perfect location and size, in itself an 'extreme' case  when deal with human data. However, because the manuscript now has a dedicated section on CNVs, we are lacking place for extra examples demonstrating the underlying biological cause of some of the outliers.

Figure 9 also demonstrates a potentially extreme event, where a duplication of 10X has no impact on the detection of the  two flanking events of lesser intensity. Even were a region to have 100X or 1000X coverage, it would have no impact on surrounding events as long as the size is less that W / 2. A strength of the running median is that extreme events are handled exceptionally well.

Minor concerns -----
The second sub-section of the methods section is titled "Building a statistics", which sounds awkward and should probably be reworded.

Changed to "Parameter estimation of the central distribution and adaptive thresholds in the original space ", which is less concise but is more explicit.

In the same section, when justifying the Gaussianity assumption for the outlier distribution, the authors claim "we can consider that the outliers population is a mix of samples and that we are in the limit of the central theorem". The wording here is a bit strange --- I would, instead, suggest something like "if we consider the outlier population as arising from a mix of different independent samples, then the central limit theorem applies, implying that $\tilde{C}_b^1$ can be treated as Gaussian."

The wording is now that suggested by the reviewer. Thank you.

REVIEWER3
======================================

This manuscript presents a novel method for detecting regions of anomalous mapped read depth. As I understand it from the manuscript, the expected uses of this appear to be to detect problems with either a genome or metagenome assembly or sequencing sample, and to assess the GC bias in a sample. The authors do really address the other application that might seem obvious: the calling of CNVs. The following review will be broken down into two pieces: the manuscript and the software. I will conclude with a summary of my recommendations.

I have two major concerns:

Any new piece of software aiming to solve a problem where other pieces of software attack the same problem should be compared to these other tools. While the authors do mention a couple of other approaches, mostly from the field of metagenomics, there exist a range of tools that use read depth to investigate CNVs. See for example Yao et al Molecular Cytogenetics 2017 10:30 doi:10.118/s13039-017-0333-5, Zhao et al BMC Bioinformatics 2013 14(Suppl 11):S1 doi:10.1186/1471-2105-14-511-S1. The authors should set their method in the context of these methods and how how it differs in its aims/approach/performance. If applicable a comparison between their method and others on the same dataset should be presented. At a minimum I would like to see a comparison to CNVnator (Abyzov et al, Genome Research, 2011 21:974-948, doi: 10.1101/gr.114876.110) or an explanation of why such a comparison is not appropriate.

ANWSER

Indeed, the original version of our manuscript did not mention CNVs or make reference to existing CNV detection tools. Thanks to the reviewers comments and feedbacks, we significantly changed the application section, adding specific reference to CNV detection. We have addressed this issue in significant depth by comparing sequana_coverage with CNOGpro, which is primarily aimed at bacterial genome. For comparison we re-used their data sets, as the authors also selected Staphylococcus as a test case (6 strains). We also compared CNVnator, which is dedicated to whole eukaryotic genomes (human).

We have several notebooks where we compare CNOGpro results with the sequana_coverage (also CNVnator). :

https://github.com/sequana/resources/tree/master/coverage/08-comp_CNOGpro_cnvnator_sequana_bacteria

https://github.com/sequana/resources/tree/master/coverage/09-comparison_cnvnator_bacteria

https://github.com/sequana/resources/tree/master/coverage/10-comparison_cnvnator_virus

https://github.com/sequana/resources/tree/master/coverage/11-comparison_cnvnator_human

In the following figure, there is an obvious CNV event with copy number of 3.5. Coverage data for one strain (black curve) is shown with the corresponding sequana_coverage event detection (horizontal black line). The other 5 horizontal coloredline show the reported event in the 5 other strains. This demonstrates a few important points: (1) all strains exhibit the same event (2) sequana_coverage called all 6 cases (3) the precision of the position and length of the events is at the same position (precise). The reported mean coverage of the events allows us to determine the average copy number. In comparison, CNOGpro identifies the events, but splits them into several events (colored green and red areas) because it uses a different paradigm (split of the genome into intergenic segments; here the green and red areas). CNVnator, by comparison, is also very precise and consistent in identifying this event.

https://tinyurl.com/y7jy5agt

This is just one example (more are provided in the notebook). The main conclusions are that sequana_coverage identifies the same events as CNOGpro and CNVnator and is often more precise. We tested sequana_coverage for human genomes and found that it is competitive with CNVnator in terms of computational time even though a full comparison on human is evidently more difficult than for bacteria, which could be checked by hand.

As reported in Zhao et al., existing CNV tools (48 tested !) do not agree and should be used together rather than independently. Thus, sequana_coverage can be

considered as a good complement to the existing tools. Moreover, one additional strength is that it can detect short events that are missed by CNOGpro and CNVnator as explained in the notebook:

https://github.com/sequana/resources/tree/master/coverage/comparison_cnvnator_bacteria

Here is an example of such an event where sequana_coverage detects 4 events (orange and blue areas) while CNVnator detects only the first and last, completely missing the short strong event and the first long but weak depleted region:

https://tinyurl.com/yaycvxzf

Of course, the different results depend on parameters in the different tools,. Nevertheless, this kind of result is observed with CNOGpro as well, and the underlying mean-shift tool is the cause of the missed events.

We have now added a 2-page section in the application section with further details in response to the reviewer's comments and suggestions.

QUESTION

The algorithm has a single tuning parameter, W. The authors state that the value of this setting has little effect on the parameters learnt by their model. The authors should expand on this further: presumably the choice of W does have an effect on the z score obtained For example I believe any region of elevated or depressed coverage larger than W would clearly be missed. The authors should include a discussion of how changing W changes the results and how to choose a good value for W.

ANSWER

We have clarified the text to explain the impact of the window length parameter, W, adding a section called 'Impact of the running median parameter'. We have also provided a notebook with examples and figures:

https://github.com/sequana/resources/tree/master/coverage/window_impact

In summary, in order to detect (and avoid the impact) of a deleted or duplicated event of length N, one should use a window parameter W > 2N . This is independent of the genome size. However, if you have a small genome (e.g. virus), then the window size must be less than this genome size of course. In such situation, we would recommend a fifth of the genome size.

The impact of the W parameter on z-score is marginal. One should not set W to large values (e.g., 500,000) otherwise we may not estimate the trend correctly. We also recommend a W>20,000 ; below, there is a slight increase of false positives (see image below and notebooks https://github.com/sequana/resources/tree/master/coverage/window_impact).

https://tinyurl.com/y888n8jd

So, we can detect events from 1 base to W / 2 bases. The drawback of using larger windows are (i) slower computation, although the implementation in place is very efficient so this is marginal, (ii) too much smoothing will decrease the detection power if the signal is very noisy.

We have changed the text to mention those different aspects.

QUESTION

There are also a number of smaller concerns or comments. Under "building a statistic" the authors state that the per-base coverage should theoretically follow a poisson distribution. Is it not actually the case that it is the number of reads that start at any given base that should follow a poisson distribution, and the non-independent nature of

depths and nearby bases may account for some of the over-dispersion?

ANSWER

We have added a reference (Lindner et al.) concerning the Poisson distribution . We have not found any reference regarding a theoretical reason for the over-dispersion.

QUESTION

It is not clear to me why the gaussian approximation of the negative binomial becomes valid at 10X coverage, rather than 5 or 20.

This is an empirical choice. One can analyse data with coverage of 5X, as we did for the human genome. However, we can see (visually) that the distribution is skewed to large depth, meaning the gaussian approximation is not valid. One can still change the threshold to reduce the number of reported events. Practically, we propose 10X to be a level of coverage where the statistical assumptions upon which event detect is based become valid. (Ajay et al., 2011), cited in the manuscript, suggests 50X coverage for the detection of certain events and the biologist must assume some responsibility for the amount of sequencing necessary to answer a specific question..

QUESTION

The authors state that bases of 0 coverage are not included in their fitting, but would these values not be important in finding the mixing parameters?

ANSWER

No, we are interested in fitting a distribution on the central part of the distribution. The zeros are zeros and belong to the population of outliers. Yet, if we include them, they may have a negative impact of the EM algorithm. So, we decided to ignore them.

QUESTION

The authors state that one of the benefits of their approach is that a statistically meaningful value is attached to each base and that they can control the false positive rate this way. However, they then go on to use a two step threshold to identify regions. It seems to me that that the above benefit does not transfer over to the regions and it is difficult to assess the statistical properties of the regions. Perhaps the authors should comment on this in their discussion.

ANSWER

With the algorithm in place, a z-score on a per-base is computed. The 2-step threshold allows us to cluster events. In order to report a dedicated z-score (probability to see N bases crossing a threshold), we should assume that two consecutive bases are independent, which is not the case. So, we report the mean-zscore and max z-score of the events. A robust statistical property for such events would require more investigations.

QUESTION

The authors comment on how the method scales to viral, bacterial and yeast datasets. How would the performance scale if applied to mammalian or plant genomes?

ANSWER

Since the first version of the manuscript, we have improved the code to handle eukaryote data sets. We use the same data set from the 1000 genomes project as in CNVnator (3.5Gb). We found that performance scales well and that we can analyse the data in a couple of hours, compared with CNVnator that took 5-6 hours. To be fair, CNVnator takes as input a BAM file whereas sequana_coverage still needs the BED file as input. So, if we take into account the BAM to BED conversion, this needs an extra hour but is done once for all. Finally, to reach a

2-hours computational time, we also use the same binning of 100 used by CNVnator.

Software

Of the three available installation routes I was able to install the software without an error message at install time using only one route - via the sequana conda package. I was a little perturbed to be made to download and install several Gb of dependencies just to use what one would image would be a fairly dependency-lite piece of software. Indeed I several times ran out of disk space on both the Ubuntu virtual machine and institutional HPC accounts I attempted the installation on. This was due to having to download an install the whole of the sequana collection, which has many dependencies way beyond the scope of the tool under review here. I obtained the viral dataset used in the manuscript from the synergy and attempted to run the tool. The tool seemed to get though most of its processes, unfortunately I was met with an error I was unable to solve in what I assume was the reporting phase. Unfortunately I don't feel that I can provide a full review of the software until I am able to run it fully with out error. My personal suggestion for a tool as self contained as this it would be better it to could be installed on a stand alone basis with a minimal number of dependencies. This would minimise the chance for things to go wrong and also minimise the footprint of the tool on the users system. To be useful to many bioinformaticians, who do most of their work on institutional clusters, it needs to be possible to successfully install without root permissions. Conda should allow this to be possible.

ANWSER

The easiest and most robust way to use sequana_coverage is to use the singularity solution. Singularity tool (http://singularity.lbl.gov/) can be installed in a couple of minutes. We provide sequana (version 0.7.0) as an image on Singularity hub (https://www.singularity-hub.org/collections/114) .

Although the image is 1.7Gb it contains all the tools required. It does not require any compilation and is reproducible. We are not aware of a simpler solution that would provide an executable that would work locally and on a cluster.

We also provide sequana version 0.7.0 on bioconda (https://www.biorxiv.org/content/early/2017/10/21/207092) , in review in Nature Method.
and on Pypi website for python developers. For conda, these commands installed sequana_coverage in about 5-10 minutes on a Fedora box:

```
conda create --name sequana_0_7 python=3.5
source activate sequana_0_7
conda install sequana==0.7.0
```

Everything is documented on sequana.readthedocs.io in particular, please see http://sequana.readthedocs.io/en/master/installation.html#singularity

Recommendations

QUESTION

I believe that the work here is a valuable contribution to the field and could be suitable for publication if the authors addressed the comments outlined. Of particular importance:

Comparisons of the method to published methods using read-depth to call CNVs (for example CNVnator).

ANWSER

We have compared sequana_coverage tool with CNVnator and CNOGpro on bacterial genome including 6 isolates of staphylococcus.
Moreover, we compared CNVnator and sequana_coverage on (i) a viral genome,

| | |
|---|---|
| | (ii)<br>a human genome. We included a 2 pages section in the new version of the manuscript<br><br>QUESTION<br>A discussion of the effect of changing the W parameter and how to choose a good value<br><br>ANWSER<br><br>We have added a subsection "Impact of the running median parameter" to discuss this aspect.<br><br>QUESTION<br><br>I need to be able to install the software flawlessly on, for example, a freshly minted Ubuntu virtual machine or similar (X and conda or pip installed) and run without error. Ideally there should be a non-root requiring way to install.<br><br>ANWSER<br><br>We recommend the singularity solution as described here:<br>http://sequana.readthedocs.io/en/master/installation.html#singularity<br><br>First install singularity 2.4.2:<br>Second, download the sequana singularity 0.7.0 image:<br><br>singularity pull --name sequana_0_7_0.img shub://sequana/sequana:0_7_0<br><br>Third use the standalone:<br><br>Singularity exec sequana_0_7_0.img sequana_coverage --input test.bed |
| **Additional Information:** | |
| **Question** | **Response** |
| Are you submitting this manuscript to a special series or article collection? | No |
| **Experimental design and statistics**<br><br>Full details of the experimental design and statistical methods used should be given in the Methods section, as detailed in our Minimum Standards Reporting Checklist. Information essential to interpreting the data presented should be made available in the figure legends.<br><br>Have you included all the information requested in your manuscript? | Yes |
| **Resources**<br><br>A description of all resources used, including antibodies, cell lines, animals and software tools, with enough information to allow them to be uniquely identified, should be included in the | Yes |

| | |
|---|---|
| Methods section. Authors are strongly encouraged to cite Research Resource Identifiers (RRIDs) for antibodies, model organisms and tools, where possible.<br><br>Have you included the information requested as detailed in our Minimum Standards Reporting Checklist? | |
| **Availability of data and materials**<br><br>All datasets and code on which the conclusions of the paper rely must be either included in your submission or deposited in publicly available repositories (where available and ethically appropriate), referencing such data using a unique identifier in the references and in the “Availability of Data and Materials” section of your manuscript.<br><br>Have you have met the above requirement as detailed in our Minimum Standards Reporting Checklist? | Yes |

Manuscript
Click here to download Manuscript mainv2.pdf

GigaScience, 2017, 1–11

doi: xx.xxxx/xxxx
Manuscript in Preparation
Technical Note

TECHNICAL NOTE

# Sequana Coverage: Detection and Characterization of Genomic Variations using Running Median and Mixture Models.

Dimitri Desvillechabrol[1†], Christiane Bouchier[1], Sean Kennedy[1] and Thomas Cokelaer[1,2,†,*]

[1]Institut Pasteur – Pole Biomics – Paris, France and [2]Institut Pasteur – Bioinformatics and Biostatistics Hub – C3BI, USR 3756 IP CNRS – Paris, France

[†]equal contributions
[*]corresponding author
Emails: ddesvillechabrol@gmail.com, christiane.bouchier@pasteur.fr, sean.kennedy@pasteur.fr, thomas.cokelaer@pasteur.fr

## Abstract

Background: In addition to mapping quality information, the Genome coverage contains valuable biological information like the presence of repetitive regions, deleted genes or copy number variations. It is essential to take into consideration atypical regions, trends (e.g., origin of replication) or known and unknown biases that influence coverage. It is also important that reported events have robust statistics (e.g. z-score) associated with their detections as well as precise location.
Results: We provide a standalone application – sequana_coverage – that reports genomic regions of interest (ROIs) which are significantly over- or under-represented in HTS sequencing data. Significance is associated with the events as well as characteristics such as length of the regions. The algorithm first detrends the data using an efficient running median algorithm. It then estimates the distribution of the normalized genome coverage with a Gaussian mixture model. Finally, a z-score statistic is assigned to each base position and used to separate the central distribution from the ROIs (i.e., under- and over-covered regions). A double thresholds mechanism is used to cluster the genomic ROIs. HTML reports provide a summary with interactive visual representations of the genomic ROIs with standard plots and metrics. Genomic variations such as single nucleotide variants (SNVs) or copy number variations (CNVs) can be effectively identified at the same time.

Key words: genome coverage, sequencing depth, running median, Sequana, NGS, Python, Snakemake, CNV

## Background

Sequencing technologies allow researchers to investigate a wide range of genomic questions [1], covering research fields such as the expression of genes (transcriptomics) [2], the discovery of somatic mutations, or the sequencing of complete genomes of cancer samples to name a few examples [3, 4]. The emergence of the second generation sequencing, which is also known as Next-Generation Sequencing or NGS hereafter, has dramatically reduced the sequencing cost. This breakthrough multiplied the number of genomic analyses undertaken by research laboratories but also yielded vast amount of data. Consequently, NGS analysis pipelines require efficient algorithms and scalable visualization tools to process this data and to interpret the results.

Raw data generated by NGS experiments are usually stored in the form of sequencing reads (hereafter simply called reads). A read stores the information about a DNA fragment and also an error probability vector for each base. Read lengths vary from 35-300 bases for current short-read approaches [1] to several tens of thousands of bases possible with long-read technologies such as Pacific Biosciences [5, 6] or Oxford Nanopore [7].

After trimming steps (quality, adapter removal), most high-throughput sequencing (HTS) experiments will require mapping the reads onto a genome of reference [8]. If no reference is available, a de-novo genome assembly can be performed [9]. In both cases, reads can be mapped back on the reference taking into account their quality. We define the genome coverage as the number of reads mapped to a specific position within the reference genome. The theoretical distribution of the genome coverage has been thoroughly studied following the seminal work of Lander-Waterman model [10, 11]. A common metric used to characterize the genome coverage is the sequencing depth: the empirical average of the genome coverage. It may also be called depth of coverage (DOC), fold coverage, read depth, or confusingly, depth or coverage. The sequencing depth unit is denoted X. An example of a genome coverage with a sequencing depth of about 450 X is shown in Figure 1. Another useful metric is the breadth of coverage (BOC): the proportion of the intended genome reference covered by at least one read.

The required sequencing depth depends on the experimental ap-

Key Points

- We propose a novel algorithm to automatically detect genomic regions of interest (e.g., CNV) that depart from the overall genome coverage.
- Normalization is performed with an efficient running median. Using a mixture Gaussian model, we assign to each per-base coverage a z-score. A double threshold clustering is used to report the final list of genomic ROIs.
- We provide a standalone application called sequana_coverage – available in the Sequana [32] project.
- Starting from a BAM or BED file, HTML reports provide the coverage metrics, genomic ROIs, coverage versus GC content plot, genbank annotations, and Javascript visualisation (for viral and bacterial genomes).
- The tool handles multi-chromosomes genomes and provide multiqc [35] reports in such cases.
- Both short (SNVs) and long events (CNVs) are detected by the algorithm with accurate breakpoints reported.
- sequana_coverage was designed for viral and bacterial genomes but can also handle eukaryotes genomes.

plication. For instance, to detect human genome mutations, single-nucleotide polymorphisms (SNPs), and rearrangements, a 30 to 50 X depth is recommended [1, 12] in order to distinguish between sequencing errors and true SNPs. In contrast, the detection of rarely expressed genes in transcriptomics experiments often requires greater sequencing depth. However, greater sequencing depth is not always desirable. Indeed, in addition to a higher cost, ultra-deep sequencing (large sequencing depth in excess of 1000 X) may be an issue for a de-novo genome assembly [13].

The Lander-Waterman model provides a good theoretical estimate of the required sequencing depth to guarantee that all nucleotides are covered at least N times. This is, however, a theoretical estimate that does not take into account technical and biological limitations; some regions being difficult to efficiently map (e.g., repetitive DNA) or containing compositional biases (e.g., GC bias [14]). Furthermore, the genome coverage itself may contain a non-constant trend along the genome due to the impact of replication from the origin of replication. Finally, some regions may be deleted or duplicated. The genome coverage example shown in Figure 1 demonstrates these different features.

While the sequencing depth and other metrics (e.g. BOC) provides a quick understanding about the quality of sequencing and mapping, the genome coverage can also be analysed to identify genomic variations such as single nucleotide variations (SNVs) or copy number variations (CNVs) [15, 16, 17].

In order to detect genomic regions of interests (ROIs) based on genome coverage, a simple and fast approach might be to set two arbitrary thresholds bounding the sequencing depth. However, there are two major drawbacks with this approach. First, as shown in Figure 1 (top panel) and Notebook 4 in [45], with a fixed threshold, one may detect numerous false signals (type I errors) or fail to detect real events (type II errors). An adaptive thresholds that follows the trend of the genome coverage is thus required. Furthermore, a fixed threshold is arbitrary and so the detected events lack a robust means of assigning significance. A more robust alternative is to estimate the genome coverage profile histogram [18] from which a z-score statistics can be used to identify outliers more precisely. Due to a number of known and unknown biases, one should still normalize the data [15]. There are a number of different methods for detecting the ROIs. For example, for CNV detection, numerous techniques are used [17] such as the mean-shift technique [16] or bias correction followed by application of a complex statistical model [15].

In this paper we describe a novel approach that can efficiently detect various types of genomic ROIs. The algorithm does not target any specific type of genomic variations but instead systematically reports all positions (with a z-score) that have depth departing from the overall distribution. The algorithm normalizes the genome coverage using a running median and then calculate a robust statistic (z-score) for each base position based on the parameter estimation of the underlying distribution. This allows us to obtain robust and non-constant thresholds at each genome position. Various types of clustering or filtering can then be implemented to focus on specific categories of variations.

In the Data Description section, we describe the data sets used throughout the paper as test-case examples. In the Methods section, we describe (i) the running median used to detrend the genome coverage, (ii) the statistical methods used to characterize the central distribution from which outliers can be identified and (iii) a double thresholds method proposed to cluster the ROIs. Finally, in the Applications section, we describe the standalone application, sequana_coverage, and potential applications for HTS-dependant research projects including CNVs detection.

## Data Description

Three test-cases of genome coverage are presented here, covering representative organisms and sequencing depths. The genome coverage data sets are in BED (Browser Extensible Data) format, a tabulated file containing the coverage, reference (e.g., chromosome number, contig) and position on the reference. BED files can be created from BAM files (mapped reads) using `bedtools` [19], in particular the `genomecov` tool.

We first considered a bacteria from a study of methicillin resistant Staphylococcus aureus [20]. One circular chromosome of 3 Mbp is present. The sequencing depth is 450 X and the genome coverage exhibits a non-constant trend along the genome (see Figure 1). This pattern, often observed in rapidly growing bacteria, is the result of an unsynchronized population where genome replication occurs bi-directionally from a single origin of replication [21, 22]. The proportion of outliers (see Table 1) is about 2.5 % of the total bases. The original data sets (Illumina sequencing reads, paired-end, 100 bp) are available at the European Nucleotide Archive (ENA) [23] under study accession number PRJEB2076 (ERR036019). The accession number of the reference is FN433596.

The second organism is a virus with a sequencing depth of 1000 X [24]. A circular plasmid, containing the virus chromosome, is 19 795 bp-long. About 13% of the genome coverage contains large or low coverage regions (outliers). It also contains two large under-covered regions (one partially under-covered and one region that is not covered at all) as shown in the Notebook 1 of [45]). The accession number of the reference is JB409847.

The third test case is a fungus (Schizosaccharomyces pombe) [25]. The genome coverage has a sequencing depth of 105 X. It has three non-circular chromosomes of 5.5 Mbp, 4.5 Mbp and 2.5 Mbp. The

Compiled on: March 9, 2018.
Draft manuscript prepared by the author.

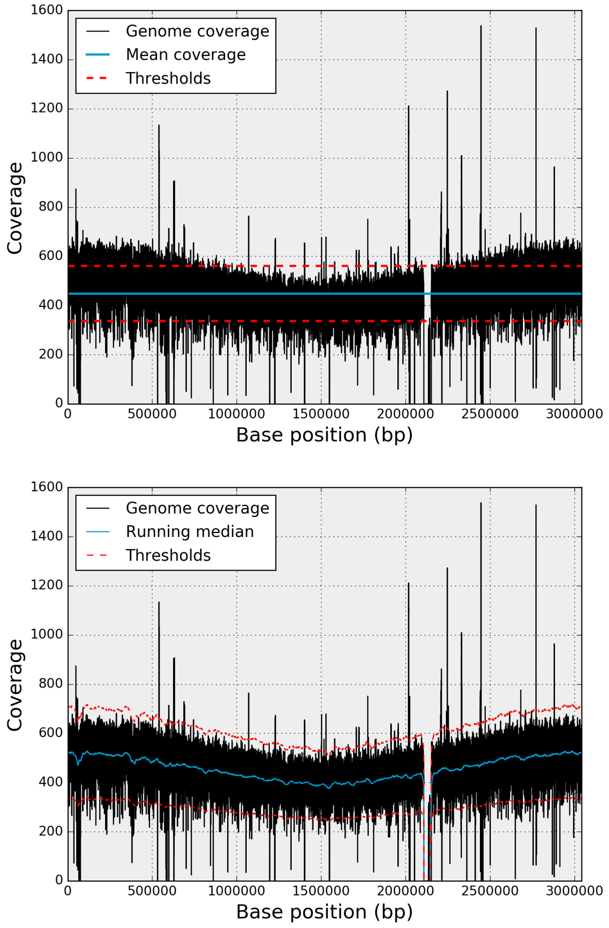


Figure 1. Example of a genome coverage series (in black in both panels). The genome coverage corresponds to the bacteria test case (see text). It contains a deleted region (around 2.2 Mbp) and various under- and over-covered regions (from 100 bp to several Kbp). Although the sequencing depth is about 500 X, there is non-linear trend from 500 X on both ends to 400 X in the middle of the genome. The top panel shows the sequencing depth (blue horizontal line) and two arbitrary fixed thresholds (dashed red lines) at 400 X and 500 X. Due to the non-linear trend, the fixed thresholds lead to an increase of Type I and Type II errors. On the contrary, in the bottom figure, the trend is estimated using a running median (red line) and adaptive lower and upper thresholds (dashed red lines) can be derived.

references from ENA are CU329670.1, CU329671.1 and CU329672.1. Although we will look at the first chromosome only (1.5% of outliers), the tools presented hereafter handles circular chromosomes and multiple chromosomes. See examples in Notebook 3 of [45].

We provide the 3 genome coverage data files in BED format on Synapse [26, 27]. See Section Availability of supporting data and materials for more details.

In addition to these three single-sample cases, we also use a population composed of 6 Staphylococcus aureus isolates from [15] (supplementary), which is used to measure the efficiency of our algorithm against two dedicated CNVs detection tools: CNOGpro [15] and CNVnator [16].

## Methods

### Detrending the genome coverage

The genome coverage function is denoted C(b) where b is the base (nucleotide) position on the genome of reference. The genome coverage and reference lengths are denoted N. For simplicity, we drop the parentheses and refer to the genome coverage as $C_b$. The empirical sequencing depth (average of genome coverage) is denoted $\delta = \overline{C_b}$. Ideally, $C_b$ is made of a continuous homogeneous central region. In practice, however, this may be interrupted by a succession of under- and over-covered regions: the genomic ROIs that we want to detect.

A naive classifier consists in setting two fixed thresholds $\delta^-$ and $\delta^+$ whereby low and high ROIs are defined as $C_b^- = C_b \leq \delta^-$ and $C_b^+ = C_b \geq \delta^+$, respectively. If $C_b^0$ denotes the remaining data such that $\delta^- < C_b^0 < \delta^+$, then the genome coverage can be written as $C_b = \{C_b^0, C_b^+, C_b^-\}$.

The advantage of the fixed-thresholds method is that it is conceptually simple and computationally inexpensive. However, there are two major drawbacks manifest. First, as shown in Figure 1-A, false negatives and false positives will increase as soon as there is a non-constant trend present in the data. It may be a low frequency trend as shown here but high frequency trend are also present (see e.g., Figure 2). Also of importance is that an arbitrary choice of threshold(s) is unsatisfactory from a statistical point of view since we cannot associate any level of significance to a genomic region.

In order to account for a possible trend in the genome coverage series (and remove it), a standard method consists in dividing the series by a representative alternative such as its moving average or running median.

The moving average (MA) is computed at each position, b, as the average of W data points around that position and defined as follows:

$$MA_W(b) = \frac{1}{W} \sum_{i=-V}^{V} C(b+i), \quad (1)$$

where W is the length of the moving window (odd number) and $V = (W-1)/2$. Note that the first and last V values are undefined. However, in the case of circular DNA (e.g., viral or bacterial genomes), then the first and last V points are defined since $C_b$ is now a circular series.

Similarly, the running median (RM) is computed at each position, b, as the median of W data points around that position:

$$RM_W(b) = \text{median}(\{C(b-V), .., C(b+V)\}), \quad (2)$$

where W and V are defined as before and the median function is defined as the middle point of the sample set (half of the data is below the median and half is above). A mathematical expression of the median and running median are given in the Appendix section (Eq. 8).

The mean estimator is commonly used to estimate the central tendency of a sample, nevertheless it should be avoided in the presence of extraneous outliers, which are common in NGS genome coverage series (see e.g., Figure 1). Figure 2 shows the impact of outliers when using a moving average or a running mean. We will use the running median only and define the normalized genome coverage as follows:

$$\widetilde{C}_b = \frac{C_b}{RM_W(b)}. \quad (3)$$

We will use the tilde symbol for all metrics associated with the normalized genome coverage, $\widetilde{C}_b$. For instance, $\widetilde{C}_b = \{\widetilde{C}_b^0, \widetilde{C}_b^+, \widetilde{C}_b^-\}$.

The running median is used in various research fields, in particular in spectral analysis [28] to estimate the noise floor while ignoring biases due to narrow frequency bands (e.g., [29]). Here, the goal is to avoid narrow peaks but also to be insensitive to long deleted regions. This can be a major issue in NGS as the running median estimator complexity is a function of the window length. Indeed the running median algorithm involves the sorting of a sample of length W at each position of the genome. So, the running median estimator must be efficient and scalable. This is not an issue in spectral analysis and most fields where running median are used but is a bottleneck

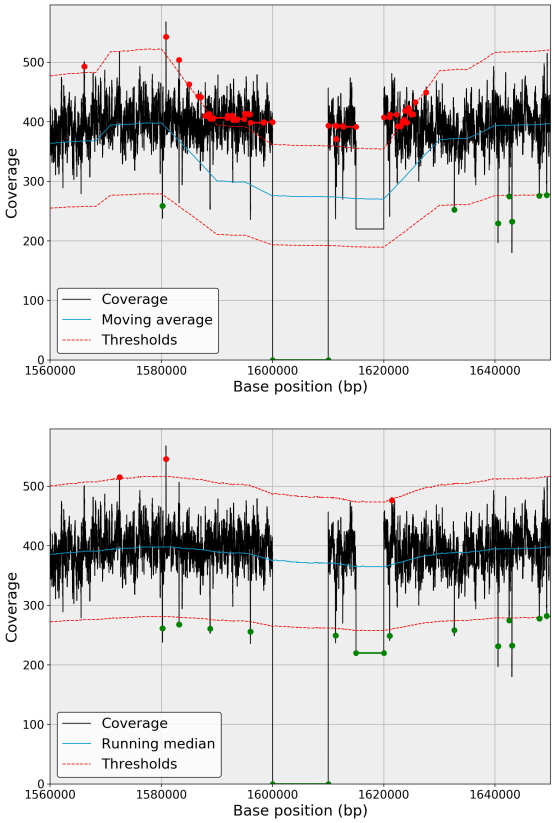


Figure 2. Moving average (top panel) and running median (bottom panel) behaviour in presence of outliers (here, a deleted region in the center followed by a depleted region). In both cases, the window parameter is set to 40,000 bases. The presence of the deleted and depleted regions shows how the trend (blue line) moving average estimation can be shifted as compared to the running median. The thresholds (red lines) are also shifted and the depleted region (position 1,620,000) is not detected. The rate of false detection increases (red dots).

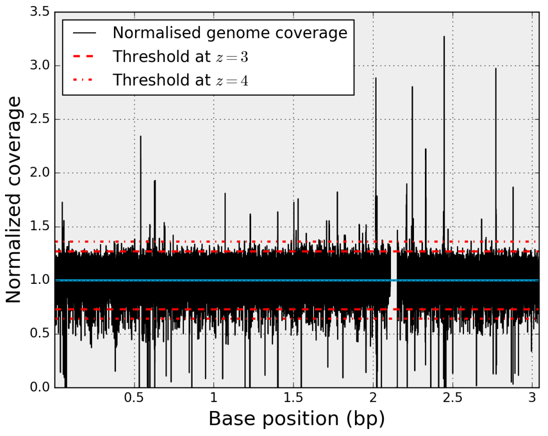


Figure 3. Normalized genome coverage $\widetilde{C}_b$ (bacteria test case). The outliers present in the original genome coverage $C_b$ (see Figure 1) are still present as well as the deleted regions. The distribution is now centred around unity (blue line). Since the distribution is normalized, constant thresholds can be used (dashed lines).

for NGS analysis where W is large. As explained in the Appendix section, the complexity of the sorting part is in $O(n^2)$ in the worst case but similarly to the moving average, one can take advantage of the rolling window and the fact that the previous block is already sorted. We opted for the very efficient Pandas [30] implementation (See Appendix for details). In our implementation, both the moving average and running median have the ability to account for circular DNA data, which is essential to handle circular series.

If we normalize the genome coverage from the bacteria example (Figure 1), we obtain the results shown in Figure 3. Finally, note that the genome coverage being discrete, the running median is also discrete as well as the normalized genome coverage. The discreteness will become more pronounced as sequencing depth decreases.

Hereafter, we will discuss the impact of the W parameter on the detection of genomic ROIs and how to set its value.

## Parameter estimation of the central distribution and adaptive thresholds in the original space

In the ideal case of randomly distributed reads across the genome, the number of reads covering each base follows a Poisson distribution [10]. This distribution is discrete and has one parameter that corresponds to the sequencing depth (mean of the distribution). Yet, the Poisson distribution is often too narrow [18], as can be observed in the three test cases considered. This is generally due to biological over-dispersion. In order to account for over dispersion, the Poisson parameter can be distributed according to a second distribution. For instance when the Poisson parameter is distributed according to a Gamma distribution, we obtain a negative binomial, which has two shape parameters [18].

A Poisson distribution with a large mean parameter approximates a normal distribution, even though, technically, it is not (discrete versus continuous and one parameter versus two). Yet, for $\delta \gg 1$, we can assume that the $C_b$ distribution exhibits a Gaussian distribution denoted $\mathcal{N}(\mu, \sigma^2)$ hereafter where $\mu$ is the average of the genome coverage ($\delta$ in an ideal case) and $\sigma$ is its standard deviation. What about the normalized genome coverage $\widetilde{C}_b$? It is a ratio distribution where the numerator follows $\mathcal{N}(\mu, \sigma^2)$ distribution while the denominator's distribution is that of the running median. We can see empirically that for large $\delta$ and small W parameter, the distribution of the running median follows a Gaussian distribution while for large W or small $\delta$ the running median tends to be discrete and the distribution may depart from a Gaussian distribution (See Notebook 7 of [45]). Even if we knew the running median distribution, the ratio distribution is only known for two Gaussian distributions X and Y (Cauchy distribution) and when (i) the two distributions are centred around zero, which is not the case, and (ii) when they are independent, which is also not the case. Furthermore, the scenario we considered (ideal distribution, $\delta \gg 1$) is too restrictive since we are interested in identifying outliers in real data and may encounter cases where $\delta$ is small (for which $C_b$ follows a negative binomial, not a Gaussian distribution).

Genome coverage is a mix of distributions. Consider for instance the presence of many CNVs, each with a different copy number (either depletion or duplication). The overall distribution here would be very difficult to model analytically. Therefore, the assumption and our goal is to fit a known distribution on the central distribution so as to establish z-scores on the remaining data.

Our first hypothesis is that $\widetilde{C}_b$ can be decomposed into a central distribution, $\widetilde{C}_b^0$, and a set of outliers, $\widetilde{C}_b^1 = \{\widetilde{C}_b^+, \widetilde{C}_b^-\}$ where the central distribution is predominant: $\left|\widetilde{C}_b^0\right| > \left|\widetilde{C}_b^1\right|$ (vertical bars indicate the cardinality of the sets).

Our second hypothesis is that the mixture model that represents $\widetilde{C}_b$ is a Gaussian mixture model of $k = 2$ models only: $\widetilde{C}_b^0 \sim \mathcal{N}(\widetilde{\mu}_0, \widetilde{\sigma}_0^2)$ and $\widetilde{C}_b^1 \sim \mathcal{N}(\widetilde{\mu}_1, \widetilde{\sigma}_1^2)$. The central distribution $\widetilde{C}_b^0$ exhibits a clear Gaussian distribution both on simulated data (see Notebook 7 in [45]) and on real data (see the three examples in Figure4). The second model is used to identify outliers (below or above the central distribution). The parameters of the second model are not used in defining the central distribution so have little impact on detection.

Similarly to the method deployed in [18] to identify a mixture model of negative binomials (on raw genome coverage), we will use an Expectation Maximization (EM) [31] method to estimate the parameters $\widetilde{\mu}_{0,1}$ and $\widetilde{\sigma}_{0,1}$ (on the normalised genome coverage).

The EM algorithm is an iterative method that alternates between two steps: (i) an Expectation step that creates a function for the expectation of the log-likelihood using the current estimate of the parameters, and (ii) a Minimization step that computes parameters maximizing the expected log-likelihood found in the first step. The likelihood function and the maximum likelihood estimate (MLE) can be derived analytically in the context of Gaussian distributions. Note that in addition to the means and standard deviations, the mixture parameters also need to be estimated. These are denoted $\widetilde{\pi}_0$ and $\widetilde{\pi}_1$. The EM algorithm is standard and can be found in various scientific libraries. Note, however, that the normalized genome coverage may contain zeros in the presence of deleted regions and the estimation of the mixture model should ignore them.

We have applied the EM algorithm on the normalized genome coverage vector on various real NGS data sets including the three test cases in Figure 4. The EM retrieves the parameters of the central distribution (in particular $\tilde{\mu}_0 = 1$) and the outliers. Note that the choice of the running median parameter, W, does not significantly affect the parameter estimation. In each case, the mean of the central distribution is very close to unity. The standard deviation varies significantly and is a function of the sequencing depth only (since the outliers are now incorporated in $C_b^1$). Finally, we can confirm that the proportion of outliers is small as compared to the central distributions by inspection of parameters $\pi_0$ and $\pi_1$: $\widetilde{\pi}_0 >> \widetilde{\pi}_1$.

Once we have identified the parameters of the central distribution $\widetilde{C}_0$, we can assign statistics for $\widetilde{C}_b$ in terms of z-score:

$$z(b) = \frac{\widetilde{C}(b) - \widetilde{\mu}_0}{\widetilde{\sigma}_0}. \quad (4)$$

Since the z-score corresponds to a normal distribution, we can now set a threshold in terms of tolerance interval within which a specified proportion of the genome coverage falls. For instance, with a threshold of 3, we know from the normal distribution that 99.97% of the sample lies in the range -3 and +3. The exact mathematical value is given by the complementary error function, erfc(x), where $x = n/\sqrt{2}$. Note that for n = 3, 4 and 5, the tolerance interval is 99.73%, 99.993% and 99.999942%, respectively. Thus, for a genome of 1 Mbp, by pure chance we should obtain about 2700, 70 and 1 outlier(s), respectively.

If we now replace $\widetilde{C}_b$ in Eq.4 using its expression from Eq. 3, we can express the original genome coverage as a function of the running median, the z-score and the parameters of the central distribution:

$$C(b) = \left(\widetilde{\mu}_0 + z(b)\widetilde{\sigma}_0\right) RM_W(b). \quad (5)$$

We can now set a fixed threshold $z(b) = \pm n$ in the normalized space. This is much easier to manipulate. Moreover, we can derive a variable threshold in the original space that is function of the genome position:

$$\tilde{\delta}^{\pm}(b) = \left(\widetilde{\mu}_0 \pm n^{\pm} \times \widetilde{\sigma}_0\right) RM_W(b). \quad (6)$$

Examples of variable upper and lower threshold functions are shown in Figure 1 and Figure ?? (red dashed lines). This manipulation results in a robust statistical estimate of the presence of outliers in the genome coverage. The z-score, computed earlier, provides a precise level of confidence.

Using the normalization presented above, we can define the centralness as one minus the proportion of outliers contained in the genome coverage:

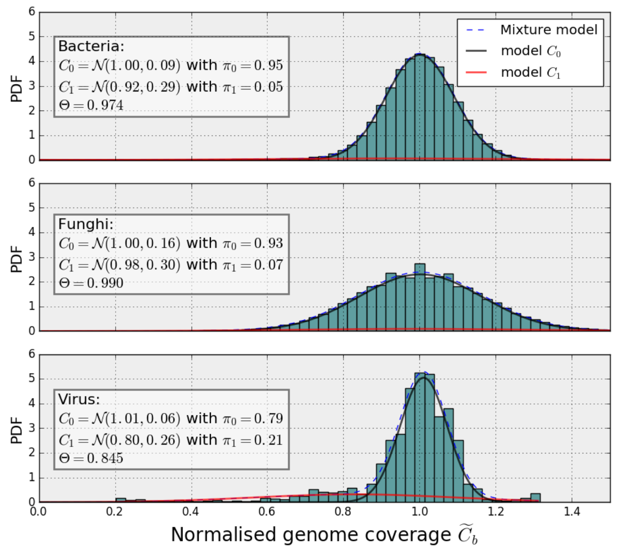


Figure 4. Probability density functions (PDFs) of the normalized genome coverage function concerning the three test cases. The distributions were fitted with a Gaussian mixture models with k = 2 models. The first model (black line) fits the central distribution's PDF and the second model (red line close to y = 0) fits the outliers' PDF. The dashed lines (close to the black lines) indicates the mixture distribution. In each panel, we report the parameters of the two Gaussian distributions, the proportions $\pi_0$, $\pi_1$ and the $\Theta$ parameter introduced in the text that gives the centralness of the data for each test cases.

$$\Theta_n = 1 - \frac{\left|\widetilde{C}_b^1\right|}{\left|\widetilde{C}_b\right|} = 1 - \frac{\left|\widetilde{C}_b^1\right|}{G}, \quad (7)$$

where G is the length of the genome, and vertical bars indicate the cardinality. This necessarily depends on how the threshold n is set in the normalized space. In the case of an ideal Gaussian distribution and n = 3, the centralness should equal the tolerance interval of a normal distribution $\mathcal{N}(0, 1)$ that is the error function, $\mathrm{erf}(n/\sqrt{2})$. The centralness equals unity when there are no outliers i.e., $n \to \infty$. Finally, note that the centralness is meaningless for values below 0.5 (meaning that the central distribution is not central!). As shown in Table 1, $\Theta_3$ equals 0.974, 0.99 and 0.86 in the three cases considered (bacteria, fungus, virus). So the proportion of outliers in the virus case is higher than in the two other test cases, which is not obvious at first glance given the very different lengths of the genome considered.

Finally, it is important to note that the z-scores assigned to each position on the genome coverage are robust with respect to the choice made for the W parameter. As shown in the Notebook 7 of [45], the mean and standard deviation of the distribution of the normalised genome coverage are not affected by the choice of W. Moreover, the parameters of the central distribution estimated with the EM algorithm take into account any effects induced by W.

## Genomic ROIs

From the normalized genome coverage, $\tilde{C}$, and the estimation of its central distribution parameters, a z-score is assigned to each position. All values above the threshold $n^+$ are stored into a subset of events denoted $\widetilde{C}_b^+$ and all values below the threshold $n^-$ are stored into $\widetilde{C}_b^-$. The selected data can be continuous or non-continuous regions. The number of events can be quite large for low thresholds (e.g., for $n^+ = 2.5$, the bacteria has 35 Kbp such events). However, many positions belong to the same event (i.e., same cluster). Considering the short genomic region in Figure 5, which is made of 2000

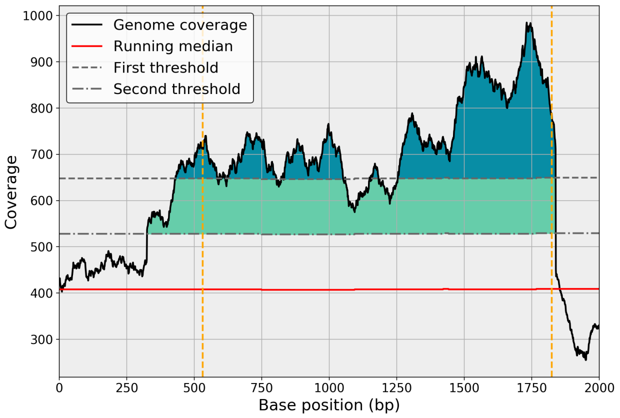


Figure 5. Example of a genomic region of interest (ROI) clustered using a double threshold method. The genome coverage (black line) and its running median (red) on a short genome location of 2 kbp. The first threshold (top dashed gray line) alone identifies many short ROIs (dark blue areas). Using a second threshold (bottom dashed gray line), the short ROIs are clustered and identified as a single ROI (coloured areas). Yellow vertical lines indicates the beginning and end of the cluster.

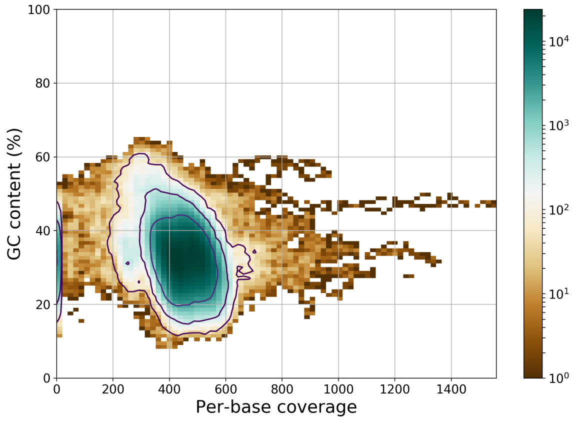


Figure 6. 2-dimensional histogram of the GC content versus coverage available in the HTML reports. The data used correspond to the bacteria test case. We can quickly see that (i) the mean coverage is around 450, (ii) the mean GC is around 30 % (iii) there are part of the genome coverage with zero coverage (left hand side blue line), (iv) there are low and high ROI with coverage up to 1500X that would possibly require more investigations. Be aware of the logarithmic scale: most of the data is indeed centered in the blue area and the brown outliers represent less than a few percentage of the data.

base positions. It contains 5 different regions that cross the threshold $n^+$. Ideally, the 5 events should be clustered together. To do so, we proceed with a double-threshold approach [29] where a second fixed threshold $m^+$ is defined as $m^+ = \alpha^+ n^+$ where $\alpha^+ \leq 1$ and usually set to 1/2.

In the normalized space, the double threshold method works as follows. We scan the entire genome coverage vector starting from the first position $b = 0$. As soon as a per-base coverage value crosses the threshold $m^+$, a new cluster starts. We then accumulate following bases until the per-base coverage crosses $m+$ again (going down). If the maximum of the cluster is above the first threshold, $n^+$, then the cluster is classified as a region of interest. The process carries on until the end of the vector is reached. We repeat this classification for the lower case (with $m^- = \alpha^- n^-$). This method dramatically reduces the number of short ROIs. Finally, we can characterize each region with various metrics such as the length of the region, maximum coverage, mean coverage. If consecutive data points were independent, we could also report a z-score for large events (probability that an event of length N crosses a pre-defined threshold). Instead, for simplicity, we report the mean and max z-score of the event only.

### Impact of the running median window parameter

In order to estimate the general trend of the genome coverage, we should avoid the impact of deleted, duplicated or depleted regions. Because the median takes the middle point of a segment, the parameter W should be set to 2N where N is the longest atypical genomic region present in the data. For instance, an expected CNV region with a length of 50,000 would imply setting $W = 100,000$ so that the genome coverage trend remains appropriate (see Notebook 6 in [45] for a counter example). Since such regions are not known in advance, W should be as large as possible so as to avoid the presence of any long regions that depart from the central distribution. Yet, over-increasing W may have undesired effects. For instance, in the extreme case where W is set to the full genome length, one would obtain the same value all along the genome (the sequencing depth itself) and thus lose the interest of the running median by introducing too much smoothing. This could lead to an increase of false detections or missed detections. By default, we recommend to set W to 20,000. Indeed, below this value, it seems that there is a slight increase of marginal false detections while for values in the range $W = 20,000$ to 500,000, the list of ROIs is similar (see Notebook 6 in [45]). As mentioned above, the impact of the W parameter on the z-scores is marginal so one can safely change it from 20,000 to 100,000. A strategy could be to run two analysis: one with $W = 20,000$ to list the short events, and one with very large W for longer events.

| Metric | Bacteria | Fungus | Virus |
|---|---|---|---|
| Genome length | 3 Mbp | 5.5Mbp | 19795 |
| BOC | 0.985 | 1.0 | 0.966 |
| mean $\delta$ | 447.8 | 105.49 | 931.3 |
| median $\delta$ | 453 | 105 | 988 |
| $\sigma$ | 84.1 | 19.9 | 237.2 |
| CV | 0.19 | 0.19 | 0.25 |
| W | 5001 / (20001) | 5001 / (20001) | 5001 |
| $\widetilde{\mu}_0$ | 1.000 / (1.001) | 1.002 / (1.002) | 1.011 |
| $\widetilde{\sigma}_0$ | 0.073 / (0.073) | 0.162 / (0.158) | 0.069 |
| $\Theta_4$ | 0.957 / (0.960) | 0.986 / (0.985) | 0.868 |

Table 1. Metrics derived from the genome coverage of the three test cases considered (Bacteria, Fungus, Virus). The top part of the table contains metrics derived from the genome coverage only, while the bottom part contains metrics derived from the normalized genome coverage, $\widetilde{C}_b$. All metrics are defined in the text; BOC stands for breadth of coverage, $\delta$ for sequencing depth, CV for coefficient of variation. The standard deviation is denoted $\sigma$. In the bacteria and fungus cases, the running window W is set to 5 001 or 20 001 while for the virus we used 5 001 only. The parameters of the central distribution, $\widetilde{\mu}_0$ and $\widetilde{\sigma}_0$ and the centralness, $\Theta_3$ are reported. Proportion of outliers (1-$\Theta_3$) are about 4.5, 1.5 and 13% for the bacteria, fungus and virus, respectively.

## Applications

### Standalone and computational time

Although the algorithm described here above is quite simple per se, each of the three steps required optimization in order to handle HTS data sets. We provide an implementation within the **Sequana** project [32], which is a Python library that also provides HTS pipelines based on the workflow management system called Snake-

make [33] (Makefile-like with a Python syntax). Standalone applications are provided including **sequana_coverage**. In addition to the algorithm described above, the standalone application has several additional features as explained. The input file can be either a BAM or a BED file [19] encoded as a 3-column tab delimited file (chromosome, position, coverage). Consider this command:

```
sequana_coverage --input virus.bed -w 4001 -o
```

The -o option indicates that the input is a circular DNA molecule. The running median window can be tuned using the -w option. Several chromosomes may be present (e.g., fungus case). By default, all chromosomes are analysed but users can select a specific one using the -c option. Other useful options are the ability to change the thresholds on the z-score, ability to cluster close ROIs or to analyse the data by chunks (useful for large eukaryotes genomes). An additional feature is the ability to download a reference genome (given its ENA [23] accession number). This is achieved internally using BioServices [34] which can switch between the ENA or NCBI web services to download the data automatically. Regions of lower genome coverage are sometimes related to repeated content or unusual GC content [36]. Using the reference, we provide a GC content versus coverage plot in the report as shown in Figure 6. Genbank annotations can also be downloaded to annotate ROIs.

The output is a directory that contains, for each molecule: (i) an HTML report, (ii) a summary file (JSON format) and (iii) a CSV file with detected ROIs. In addition, we provide a multiQC report [35] via a plugin available in the Sequana library. The mutliQC report contains a summary of the mapping metrics including the DOC and BOC metrics, the number of ROIs and the centralness (defined in this manuscript). The CSV file is structured with one ROI per row, including information such as the location, length, mean z-score, mean coverage, . . . In the individual HTML reports, **JavaScript** plots are provided together with the ROIs for a quick inspection (not available for large genome >5Mbp).

Finally, the standalone application is designed to be scalable: the virus genome is analysed in a few seconds while the 5 Mbp bacteria genome is analysed in about one minute on a standard computer including analysis and HTML reports (Python implementation). Although the standalone was initially designed for bacterial genomes (genome could fit in memory), we extended the functionality so that larger genomes could also be analysed. In particular, we looked at human genome used in [16]. Although the algorithm is not designed for this lower DOC (around 5X), as the central distribution does not follow a Gaussian distribution, the genome coverage can still be analysed. Thresholds were increased (from 4 to 6) to avoid an abundance of false detections. The 3.5Gb genome could be analysed in a few hours, which is competitive with CNVnator. This required adding an option called binning that merges data before analysis. Similarly to the CNVnator implementation, this reduces the breakpoint accuracy and prevents the tool from identifying short events.

## CNV detection

In extending the functionality of **sequana_coverage** to include larger genomes, we also explored its use in detecting copy number variations (CNVs). CNV detection methods can be categorized into five different strategies depending on the input data: paired-end mapping, split-read, read depth (i.e., genome coverage), de novo assembly and combinations of the above approaches. Amongst the numerous tools based on the genome coverage reported in [17], we considered CNVnator [16], which is able to detect CNVs in various sizes ranging from a few hundred bases to mega-bases. CNVnator can also handle whole genome data sets and exhibits a good precision at detecting breakpoints. We then also consider a more recent tool called CNOGpro [15], which is dedicated to prokaryotic whole genome sequencing data. As stated in [17], none of the various tools have been able to detect the full spectrum of all types of CNVs

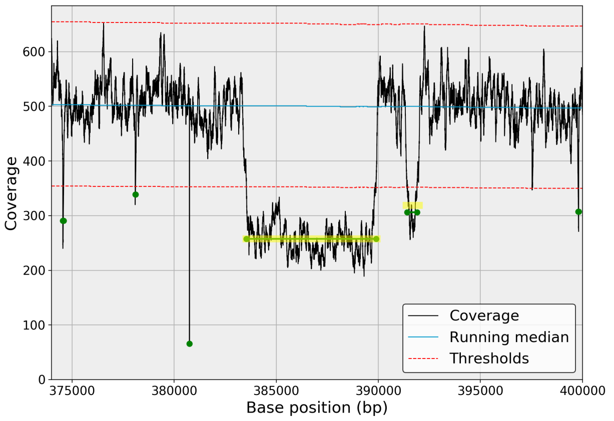


Figure 7. Detection of a depleted region (copy number 0.5). CNVnator (thick yellow segments) and sequana_coverage (thin green segments and green dots) identifies the 6,300 long event with the correct location and similar copy number (based on the mean of the data). sequana_coverage identifies the other depleted region of about 500 bases at position 392,000. CNVnator ability to detect that event depends on the bin parameter: missed for a value of 1 or 100, found with a value of 6. All short events (few bases long) are missed by CNVnator. Conversely, CNVnator is able to identify very long CNV regions up to mega-bases.

with high sensitivity and specificity. A combinatorial approach that would take advantage of different methods to increase the performance in detecting CNVs and reduce false positives is preferred.

We first examined the sensitivity and specificity of sequana_coverage on simulated data. Technical details can be found in the Notebook 5 [45]. Simulated paired-end data were used to create 100X genome coverage data for Staphylococcus aureus. The number of ROIs detected with sequana_coverage varies from one simulation to the other but the main trend is that reported ROIs have short lengths (below 50) and low mean z-scores (below 5); some rare events may have length of 100 bases. There are a small number of false positives (1 or 2 on a 3Mbp genome) when searching for CNV of size above 100 bases. We then injected 3 sets of CNVs: First, we deleted 30 non-overlapping regions (length between 1,000 and 8,000). We achieve a high sensitivity with all deleted regions reported with starting/ending positions accuracy of a few bases. Second, we duplicated 80 non-overlapping regions (same length as above, CN=2). Again, we have a 100% sensitivity with high location accuracy. Sequana_coverage stores a value called log2_ratio for each ROI . This value is the ratio of the mean coverage and mean running median for that ROI and is equivalent of copy number. The average copy number reported for the 80 injected CNVs (W=40,000 bases) is CN $= 1.96 \pm 0.04$ (slightly biased). Third, we injected a mix of 80 depleted and duplicated events (same length as above) at a coverage of 150X (CN=1.5) or 50X (CN=0.5). The 80 events are found again with slightly-reduced accuracy (still below 20 bases). The CN reported for duplicated and deleted events is $1.49 \pm 0.023$ and $0.5 \pm 0.026$, respectively. The simulated data indicates that the algorithm can detect short CNVs (from 1000 to 8000) with high sensitivity, accurate estimate of copy number and location.

For a comparison against published tools using real data, we examined the Staphylococcus aureus case used in [16]. We ran sequana_coverage and CNVnator on the 3Mbp genome. CNVnator has a parameter called bin, which is essentially used to define the breakpoint resolution accuracy. We used a bin parameter of 1, 6 and 100 (default) where 6 was chosen as the optimal bin size for the sequencing depth considered (500X). Here, we refered to the instructions found in [16] that led to an empirical equation bin $= 2500/\text{DOC}$ (see also Notebook 8 [45]). All results can be found in the Notebook 9 in [45]. The number of events reported by CNVnator are 207, 72 and 13, for bin = 1, 6 and 100 respectively.

With sequana_coverage, W was set to 40,000 bases. The number of reported events is about 600 events (quite stable with respect to W parameter). Only 200 events have a size larger than 10 bases and a mean z-score above 5. All events reported by CNVnator with a bin = 6 or 100 are also detected by sequana_coverage with the same breakpoint resolution. The additional CNVnator events, obtained with bin = 1, are mostly false positives (see Notebook 9 for examples). Visual inspection of events reported by sequana_coverage – but not found by CNVnator – show that they are close to the threshold and appear to be real events (see example in Figure 7). In terms of computational time, sequana_coverage takes one minute on this 3Mbp genome, irrespective of W, while CNVnator takes about 25 minutes, 5 minutes and 40 seconds for the bin = 1, 6 and 100, respectively.

Although CNVnator is more suitable for larger genome sequencing data, we also analysed the viral test case and compared the results with sequana_coverage. This 18 Kb viral genome contains (i) 3 SNVs (coverage of zero) of length 3, 1, and 1 bases; two of them are separated by only 2 bases (ii) two CNV-like events (700 and 800 bases long) and (iii) two short depleted regions with a low signal-to-noise ratio, which are ignored hereafter. Plots are available in the Notebook 10 of [45]. In summary, CNVnator yields different results depending on the bin parameter. With a bin set to 5 (optimal), the two CNV-like events are detected, but not the SNVs ones. Two false positives are called. With a bin parameter of 10 or 20, the two CNV-like events are detected while SNVs are still not detected. Finally, with a bin parameter set to 1, almost the entire genome is classified into 8 different CNVs (2 corrects, 6 false positives) and the SNVs are still missed. With sequana_coverage, irrespective of the window parameter (1000, 2000, 3000, 4000, 5000), the CNVs and SNVs events are correctly detected in addition to the two short depleted regions. Importantly, the running median is not effective for a W parameter which is too small (here 1000). For viral genomes, we would recommend to set W to a quarter of the genome size (here 4000).

We next looked at a comparison using a population of six isolates of Staphylococcus aureus from. The six data sets have a wide range of sequencing depth: 165, 61, 36, 94, 1100, 34, for the isolate ERR043367, ERR043371, ERR073375, ERR043379, ERR14216 and ERR316404, respectively. We compared the results provided in the supplementary data of [15] with those obtained by running sequana_coverage and CNVnator. Amongst the 5466 segments reported in [15], 43 are reported to have a copy number (CN) different from unity. Of the 43 events, 7 are false positives while the remaining are confirmed with sequana_coverage and visual inspection. It is important to note that, unlike CNVnator and sequana_coverage, which rely on the data to find the breakpoint of the ROIs, CNOGpro breakpoints are based on annotation and individual gene (or intergenic segment) assuming that duplications and deletions work at the gene level. Indeed, visual inspection reveals that many of CNOGpro events are sub sets of a larger event. This does not always look appropriate unlike the results provided by CNVnator or sequana_coverage, as shown in Figure 8. For the same reason, several narrow events found in the same intergenic segment will be averaged together whereas sequana_coverage reports the events individually as demonstrated in Figure 9. We also ran CNVnator, with the bin to the optimal value (see above), and for sequana_coverage, we set the W parameter to 40,000. The detected events between CNVnator and sequana_coverage are generally consistent in location and copy number. Both tools have a very good breakpoint accuracy as shown in Figure 8 with the main difference being that sequana_coverage splits events with gap in between (correctly from our point of view). Again, CNVnator is optimised to detect long CNV events and may miss narrower events, even if those event have large variations, as shown in Figure 9.

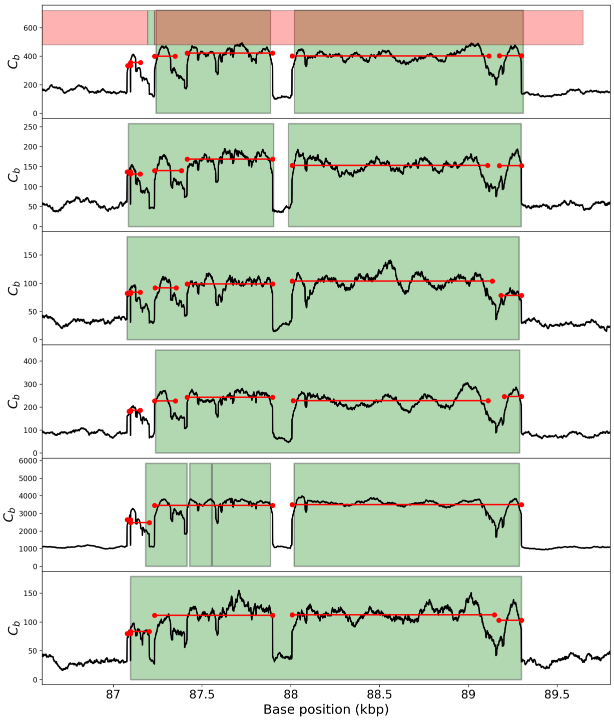


Figure 8. Detection and segmentation of complex events in a population sample. We focus on the region between positions 86,500 and 90,000. We analyse the data (black lines) with sequana_coverage (horizontal colored segments) and CNVnator (green areas). We also report the results of CNOGpro (red areas in the top panel only). CNOGpro detects the complex event as a single event with poor breakpoint resolution (end location is offset by 300 bases); see text for an explanation. CNVnator detects 1 event in 3 isolates, 2 events in 2 isolates) and 4 events in 1 isolate (fifth row); the gap in the middle of the genomic region considered is missed in 50% of the cases; breakpoint resolution is high. sequana_coverage reports 4 to 6 events; the breakpoint resolution is high; the event in the middle is systematically ignored, as it should be given its length of about 100 bases.

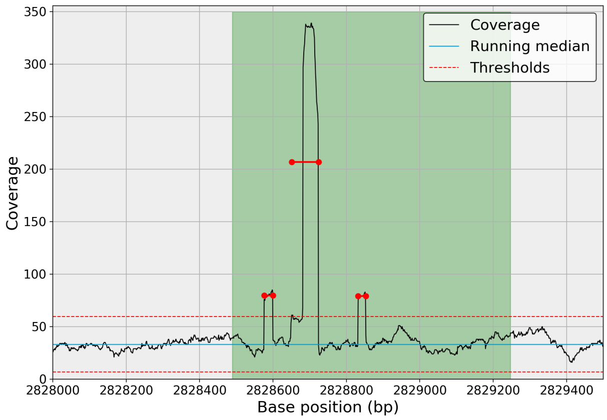


Figure 9. Narrow event made of a strong central peak (copy number CN=10) and two secondary weak peaks (CN=2.5). The 3 peaks can be identified visually in the 6 isolates. In this plot, we only show the isolate ERR316404, which is representative of the 6 others. The algorithm designed in sequana_coverage detects the main peak (CN=5) and the secondary peaks with CN=2.5 (red segments). Note that in the 6 isolates the main peak is detected while the secondary peaks are reported in 66% of the cases (8 peaks out of 12). CNVnator does not detect those events in none of the 6 isolates most probably because the length of those events (irrespective of their strength) are too short. CNOGpro detect 1 event shown here as the green area with a CN=2 for the overall event (although indicating large error with possible value between 1 and 5).

## Conclusion

The method presented in this paper provides a robust statistical framework to detect under and over-covered genomic regions that are then further annotated (length, mean coverage, maximum z-score, . . . ). Although robust, the method is straighforward and can be summarized in three main steps: (1) detrending of genome coverage series using a running median (ii) parameter estimation of the central distribution of the normalized genome coverage series using an EM approach (for a Gaussian mixture model), (iii) clustering and characterization of the outliers as genomic regions of interest (ROI) using a double threshold clustering method.

We underlined the value of the running median algorithm as compared to a moving average while emphasizing the practical impact of the running median algorithm complexity. An efficient implementation is of paramount importance in the context of HTS analysis. In addition, circular molecules and multi-chromosome organisms are handled.

We implemented the algorithm within the standalone application `sequana_coverage`, which also provides HTML reports with a summary of the genomic ROIs. The HTML reports provide easy visual inspection of genome coverage, list of genomic ROIs and statistics such as the centralness, a metric that encompasses the preponderance of the central distribution with respect to the outliers.

We presented test cases with relatively large sequencing depth (30X to 1000X), although we believe that the algorithm can be for sequencing depths as low as 10X. A natural extension to this work is to consider sequencing depths below 10X by using a mixture model of binomial models instead of gaussian models.

One obvious application of the algorithm presented is the systematic identification of SNVs or CNVs in a single sample or population of samples. We have shown that sequana_coverage is competitive with dedicated tools such as CNOGpro and CNVnator. We believe that sequana_coverage could be used in a combinatorial approach with existing tools to complement and complete the toolkit of CNV detection.

The tool is also relatively fast. Viral and bacterial genomes can be analysed in less than a minute. For larger Eukaryotic genomes (human), once the individual BED files are created for each chromosome, the analysis of the 24 files could be as low as 30 minutes on a standard dual-core. A Snakemake pipeline was also recently implemented in Sequana [32] allowing a human genome to be analysed in less than XX minutes thanks to parallel analysis of the individual chromosomes. A graphical interface using Sequanix [40], a Snakemake GUI is also available, which would make the configuration of the parameters and execution of the analysis on a cluster straightforward.

With additional features such as the ability to annotate the ROIs with genbank files and the identification of repeated regions, we believe that the standalone application `sequana_coverage` will help researchers in deciphering the information contained in the genome coverage. Finally, numerous notebooks, examples and code available in Sequana [32] and [45] should be helpful for integration in other libraries.

## Availability of source code

- Project name: Sequana (sequana_coverage standalone), version 0.7.0
- Project home page: http://sequana.readthedocs.org
- Operating system(s): Platform independent
- Programming language: Python 3
- Containers: Sequana is available on Bioconda channel [42, 43] and we also provide a Singularity container [44] (version 0.7.0). See http://sequana.readthedocs.org for details.
- License: BSD 3-clause Revised License

## Availability of supporting data and materials

The data sets supporting the results as well as additional files used to created them are available within a Synapse project [27]. More specifically, the BED files mentioned in Section Data Description corresponding to the virus, bacteria and fungus are available under: doi:10.7303/syn10638370.1 (JB409847.filtered.bed), doi:10.7303/syn10638494.1 (JB409847.filtered.bed) and doi:10.7303/syn10638487.1 (S_pombe.filtered.bed), respectively. In addition, we provide the genome reference used in Figure 6 (doi:10.7303/syn10638477.1). The data sets are also available on a Github repository [45] together with a notebook that reproduces the figures. Finally, note that the BED files can be recreated using the original FastQ files available on doi:10.7303/syn10638358. We also provide recipes to create the BED files from the FastQ files as notebooks in [45]. All notebooks mentioned are available in [45].

## Declaration

### List of abbreviations

- BAM: Binary Alignment Map, the binary version of the Sequence Alignment Map (SAM) format.
- BED: Browser Extensible Data
- BOC: Breadth of Coverage
- DOC: Depth of Coverage
- CV: Coefficient of Variation
- EM: Expectation Maximization
- MA: Moving Average
- MLE: Maximum Likelihood Estimate
- RM: Running Median
- ROI: Regions of Interest, samples within a data set identified for a particular purpose.
- SNP: Single Nucleotide Polymorphisms

### Competing Interests

All authors have no conflicts of interest to this manuscript.

### Funding

This work has been supported by France Génomique consortium ANR10-INBS-09-08.

### Author's Contributions

D.D. and T.C. conceived the study. D.D and T.C. implemented the software. C.B. provided the data. D.D. and T.C. contributed to the initial writing. C.B and S.K contributed to the final manuscript. All authors contributed to writing and revision and approved the submission.

## Acknowledgements

We are grateful to Nicolas Escriou (Institut Pasteur) for providing the FastQ and reference of the Virus test case. We are also grateful to Benoit Arcangioli (Institut Pasteur) and Serge Gangloff (Institut Pasteur) for providing the FastQ files and reference of the S. Pombe test case.

## References

1. Goodwin, S., et al. (2016) Coming of age: ten years of next-generation sequencing technologies. Nature Reviews Genetics, 17(6), 333-351.

2. Wang, Z. et al. (2009) RNA-Seq: a revolutionary tool for transcriptomics. Nature reviews genetics, 10 (1), 57-63.
3. Meyerson, M. et al. (2010) Advances in understanding cancer genomes through second-generation sequencing. Nature Reviews Genetics, 11(10), 685-696.
4. Iorio, F. et al. (2016) A Landscape of Pharmacogenomic Interactions in Cancer. Cell, 166(3), 740-754.
5. Eid, J. et al. (2009) Real-time DNA sequencing from single polymerase molecules. Science 323, (5910) 133-138.
6. Lee, H. et al. (2004) Error correction and assembly complexity of single molecule sequencing reads. BioRxiv, 006395.
7. Eisenstein, M. (2012) Oxford Nanopore announcement sets sequencing sector abuzz Nat. Biotechnology 30(4), 295-296
8. Li, H. (2013) Aligning sequence reads, clone sequences and assembly contigs with BWA-MEM. arXiv preprint arXiv:1303.3997.
9. Bankevich, A. et al. (2012) SPAdes: a New genome assembly algorithm and its applications to single-cell sequencing. J. Comput. Biol. 19(5): 455-477.
10. Lander, E.S. and Waterman, M.S. (1988) Genomic mapping by fingerprinting random clones: a mathematical analysis. Genomics, 2(3), 231-239.
11. Wendl, M.C. and Barbazuk, W.B. (2005) Extension of Lander-Waterman theory for sequencing filtered DNA libraries. BMC Bioinformatics, 6(1):245.
12. Ajay S.S., Parker S.C., Abaan H.O., Fajardo K.V., Margulies E.H. (2011) Accurate and comprehensive sequencing of personal genomes. Genome Res. 21(9):1498-505.
13. Mirebrahim, H. et al. (2015) De novo meta-assembly of ultra-deep sequencing data. Bioinformatics, 31(12), i9-i16.
14. Yoon S., Xuan Z., Makarov V., Ye K., Sebat J. (2009) Sensitive and accurate detection of copy number variants using read depth of coverage. Genome Research 19:1586-1592.
15. Brynildsrud, O., Snipen L.G, Bohlin J. (2015) CNOGpro: detection and quantification of CNVs in prokaryotic whole-genome sequencing data. Bioinformatics, 31(11), 2015, 1708-1715.
16. Abyzov A., Urban A.E., Snyder M., Gerstein M. (2011) CNVnator: An approach to discover, genotype, and charaterize typical and atypical CNVs from family and population genome sequencing. Genome Research 21:974-984.
17. Zhao M, Wang Q., Wang, Q, Jia P., Zhao Z. (2013) Computational tools for copy number variation (CNV) detection using next-generation sequencing data: features and perspectives. BMC Bioinformatics 2013, 14 (Suppl 11):S1.
18. Lindner, M.S. et al. (2013) Analyzing genome coverage profiles with applications to quality control in metagenomics. Bioinformatics, 29(10) 1260-1267.
19. Quinlan, A.R. and Hall, I.M., (2010). BEDTools: a flexible suite of utilities for comparing genomic features. Bioinformatics. 26, 6, pp. 841-842. http://bedtools.readthedocs.io
20. Tong, S.Y. et al. (2015) Genome sequencing defines phylogeny and spread of methicillin-resistant Staphylococcus aureus in a high transmission setting. Genome Res., 25(1), 111-118.
21. Bremer, H. Churchward, G (1977) An examination of the Cooper-Helmstetter theory of DNA replication in bacteria and its underlying assumptions. Journal of Theoretical Biology, 69(4): 645-654.
22. Prescott, D.M. and Kuempel, P.L., (1972) Bidirectional replication of the chromosome in Escherichia coli. Proceedings of the National Academy of Sciences, 69(10): 2842-2845.
23. European Nucleotide Archive (ENA). http://www.ebi.ac.uk/ENA. Accessed 8 Sept 2017.
24. Combredet, C. et al. (2003), A molecularly cloned Schwarz strain of measles virus vaccine induces strong immune responses in macaques and transgenic mice. J. Virol., 77(21): 11546-11554
25. Wood, V. et al., (2002) The genome sequence of Schizosaccharomyces pombe. Nature 415(6874), 871-880.
26. Sages's Synapse platform https://www.synapse.org. Accessed 8 Sept 2017.
27. Supporting materials on Synapse project page (BEDs, FastQs, Genome references and genbanks). http://dx.doi.org/doi:10.7303/syn10638358. Accessed 8 Sept 2017.
28. Percival, D.B. and Walden, A.T. (1993) Spectral analysis for physical applications. Cambridge University Press.
29. Balasubramanian, R. et al. (2005) GEO 600 online detector characterization system. Classical Quant. Grav., 22(23), 4973-4986.
30. McKinney, W. Data Structures for Statistical Computing in Python, Proceedings of the 9th Python in Science Conference, 51-56 (2010).
31. Dempster, A.P. and Laird,N.M., and Rubin,D.B. (1977). Maximum likelihood from incomplete data via the EM algorithm. Journal of the royal statistical society. Series B (methodological) 39(1) 1-38.
32. Cokelaer, T. and Desvillechabrol, D. and Legendre, R. and Cardon, M. (2017) Sequana: a Set of Snakemake NGS pipelines. The Journal of Open Source Software, 2, 16 https://doi.org/10.21105/joss.00352. Accessed 8 Sept 2017.
33. Köster, J., and Rahmann, S. (2012). Snakemake - a scalable bioinformatics workflow engine. Bioinformatics, 28(19), 2520-2522.
34. Cokelaer, T. et al.(2013). BioServices: a common Python package to access biological Web Services programmatically. Bioinformatics, 29(24), 3241-3242.
35. Ewels P., Magnusson M., Lundin S., Käller M (2016) MultiQC: Summarize analysis results for multiple tools and samples in a single report Bioinformatics 32, 19, 3047–3048.
36. Dohm, J.C. and Lottaz, C. and Borodina, T. and Himmelbauer, H. (2008) Substantial biases in ultra-short read data sets from high-throughput DNA sequencing. Nucleic Acids Res. 36(16): e105
37. Mohanty, S.D. (2002). Median based line tracker (MBLT): model independent and transient preserving line removal from interferometric data. Class. Quantum Grav., 19(7): 1513-1519.
38. Jones, E. and Oliphant, T. and Peterson, P. et al. (2001) SciPy: Open source scientific tools for Python.
39. Mokry, M. et al (2010) Accurate SNP and mutation detection by targeted custom microarray-based genomic enrichment of short-fragment sequencing libraries. Nucleic Acids Res. 38(10) e116
40. Desvillechabrol D., Legendre R., Rioualen, C., Bouchier C., van Helden J., Kennedy Sean, Cokelaer, T. (2017) Sequanix: a dynamic graphical interface for Snakemake workflows. Bioinformatics, 10.1093/bioinformatics/bty034.
41. Sims, D. et al. (2014) Sequencing depth and coverage: key considerations in genomic analyses. Nature Reviews Genetics, 15(2), 121-132.
42. Conda: Package, dependency and environment management for any language. https://conda.io/docs. Accessed 8 Sept 2017.
43. Bioconda is a channel for the conda package manager specializing in bioinformatics software. http://bioconda.github.io/. Accessed 8 Sept 2017.
44. Kurtzer, G.M and Sochat, V. and Bauer, M.W. (2017) Singularity: Scientific containers for mobility of compute. PLoS One. 12(5).
45. The Sequana resources GitHub repository. https://github.com/sequana/resources/coverage. Accessed 23d Feb 2018.


## Appendix

## Running median implementation

The mean is a measure of the central tendency of a population. It is not a robust estimator in the presence of large extraneous outliers in the population. In such a situation, it is preferable to consider a truncated mean or a median estimator. The median is the middle point of a sample set in which half the numbers are above the median and half are below. More formally, let us consider a sample $s[i], i = 1, .., n$ and $S[i]$ the sequence obtained by sorting $s[i]$ in ascending order (ordering of equal elements is not important here). Then, the median is defined as

$$\nu = \text{median}(\{s[1], s[2], .., s[n]\}) = \begin{cases} S\left[\frac{n+1}{2}\right] & n \text{ odd}, \\ \frac{S[n/2]+S[n/2+1]}{2} & n \text{ even}. \end{cases} \quad (8)$$

Let us now consider a series $X(k)$ where $k = 1, .., N$. Then, the running median of $X(k)$ is defined as the sequence $\nu(k) = \text{median}(\{X(k), X(k+1), .., X(k+W)\}), k = W/2, .., N - W/2$ where $W$ is a window size defined by the user and the application. The first $W/2$ and last $W/2$ values are undefined so we should have $W \ll N$.

Since we perform a sorting of an array of W elements at N positions, the complexity of the running median is N times the complexity of the sorting algorithm. If W and N are small (e.g., removal of narrow lines in power spectral density in addition to the overall smoothing of time or frequency series [29]), a naive quick-sort algorithm ($\mathcal{O}(W^2)$ in the worst case scenario) may be used. However, better algorithms do exist and can be decreased to $\mathcal{O}(\sqrt{W})$ in the worst case as implemented in [37]. Yet, in NGS applications, N could easily reach several millions and W may need to be set to large values up to 50,000 (e.g., to identify long deleted regions).

Instead of computing the median at each position, k, a more efficient solution consists in re-using the sorted block at $k - 1$, and to maintain the block sorted as new elements are added. Indeed, one only needs to insert the next sample into the sorted block and delete the earliest sample from the sorted block. A standard Python module named bisect provides an efficient insertion in sorted data (keeping the data sorted). The complexity of this sorting algorithm is $\mathcal{O}(\log W)$.

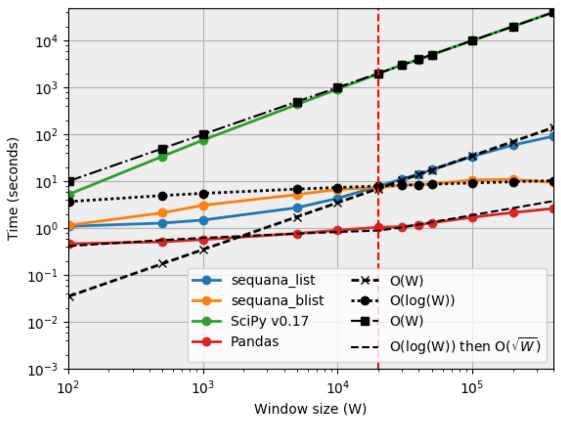


Figure 10. Computational cost of running median algorithms as a function of the window size parameter W (for N = 1e6). Four variants are considered: `SciPy` [38] implementation (function medfilt v0.17), Pandas [30] and 2 Python variants available in `Sequana` based on a `list` or `blist` data containers (see text for details). The `SciPy` variant has a $\mathcal{O}(W)$ complexity irrespective of the W value. For low W values ($W < 20\ 000$), the two Python variants have $\mathcal{O}(\log(W))$ complexity. For larger W values, the blist keeps its $\mathcal{O}(\log(W))$ complexity while the list container follows a $\mathcal{O}(W)$ complexity. Pandas complexity is less clear with a O(W) for $W < 20\ 000$ and $\mathcal{O}(\log(W))$ otherwise. The fastest implementation is clearly the Pandas one even for large W values.

So far, we have neglected the cost of the insertion and deletion steps, which is not negligible. For instance, in Python language, one of the most common data structure is the list. It is a dynamically-sized array (i.e., insertion and deletion of an item from the beginning or middle of the list requires to move most of the list in memory) and the look-up, insertion and deletion have a $\mathcal{O}(n)$ complexity. So the running median is actually dominated by the slow O(n) insertion and deletion steps. A better data structure is available thanks to the blist package; it is based on a so-called B-tree, which is a self-balancing tree data structure that keeps data sorted. The blist allows searches, sequential access, insertions, and deletions in $\mathcal{O}(\log n)$ (see https://pypi.python.org/pypi/blist/ for details).

Based on materials from http://code.activestate.com/recipes/576930/, we have implemented these two variants of running median functions in Python available in Sequana [32] library. We also considered established numerical analysis tools from the `SciPy` [38] and Pandas [30] libraries. We finally compare the four implementations in terms of computation time and complexity as shown in Figure 10. It appears that the Pandas implementation is the fastest. For $W > 20,000$ up to 200,000, our implementation is 2-3 order of magnitude faster than the `SciPy` version but 4-5 times slower than Pandas. We should emphasize the fact that the `SciPy` function has additional features since it is available for N-dimensional data sets whereas we restrict ourselves to 1-D data sets. In `Sequana`, the two variants only differ in the data structure being used to hold the data (list versus `blist`). The Figure 10 shows the difference between the list and blist data structures that is marginal for low W values while for large values asymptotic behaviours are reached showing the interest of the blist over the list choice. We also see that our implementation with blist has a lower complexity than the Pandas implementation. However, for the range considered Pandas is always the fastest choice.

Annotated manuscript differences previous version
Click here to download Personal Cover main_annotated.pdf

GigaScience, 2017, 1–11

doi: xx.xxxx/xxxx
Manuscript in Preparation
Technical Note

TECHNICAL NOTE

# Sequana Coverage: Detection and Characterization of Genomic Variations using Running Median and Mixture Models.

Dimitri Desvillechabrol[1†], Christiane Bouchier[1], Sean Kennedy[1] and Thomas Cokelaer[1,2,†,*]

[1]Institut Pasteur – Pole Biomics – Paris, France and [2]Institut Pasteur – Bioinformatics and Biostatistics Hub – C3BI, USR 3756 IP CNRS – Paris, France

[†]equal contributions
[*]corresponding author
Emails: ddesvillechabrol@gmail.com, christiane.bouchier@pasteur.fr, sean.kennedy@pasteur.fr, thomas.cokelaer@pasteur.fr

## Abstract

Background: In addition to mapping quality information, the Genome coverage contains valuable biological information like the presence of repetitive regions, deleted genes or copy number variations. It is essential to take into consideration atypical regions, trends (e.g., origin of replication) or known and unknown biases that influence coverage. It is also important that reported events have robust statistics (e.g. z-score) associated with their detections as well as precise location.
Results: We provide a standalone application – sequana_coverage – that reports genomic regions of interest (ROIs) which are significantly over- or under-represented in HTS sequencing data. Significance is associated with the events as well as characteristics such as length of the regions. The algorithm first detrends the data using an efficient running median algorithm. It then estimates the distribution of the normalized genome coverage with a Gaussian mixture model. Finally, a z-score statistic is assigned to each base position and used to separate the central distribution from the ROIs (i.e., under- and over-covered regions). A double thresholds mechanism is used to cluster the genomic ROIs. HTML reports provide a summary with interactive visual representations of the genomic ROIs with standard plots and metrics. Genomic variations such as single nucleotide variants (SNVs) or copy number variations (CNVs) can be effectively identified at the same time.

Key words: genome coverage, sequencing depth, running median, Sequana, NGS, Python, Snakemake, CNV

## Background

Sequencing technologies allow researchers to investigate a wide range of genomic questions [1], covering research fields such as the expression of genes (transcriptomics) [2], the discovery of somatic mutations, or the sequencing of complete genomes of cancer samples to name a few examples [3, 4]. The emergence of the second generation sequencing, which is also known as Next-Generation Sequencing or NGS hereafter, has dramatically reduced the sequencing cost. This breakthrough multiplied the number of genomic analyses undertaken by research laboratories but also yielded vast amount of data. Consequently, NGS analysis pipelines require efficient algorithms and scalable visualization tools to process this data and to interpret the results.

Raw data generated by NGS experiments are usually stored in the form of sequencing reads (hereafter simply called reads). A read stores the information about a DNA fragment and also an error probability vector for each base. Read lengths vary from 35-300 bases for current short-read approaches [1] to several tens of thousands of bases possible with long-read technologies such as Pacific Biosciences [5, 6] or Oxford Nanopore [7].

After trimming steps (quality, adapter removal), most high-throughput sequencing (HTS) experiments will require mapping the reads onto a genome of reference [8]. If no reference is available, a de-novo genome assembly can be performed [9]. In both cases, reads can be mapped back on the reference taking into account their quality. We define the genome coverage as the number of reads mapped to a specific position within the reference genome. The theoretical distribution of the genome coverage has been thoroughly studied following the seminal work of Lander-Waterman model [10, 11]. A common metric used to characterize the genome coverage is the sequencing depth: the empirical average of the genome coverage. It may also be called depth of coverage (DOC), fold coverage, read depth, or confusingly, depth or coverage. The sequencing depth unit is denoted X. An example of a genome coverage with a sequencing depth of about 450 X is shown in Figure 1. Another useful metric is the breadth of coverage (BOC): the proportion of the intended genome reference covered by at least one read.

The required sequencing depth depends on the experimental ap-

Key Points

- We propose a novel algorithm to automatically detect genomic regions of interest (e.g., CNV) that depart from the overall genome coverage.
- Normalization is performed with an efficient running median. Using a mixture Gaussian model, we assign to each per-base coverage a z-score. A double threshold clustering is used to report the final list of genomic ROIs.
- We provide a standalone application called sequana_coverage – available in the Sequana [32] project.
- Starting from a BAM or BED file, HTML reports provide the coverage metrics, genomic ROIs, coverage versus GC content plot, genbank annotations, and Javascript visualisation (for viral and bacterial genomes).
- The tool handles multi-chromosomes genomes and provide multiqc [35] reports in such cases.
- Both short (SNVs) and long events (CNVs) are detected by the algorithm with accurate breakpoints reported.
- sequana_coverage was designed for viral and bacterial genomes but can also handle eukaryotes genomes.

plication. For instance, to detect human genome mutations, single-nucleotide polymorphisms (SNPs), and rearrangements, a 30 to 50 X depth is recommended [1, 12] in order to distinguish between sequencing errors and true SNPs. In contrast, the detection of rarely expressed genes in transcriptomics experiments often requires greater sequencing depth. However, greater sequencing depth is not always desirable. Indeed, in addition to a higher cost, ultra-deep sequencing (large sequencing depth in excess of 1000 X) may be an issue for a de-novo genome assembly [13].

The Lander-Waterman model provides a good theoretical estimate of the required sequencing depth to guarantee that all nucleotides are covered at least N times. This is, however, a theoretical estimate that does not take into account technical and biological limitations; some regions being difficult to efficiently map (e.g., repetitive DNA) or containing compositional biases (e.g., GC bias [14]). Furthermore, the genome coverage itself may contain a non-constant trend along the genome due to the impact of replication from the origin of replication. Finally, some regions may be deleted or duplicated. The genome coverage example shown in Figure 1 demonstrates these different features.

While the sequencing depth and other metrics (e.g. BOC) provides a quick understanding about the quality of sequencing and mapping, the genome coverage can also be analysed to identify genomic variations such as single nucleotide variations (SNVs) or copy number variations (CNVs) [15, 16, 17].

In order to detect genomic regions of interests (ROIs) based on genome coverage, a simple and fast approach might be to set two arbitrary thresholds bounding the sequencing depth. However, there are two major drawbacks with this approach. First, as shown in Figure 1 (top panel) and Notebook 4 in [45], with a fixed threshold, one may detect numerous false signals (type I errors) or fail to detect real events (type II errors). An adaptive thresholds that follows the trend of the genome coverage is thus required. Furthermore, a fixed threshold is arbitrary and so the detected events lack a robust means of assigning significance. A more robust alternative is to estimate the genome coverage profile histogram [18] from which a z-score statistics can be used to identify outliers more precisely. Due to a number of known and unknown biases, one should still normalize the data [15]. There are a number of different methods for detecting the ROIs. For example, for CNV detection, numerous techniques are used [17] such as the mean-shift technique [16] or bias correction followed by application of a complex statistical model [15].

In this paper we describe a novel approach that can efficiently detect various types of genomic ROIs. The algorithm does not target any specific type of genomic variations but instead systematically reports all positions (with a z-score) that have depth departing from the overall distribution. The algorithm normalizes the genome coverage using a running median and then calculate a robust statistic (z-score) for each base position based on the parameter estimation of the underlying distribution. This allows us to obtain robust and non-constant thresholds at each genome position. Various types of clustering or filtering can then be implemented to focus on specific categories of variations.

In the Data Description section, we describe the data sets used throughout the paper as test-case examples. In the Methods section, we describe (i) the running median used to detrend the genome coverage, (ii) the statistical methods used to characterize the central distribution from which outliers can be identified and (iii) a double thresholds method proposed to cluster the ROIs. Finally, in the Applications section, we describe the standalone application, sequana_coverage, and potential applications for HTS-dependant research projects including CNVs detection.

## Data Description

Three test-cases of genome coverage are presented here, covering representative organisms and sequencing depths. The genome coverage data sets are in BED (Browser Extensible Data) format, a tabulated file containing the coverage, reference (e.g., chromosome number, contig) and position on the reference. BED files can be created from BAM files (mapped reads) using `bedtools` [19], in particular the `genomecov` tool.

We first considered a bacteria from a study of methicillin resistant Staphylococcus aureus [20]. One circular chromosome of 3 Mbp is present. The sequencing depth is 450 X and the genome coverage exhibits a non-constant trend along the genome (see Figure 1). This pattern, often observed in rapidly growing bacteria, is the result of an unsynchronized population where genome replication occurs bi-directionally from a single origin of replication [21, 22]. The proportion of outliers (see Table 1) is about 2.5 % of the total bases. The original data sets (Illumina sequencing reads, paired-end, 100 bp) are available at the European Nucleotide Archive (ENA) [23] under study accession number PRJEB2076 (ERR036019). The accession number of the reference is FN433596.

The second organism is a virus with a sequencing depth of 1000 X [24]. A circular plasmid, containing the virus chromosome, is 19 795 bp-long. About 13% of the genome coverage contains large or low coverage regions (outliers). It also contains two large under-covered regions (one partially under-covered and one region that is not covered at all) as shown in the Notebook 1 of [45]). The accession number of the reference is JB409847.

The third test case is a fungus (Schizosaccharomyces pombe) [25]. The genome coverage has a sequencing depth of 105 X. It has three non-circular chromosomes of 5.5 Mbp, 4.5 Mbp and 2.5 Mbp. The

Compiled on: March 9, 2018.
Draft manuscript prepared by the author.

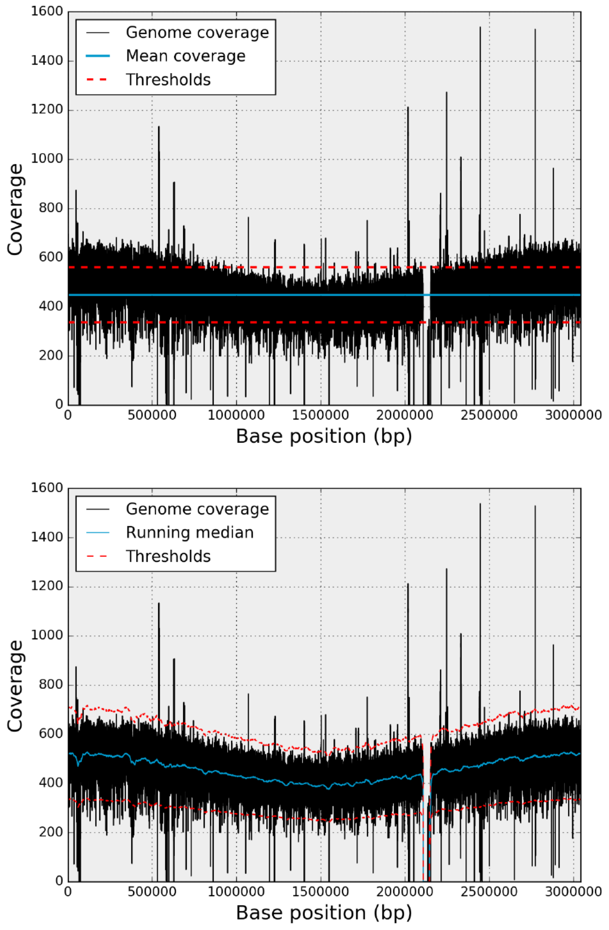


Figure 1. Example of a genome coverage series (in black in both panels). The genome coverage corresponds to the bacteria test case (see text). It contains a deleted region (around 2.2 Mbp) and various under- and over-covered regions (from 100 bp to several Kbp). Although the sequencing depth is about 500 X, there is non-linear trend from 500 X on both ends to 400 X in the middle of the genome. The top panel shows the sequencing depth (blue horizontal line) and two arbitrary fixed thresholds (dashed red lines) at 400 X and 500 X. Due to the non-linear trend, the fixed thresholds lead to an increase of Type I and Type II errors. On the contrary, in the bottom figure, the trend is estimated using a running median (red line) and adaptive lower and upper thresholds (dashed red lines) can be derived.

references from ENA are CU329670.1, CU329671.1 and CU329672.1. Although we will look at the first chromosome only (1.5% of outliers), the tools presented hereafter handles circular chromosomes and multiple chromosomes. See examples in Notebook 3 of [45].

We provide the 3 genome coverage data files in BED format on Synapse [26, 27]. See Section Availability of supporting data and materials for more details.

In addition to these three single-sample cases, we also use a population composed of 6 Staphylococcus aureus isolates from [15] (supplementary), which is used to measure the efficiency of our algorithm against two dedicated CNVs detection tools: CNOGpro [15] and CNVnator [16].

## Methods

### Detrending the genome coverage

The genome coverage function is denoted $C(b)$ where $b$ is the base (nucleotide) position on the genome of reference. The genome coverage and reference lengths are denoted $N$. For simplicity, we drop the parentheses and refer to the genome coverage as $C_b$. The empirical sequencing depth (average of genome coverage) is denoted $\delta = \overline{C_b}$. Ideally, $C_b$ is made of a continuous homogeneous central region. In practice, however, this may be interrupted by a succession of under- and over-covered regions: the genomic ROIs that we want to detect.

A naive classifier consists in setting two fixed thresholds $\delta^-$ and $\delta^+$ whereby low and high ROIs are defined as $C_b^- = C_b \leq \delta^-$ and $C_b^+ = C_b \geq \delta^+$, respectively. If $C_b^0$ denotes the remaining data such that $\delta^- < C_b^0 < \delta^+$, then the genome coverage can be written as $C_b = \{C_b^0, C_b^+, C_b^-\}$.

The advantage of the fixed-thresholds method is that it is conceptually simple and computationally inexpensive. However, there are two major drawbacks manifest. First, as shown in Figure.1-A, false negatives and false positives will increase as soon as there is a non-constant trend present in the data. It may be a low frequency trend as shown here but high frequency trend are also present (see e.g., Fig ??). Also of importance is that an arbitrary choice of threshold(s) is unsatisfactory from a statistical point of view since we cannot associate any level of significance to a genomic region.

In order to account for a possible trend in the genome coverage series (and remove it), a standard method consists in dividing the series by a representative alternative such as its moving average or running median.

The moving average (MA) is computed at each position, b, as the average of W data points around that position and defined as follows:

$$MA_W(b) = \frac{1}{W} \sum_{i=-V}^{V} C(b+i), \tag{1}$$

where W is the length of the moving window (odd number) and $V = (W-1)/2$. Note that the first and last V values are undefined. However, in the case of circular DNA (e.g., viral or bacterial genomes), then the first and last V points are defined since $C_b$ is now a circular series.

Similarly, the running median (RM) is computed at each position, b, as the median of W data points around that position:

$$RM_W(b) = \text{median}(\{C(b-V), .., C(b+V)\}), \tag{2}$$

where W and V are defined as before and the median function is defined as the middle point of the sample set (half of the data is below the median and half is above). A mathematical expression of the median and running median are given in the Appendix section (Eq. 8).

The mean estimator is commonly used to estimate the central tendency of a sample, nevertheless it should be avoided in the presence of extraneous outliers, which are common in NGS genome coverage series (see e.g., Figure 1). Figure 2 shows the impact of outliers when using a moving average or a running mean. We will use the running median only and define the normalized genome coverage as follows:

$$\widetilde{C}_b = \frac{C_b}{RM_W(b)}. \tag{3}$$

We will use the tilde symbol for all metrics associated with the normalized genome coverage, $\widetilde{C}_b$. For instance, $\widetilde{C}_b = \{\widetilde{C}_b^0, \widetilde{C}_b^+, \widetilde{C}_b^-\}$.

The running median is used in various research fields, in particular in spectral analysis [28] to estimate the noise floor while ignoring biases due to narrow frequency bands (e.g., [29]). Here, the goal is to avoid narrow peaks but also to be insensitive to long deleted regions. This can be a major issue in NGS as the running median estimator complexity is a function of the window length. Indeed the running median algorithm involves the sorting of a sample of length W at each position of the genome. So, the running median estimator must be efficient and scalable. This is not an issue in spectral analysis and most fields where running median are used but is a bottleneck

New set of figures replacing
virus genome, which was less clear

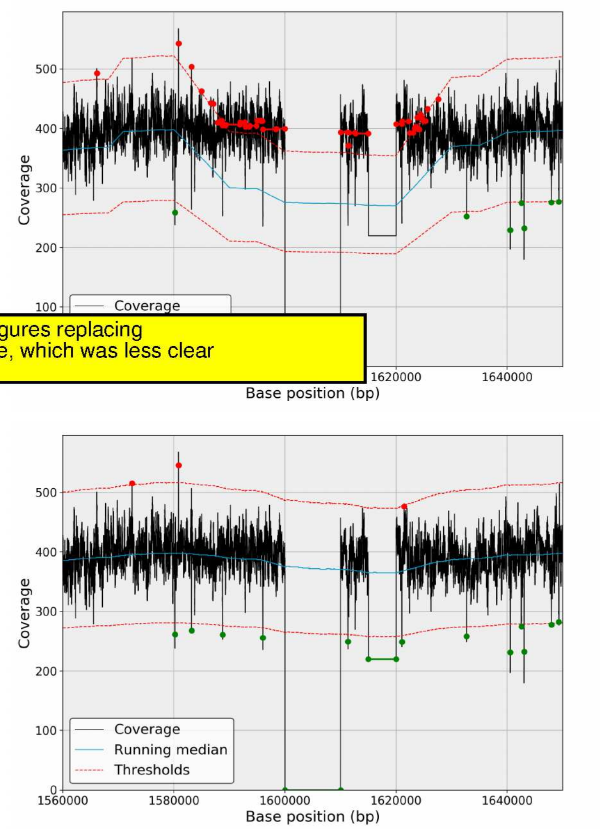


Figure 2. Moving average (top panel) and running median (bottom panel) behaviour in presence of outliers (here, a deleted region in the center followed by a depleted region). In both cases, the window parameter is set to 40,000 bases. The presence of the deleted and depleted regions shows how the trend (blue line) moving average estimation can be shifted as compared to the running median. The thresholds (red lines) are also shifted and the depleted region (position 1,620,000) is not detected. The rate of false detection increases (red dots).

for NGS analysis where W is large. As explained in the Appendix section, the complexity of the sorting part is in $O(n^2)$ in the worst case but similarly to the moving average, one can take advantage of the rolling window and the fact that the previous block is already sorted. We opted for the very efficient Pandas [30] implementation (See Appendix for details). In our implementation, both the moving average and running median have the ability to account for circular DNA data, which is essential to handle circular series.

If we normalize the genome coverage from the bacteria example (Figure 1), we obtain the results shown in Figure 3. Finally, note that the genome coverage being discrete, the running median is also discrete as well as the normalized genome coverage. The discreteness will become more pronounced as sequencing depth decreases.

Hereafter, we will discuss the impact of the W parameter on the detection of genomic ROIs and how to set its value.

### Parameter estimation of the central distribution and adaptive thresholds in the original space

In the ideal case of randomly distributed reads across the genome, the number of reads covering each base follows a Poisson distribution [10]. This distribution is discrete and has one parameter that corresponds to the sequencing depth (mean of the distribution). Yet, the Poisson distribution is often too narrow [18], as can be observed in the three test cases considered. This is generally due to biological over-dispersion. In order to account for over dispersion, the Poisson parameter can be distributed according to a second distribution. For

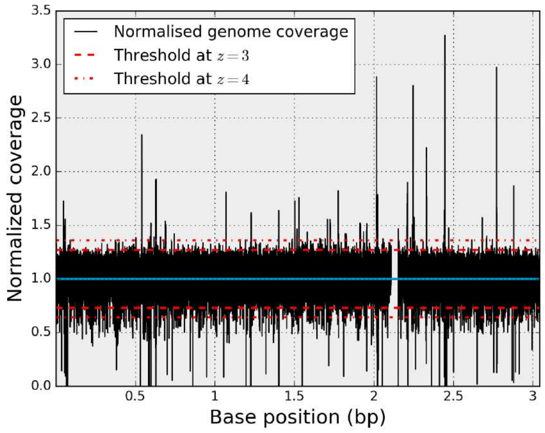


Figure 3. Normalized genome coverage $\widetilde{C}_b$ (bacteria test case). The outliers present in the original genome coverage $C_b$ (see Figure 1) are still present as well as the deleted regions. The distribution is now centred around unity (blue line). Since the distribution is normalized, constant thresholds can be used (dashed lines).

instance when the Poisson parameter is distributed according to a Gamma distribution, we obtain a negative binomial, which has two shape parameters [18].

A Poisson distribution with a large mean parameter approximates a normal distribution, even though, technically, it is not (discrete versus continuous and one parameter versus two). Yet, for $\delta \gg 1$, we can assume that the $C_b$ distribution exhibits a Gaussian distribution denoted $\mathcal{N}(\mu, \sigma^2)$ hereafter where $\mu$ is the average of the genome coverage ($\delta$ in an ideal case) and $\sigma$ is its standard deviation. What about the normalized genome coverage $\widetilde{C}_b$? It is a ratio distribution where the numerator follows $\mathcal{N}(\mu, \sigma^2)$ distribution while the denominator's distribution is that of the running median. We can see empirically that for large $\delta$ and small W parameter, the distribution of the running median follows a Gaussian distribution while for large W or small $\delta$ the running median tends to be discrete and the distribution may depart from a Gaussian distribution (See Notebook 7 of [45]). Even if we knew the running median distribution, the ratio distribution is only known for two Gaussian distributions X and Y (Cauchy distribution) and when (i) the two distributions are centred around zero, which is not the case, and (ii) when they are independent, which is also not the case. Furthermore, the scenario we considered (ideal distribution, $\delta \gg 1$) is too restrictive since we are interested in identifying outliers in real data and may encounter cases where $\delta$ is small (for which $C_b$ follows a negative binomial, not a Gaussian distribution).

Genome coverage is a mix of distributions. Consider for instance the presence of many CNVs, each with a different copy number (either depletion or duplication). The overall distribution here would be very difficult to model analytically. Therefore, the assumtion and our goal is to fit a known distribution on the central distribution so as to establish z-scores on the remaining data.

Our first hypothesis is that $\widetilde{C}_b$ can be decomposed into a central distribution, $\widetilde{C}_b^0$, and a set of outliers, $\widetilde{C}_b^1 = \{\widetilde{C}_b^+, \widetilde{C}_b^-\}$ where the central distribution is predominant: $\left|\widetilde{C}_b^0\right| > \left|\widetilde{C}_b^1\right|$ (vertical bars indicate the cardinality of the sets).

Our second hypothesis is that the mixture model that represents $\widetilde{C}_b$ is a Gaussian mixture model of $k = 2$ models only: $\widetilde{C}_b^0 \sim \mathcal{N}(\widetilde{\mu}_0, \widetilde{\sigma}_0^2)$ and $\widetilde{C}_b^1 \sim \mathcal{N}(\widetilde{\mu}_1, \widetilde{\sigma}_1^2)$. The central distribution $\widetilde{C}_b^0$ exhibits a clear Gaussian distribution both on simulated data (see Notebook 7 in [45]) and on real data (see the three examples in Figure4). The second model is used to identify outliers (below or above the central distribution). The parameters of the second model are not used in defining the central distribution so have little impact on detection.

Similarly to the method deployed in [18] to identify a mixture model of negative binomials (on raw genome coverage), we will use an Expectation Maximization (EM) [31] method to estimate the parameters $\widetilde{\mu}_{0,1}$ and $\widetilde{\sigma}_{0,1}$ (on the normalised genome coverage).

The EM algorithm is an iterative method that alternates between two steps: (i) an Expectation step that creates a function for the expectation of the log-likelihood using the current estimate of the parameters, and (ii) a Minimization step that computes parameters maximizing the expected log-likelihood found in the first step. The likelihood function and the maximum likelihood estimate (MLE) can be derived analytically in the context of Gaussian distributions. Note that in addition to the means and standard deviations, the mixture parameters also need to be estimated. These are denoted $\widetilde{\pi}_0$ and $\widetilde{\pi}_1$. The EM algorithm is standard and can be found in various scientific libraries. Note, however, that the normalized genome coverage may contain zeros in the presence of deleted regions and the estimation of the mixture model should ignore them.

We have applied the EM algorithm on the normalized genome coverage vector on various real NGS data sets including the three test cases in Figure 4. The EM retrieves the parameters of the central distribution (in particular $\tilde{\mu}_0 = 1$) and the outliers. Note that the choice of the running median parameter, W, does not significantly affect the parameter estimation. In each case, the mean of the central distribution is very close to unity. The standard deviation varies significantly and is a function of the sequencing depth only (since the outliers are now incorporated in $C_b^1$). Finally, we can confirm that the proportion of outliers is small as compared to the central distributions by inspection of parameters $\pi_0$ and $\pi_1$: $\widetilde{\pi}_0 >> \widetilde{\pi}_1$.

Once we have identified the parameters of the central distribution $\widetilde{C}_0$, we can assign statistics for $\widetilde{C}_b$ in terms of z-score:

$$z(b) = \frac{\widetilde{C}(b) - \widetilde{\mu}_0}{\widetilde{\sigma}_0}. \quad (4)$$

Since the z-score corresponds to a normal distribution, we can now set a threshold in terms of tolerance interval within which a specified proportion of the genome coverage falls. For instance, with a threshold of 3, we know from the normal distribution that 99.97% of the sample lies in the range -3 and +3. The exact mathematical value is given by the complementary error function, erfc(x), where $x = n/\sqrt{2}$. Note that for $n = 3$, 4 and 5, the tolerance interval is 99.73%, 99.993% and 99.999942%, respectively. Thus, for a genome of 1 Mbp, by pure chance we should obtain about 2700, 70 and 1 outlier(s), respectively.

If we now replace $\widetilde{C}_b$ in Eq.4 using its expression from Eq. 3, we can express the original genome coverage as a function of the running median, the z-score and the parameters of the central distribution:

$$C(b) = \left(\widetilde{\mu}_0 + z(b)\widetilde{\sigma}_0\right) \mathrm{RM}_W(b). \quad (5)$$

We can now set a fixed threshold $z(b) = \pm n$ in the normalized space. This is much easier to manipulate. Moreover, we can derive a variable threshold in the original space that is function of the genome position:

$$\tilde{\delta}^{\pm}(b) = \left(\widetilde{\mu}_0 \pm n^{\pm} \times \widetilde{\sigma}_0\right) \mathrm{RM}_W(b). \quad (6)$$

Examples of variable upper and lower threshold functions are shown in Figure 1 and Figure ?? (red dashed lines). This manipulation results in a robust statistical estimate of the presence of outliers in the genome coverage. The z-score, computed earlier, provides a precise level of confidence.

Using the normalization presented above, we can define the centralness as one minus the proportion of outliers contained in the genome coverage:

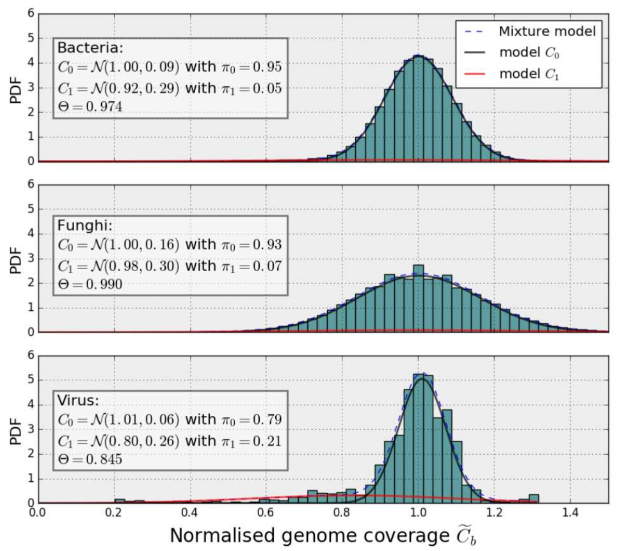


Figure 4. Probability density functions (PDFs) of the normalized genome coverage function concerning the three test cases. The distributions were fitted with a Gaussian mixture models with $k = 2$ models. The first model (black line) fits the central distribution's PDF and the second model (red line close to $y = 0$) fits the outliers' PDF. The dashed lines (close to the black lines) indicates the mixture distribution. In each panel, we report the parameters of the two Gaussian distributions, the proportions $\pi_0$, $\pi_1$ and the $\Theta$ parameter introduced in the text that gives the centralness of the data for each test cases.

$$\Theta_n = 1 - \frac{\left|\widetilde{C}_b^1\right|}{\left|\widetilde{C}_b\right|} = 1 - \frac{\left|\widetilde{C}_b^1\right|}{G}, \quad (7)$$

where G is the length of the genome, and vertical bars indicate the cardinality. This necessarily depends on how the threshold n is set in the normalized space. In the case of an ideal Gaussian distribution and $n = 3$, the centralness should equal the tolerance interval of a normal distribution $\mathcal{N}(0, 1)$ that is the error function, $\mathrm{erf}(n/\sqrt{2})$. The centralness equals unity when there are no outliers i.e., $n \to \infty$. Finally, note that the centralness is meaningless for values below 0.5 (meaning that the central distribution is not central!). As shown in Table 1, $\Theta_3$ equals 0.974, 0.99 and 0.86 in the three cases considered (bacteria, fungus, virus). So the proportion of outliers in the virus case is higher than in the two other test cases, which is not obvious at first glance given the very different lengths of the genome considered.

Finally, it is important to note that the z-scores assigned to each position on the genome coverage are robust with respect to the choice made for the W parameter. As shown in the Notebook 7 of [45], the mean and standard deviation of the distribution of the normalised genome coverage are not affected by the choice of W. Moreover, the parameters of the central distribution estimated with the EM algorithm take into account any effects induced by W.

## Genomic ROIs

From the normalized genome coverage, $\tilde{C}$, and the estimation of its central distribution parameters, a z-score is assigned to each position. All values above the threshold $n^+$ are stored into a subset of events denoted $\widetilde{C}_b^+$ and all values below the threshold $n^-$ are stored into $\widetilde{C}_b^-$. The selected data can be continuous or non-continuous regions. The number of events can be quite large for low thresholds (e.g., for $n^+ = 2.5$, the bacteria has 35 Kbp such events). However, many positions belong to the same event (i.e., same cluster). Considering the short genomic region in Figure 5, which is made of 2000

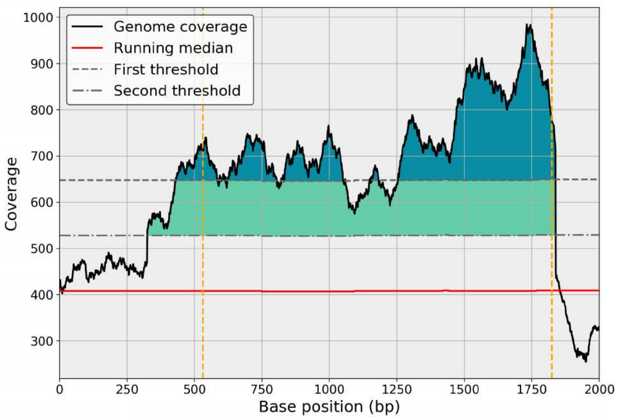


Figure 5. Example of a genomic region of interest (ROI) clustered using a double threshold method. The genome coverage (black line) and its running median (red) on a short genome location of 2 kbp. The first threshold (top dashed gray line) alone identifies many short ROIs (dark blue areas). Using a second threshold (bottom dashed gray line), the short ROIs are clustered and identified as a single ROI (coloured areas). Yellow vertical lines indicates the beginning and end of the cluster.

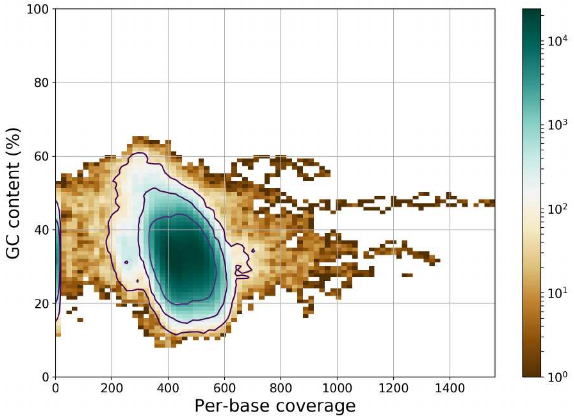


Figure 6. 2-dimensional histogram of the GC content versus coverage available in the HTML reports. The data used correspond to the bacteria test case. We can quickly see that (i) the mean coverage is around 450, (ii) the mean GC is around 30 % (iii) there are part of the genome coverage with zero coverage (left hand side blue line), (iv) there are low and high ROI with coverage up to 1500X that would possibly require more investigations. Be aware of the logarithmic scale: most of the data is indeed centered in the blue area and the brown outliers represent less than a few percentage of the data.

base positions. It contains 5 different regions that cross the threshold $n^+$. Ideally, the 5 events should be clustered together. To do so, we proceed with a double-threshold approach [29] where a second fixed threshold $m^+$ is defined as $m^+ = \alpha^+ n^+$ where $\alpha^+ \leq 1$ and usually set to 1/2.

In the normalized space, the double threshold method works as follows. We scan the entire genome coverage vector starting from the first position $b = 0$. As soon as a per-base coverage value crosses the threshold $m^+$, a new cluster starts. We then accumulate following bases until the per-base coverage crosses $m+$ again (going down). If the maximum of the cluster is above the first threshold, $n^+$, then the cluster is classified as a region of interest. The process carries on until the end of the vector is reached. We repeat this classification for the lower case (with $m^- = \alpha^- n^-$). This method dramatically reduces the number of short ROIs. Finally, we can characterize each region with various metrics such as the length of the region, maximum coverage, mean coverage. If consecutive data points were independent, we could also report a z-score for large events (probability that an event of length N crosses a pre-defined threshold). Instead, for simplicity, we report the mean and max z-score of the event only.

### Impact of the running median parameter

In order to estimate the general trend of the genome coverage, we should avoid the impact of deleted, duplicated or depleted regions. Because the median takes the middle point of a segment, the parameter W should be set to 2N where N is the longest atypical genomic region present in the data. For instance, an expected CNV region with a length of 50,000 would imply setting $W = 100,000$ so that the genome coverage trend remains appropriate (see Notebook 6 in [45] for a counter example). Since such regions are not known in advance, W should be as large as possible so as to avoid the presence of any long regions that depart from the central distribution. Yet, over-increasing W may have undesired effects. For instance, in the extreme case where W is set to the full genome length, one would obtain the same value all along the genome (the sequencing depth itself) and thus lose the interest of the running median by introducing too much smoothing. This could lead to an increase of false detections or missed detections. By default, we recommend to set W to 20,000. Indeed, below this value, it seems that there is a slight increase of marginal false detections while for values in the range $W = 20,000$ to 500,000, the list of ROIs is similar (see Notebook 6 in [45]). As mentioned above, the impact of the W parameter on the z-scores is marginal so one can safely change it from 20,000 to 100,000. A strategy could be to run two analysis: one with $W = 20,000$ to list the short events, and one with very large W for longer events.

| Metric | Bacteria | Fungus | Virus |
|---|---|---|---|
| Genome length | 3 Mbp | 5.5Mbp | 19795 |
| BOC | 0.985 | 1.0 | 0.966 |
| mean $\delta$ | 447.8 | 105.49 | 931.3 |
| median $\delta$ | 453 | 105 | 988 |
| $\sigma$ | 84.1 | 19.9 | 237.2 |
| CV | 0.19 | 0.19 | 0.25 |
| W | 5001 / (20001) | 5001 / (20001) | 5001 |
| $\widetilde{\mu}_0$ | 1.000 / (1.001) | 1.002 / (1.002) | 1.011 |
| $\widetilde{\sigma}_0$ | 0.073 / (0.073) | 0.162 / (0.158) | 0.069 |
| $\Theta_4$ | 0.957 / (0.960) | 0.986 / (0.985) | 0.868 |

Table 1. Metrics derived from the genome coverage of the three test cases considered (Bacteria, Fungus, Virus). The top part of the table contains metrics derived from the genome coverage only, while the bottom part contains metrics derived from the normalized genome coverage, $\widetilde{C}_b$. All metrics are defined in the text; BOC stands for breadth of coverage, $\delta$ for sequencing depth, CV for coefficient of variation. The standard deviation is denoted $\sigma$. In the bacteria and fungus cases, the running window W is set to 5 001 or 20 001 while for the virus we used 5 001 only. The parameters of the central distribution, $\widetilde{\mu}_0$ and $\widetilde{\sigma}_0$ and the centralness, $\Theta_3$ are reported. Proportion of outliers (1-$\Theta_3$) are about 4.5, 1.5 and 13% for the bacteria, fungus and virus, respectively.

## Applications

### Standalone and computational time

Although the algorithm described here above is quite simple per se, each of the three steps required optimization in order to handle HTS data sets. We provide an implementation within the **Sequana** project [32], which is a Python library that also provides HTS pipelines based on the workflow management system called Snake-

make [33] (Makefile-like with a Python syntax). Standalone applications are provided including `sequana_coverage`. In addition to the algorithm described above, the standalone application has several additional features as explained. The input file can be either a BAM or a BED file [19] encoded as a 3-column tab delimited file (chromosome, position, coverage). Consider this command:

```
sequana_coverage --input virus.bed -w 4001 -o
```

The -o option indicates that the input is a circular DNA molecule. The running median window can be tuned using the -w option. Several chromosomes may be present (e.g., fungus case). By default, all chromosomes are analysed but users can select a specific one using the -c option. Other useful options are the ability to change the thresholds on the z-score, ability to cluster close ROIs or to analyse the data by chunks (useful for large eukaryotes genomes). An additional feature is the ability to download a reference genome (given its ENA [23] accession number). This is achieved internally using BioServices [34] which can switch between the ENA or NCBI web services to download the data automatically. Regions of lower genome coverage are sometimes related to repeated content or unusual GC content [36]. Using the reference, we provide a GC content versus coverage plot in the report as shown in Figure 6. Genbank annotations can also be downloaded to annotate ROIs.

The output is a directory that contains, for each molecule: (i) an HTML report, (ii) a summary file (JSON format) and (iii) a CSV file with detected ROIs. In addition, we provide a multiQC report [35] via a plugin available in the Sequana library. The mutliQC report contains a summary of the mapping metrics including the DOC and BOC metrics, the number of ROIs and the centralness (defined in this manuscript). The CSV file is structured with one ROI per row, including information such as the location, length, mean z-score, mean coverage, . . . In the individual HTML reports, `JavaScript` plots are provided together with the ROIs for a quick inspection (not available for large genome >5Mbp).

Finally, the standalone application is designed to be scalable: the virus genome is analysed in a few seconds while the 5 Mbp bacteria genome is analysed in about one minute on a standard computer including analysis and HTML reports (Python implementation). Although the standalone was initially designed for bacterial genomes (genome could fit in memory), we extended the functionality so that larger genomes could also be analysed. In particular, we looked at human genome used in [16]. Although the algorithm is not designed for this lower DOC (around 5X), as the central distribution does not follow a Gaussian distribution, the genome coverage can still be analysed. Thresholds were increased (from 4 to 6) to avoid an abundance of false detections. The 3.5Gb genome could be analysed in a few hours, which is competitive with CNVnator. This required adding an option called binning that merges data before analysis. Similarly to the CNVnator implementation, this reduces the breakpoint accuracy and prevents the tool from identifying short events.

## CNV detection

In extending the functionality of `sequana_coverage` to include larger genomes, we also explored its use in detecting copy number variations (CNVs). CNV detection methods can be categorized into five different strategies depending on the input data: paired-end mapping, split-read, read depth (i.e., genome coverage), de novo assembly and combinations of the above approaches. Amongst the numerous tools based on the genome coverage reported in [17], we considered CNVnator [16], which is able to detect CNVs in various sizes ranging from a few hundred bases to mega-bases. CNVnator can also handle whole genome data sets and exhibits a good precision at detecting breakpoints. We then also consider a more recent tool called CNOGpro [15], which is dedicated to prokaryotic whole genome sequencing data. As stated in [17], none of the various tools have been able to detect the full spectrum of all types of CNVs with high sensitivity and specificity. A combinatorial approach that would take advantage of different methods to increase the performance in detecting CNVs and reduce false positives is preferred.

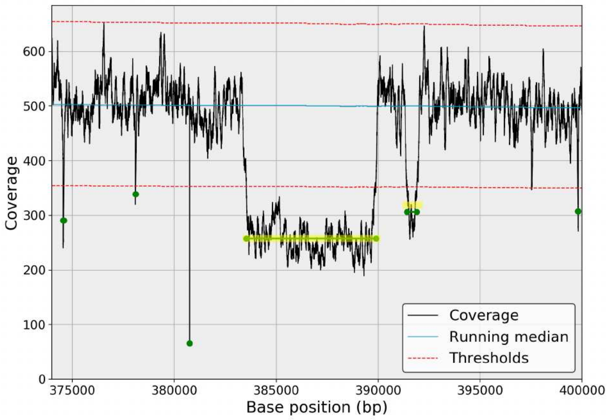


Figure 7. Detection of a depleted region (copy number 0.5). CNVnator (thick yellow segments) and sequana_coverage (thin green segments and green dots) identifies the 6,300 long event with the correct location and similar copy number (based on the mean of the data). sequana_coverage identifies the other depleted region of about 500 bases at position 392,000. CNVnator ability to detect that event depends on the bin parameter: missed for a value of 1 or 100, found with a value of 6. All short events (few bases long) are missed by CNVnator. Conversely, CNVnator is able to identify very long CNV regions up to mega-bases.

We first examined the sensitivity and specificity of sequana_coverage on simulated data. Technical details can be found in the Notebook 5 [45]. Simulated paired-end data were used to create 100X genome coverage data for Staphylococcus aureus. The number of ROIs detected with sequana_coverage varies from one simulation to the other but the main trend is that reported ROIs have short lengths (below 50) and low mean z-scores (below 5); some rare events may have length of 100 bases. There are a small number of false positives (1 or 2 on a 3Mbp genome) when searching for CNV of size above 100 bases. We then injected 3 sets of CNVs: First, we deleted 30 non-overlapping regions (length between 1,000 and 8,000). We achieve a high sensitivity with all deleted regions reported with starting/ending positions accuracy of a few bases. Second, we duplicated 80 non-overlapping regions (same length as above, CN=2). Again, we have a 100% sensitivity with high location accuracy. Sequana_coverage stores a value called log2_ratio for each ROI . This value is the ratio of the mean coverage and mean running median for that ROI and is equivalent of copy number. The average copy number reported for the 80 injected CNVs (W=40,000 bases) is CN = 1.96 ± 0.04 (slightly biased). Third, we injected a mix of 80 depleted and duplicated events (same length as above) at a coverage of 150X (CN=1.5) or 50X (CN=0.5). The 80 events are found again with slightly-reduced accuracy (still below 20 bases). The CN reported for duplicated and deleted events is 1.49 ± 0.023 and 0.5 ± 0.026, respectively. The simulated data indicates that the algorithm can detect short CNVs (from 1000 to 8000) with high sensitivity, accurate estimate of copy number and location.

For a comparison against published tools using real data, we examined the Staphylococcus aureus case used in [16]. We ran sequana_coverage and CNVnator on the 3Mbp genome. CNVnator has a parameter called bin, which is essentially used to define the breakpoint resolution accuracy. We used a bin parameter of 1, 6 and 100 (default) where 6 was chosen as the optimal bin size for the sequencing depth considered (500X). Here, we refered to the instructions found in [16] that led to an empirical equation bin = 2500/DOC (see also Notebook 8 [45]). All results can be found in the Notebook 9 in [45]. The number of events reported by CNVnator are 207, 72 and 13, for bin = 1, 6 and 100 respectively.

With sequana_coverage, W was set to 40,000 bases. The number of reported events is about 600 events (quite stable with respect to W parameter). Only 200 events have a size larger than 10 bases and a mean z-score above 5. All events reported by CNVnator with a bin = 6 or 100 are also detected by sequana_coverage with the same breakpoint resolution. The additional CNVnator events, obtained with bin = 1, are mostly false positives (see Notebook 9 for examples). Visual inspection of events reported by sequana_coverage – but not found by CNVnator – show that they are close to the threshold and appear to be real events (see example in Figure 7). In terms of computational time, sequana_coverage takes one minute on this 3Mbp genome, irrespective of W, while CNVnator takes about 25 minutes, 5 minutes and 40 seconds for the bin = 1, 6 and 100, respectively.

Although CNVnator is more suitable for larger genome sequencing data, we also analysed the viral test case and compared the results with sequana_coverage. This 18 Kb viral genome contains (i) 3 SNVs (coverage of zero) of length 3, 1, and 1 bases; two of them are separated by only 2 bases (ii) two CNV-like events (700 and 800 bases long) and (iii) two short depleted regions with a low signal-to-noise ratio, which are ignored hereafter. Plots are available in the Notebook 10 of [45]. In summary, CNVnator yields different results depending on the bin parameter. With a bin set to 5 (optimal), the two CNV-like events are detected, but not the SNVs ones. Two false positives are called. With a bin parameter of 10 or 20, the two CNV-like events are detected while SNVs are still not detected. Finally, with a bin parameter set to 1, almost the entire genome is classified into 8 different CNVs (2 corrects, 6 false positives) and the SNVs are still missed. With sequana_coverage, irrespective of the window parameter (1000, 2000, 3000, 4000, 5000), the CNVs and SNVs events are correctly detected in addition to the two short depleted regions. Importantly, the running median is not effective for a W parameter which is too small (here 1000). For viral genomes, we would recommend to set W to a quarter of the genome size (here 4000).

We next looked at a comparison using a population of six isolates of Staphylococcus aureus from. The six data sets have a wide range of sequencing depth: 165, 61, 36, 94, 1100, 34, for the isolate ERR043367, ERR043371, ERR073375, ERR043379, ERR14216 and ERR316404, respectively. We compared the results provided in the supplementary data of [15] with those obtained by running sequana_coverage and CNVnator. Amongst the 5466 segments reported in [15], 43 are reported to have a copy number (CN) different from unity. Of the 43 events, 7 are false positives while the remaining are confirmed with sequana_coverage and visual inspection. It is important to note that, unlike CNVnator and sequana_coverage, which rely on the data to find the breakpoint of the ROIs, CNOGpro breakpoints are based on annotation and individual gene (or intergenic segment) assuming that duplications and deletions work at the gene level. Indeed, visual inspection reveals that many of CNOGpro events are sub sets of a larger event. This does not always look appropriate unlike the results provided by CNVnator or sequana_coverage, as shown in Figure 8. For the same reason, several narrow events found in the same intergenic segment will be averaged together whereas sequana_coverage reports the events individually as demonstrated in Figure 9. We also ran CNVnator, with the bin to the optimal value (see above), and for sequana_coverage, we set the W parameter to 40,000. The detected events between CNVnator and sequana_coverage are generally consistent in location and copy number. Both tools have a very good breakpoint accuracy as shown in Figure 8 with the main difference being that sequana_coverage splits events with gap in between (correctly from our point of view). Again, CNVnator is optimised to detect long CNV events and may miss narrower events, even if those event have large variations, as shown in Figure 9.

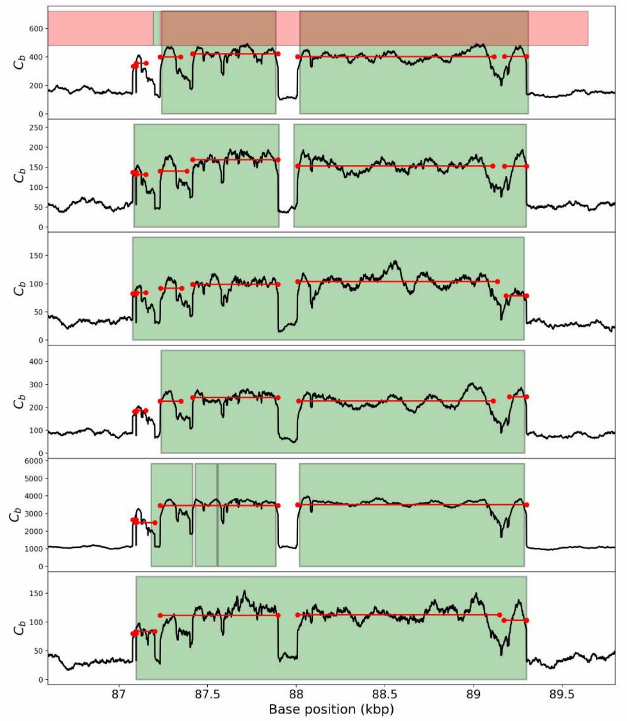


Figure 8. Detection and segmentation of complex events in a population sample. We focus on the region between positions 86,500 and 90,000. We analyse the data (black lines) with sequana_coverage (horizontal colored segments) and CNVnator (green areas). We also report the results of CNOGpro (red areas in the top panel only). CNOGpro detects the complex event as a single event with poor breakpoint resolution (end location is offset by 300 bases); see text for an explanation. CNVnator detects 1 event in 3 isolates, 2 events in 2 isolates) and 4 events in 1 isolate (fifth row); the gap in the middle of the genomic region considered is missed in 50% of the cases; breakpoint resolution is high. sequana_coverage reports 4 to 6 events; the breakpoint resolution is high; the event in the middle is systematically ignored, as it should be given its length of about 100 bases.

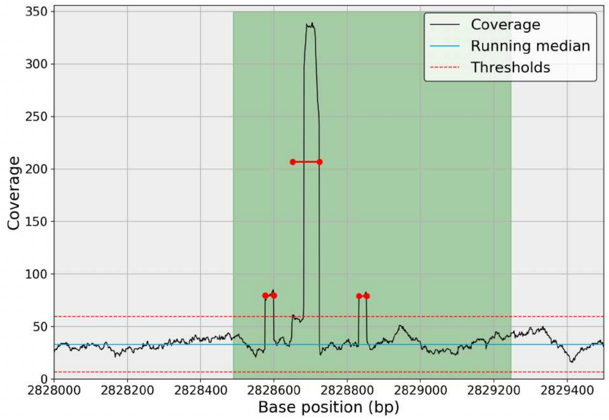


Figure 9. Narrow event made of a strong central peak (copy number CN—10) and two secondary weak peaks (CN—2.5). The 3 peaks can be identified visually in the 6 isolates. In this plot, we only show the isolate ERR316404, which is representative of the 6 others. The algorithm designed in sequana_coverage detects the main peak (CN—5) and the secondary peaks with CN—2.5 (red segments). Note that in the 6 isolates the main peak is detected while the secondary peaks are reported in 66% of the cases (8 peaks out of 12). CNVnator does not detect those events in none of the 6 isolates most probably because the length of those events (irrespective of their strength) are too short. CNOGpro detect 1 event shown here as the green area with a CN—2 for the overall event (although indicating large error with possible value between 1 and 5).

## Conclusion

The method presented in this paper provides a robust statistical framework to detect under and over-covered genomic regions that are then further annotated (length, mean coverage, maximum z-score, ...). Although robust, the method is straighforward and can be summarized in three main steps: (1) detrending of genome coverage series using a running median (ii) parameter estimation of the central distribution of the normalized genome coverage series using an EM approach (for a Gaussian mixture model), (iii) clustering and characterization of the outliers as genomic regions of interest (ROI) using a double threshold clustering method.

We underlined the value of the running median algorithm as compared to a moving average while emphasizing the practical impact of the running median algorithm complexity. An efficient implementation is of paramount importance in the context of HTS analysis. In addition, circular molecules and multi-chromosome organisms are handled.

We implemented the algorithm within the standalone application `sequana_coverage`, which also provides HTML reports with a summary of the genomic ROIs. The HTML reports provide easy visual inspection of genome coverage, list of genomic ROIs and statistics such as the centralness, a metric that encompasses the preponderance of the central distribution with respect to the outliers.

We presented test cases with relatively large sequencing depth (30X to 1000X), although we believe that the algorithm can be for sequencing depths as low as 10X. A natural extension to this work is to consider sequencing depths below 10X by using a mixture model of binomial models instead of gaussian models.

One obvious application of the algorithm presented is the systematic identification of SNVs or CNVs in a single sample or population of samples. We have shown that sequana_coverage is competitive with dedicated tools such as CNOGpro and CNVnator. We believe that sequana_coverage could be used in a combinatorial approach with existing tools to complement and complete the toolkit of CNV detection.

The tool is also relatively fast. Viral and bacterial genomes can be analysed in less than a minute. For larger Eukaryotic genomes (human), once the individual BED files are created for each chromosome, the analysis of the 24 files could be as low as 30 minutes on a standard dual-core. A Snakemake pipeline was also recently implemented in Sequana [32] allowing a human genome to be analysed in less than XX minutes thanks to parallel analysis of the individual chromosomes. A graphical interface using Sequanix [40], a Snakemake GUI is also available, which would make the configuration of the parameters and execution of the analysis on a cluster straightforward.

With additional features such as the ability to annotate the ROIs with genbank files and the identification of repeated regions, we believe that the standalone application `sequana_coverage` will help researchers in deciphering the information contained in the genome coverage. Finally, numerous notebooks, examples and code available in Sequana [32] and [45] should be helpful for integration in other libraries.

## Availability of source code

- Project name: Sequana (sequana_coverage standalone), version 0.7.0
- Project home page: http://sequana.readthedocs.org
- Operating system(s): Platform independent
- Programming language: Python 3
- Containers: Sequana is available on Bioconda channel [42, 43] and we also provide a Singularity container [44] (version 0.7.0). See http://sequana.readthedocs.org for details.
- License: BSD 3-clause Revised License

## Availability of supporting data and materials

The data sets supporting the results as well as additional files used to created them are available within a Synapse project [27]. More specifically, the BED files mentioned in Section Data Description corresponding to the virus, bacteria and fungus are available under: doi:10.7303/syn10638370.1 (JB409847.filtered.bed), doi:10.7303/syn10638494.1 (JB409847.filtered.bed) and doi:10.7303/syn10638487.1 (S_pombe.filtered.bed), respectively. In addition, we provide the genome reference used in Figure 6 (doi:10.7303/syn10638477.1). The data sets are also available on a Github repository [45] together with a notebook that reproduces the figures. Finally, note that the BED files can be recreated using the original FastQ files available on doi:10.7303/syn10638358. We also provide recipes to create the BED files from the FastQ files as notebooks in [45]. All notebooks mentioned are available in [45].

## Declaration

### List of abbreviations

- BAM: Binary Alignment Map, the binary version of the Sequence Alignment Map (SAM) format.
- BED: Browser Extensible Data
- BOC: Breadth of Coverage
- DOC: Depth of Coverage
- CV: Coefficient of Variation
- EM: Expectation Maximization
- MA: Moving Average
- MLE: Maximum Likelihood Estimate
- RM: Running Median
- ROI: Regions of Interest, samples within a data set identified for a particular purpose.
- SNP: Single Nucleotide Polymorphisms

### Competing Interests

All authors have no conflicts of interest to this manuscript.

### Funding

This work has been supported by France Génomique consortium ANR10-INBS-09-08.

### Author's Contributions

D.D. and T.C. conceived the study. D.D and T.C. implemented the software. C.B. provided the data. D.D. and T.C. contributed to the initial writing. C.B and S.K contributed to the final manuscript. All authors contributed to writing and revision and approved the submission.

## Acknowledgements

We are grateful to Nicolas Escriou (Institut Pasteur) for providing the FastQ and reference of the Virus test case. We are also grateful to Benoit Arcangioli (Institut Pasteur) and Serge Gangloff (Institut Pasteur) for providing the FastQ files and reference of the S. Pombe test case.

## References

1. Goodwin, S., et al. (2016) Coming of age: ten years of next-generation sequencing technologies. Nature Reviews Genetics, 17(6), 333-351.

2. Wang, Z. et al. (2009) RNA-Seq: a revolutionary tool for transcriptomics. Nature reviews genetics, 10 (1), 57-63.
3. Meyerson, M. et al. (2010) Advances in understanding cancer genomes through second-generation sequencing. Nature Reviews Genetics, 11(10), 685-696.
4. Iorio, F. et al. (2016) A Landscape of Pharmacogenomic Interactions in Cancer. Cell, 166(3), 740-754.
5. Eid, J. et al. (2009) Real-time DNA sequencing from single polymerase molecules. Science 323, (5910) 133-138.
6. Lee, H. et al. (2004) Error correction and assembly complexity of single molecule sequencing reads. BioRxiv, 006395.
7. Eisenstein, M. (2012) Oxford Nanopore announcement sets sequencing sector abuzz Nat. Biotechnology 30(4), 295-296
8. Li, H. (2013) Aligning sequence reads, clone sequences and assembly contigs with BWA-MEM. arXiv preprint arXiv:1303.3997.
9. Bankevich, A. et al. (2012) SPAdes: a New genome assembly algorithm and its applications to single-cell sequencing. J. Comput. Biol. 19(5): 455-477.
10. Lander, E.S. and Waterman, M.S. (1988) Genomic mapping by fingerprinting random clones: a mathematical analysis. Genomics, 2(3), 231-239.
11. Wendl, M.C. and Barbazuk, W.B. (2005) Extension of Lander-Waterman theory for sequencing filtered DNA libraries. BMC Bioinformatics, 6(1):245.
12. Ajay S.S., Parker S.C., Abaan H.O., Fajardo K.V., Margulies E.H. (2011) Accurate and comprehensive sequencing of personal genomes. Genome Res. 21(9):1498-505.
13. Mirebrahim, H. et al. (2015) De novo meta-assembly of ultra-deep sequencing data. Bioinformatics, 31(12), i9-i16.
14. Yoon S., Xuan Z., Makarov V., Ye K., Sebat J. (2009) Sensitive and accurate detection of copy number variants using read depth of coverage. Genome Research 19:1586-1592.
15. Brynildsrud, O., Snipen L.G, Bohlin J. (2015) CNOGpro: detection and quantification of CNVs in prokaryotic whole-genome sequencing data. Bioinformatics, 31(11), 2015, 1708-1715.
16. Abyzov A., Urban A.E., Snyder M., Gerstein M. (2011) CNVnator: An approach to discover, genotype, and charaterize typical and atypical CNVs from family and population genome sequencing. Genome Research 21:974-984.
17. Zhao M, Wang Q., Wang, Q, Jia P., Zhao Z. (2013) Computational tools for copy number variation (CNV) detection using next-generation sequencing data: features and perspectives. BMC Bioinformatics 2013, 14 (Suppl 11):S1.
18. Lindner, M.S. et al. (2013) Analyzing genome coverage profiles with applications to quality control in metagenomics. Bioinformatics, 29(10) 1260-1267.
19. Quinlan, A.R. and Hall, I.M., (2010). BEDTools: a flexible suite of utilities for comparing genomic features. Bioinformatics. 26, 6, pp. 841-842. http://bedtools.readthedocs.io
20. Tong, S.Y. et al. (2015) Genome sequencing defines phylogeny and spread of methicillin-resistant Staphylococcus aureus in a high transmission setting. Genome Res., 25(1), 111-118.
21. Bremer, H. Churchward, G (1977) An examination of the Cooper-Helmstetter theory of DNA replication in bacteria and its underlying assumptions. Journal of Theoretical Biology, 69(4): 645-654.
22. Prescott, D.M. and Kuempel, P.L., (1972) Bidirectional replication of the chromosome in Escherichia coli. Proceedings of the National Academy of Sciences, 69(10): 2842-2845.
23. European Nucleotide Archive (ENA). http://www.ebi.ac.uk/ENA. Accessed 8 Sept 2017.
24. Combredet, C. et al. (2003), A molecularly cloned Schwarz strain of measles virus vaccine induces strong immune responses in macaques and transgenic mice. J. Virol., 77(21): 11546-11554
25. Wood, V. et al., (2002) The genome sequence of Schizosaccharomyces pombe. Nature 415(6874), 871-880.
26. Sages's Synapse platform https://www.synapse.org. Accessed 8 Sept 2017.
27. Supporting materials on Synapse project page (BEDs, FastQs, Genome references and genbanks). http://dx.doi.org/doi:10.7303/syn10638358. Accessed 8 Sept 2017.
28. Percival, D.B. and Walden, A.T. (1993) Spectral analysis for physical applications. Cambridge University Press.
29. Balasubramanian, R. et al. (2005) GEO 600 online detector characterization system. Classical Quant. Grav., 22(23), 4973-4986.
30. McKinney, W. Data Structures for Statistical Computing in Python, Proceedings of the 9th Python in Science Conference, 51-56 (2010).
31. Dempster, A.P. and Laird,N.M., and Rubin,D.B. (1977). Maximum likelihood from incomplete data via the EM algorithm. Journal of the royal statistical society. Series B (methodological) 39(1) 1-38.
32. Cokelaer, T. and Desvillechabrol, D. and Legendre, R. and Cardon, M. (2017) Sequana: a Set of Snakemake NGS pipelines. The Journal of Open Source Software, 2, 16 https://doi.org/10.21105/joss.00352. Accessed 8 Sept 2017.
33. Köster, J., and Rahmann, S. (2012). Snakemake - a scalable bioinformatics workflow engine. Bioinformatics, 28(19), 2520-2522.
34. Cokelaer, T. et al.(2013). BioServices: a common Python package to access biological Web Services programmatically. Bioinformatics, 29(24), 3241-3242.
35. Ewels P., Magnusson M., Lundin S., Käller M (2016) MultiQC: Summarize analysis results for multiple tools and samples in a single report Bioinformatics 32, 19, 3047–3048.
36. Dohm, J.C. and Lottaz, C. and Borodina, T. and Himmelbauer, H. (2008) Substantial biases in ultra-short read data sets from high-throughput DNA sequencing. Nucleic Acids Res. 36(16): e105
37. Mohanty, S.D. (2002). Median based line tracker (MBLT): model independent and transient preserving line removal from interferometric data. Class. Quantum Grav., 19(7): 1513-1519.
38. Jones, E. and Oliphant, T. and Peterson, P. et al. (2001) SciPy: Open source scientific tools for Python.
39. Mokry, M. et al (2010) Accurate SNP and mutation detection by targeted custom microarray-based genomic enrichment of short-fragment sequencing libraries. Nucleic Acids Res. 38(10) e116
40. Desvillechabrol D., Legendre R., Rioualen, C., Bouchier C., van Helden J., Kennedy Sean, Cokelaer, T. (2017) Sequanix: a dynamic graphical interface for Snakemake workflows. Bioinformatics, 10.1093/bioinformatics/bty034.
41. Sims, D. et al. (2014) Sequencing depth and coverage: key considerations in genomic analyses. Nature Reviews Genetics, 15(2), 121-132.
42. Conda: Package, dependency and environment management for any language. https://conda.io/docs. Accessed 8 Sept 2017.
43. Bioconda is a channel for the conda package manager specializing in bioinformatics software. http://bioconda.github.io/. Accessed 8 Sept 2017.
44. Kurtzer, G.M and Sochat, V. and Bauer, M.W. (2017) Singularity: Scientific containers for mobility of compute. PLoS One. 12(5).
45. The Sequana resources GitHub repository. https://github.com/sequana/resources/coverage. Accessed 23d Feb 2018.


## Appendix

## Running median implementation

The mean is a measure of the central tendency of a population. It is not a robust estimator in the presence of large extraneous outliers in the population. In such a situation, it is preferable to consider a truncated mean or a median estimator. The median is the middle point of a sample set in which half the numbers are above the median and half are below. More formally, let us consider a sample $s[i], i = 1, .., n$ and $S[i]$ the sequence obtained by sorting $s[i]$ in ascending order (ordering of equal elements is not important here). Then, the median is defined as

$$\nu = \text{median}(\{s[1], s[2], .., s[n]\}) = \begin{cases} S\left[\frac{n+1}{2}\right] & n \text{ odd}, \\ \frac{S[n/2]+S[n/2+1]}{2} & n \text{ even}. \end{cases} \quad (8)$$

Let us now consider a series $X(k)$ where $k = 1, .., N$. Then, the running median of $X(k)$ is defined as the sequence $\nu(k) = \text{median}(\{X(k), X(k+1), .., X(k+W)\})$, $k = W/2, .., N - W/2$ where $W$ is a window size defined by the user and the application. The first $W/2$ and last $W/2$ values are undefined so we should have $W \ll N$.

Since we perform a sorting of an array of $W$ elements at $N$ positions, the complexity of the running median is $N$ times the complexity of the sorting algorithm. If $W$ and $N$ are small (e.g., removal of narrow lines in power spectral density in addition to the overall smoothing of time or frequency series [29]), a naive quick-sort algorithm ($\mathcal{O}(W^2)$ in the worst case scenario) may be used. However, better algorithms do exist and can be decreased to $\mathcal{O}(\sqrt{W})$ in the worst case as implemented in [37]. Yet, in NGS applications, $N$ could easily reach several millions and $W$ may need to be set to large values up to 50,000 (e.g., to identify long deleted regions).

Instead of computing the median at each position, $k$, a more efficient solution consists in re-using the sorted block at $k - 1$, and to maintain the block sorted as new elements are added. Indeed, one only needs to insert the next sample into the sorted block and delete the earliest sample from the sorted block. A standard Python module named bisect provides an efficient insertion in sorted data (keeping the data sorted). The complexity of this sorting algorithm is $\mathcal{O}(\log W)$.

So far, we have neglected the cost of the insertion and deletion steps, which is not negligible. For instance, in Python language, one of the most common data structure is the list. It is a dynamically-sized array (i.e., insertion and deletion of an item from the beginning or middle of the list requires to move most of the list in memory) and the look-up, insertion and deletion have a $\mathcal{O}(n)$ complexity. So the running median is actually dominated by the slow $O(n)$ insertion and deletion steps. A better data structure is available thanks to the blist package; it is based on a so-called B-tree, which is a self-balancing tree data structure that keeps data sorted. The blist allows searches, sequential access, insertions, and deletions in $\mathcal{O}(\log n)$ (see https://pypi.python.org/pypi/blist/ for details).

Based on materials from http://code.activestate.com/recipes/576930/, we have implemented these two variants of running median functions in Python available in Sequana [32] library. We also considered established numerical analysis tools from the **SciPy** [38] and Pandas [30] libraries. We finally compare the four implementations in terms of computation time and complexity as shown in Figure 10. It appears that the Pandas implementation is the fastest. For $W > 20,000$ up to 200,000, our implementation is 2-3 order of magnitude faster than the **SciPy** version but 4-5 times slower than Pandas. We should emphasize the fact that the **SciPy** function has additional features since it is available for N-dimensional data sets whereas we restrict ourselves to 1-D data sets. In **Sequana**, the two variants only differ in the data structure being used to hold the data (list versus **blist**). The Figure 10 shows the difference between the list and blist data structures that is marginal for low W values while for large values asymptotic behaviours are reached showing the interest of the blist over the list choice. We also see that our implementation with blist has a lower complexity than the Pandas implementation. However, for the range considered Pandas is always the fastest choice.

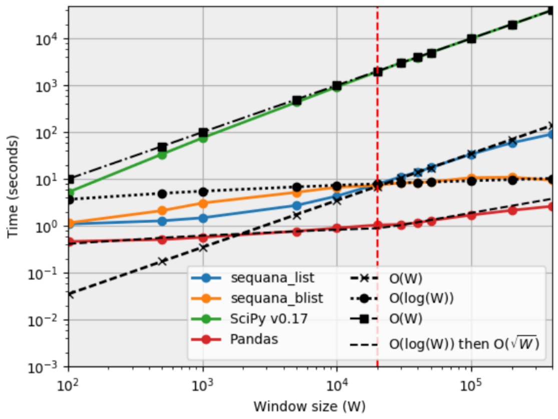


Figure 10. Computational cost of running median algorithms as a function of the window size parameter W (for N = 1e6). Four variants are considered: **SciPy** [38] implementation (function medfilt v0.17), Pandas [30] and 2 Python variants available in **Sequana** based on a **list** or **blist** data containers (see text for details). The **SciPy** variant has a $\mathcal{O}(W)$ complexity irrespective of the W value. For low W values ($W < 20\ 000$), the two Python variants have $\mathcal{O}(\log(W))$ complexity. For larger W values, the blist keeps its $\mathcal{O}(\log(W))$ complexity while the list container follows a $\mathcal{O}(W)$ complexity. Pandas complexity is less clear with a $O(W)$ for $W < 20\ 000$ and $\mathcal{O}(\log(W))$ otherwise. The fastest implementation is clearly the Pandas one even for large W values.
