# Supplementary material for: Sequana coverage: detection and characterization of genomic variations using running median and mixture models
Source: Gigascience. 2018 Sep 6;7(12):giy110. doi: 10.1093/gigascience/giy110 (PMC6275460; doi:10.1093/gigascience/giy110)
Supplement: GIGA-D-17-00238_Revision_2.pdf [file giy110_giga-d-17-00238_revision_2.pdf]

GigaScience

Sequana Coverage: Detection and Characterization of Genomic Variations using Running Median and Mixture Models

--Manuscript Draft--

Manuscript Number:	GIGA-D-17-00238R2
Full Title:	Sequana Coverage: Detection and Characterization of Genomic Variations using Running Median and Mixture Models
Article Type:	Technical Note
Funding Information:	Agence Nationale de la Recherche (ANR10-INBS-09-08) — Mr Dimitri Desvillechabrol
Abstract:	Background: In addition to mapping quality information, Genome coverage contains valuable biological information like the presence of repetitive regions, deleted genes or copy number variations. It is essential to take into consideration atypical regions, trends (e.g., origin of replication) or known and unknown biases that influence coverage. It is also important that reported events have robust statistics (e.g. z-score) associated with their detections as well as precise location<br><br>Results: We provide a standalone application -- sequana_coverage -- that reports genomic regions of interest (ROIs) which are significantly over- or under-represented in HTS sequencing data. Significance is associated with the events as well as characteristics such as length of the regions. The algorithm first detrends the data using an efficient running median algorithm. It then estimates the distribution of the normalized genome coverage with a Gaussian mixture model. Finally, a z-score statistic is assigned to each base position and used to separate the central distribution from the ROIs (i.e., under- and over-covered regions). A double thresholds mechanism is used to cluster the genomic ROIs. HTML reports provide a summary with interactive visual representations of the genomic ROIs with standard plots and metrics. Genomic variations such as single nucleotide variants (SNVs) or copy number variations (CNVs) can be effectively identified at the same time.
Corresponding Author:	Thomas Cokelaer, Ph. D.<br>Institut Pasteur<br>Paris, FRANCE
Corresponding Author Secondary Information:	
Corresponding Author's Institution:	Institut Pasteur
Corresponding Author's Secondary Institution:	
First Author:	Dimitri Desvillechabrol
First Author Secondary Information:	
Order of Authors:	Dimitri Desvillechabrol
	Christiane Bouchier, Ph. D.
	Sean Kennedy, Ph. D.
	Thomas Cokelaer, Ph. D.
Order of Authors Secondary Information:	
Response to Reviewers:	Dear editor,<br><br>The main changes are as followed:<br><br>We created a new sub section called Example: viral genome characterisation before the section CNV detection. In this section, we moved the discussion about the viral genome (previously in the CNV detection section), added a Figure and a Table and

Powered by Editorial Manager® and ProduXion Manager® from Aries Systems Corporation

answered to the first point of the reviewer concerning the false positive (a new image Fig 8 was added to complement the text, one of the reviewer's concern) . Then, in the CNV section, we cleaned up the text with respect to english style by editing the different paragraphs. Finally, we rephrased the last paragraphs of the conclusions; in particular, we clarify the information with respect to the performance of the sequana_coverage tool to analyse the whole human genome (last reviewer's point).

Best regards,

Thomas Cokelaer on behalf of the authors

Reviewer #3:

The authors present a thoroughly updated revision of the manuscript that makes a serious attempt at updating many of my previous points. Following the authors advice, I am now able to install the software on a clean virtual machine through the singularity route. This route would still not present though for users without the necessary privileges to install the singularity software (e.g. institutional HPC), but I guess it is better than many packages. I was able to run the software on the provided example files. In the case of the viral genome I successfully identified, 8 depleted regions and one enriched region (see below).

ANSWER: Given the numbers, we presume that the W parameter was set to 2000 and -o option (circularity) was used. If so, indeed, as shown in the notebook 10, there are 8 depleted regions and one enriched region detected by sequana_coverage. See hereafter for more comments following the next point/question

--

The enriched region is clearly a false positive as its depth is almost exactly at the long term trend for the genome, but is surrounded by two strong depleted regions that are pulling the average down.

ANSWER: Indeed, the enriched region is a false positive caused by the presence of two depleted regions around it. Playing with the W parameter, one can see that for W>4,500 the running median is smoother and that the false positive is not detected anymore. Note that the strength of this False Positive is weak: the mean z-score is around 5.

In the previous version of the manuscript, there was a small discussion in the CNV section concerning the viral case. We have moved and improved that section into a subsection called "Example: viral genome characterisation".

The 8 depleted events (2 long and strong, 3 weak events, 3 strong but few bases long) are all detected by sequana_coverage. This number remains stable for various values of the window parameter.

To give a comparison, we shown that CNVnator detects the two large events, do not show a False positive for the enriched region but misses the 3 weak events as well as the 3 short events. We are aware that CNVnator was designed to detect long CNVs so this result is not surprising.

We have also added in the notebook 10 a comparison using CNOGpro, which was designed for eukaryotic genomes. Only the two large depleted events are detected but with poor precision of the actual length. All short events are missed.

So despite the detection of a False Positive with sequana_coverage, the overall behaviour seems more robust and allows the detection of very short events in addition to the longer ones.

We have added a Figure (8) and a Table (2) to illustrate this point in the new section Example: viral genome characterisation

----

Powered by Editorial Manager® and ProduXion Manager® from Aries Systems Corporation

The largest update to the manuscript concerns an investigation into the use of sequana_coverage for detecting CNVs. This study begins with a simulation experiment. The authors do not report in the main text the number of false positives discovered in simulations with not simulated CNVs saying only "the number of ROIs detected with sequana_coverage varies from one simulation to the other". Examination of the referenced notebook reveals that their representative example has 14 regions.

ANSWER: Indeed in the notebook, a simulated data set with a coverage of 100X for a bacterial genome of about 3Mb led to 14 detected events but is not reported in the manuscript. We have now performed more simulations as explained hereafter.

The authors state that these false positives "have short lengths (below 50) and low mean z-scores (below 5)." All methods produce false positive; using a threshold of 4, one would expect to find around 200 bases beyond the threshold if each base was independent (which it clearly isn't). Does this mean that ROIs shorter than 50bp and with z-scores below 5 should be ignored in CNV or other analyses, or are these bases identifying genuine features in the genome (such as unmappable sequence)?

ANSWER: Using simulated data, the number and strength of the detected events tell us the level of False Positives that are expected. Indeed, if the data were purely gaussian and independent, we would expect for such a genome (3Mbp) about 200 bases beyond a threshold of 4. Here we get only about 17 events (we repeated the simulated data 100 times to get a good statistics) probably due to the nature of the mapping process. Those events are False Positives since we have only random reads covering uniformly the genome. By repeating the simulation 100 times (compare to only one simulation in the previous manuscript), we could get a better idea of the distribution of the False Positives. More importantly we can confirm that the detected events are not genuine features in the genome (e.g. due to GC biases, repeats, etc). Indeed, if this was the case, from one simulation to the other we would possibly detect events at the same location (where there is a genuine feature) and this does not happen across the 100 independent simulated data sets: an event of 50 bases is not seen at the same location from one simulation to the other.

In the simulated data, none of the 1800 ROIs have a length above 80 and a mean z-score (absolute value) above 5. So, in our opinion, with this algorithm, short CNVs below 50 or mean z-score below 5 should be considered as noise.

Clearly what might be considered a false positive for one analysis would not be for another, while so me regions are genuinely false. This is a point that could perhaps be made in the discussion.

ANWSER: we have added an image in the manuscript with the distribution of the ROIs on simulated data and include a paragraph in the CNV section to emphasize the fact that events with zscore below 5 and length below 50 should be considered as noise.

The authors then compare their tool to other tools, and find roughly comparable performance, although it is unfortunately that the same level of analysis doesn't seem to have been applied to the sequana_coverage only ROIs as has been applied to the CNVnator and CNOGpro only events. In particular the authors do not mention any false positives in the viral genome. There are several, as I found when I ran the viral genome analysis myself, including one an enrichment that appears to be caused by normal depth sequence sitting between two depleted regions, which could point to the necessity of further W value tuning. The authors briefly touch on performance of the tool on the human genome as requested, although I am somewhat confused as to the

	results. In the paper they mention that the tools runs as quickly as 30 minutes on a dual core machine for the human genome.<br><br>However, in response to another author they say that running on a human bed takes 1 hour on 24 cores, or 20 hours on one core.<br><br>ANSWER: There was probably a confusion or an error in our responses due to executions on different machines. We double checked the number. It takes 1 hour to 1 hour and a half tto analyse the 24 chromosomes (sequentially) on one core. To be conservative, we specify less than 2 hours in the text (on a single core). However, there are 24 chromosomes. So, we implemented a Snakemake pipeline that allows the analysis of the 24 chromosomes in parallel. So, it can actually go down to only 7 minutes when using 24 cores, which is the time required to analyse the longest chromosome. In the conclusion, we have added<br><br>"the analysis of the 24 human chromosome files should take less than 2 hours (1.5 hours on an HPC cluster using only one core and 1 hour on a DELL Latitude with a SSD hard disk using only one core)"<br><br>And<br><br>"The longest chromosome (chr1) with 250Mb is analysed in about 5-6 minutes. A Snakemake pipeline was also recently implemented within sequana (called coverage) allowing each chromosome to be analysed independently. Using 24 cores, we could analyse the 24 chromosomes in about 7-8 minutes, which is basically the time taken to analyse the longest chromosome."<br><br>-----<br><br>In their response the authors repeated refer to leaving things out because of limited space, but I was under the impression that GIGA-science did not impose length limits on articles. In particular the revised manuscript contains a large number of references to notebooks stored on the sequana github. I feel like many of these would make useful figures or supplementary figures for those not conversant with the python. One might also wonder if the notebooks would be better as supplementary files rather than links to a potentially volatile github site.<br><br>ANSWER: We decided to put the notebooks under a "neutral" organization project on github (rather than a username) so that it can be updated in the future. We can provide a snapshot of the notebook within an archive that can be deposited on giga science web site if required.<br><br>---<br><br>Some of the lanuage is less polished than in the original submission.<br><br>ANSWER: We believe we have now checked the new sections more thoroughly<br><br>---<br><br>The authors have address many of my points. While I would appreciate some further discussion, particularly on the nature of the false-positives, I do not think that this should prevent publication.<br><br>ANSWER: We would like to thank again the reviewer for his suggestions and useful comments that made the final manuscript and the sequana_coverage tool even more useful to the community.
**Additional Information:**	
**Question**	**Response**
Are you submitting this manuscript to a special series or article collection?	No

**Experimental design and statistics**<br><br>Full details of the experimental design and statistical methods used should be given in the Methods section, as detailed in our Minimum Standards Reporting Checklist. Information essential to interpreting the data presented should be made available in the figure legends.<br><br>Have you included all the information requested in your manuscript?	Yes
**Resources**<br><br>A description of all resources used, including antibodies, cell lines, animals and software tools, with enough information to allow them to be uniquely identified, should be included in the Methods section. Authors are strongly encouraged to cite Research Resource Identifiers (RRIDs) for antibodies, model organisms and tools, where possible.<br><br>Have you included the information requested as detailed in our Minimum Standards Reporting Checklist?	Yes
**Availability of data and materials**<br><br>All datasets and code on which the conclusions of the paper rely must be either included in your submission or deposited in publicly available repositories (where available and ethically appropriate), referencing such data using a unique identifier in the references and in the "Availability of Data and Materials" section of your manuscript.<br><br>Have you have met the above requirement as detailed in our Minimum Standards Reporting Checklist?	Yes

Manuscript
Click here to access/download;Manuscript;main_v2.pdf
Click here to view linked References

GigaScience, 2017, 1–12

doi: xx.xxxx/xxxx
Manuscript in Preparation
Technical Note

TECHNICAL NOTE

Sequana Coverage: Detection and Characterization of Genomic Variations using Running Median and Mixture Models.

Dimitri Desvillechabrol[1†], Christiane Bouchier[1], Sean Kennedy[1] and Thomas Cokelaer[1,2,†,*]

[1]Institut Pasteur – Pole Biomics – Paris, France and [2]Institut Pasteur – Bioinformatics and Biostatistics Hub – C3BI, USR 3756 IP CNRS – Paris, France

[†]equal contributions
[*]corresponding author
Emails: ddesvillechabrol@gmail.com, christiane.bouchier@pasteur.fr, sean.kennedy@pasteur.fr, thomas.cokelaer@pasteur.fr

Abstract

Background: In addition to mapping quality information, the Genome coverage contains valuable biological information like the presence of repetitive regions, deleted genes or copy number variations. It is essential to take into consideration atypical regions, trends (e.g., origin of replication) or known and unknown biases that influence coverage. It is also important that reported events have robust statistics (e.g. z-score) associated with their detections as well as precise location.
Results: We provide a standalone application – sequana_coverage – that reports genomic regions of interest (ROIs) which are significantly over- or under-represented in HTS sequencing data. Significance is associated with the events as well as characteristics such as length of the regions. The algorithm first detrends the data using an efficient running median algorithm. It then estimates the distribution of the normalized genome coverage with a Gaussian mixture model. Finally, a z-score statistic is assigned to each base position and used to separate the central distribution from the ROIs (i.e., under- and over-covered regions). A double thresholds mechanism is used to cluster the genomic ROIs. HTML reports provide a summary with interactive visual representations of the genomic ROIs with standard plots and metrics. Genomic variations such as single nucleotide variants (SNVs) or copy number variations (CNVs) can be effectively identified at the same time.

Key words: genome coverage, sequencing depth, running median, Sequana, NGS, Python, Snakemake, CNV

Background

Sequencing technologies allow researchers to investigate a wide range of genomic questions [1], covering research fields such as the expression of genes (transcriptomics) [2], the discovery of somatic mutations, or the sequencing of complete genomes of cancer samples to name a few examples [3, 4]. The emergence of the second generation sequencing, which is also known as Next-Generation Sequencing or NGS hereafter, has dramatically reduced the sequencing cost. This breakthrough multiplied the number of genomic analyses undertaken by research laboratories but also yielded vast amount of data. Consequently, NGS analysis pipelines require efficient algorithms and scalable visualization tools to process this data and to interpret the results.

Raw data generated by NGS experiments are usually stored in the form of sequencing reads (hereafter simply called reads). A read stores the information about a DNA fragment and also an error probability vector for each base. Read lengths vary from 35-300 bases for current short-read approaches [1] to several tens of thousands of bases possible with long-read technologies such as Pacific Biosciences [5, 6] or Oxford Nanopore [7].

After trimming steps (quality, adapter removal), most high-throughput sequencing (HTS) experiments will require mapping the reads onto a genome of reference [8]. If no reference is available, a de-novo genome assembly can be performed [9]. In both cases, reads can be mapped back on the reference taking into account their quality. We define the genome coverage as the number of reads mapped to a specific position within the reference genome. The theoretical distribution of the genome coverage has been thoroughly studied following the seminal work of Lander-Waterman model [10, 11]. A common metric used to characterize the genome coverage is the sequencing depth: the empirical average of the genome coverage. It may also be called depth of coverage (DOC), fold coverage, read depth, or confusingly, depth or coverage. The sequencing depth unit is denoted X. An example of a genome coverage with a sequencing depth of about 450 X is shown in Figure 1. Another useful metric is the breadth of coverage (BOC): the proportion of the intended genome reference covered by at least one read.

The required sequencing depth depends on the experimental ap-

Key Points

- We propose a novel algorithm to automatically detect genomic regions of interest (e.g., CNV) that depart from the overall genome coverage.
- Normalization is performed with an efficient running median. Using a mixture Gaussian model, we assign to each per-base coverage a z-score. A double threshold clustering is used to report the final list of genomic ROIs.
- We provide a standalone application called sequana_coverage – available in the **Sequana** [32] project.
- Starting from a BAM or BED file, HTML reports provide the coverage metrics, genomic ROIs, coverage versus GC content plot, genbank annotations, and Javascript visualisation (for viral and bacterial genomes).
- The tool handles multi-chromosomes genomes and provide multiqc [35] reports in such cases.
- Both short (SNVs) and long events (CNVs) are detected by the algorithm with accurate breakpoints reported.
- Sequana_coverage was designed for viral and bacterial genomes but can also handle eukaryotes genomes.

plication. For instance, to detect human genome mutations, single-nucleotide polymorphisms (SNPs), and rearrangements, a 30 to 50 X depth is recommended [1, 12] in order to distinguish between sequencing errors and true SNPs. In contrast, the detection of rarely expressed genes in transcriptomics experiments often requires greater sequencing depth. However, greater sequencing depth is not always desirable. Indeed, in addition to a higher cost, ultra-deep sequencing (large sequencing depth in excess of 1000 X) may be an issue for a de-novo genome assembly [13].

The Lander-Waterman model provides a good theoretical estimate of the required sequencing depth to guarantee that all nucleotides are covered at least N times. This is, however, a theoretical estimate that does not take into account technical and biological limitations; some regions being difficult to efficiently map (e.g., repetitive DNA) or containing compositional biases (e.g., GC bias [14]). Furthermore, the genome coverage itself may contain a non-constant trend along the genome due to the impact of the origin of replication. Finally, some regions may be deleted or duplicated. The genome coverage example shown in Figure 1 shows these different features.

While the sequencing depth and other metrics (e.g. BOC) provide a quick understanding about the quality of sequencing and mapping, the genome coverage can also be analysed to identify genomic variations such as single nucleotide variations (SNVs) or copy number variations (CNVs) [15, 16, 17].

In order to detect genomic regions of interests (ROIs) based on genome coverage, a simple and fast approach might be to set two arbitrary thresholds bounding the sequencing depth. However, there are two major drawbacks with this approach. First, as shown in Figure 1 (top panel) and Notebook 4 in [45], with a fixed threshold, one may detect numerous false signals (type I errors) or fail to detect real events (type II errors). An adaptive thresholds that follows the trend of the genome coverage is thus required. Furthermore, a fixed threshold is arbitrary and so the detected events lack a robust means of assigning significance. A more robust alternative is to estimate the genome coverage profile histogram [18] from which a z-score statistics can be used to identify outliers more precisely. Due to a number of known and unknown biases, one should still normalize the data [15]. There are a number of different methods for detecting the ROIs. For example, for CNV detection, numerous techniques are used [17] such as the mean-shift technique [16] or bias correction followed by application of a complex statistical model [15].

In this paper we describe a novel approach that can efficiently detect various types of genomic ROIs. The algorithm does not target any specific type of genomic variations but instead systematically reports all positions (with a z-score) that have depth departing from the overall distribution. The algorithm normalizes the genome coverage using a running median and then calculate a robust statistic (z-score) for each base position based on the parameter estimation of the underlying distribution. This allows us to obtain robust and non-constant thresholds at each genome position. Various types of clustering or filtering can then be implemented to focus on specific categories of variations.

In the Data Description section, we describe the data sets used throughout the paper as test-case examples. In the Methods section, we describe (i) the running median used to detrend the genome coverage, (ii) the statistical methods used to characterize the central distribution from which outliers can be identified and (iii) a double thresholds method proposed to cluster the ROIs. Finally, in the Applications section, we describe the standalone application, sequana_coverage, and potential applications for HTS-dependant research projects including CNVs detection.

Data Description

Three test-cases of genome coverage are presented here, covering representative organisms and sequencing depths. The genome coverage data sets are in BED (Browser Extensible Data) format, a tabulated file containing the coverage, reference (e.g., chromosome number, contig) and position on the reference. BED files can be created from BAM files (mapped reads) using `bedtools` [19], in particular the `genomecov` tool.

We first considered a bacteria from a study of methicillin resistant Staphylococcus aureus [20]. One circular chromosome of 3 Mbp is present. The sequencing depth is 450 X and the genome coverage exhibits a non-constant trend along the genome (see Figure 1). This pattern, often observed in rapidly growing bacteria, is the result of an unsynchronized population where genome replication occurs bidirectionally from a single origin of replication [21, 22]. The proportion of outliers (see Table 1) is about 2.5 % of the total bases. The original data sets (Illumina sequencing reads, paired-end, 100 bp) are available at the European Nucleotide Archive (ENA) [23] under study accession number PRJEB2076 (ERR036019). The accession number of the reference is FN433596.

The second organism is a virus with a sequencing depth of 1000 X [24]. A circular plasmid, containing the virus chromosome, is 19 795 bp-long. About 13% of the genome coverage contains large or low coverage regions (outliers). It also contains two large under-covered regions (one partially under-covered and one region that is not covered at all) as shown in the Notebook 1 of [45]). The accession number of the reference is JB409847.

The third test case is a fungus (Schizosaccharomyces pombe) [25]. The genome coverage has a sequencing depth of 105 X. It has three non-circular chromosomes of 5.5 Mbp, 4.5 Mbp and 2.5 Mbp. The

Compiled on: July 30, 2018.
Draft manuscript prepared by the author.

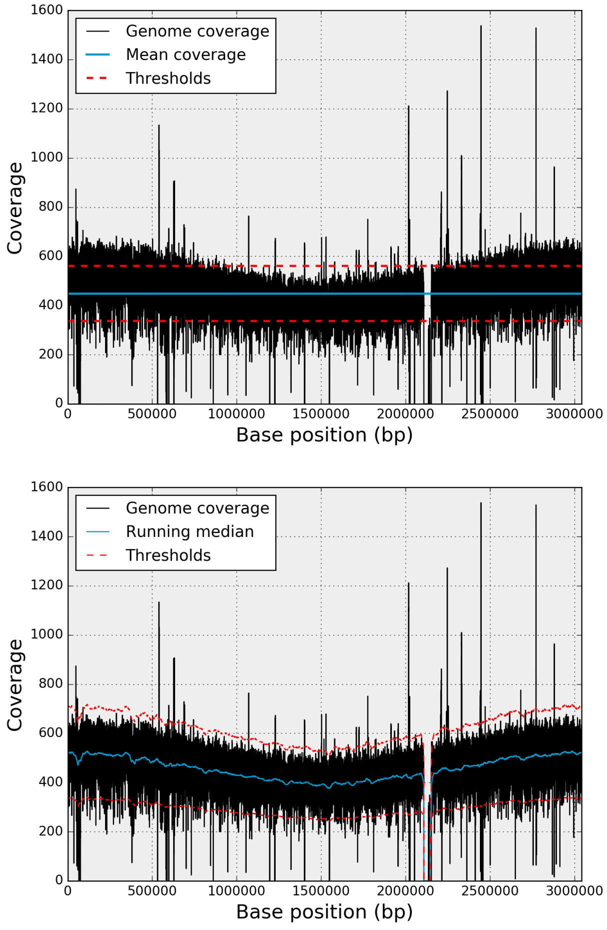

Figure 1. Example of a genome coverage series (in black in both panels). The genome coverage corresponds to the bacteria test case (see text). It contains a deleted region (around 2.2 Mbp) and various under- and over-covered regions (from 100 bp to several Kbp). Although the sequencing depth is about 500 X, there is non-linear trend from 500 X on both ends to 400 X in the middle of the genome. The top panel shows the sequencing depth (blue horizontal line) and two arbitrary fixed thresholds (dashed red lines) at 400 X and 500 X. Due to the non-linear trend, the fixed thresholds lead to an increase of Type I and Type II errors. On the contrary, in the bottom figure, the trend is estimated using a running median (red line) and adaptive lower and upper thresholds (dashed red lines) can be derived.

references from ENA are CU329670.1, CU329671.1 and CU329672.1. Although we will look at the first chromosome only (1.5% of outliers), the tools presented hereafter handles circular chromosomes and multiple chromosomes. See examples in Notebook 3 of [45].

We provide the 3 genome coverage data files in BED format on Synapse [26, 27]. See Section Availability of supporting data and materials for more details.

In addition to these three cases, we also use a population composed of 6 Staphylococcus aureus isolates from [15] (supplementary data), which is used to measure the efficiency of our algorithm against two dedicated CNVs detection tools: CNOGpro [15] and CNVnator [16].

Methods

Detrending the genome coverage

The genome coverage function is denoted C(b) where b is the base (nucleotide) position on the genome of reference. The genome coverage and reference lengths are denoted N. For simplicity, we drop the parentheses and refer to the genome coverage as C_b. The empirical sequencing depth (average of genome coverage) is denoted $\delta = \overline{C_b}$. Ideally, C_b is made of a continuous homogeneous central region. In practice, however, this may be interrupted by a succession of under- and over-covered regions: the genomic ROIs that we want to detect.

A naive classifier consists in setting two fixed thresholds δ^- and δ^+ whereby low and high ROIs are defined as $C_b^- = C_b \leq \delta^-$ and $C_b^+ = C_b \geq \delta^+$, respectively. If C_b^0 denotes the remaining data such that $\delta^- < C_b^0 < \delta^+$, then the genome coverage can be written as $C_b = \{C_b^0, C_b^+, C_b^-\}$.

The advantage of the fixed-thresholds method is that it is conceptually simple and computationally inexpensive. However, there are two major drawbacks manifest. First, as shown in Figure 1-A, false negatives and false positives will increase as soon as there is a non-constant trend present in the data. It may be a low frequency trend as shown here but high frequency trend are also present (see e.g., Figure 2). Also of importance is that an arbitrary choice of threshold(s) is unsatisfactory from a statistical point of view since we cannot associate any level of significance to a genomic region.

In order to account for a possible trend in the genome coverage series (and remove it), a standard method consists in dividing the series by a representative alternative such as its moving average or running median.

The moving average (MA) is computed at each position, b, as the average of W data points around that position and defined as follows:

$$MA_W(b) = \frac{1}{W} \sum_{i=-V}^{V} C(b+i), \qquad (1)$$

where W is the length of the moving window (odd number) and $V = (W-1)/2$. Note that the first and last V values are undefined. However, in the case of circular DNA (e.g., viral or bacterial genomes), then the first and last V points are defined since C_b is now a circular series.

Similarly, the running median (RM) is computed at each position, b, as the median of W data points around that position:

$$RM_W(b) = \text{median}(\{C(b-V), .., C(b+V)\}), \qquad (2)$$

where W and V are defined as before and the median function is defined as the middle point of the sample set (half of the data is below the median and half is above). A mathematical expression of the median and running median are given in the Appendix section (Eq. 8).

The mean estimator is commonly used to estimate the central tendency of a sample, nevertheless it should be avoided in the presence of extraneous outliers, which are common in NGS genome coverage series (see e.g., Figure 1). Figure 2 shows the impact of outliers when using a moving average or a running mean. We will use the running median only and define the normalized genome coverage as follows:

$$\widetilde{C}_b = \frac{C_b}{RM_W(b)}. \qquad (3)$$

We will use the tilde symbol for all metrics associated with the normalized genome coverage, $\widetilde{C}_b$. For instance, $\widetilde{C}_b = \{\widetilde{C}_b^0, \widetilde{C}_b^+, \widetilde{C}_b^-\}$.

The running median is used in various research fields, in particular in spectral analysis [28] to estimate the noise floor while ignoring biases due to narrow frequency bands (e.g., [29]). Here, the goal is to avoid narrow peaks but also to be insensitive to long deleted regions. This can be a major issue in NGS as the running median estimator complexity is a function of the window length. Indeed the running median algorithm involves the sorting of a sample of length W at each position of the genome. So, the running median estimator must be efficient and scalable. This is not an issue in spectral analysis and most fields where running median are used but is a bottleneck

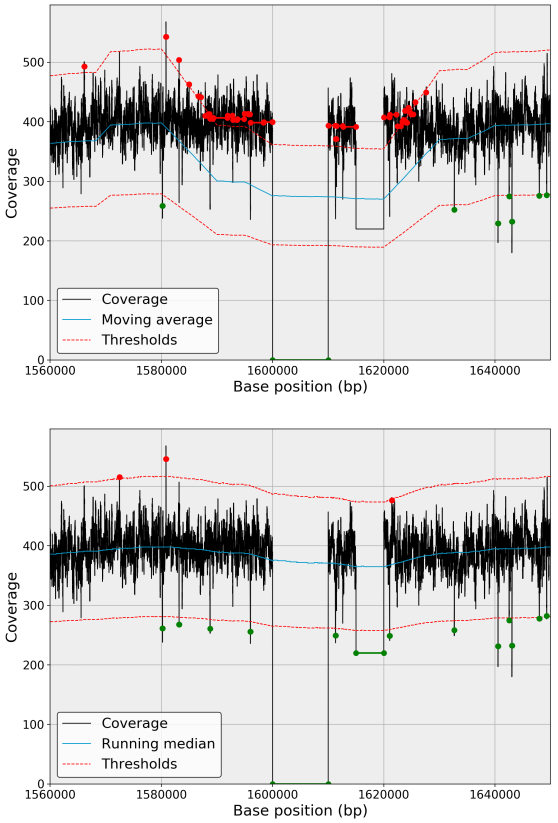

Figure 2. Moving average (top panel) and running median (bottom panel) behaviours in presence of outliers (here, a deleted region in the center followed by a depleted region). In both cases, the window parameter is set to 40,000 bases. The presence of the deleted and depleted regions shows how the moving average (blue line, top panel) can be shifted as compared to the running median (blue line, bottom panel). In the top panel, the thresholds (red lines) are also shifted and consequently the depleted region (position 1,620,000) is not detected. Besides, the rate of false detection increases (red dots). On the contrary, the running median has a better behaviour with less false detections and the ability to detect the depleted region.

for NGS analysis where W is large. As explained in the Appendix section, the complexity of the sorting part is in $O(n^2)$ in the worst case but similarly to the moving average, one can take advantage of the rolling window and the fact that the previous block is already sorted. We opted for the very efficient Pandas [30] implementation (See Appendix for details). In our implementation, both the moving average and running median have the ability to account for circular DNA data, which is essential to handle circular series.

If we normalize the genome coverage from the bacteria example (Figure 1), we obtain the results shown in Figure 3. Finally, note that the genome coverage being discrete, the running median is also discrete as well as the normalized genome coverage. The discreteness will become more pronounced as sequencing depth decreases.

Hereafter, we will discuss the impact of the W parameter on the detection of genomic ROIs and how to set its value.

Parameter estimation of the central distribution and adaptive thresholds in the original space

In the ideal case of randomly distributed reads across the genome, the number of reads covering each base follows a Poisson distribution [10]. This distribution is discrete and has one parameter that corresponds to the sequencing depth (mean of the distribution). Yet, the Poisson distribution is often too narrow [18], as can be observed in the three test cases considered. This is generally due to biological

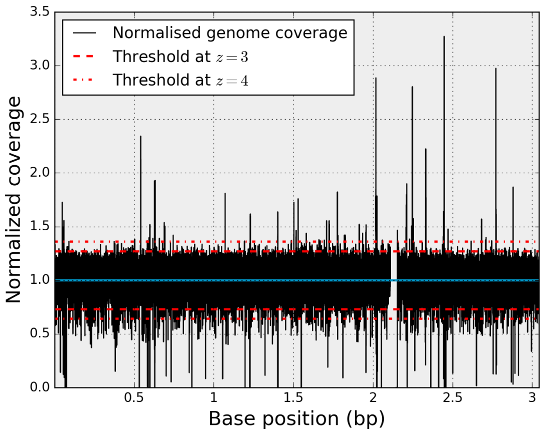

Figure 3. Normalized genome coverage $\widetilde{C}_b$ (bacteria test case). The outliers present in the original genome coverage C_b (see Figure 1) are still present as well as the deleted regions. The distribution is now centred around unity (blue line). Since the distribution is normalized, constant thresholds can be used (dashed lines).

over-dispersion. In order to account for over dispersion, the Poisson parameter can be distributed according to a second distribution. For instance when the Poisson parameter is distributed according to a Gamma distribution, we obtain a negative binomial, which has two shape parameters [18].

A Poisson distribution with a large mean parameter approximates a normal distribution, even though, technically speaking, it is not (discrete versus continuous and one parameter versus two). Yet, for $\delta \gg 1$, we can assume that the C_b distribution exhibits a Gaussian distribution denoted $\mathcal{N}(\mu, \sigma^2)$ hereafter where μ is the average of the genome coverage (δ in an ideal case) and σ is its standard deviation. What about the normalized genome coverage $\widetilde{C}_b$? It is a ratio distribution where the numerator follows $\mathcal{N}(\mu, \sigma^2)$ distribution while the denominator's distribution is that of the running median. We can see empirically that for large δ and small W parameter, the distribution of the running median follows a Gaussian distribution while for large W or small δ the running median tends to be discrete and the distribution may depart from a Gaussian distribution (See Notebook 7 of [45]). Even if we knew the running median distribution, the ratio distribution is only known for two Gaussian distributions X and Y (Cauchy distribution) and when (i) the two distributions are centred around zero, which is not the case, and (ii) when they are independent, which is also not the case. Furthermore, the scenario we considered (ideal distribution, $\delta \gg 1$) is too restrictive since we are interested in identifying outliers in real data and may encounter cases where δ is small (for which C_b follows a negative binomial, not a Gaussian distribution). So, we envisage a solution based on a mixture model as described hereafter.

Genome coverage is a mix of distributions. Consider for instance the presence of many CNVs, each with a different copy number (either depletion or duplication). The overall distribution here would be very difficult to model analytically. Therefore, the assumption and our goal is to fit a known distribution on the central distribution so as to establish z-scores on the remaining data.

Our first hypothesis is that $\widetilde{C}_b$ can be decomposed into a central distribution, $\widetilde{C}_b^0$, and a set of outliers, $\widetilde{C}_b^1 = \{\widetilde{C}_b^+, \widetilde{C}_b^-\}$ where the central distribution is predominant: $\left|\widetilde{C}_b^0\right| > \left|\widetilde{C}_b^1\right|$ (vertical bars indicate the cardinality of the sets).

Our second hypothesis is that the mixture model that represents $\widetilde{C}_b$ is a Gaussian mixture model of $k = 2$ models only: $\widetilde{C}_b^0 \sim \mathcal{N}(\widetilde{\mu}_0, \widetilde{\sigma}_0^2)$ and $\widetilde{C}_b^1 \sim \mathcal{N}(\widetilde{\mu}_1, \widetilde{\sigma}_1^2)$. The central distribution $\widetilde{C}_b^0$ exhibits a clear Gaussian distribution both on simulated data (see Notebook 7 in [45]) and on real data (see the three examples in Figure4). The second model is used to identify outliers (below

or above the central distribution). The parameters of the second model are not used in defining the central distribution so have little impact on detection.

Similarly to the method deployed in [18] to identify a mixture model of negative binomials (on raw genome coverage), we will use an Expectation Maximization (EM) [31] method to estimate the parameters $\widetilde{\mu}_{0,1}$ and $\widetilde{\sigma}_{0,1}$ (on the normalised genome coverage).

The EM algorithm is an iterative method that alternates between two steps: (i) an Expectation step that creates a function for the expectation of the log-likelihood using the current estimate of the parameters, and (ii) a Minimization step that computes parameters maximizing the expected log-likelihood found in the first step. The likelihood function and the maximum likelihood estimate (MLE) can be derived analytically in the context of Gaussian distributions. Note that in addition to the means and standard deviations, the mixture parameters also need to be estimated. These are denoted $\widetilde{\pi}_0$ and $\widetilde{\pi}_1$. The EM algorithm is standard and can be found in various scientific libraries. Note, however, that the normalized genome coverage may contain zeros in the presence of deleted regions and the estimation of the mixture model should ignore them.

We have applied the EM algorithm on the normalized genome coverage vector on various real NGS data sets including the three test cases in Figure 4. The EM retrieves the parameters of the central distribution (in particular $\tilde{\mu}_0 = 1$) and the outliers. Note that the choice of the running median parameter, W, does not significantly affect the parameter estimation. In each case, the mean of the central distribution is very close to unity. The standard deviation varies significantly and is a function of the sequencing depth only (since the outliers are now incorporated in C_b^1). Finally, we can confirm that the proportion of outliers is small as compared to the central distributions by inspection of parameters π_0 and π_1: $\widetilde{\pi}_0 >> \widetilde{\pi}_1$.

Once we have identified the parameters of the central distribution $\widetilde{C}_0$, we can assign statistics for $\widetilde{C}_b$ in terms of z-score:

$$z(b) = \frac{\widetilde{C}(b) - \widetilde{\mu}_0}{\widetilde{\sigma}_0}. \quad (4)$$

Since the z-score corresponds to a normal distribution, we can now set a threshold in terms of tolerance interval within which a specified proportion of the genome coverage falls. For instance, with a threshold of 3, we know from the normal distribution that 99.97% of the sample lies in the range -3 and +3. The exact mathematical value is given by the complementary error function, erfc(x), where $x = n/\sqrt{2}$. Note that for $n = 3$, 4 and 5, the tolerance interval is 99.73%, 99.993% and 99.999942%, respectively. Thus, for a genome of 1 Mbp, by pure chance we should obtain about 2700, 70 and 1 outlier(s), respectively.

If we now replace $\widetilde{C}_b$ in Eq.4 using its expression from Eq. 3, we can express the original genome coverage as a function of the running median, the z-score and the parameters of the central distribution:

$$C(b) = \left(\widetilde{\mu}_0 + z(b)\widetilde{\sigma}_0\right) RM_W(b). \quad (5)$$

We can now set a fixed threshold $z(b) = \pm n$ in the normalized space. This is much easier to manipulate. Moreover, we can derive a variable threshold in the original space that is function of the genome position:

$$\tilde{\delta}^{\pm}(b) = \left(\widetilde{\mu}_0 \pm n^{\pm} \times \widetilde{\sigma}_0\right) RM_W(b). \quad (6)$$

Examples of variable upper and lower threshold functions are shown in Figure 1 and Figure 2 (red dashed lines). This manipulation results in a robust statistical estimate of the presence of outliers in the genome coverage. The z-score, computed earlier, provides a precise level of confidence.

Using the normalization presented above, we can define the centralness as one minus the proportion of outliers contained in the genome coverage:

$$\Theta_n = 1 - \frac{\left|\widetilde{C}_b^1\right|}{\left|\widetilde{C}_b\right|} = 1 - \frac{\left|\widetilde{C}_b^1\right|}{G}, \quad (7)$$

where G is the length of the genome, and vertical bars indicate the cardinality. This necessarily depends on how the threshold n is set in the normalized space. In the case of an ideal Gaussian distribution and $n = 3$, the centralness should equal the tolerance interval of a normal distribution $\mathcal{N}(0, 1)$ that is the error function, $\mathrm{erf}(n/\sqrt{2})$. The centralness equals unity when there are no outliers i.e., $n \to \infty$. Finally, note that the centralness is meaningless for values below 0.5 (meaning that the central distribution is not central!). As shown in Table 1, Θ_3 equals 0.974, 0.99 and 0.86 in the three cases considered (bacteria, fungus, virus). So the proportion of outliers in the virus case is higher than in the two other test cases, which is not obvious at first glance given the very different lengths of the genome considered.

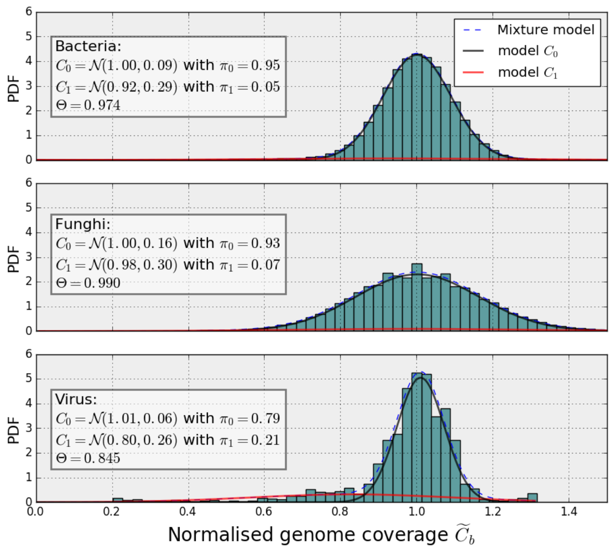

Figure 4. Probability density functions (PDFs) of the normalized genome coverage function concerning the three test cases. The distributions were fitted with a Gaussian mixture models with $k = 2$ models. The first model (black line) fits the central distribution's PDF and the second model (red line close to $y = 0$) fits the outliers' PDF. The dashed lines (close to the black lines) indicates the mixture distribution. In each panel, we report the parameters of the two Gaussian distributions, the proportions π_0, π_1 and the Θ parameter introduced in the text that gives the centralness of the data for each test cases.

Finally, it is important to note that the z-scores assigned to each position on the genome coverage are stable with respect to the W parameter. Indeed, as shown in the Notebook 7 of [45], the mean and standard deviation of the distribution of the normalised genome coverage $\tilde{C}$ are not affected by the parameter W.

Genomic ROIs

Starting from the normalized genome coverage, $\tilde{C}$, we estimate the parameters of the central distribution. This allows us to set a z-score on each genome position. All values above the threshold n^+ are stored into a subset of events denoted $\widetilde{C}_b^+$ and all values below the threshold n^- are stored into $\widetilde{C}_b^-$. The selected data can be made of continuous or non-continuous regions. The number of events can be quite large for low thresholds (e.g., for $n^+ = 2.5$, the bacteria has 35 Kbp such events). However, many positions belong to the same event (i.e., same cluster). Considering the short genomic region in Figure 5, which is made of 2000 base positions. It contains 5 different regions that cross the threshold n^+. Ideally, the 5 events

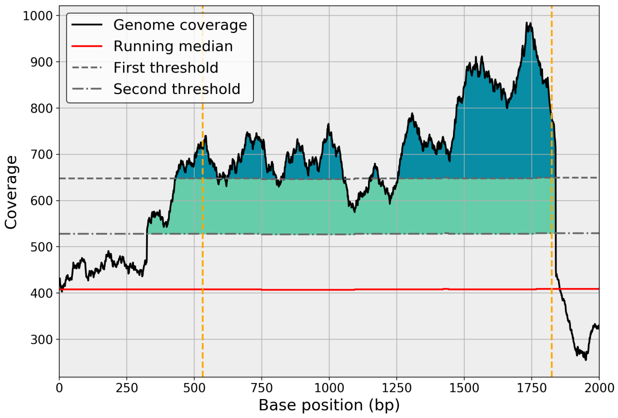

Figure 5. Example of a genomic region of interest (ROI) clustered using a double threshold method. The genome coverage (black line) and its running median (red) on a short genome location of 2 kbp. The first threshold (top dashed gray line) alone identifies many short ROIs (dark blue areas). Using a second threshold (bottom dashed gray line), the short ROIs are clustered and identified as a single ROI (coloured areas). Yellow vertical lines indicates the beginning and end of the cluster.

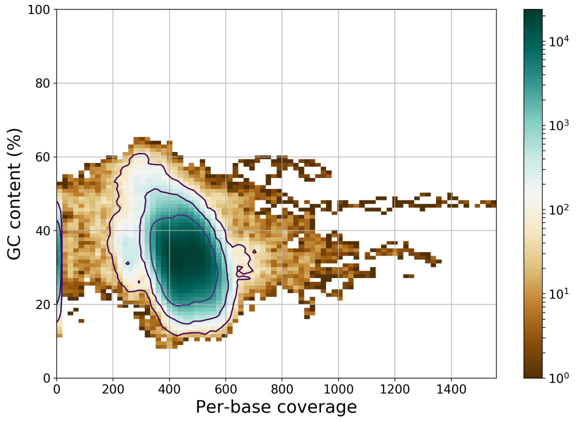

Figure 6. 2-dimensional histogram of the GC content versus coverage available in the HTML reports. The data used correspond to the bacteria test case. We can quickly see that (i) the mean coverage is around 450, (ii) the mean GC is around 30 % (iii) there are part of the genome coverage with zero coverage (left hand side blue line), (iv) there are low and high ROI with coverage up to 1500X that would possibly require more investigations. Be aware of the logarithmic scale: most of the data is indeed centered in the blue area and the brown outliers represent less than a few percentage of the data.

should be clustered together. To do so, we proceed with a double-threshold approach [29] where a second fixed threshold m^+ is defined as $m^+ = \alpha^+ n^+$ where $\alpha^+ \leq 1$ and usually set to 1/2.

In the normalized space, the double threshold method works as follows. We scan the entire genome coverage vector starting from the first position $b = 0$. As soon as a per-base coverage value crosses the threshold m^+, a new cluster starts. We then accumulate following bases until the per-base coverage crosses $m+$ again (going down). If the maximum of the cluster is above the first threshold, n^+, then the cluster is classified as a region of interest. The process carries on until the end of the vector is reached. We repeat this classification for the lower case (with $m^- = \alpha^- n^-$). This method dramatically reduces the number of short ROIs. Finally, we can characterize each region with various metrics such as the length of the region, maximum coverage, mean coverage. If consecutive data points were independent, we could also report a z-score for large events (probability that an event of length N crosses a pre-defined threshold). Instead, for simplicity, we report the mean and max z-score of the event only.

Impact of the running median window parameter

In order to estimate correctly the general trend of the genome coverage, the running median should cancel out the impact of long deleted, duplicated or depleted regions. Because the median takes the middle point of a segment as its estimate, the parameter W should be set to 2N where N is the longest atypical genomic region present in the data. For instance, an expected CNV region with a length of 50,000 would imply setting $W = 100,000$ so that the genome coverage trend remains appropriate (see Notebook 6 in [45] for a counter example). Since such regions are not known in advance, W should be as large as possible so as to avoid the presence of any long regions that depart from the central distribution. Yet, over-increasing W may have undesired effects. For instance, in the extreme case where W is set to the full genome length, one would obtain the same value all along the genome (the sequencing depth itself). This could lead to an increase of false detections or missed detections. By default, we recommend to set W to 20,000. Indeed, below this value, it seems that there is a slight increase of marginal false detections while for values in the range $W = 20,000$ to 500,000, the list of ROIs is similar (see Notebook 6 in [45]). As mentioned above, the impact of the W parameter on the z-scores is marginal so one can safely change it from 20,000 to 100,000. A strategy could be to run two analysis: one with $W = 20,000$ to list the short events, and one with very large W for longer events.

Metric	Bacteria	Fungus	Virus
Genome length	3 Mbp	5.5Mbp	19795
BOC	0.985	1.0	0.966
mean δ	447.8	105.49	931.3
median δ	453	105	988
σ	84.1	19.9	237.2
CV	0.19	0.19	0.25
W	5001 / (20001)	5001 / (20001)	5001
$\widetilde{\mu}_0$	1.000 / (1.001)	1.002 / (1.002)	1.011
$\widetilde{\sigma}_0$	0.073 / (0.073)	0.162 / (0.158)	0.069
Θ_4	0.957 / (0.960)	0.986 / (0.985)	0.868

Table 1. Metrics derived from the genome coverage of the three test cases considered (Bacteria, Fungus, Virus). The top part of the table contains metrics derived from the genome coverage only, while the bottom part contains metrics derived from the normalized genome coverage, $\widetilde{C}_b$. All metrics are defined in the text; BOC stands for breadth of coverage, δ for sequencing depth, CV for coefficient of variation. The standard deviation is denoted σ. In the bacteria and fungus cases, the running window W is set to 5 001 or 20 001 while for the virus we used 5 001 only. The parameters of the central distribution, $\widetilde{\mu}_0$ and $\widetilde{\sigma}_0$ and the centralness, Θ_3 are reported. Proportion of outliers (1-Θ_3) are about 4.5, 1.5 and 13% for the bacteria, fungus and virus, respectively.

Applications

Standalone and computational time

Although the algorithm described here above is quite simple per se, each of the three steps required optimization in order to handle HTS data sets. We provide an implementation within the `Sequana` project [32], which is a Python library that also provides HTS pipelines based on the workflow management system called Snakemake [33] (Makefile-like with a Python syntax). Standalone applications are provided including sequana_coverage. In addition to the algorithm described above, the standalone application has several

additional features as explained hereafter. The input file can be either a BAM or a BED file [19] encoded as a 3-column tab delimited file (chromosome, position, coverage). Consider this command:

```
sequana_coverage --input virus.bed -w 4001 -o
```

The -o option indicates that the input is a circular DNA molecule. The running median window can be tuned using the -w option. Several chromosomes may be present (e.g., fungus case). By default, all chromosomes are analysed but users can select a specific one using the -c option. Other useful options are the ability to change the thresholds on the z-score, ability to cluster close ROIs or to analyse the data by chunks (useful for large eukaryotes genomes). An additional feature is the ability to download a reference genome (given its ENA [23] accession number). This is achieved internally using BioServices [34] that can switch between the ENA or NCBI web services to download the data automatically. Regions of lower genome coverage are sometimes related to repeated content or unusual GC content [36]. Using the reference, we provide a GC content versus coverage plot in the report as shown in Figure 6. Genbank annotations can also be downloaded to annotate ROIs.

The output is a directory that contains (for each contig/chromosome): (i) an HTML report, (ii) a summary file (JSON format) and (iii) a CSV file with detected ROIs. In addition, we provide a multiQC report [35] via a plugin available in the `Sequana` library. The mutliQC report contains a summary of the mapping metrics including the DOC and BOC metrics, the number of ROIs and the centralness (defined in this manuscript). The CSV file is structured with one ROI per row, including information such as the location, length, mean z-score, max z-score, mean coverage . . . In the individual HTML reports, `JavaScript` plots are provided together with the ROIs for a quick inspection (not available for large genome >5 Mbp).

Finally, the standalone application is designed to be scalable: the virus genome is analysed in a few seconds while the 5 Mbp bacteria genome is analysed in about one minute on a standard computer including analysis and HTML reports (Python implementation). Although the standalone was initially designed for bacterial genomes (genome could fit in memory), we extended the functionality so that larger genomes could also be analysed. In particular, we looked at human genome used in [16]. Although the algorithm is not designed for this lower DOC (around 5X), as the central distribution does not follow a Gaussian distribution, the genome coverage can still be analysed. Thresholds were increased (from 4 to 6) to avoid an abundance of false detections. The 3.5Gb genome could be analysed in a couple of hours (see later for details) on a single core. This required adding an option called binning that merges data before analysis. Similarly to the CNVnator implementation, this reduces the breakpoint accuracy and prevents the tool from identifying short events.

Example: viral genome characterisation

In this section we illustrate the usage of sequana_coverage on the viral test case (described in Section Data Description). This 18 kb long genome contains (i) 3 SNVs (coverage of zero) of length 3, 1, and 1 base with two of them separated by only 2 bases (ii) two deleted events (700 and 800 bases long) and (iii) 3 short depleted regions with a low signal-to-noise ratio (see Figure 7 for a visual representation). When running sequana_coverage, the default window parameter is set to 20,000 bases for genome longer than 100,000 bases. Otherwise, the default value of W is set to a fifth of the genome length. Here, it means $W \sim 4,000$. Taking into account the circularity of the genome, we obtain the results shown in Figure 7 and Table 2, where 9 ROIs are found distributed into 8 depleted regions and one enriched region. We emphasize the z-score using the following code color: red, orange and yellow for large, intermediate and small values, respectively. The Table 2 reports the lengths of the ROIs as well as their starting positions. The second ROI (enriched region) can be considered as a false positive but the 8 depleted regions can be considered as true positives. The false positive is due to the presence of two depleted regions that bias the running median estimation and can be avoided by increasing the W parameter. For instance, with $W = 5,000$ the enriched regions is not detected while keeping the 8 depleted regions.

For comparison, we used CNVnator and CNOGpro tools. Although they are dedicated to the search of CNVs, we were expecting to detect at least the long deleted events (ROIs 1 and 3). As summarised in the Table 2, with a bin parameter of 10 or 20, CNVnator detects the two CNV-like events with lengths similar to what is reported by sequana_coverage. No other events were detected (none of the short ones). We got similar results with a bin set to 5 (optimal, as explained hereafter) but there are also 2 short false positives. CNOGpro tool detects the ROI 1 but the ROI 3 is either missed or only partially detected (see Notebook 10 for details). So, despite a marginal false positive, sequana_coverage is able to detect the 8 depleted ROIs with sensible length estimation. The results are also robust with respect to the window parameter W.

Detected ROIs	Sequana			CNVnator		CNOgpro	
	2kb	4kb	5kb	10	20	10	100
1:s=3553, L=699	Y	Y	Y	Y	Y	Y	Y
2:s=4756, L=295	Y	Y	N	N	N	N	N
3:s=5399, L=813	Y	Y	Y	Y	Y	Y	N
4:s=9344, L=217	Y	Y	Y	N	N	N	N
5:s=17045, L=3	Y	Y	Y	N	N	N	N
6:s=17052, L=1	Y	Y	Y	N	N	N	N
7:s=17186, L=339	Y	Y	Y	N	N	N	N
8:s=19257, L=1	Y	Y	Y	N	N	N	N
9:s=19554, L=168	Y	Y	Y	Y	N	N	N

Table 2. List of ROIs detected by sequana_coverage, CNVnator and CNOGpro tools. The sequana columns includes 3 analysis with a window parameter W set to 2, 4 and 5kb. The 4kb column corresponds to the results shown in Figure 7. The CNVnator columns includes 2 analysis with a bin parameter set to 10 and 20. The CNOGpro columns includes 2 analysis with a bin parameter set to 10 and 100. The color code is the same as in Figure 7: red, orange, yellow for significant, intermediate and small z-scores. Note that CNVnator and CNOGpro tools have no false positive results (ROI 2 is not detected). However, none of the ROIs 4 to 9 (short ones) are detected. For each event we also indicate the starting position (s) and length (L) of the events reported by sequana_coverage.

CNV detection

In extending the functionality of sequana_coverage to include larger genomes, we also explored its ability in detecting copy number variations (CNVs).

CNV detection methods can be categorized into five different strategies depending on the input data: paired-end mapping, split-read, read depth (i.e., genome coverage), de novo assembly and combinations of the above approaches. Amongst the numerous tools based on the genome coverage reported in [17], we choose CNVnator [16], which is able to detect CNVs in various sizes ranging from a few hundred bases to mega-bases. CNVnator can also handle whole genome data sets and exhibits a good precision at detecting breakpoints. We also considered a more recent tool called CNOGpro [15], which is dedicated to prokaryotic whole genome sequencing data. As stated in [17], none of the various tools have been able to detect the full spectrum of all types of CNVs with high sensitivity and specificity. To increase the performance in detecting CNVs and reduce false positives, a combinatorial approach could take advantage of the different methods.

We first examined the false positive rate of sequana_coverage on simulated data. Technical details can be found in the Notebook

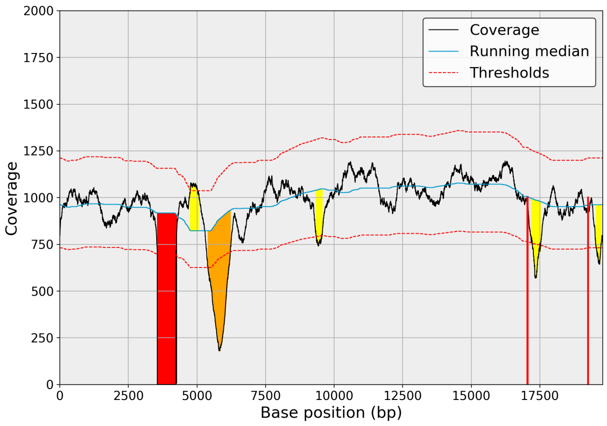

Figure 7. ROIs detected with sequana_coverage. The W window parameter was set to 4,000 bases and circularity was taken into account. We identify 9 events: 8 depleted regions and 1 enriched region. The enriched region could be considered as a false positive that appears like a detection due to the presence of two flanking deleted regions. The color code is as follows: red for max z-score above 12, orange for max z-score between 8 and 12, yellow for max z-score between 4 and 8; grey is for the false positive event. Using a larger window (e.g., 5000), the running median would be smoother between the two long deleted events (on the right hand side), therefore the false positive would not be detected anymore while keeping the 8 depleted regions in the list of ROIs.

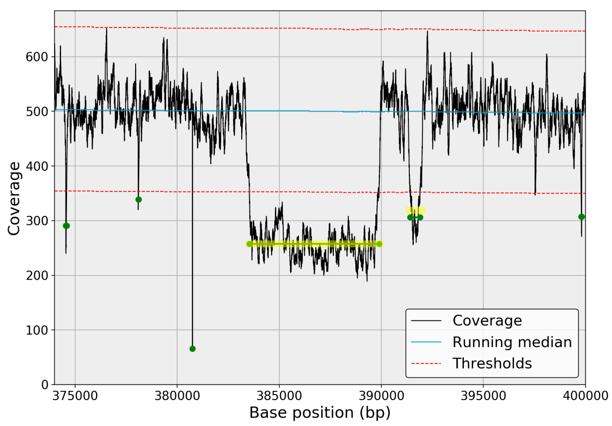

Figure 9. Detection of a depleted region (copy number 0.5). CNVnator (thick yellow segments) and sequana_coverage (thin green segments and dots) identifies the 6,300 long event with the correct location and similar copy number (based on the mean of the data). sequana_coverage identifies the other depleted region of about 500 bases at position 392,000. CNVnator ability to detect that event depends on the bin parameter: missed for a value of 1 or 100, found with a value of 6. All short events (few bases long) are missed by CNVnator. Conversely, CNVnator is able to identify very long CNV regions up to mega-bases.

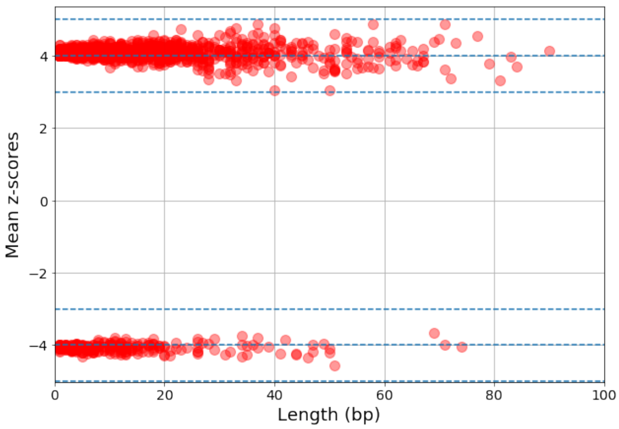

Figure 8. Distribution of ROIs found by analysing 100 simulated genome coverage data from Staphylococcus aureus at a depth of 100X. No features such as CNVs were injected. We plot the mean z-score of each ROIs versus its length (in bases). No events have z-score above 5 or below -5. All ROIs's lengths are below 100 bases. For each simulation, an average of 17 ROIs are found.

5 [45]. Simulated paired-end data were used to create 100 genome coverage data for Staphylococcus aureus, each one having a depth of 100X. The number of ROIs detected with sequana_coverage is 17.5 on average (standard deviation of 6). The 1750 ROIs are plotted in Figure 8, showing their mean z-scores versus lengths. We observed that no z-score are above 5 (in absolute value). However, the size of the ROIs vary widely, up to 80 bases. Such events are not caused by genuine features in the genome (e.g., high GC, repeats). Indeed, across the 100 independent lists of ROIs, the longest events do not appear at the same location on the reference. They are therefore real false positives. Consequently, in the context of CNVs detection, one should ignore events with mean z-score below 5 and length below 100 bases.

We then studied the sensitivity of sequana_coverage by injecting 3 types of CNVs to the simulated data. First, we deleted 30 non-overlapping regions (length between 1,000 and 8,000). We achieved a 100% sensitivity. Indeed all deleted regions were reported with starting and ending position accuracies below 5 bases. Second, we duplicated 80 non-overlapping regions (same length as above), with a copy number CN=2. Again, we have a 100% sensitivity with accuracies below 5 bases. sequana_coverage stores a value called log2_ratio for each ROI. This value corresponds to the ratio of the mean coverage and mean running median for that ROI, and is equivalent to the copy number. The average copy number reported for the 80 injected CNVs is CN $= 1.96 \pm 0.04$. Third, we injected a mix of 80 depleted and duplicated events (same length as above) at a coverage of 150X (CN=1.5) or 50X (CN=0.5). The 80 events are found with a slightly-reduced accuracy (still below 20 bases). The CN reported for duplicated and deleted events is 1.49 ± 0.023 and 0.5 ± 0.026, respectively. The simulated data indicates that the algorithm can detect short CNVs (from 1000 to 8000) with high sensitivity and accurate estimate of copy number and location. If we set the threshold to a mean z-score of 5 and discard events with length below 100 bases, there are no false positive detections.

For a comparison against published tools using real data, we examined the Staphylococcus aureus case used in [16]. We ran sequana_coverage and CNVnator on the 3Mbp genome. CNVnator has a parameter called bin, which is essentially used to define the breakpoint resolution accuracy. We used a bin parameter of 1, 6 and 100 (default) where 6 was chosen as the optimal bin size for the sequencing depth considered (500X). Here, we refered to the instructions found in [16] that led to an empirical equation bin = 2500/DOC (see also Notebook 8 [45]). All results can be found in the Notebook 9 in [45]. The number of events reported by CNVnator are 207, 72 and 13, for bin = 1, 6 and 100 respectively. With sequana_coverage, W was set to 40,000 bases. The number of reported events is about 600 events (quite stable with respect to W parameter). Only 211 events have a size larger than 10 bases and a mean z-score above 5 (47 events have a size larger than 100 bases and a mean z-score above 5). All events reported by CNVnator with a bin = 6 or 100 are also detected by sequana_coverage with the same breakpoint resolution. The additional CNVnator events, obtained with bin = 1, are mostly false positives (see Notebook 9 for examples). Visual inspection of events reported by sequana_coverage – but not found by CNVnator – show that they are close to the threshold and appear to be real events (see example in Figure 9). In terms of computational time, sequana_coverage takes one minute to process this 3Mbp genome, irrespective of W, while CNVnator takes about 25 minutes, 5 minutes and 40 seconds for the bin = 1, 6 and

100, respectively.

Then, we looked at a population of six isolates of Staphylococcus aureus used in [15] . The six data sets have a wide range of sequencing depth: 165, 61, 36, 94, 1100, 34, for the isolate ERR043367, ERR043371, ERR073375, ERR043379, ERR14216 and ERR316404, respectively. We compared the results provided in the supplementary data of [15] with those obtained by running sequana_coverage and CNVnator. In CNOGpro's supplementary, the authors report 43 CNVs with various copy numbers. After visual inspection, we believe that 7 are false positives while the remaining are confirmed by sequana_coverage. It is important to note that, unlike CNVnator and sequana_coverage, which rely on the data to find the breakpoint of the ROIs, CNOGpro breakpoints are based on annotation and individual gene (or intergenic segment) assuming that duplications and deletions work at the gene level. In Figure 10, we show an example of a 2kb-long event present in the 6 isolates. CNOGpro found this event (same gene position) in the 6 isolates, similarly to CNVnator and sequana_coverage. However, the location of the event reported by CNOGpro is not as precise as the two other tools because it is influenced by the a priori knowledge of the gene starting and ending positions. For the same reason, several narrow events found in the same intergenic segment will be averaged together whereas sequana_coverage reports the events individually as demonstrated in Figure 11.

We also ran CNVnator, with its bin parameter set to the optimal value (see above). The detected events found by CNVnator and sequana_coverage (W=40,000) are generally consistent in location and copy number. Both tools have a very good breakpoint accuracy as shown in Figure 10 with the main difference being that sequana_coverage splits events with a gap in between. Again, CNVnator is optimised to detect long CNV events and may miss narrower events, even if those events have large variations, as shown in Figure 11.

Conclusion

The method presented in this paper provides a robust statistical framework to detect under and over-covered genomic regions that are then further annotated (length, mean coverage, maximum z-score, . . .). Although robust, the method is straighforward and can be summarized in three main steps: (1) detrending of genome coverage series using a running median (ii) parameter estimation of the central distribution of the normalized genome coverage series using an EM approach (for a Gaussian mixture model), (iii) clustering and characterization of the outliers as genomic regions of interest (ROI) using a double threshold clustering method.

We underlined the value of the running median algorithm as compared to a moving average while emphasizing the practical impact of the running median algorithm complexity. We used an efficient running median algorithm (see supplementary), which is of paramount importance in the context of HTS analysis. In our implementation, we can take into account the circularity of the molecules as well as multi-chromosome organisms.

We implemented the method described in this paper within the standalone application sequana_coverage, which also provides HTML reports with a summary of the genomic ROIs detected. The HTML reports provide easy visual inspection of genome coverage, list of genomic ROIs and statistics such as the centralness, a metric that encompasses the preponderance of the central distribution with respect to the outliers.

We presented test cases with relatively large sequencing depth (30X to 1000X), although we believe that the algorithm can be used for sequencing depths as low as 10X. A natural extension to this work is to consider sequencing depths below 10X by using a mixture model of binomial models instead of gaussian models.

One obvious application of the algorithm presented is the systematic identification of SNVs or CNVs in a single sample or population

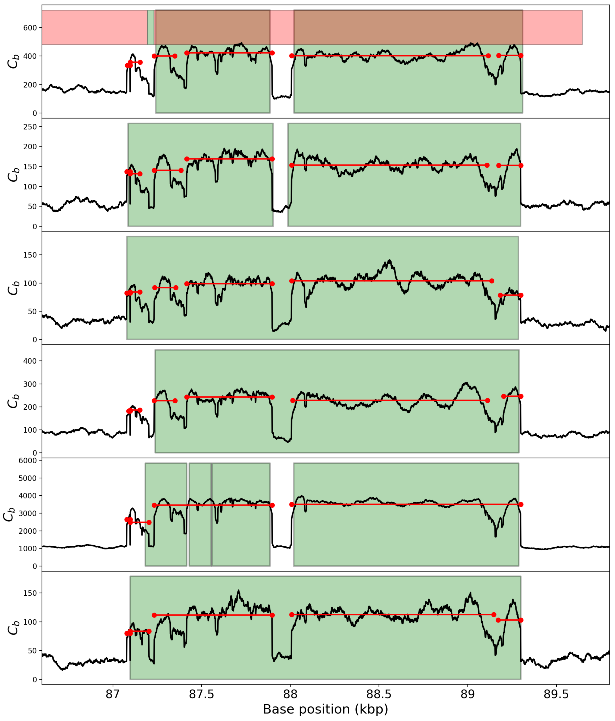

Figure 10. Detection and segmentation of complex events in a population sample. We focus on the region between positions 86,500 and 90,000. We analyse the data (black lines) with sequana_coverage (horizontal red lines) and CNVnator (green areas). We also report the results of CNOGpro (red areas in the top panel only). CNOGpro detects the complex event as a single event with poor breakpoint resolution (end location is offset by 300 bases); see text for an explanation. CNVnator detects 1 event in 3 isolates, 2 events in 2 isolates) and 4 events in 1 isolate (fifth row); the gap in the middle of the genomic region considered is missed in 50% of the cases; breakpoint resolution is high. sequana_coverage reports 4 to 6 events; the breakpoint resolution is high; the event in the middle is systematically ignored, as it should, given its length is about 100 bases.

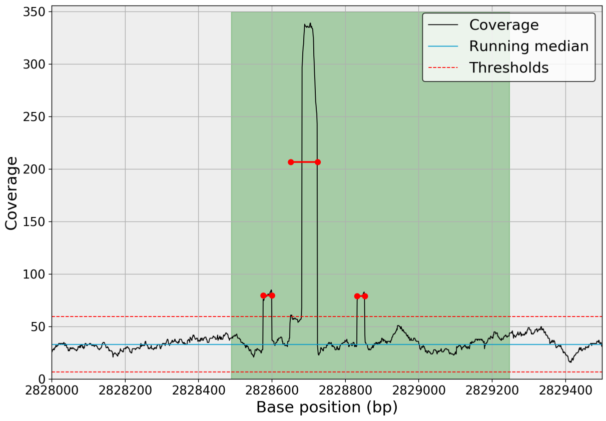

Figure 11. Narrow event made of a strong central peak (copy number CN=10) and two secondary weak peaks (CN=2.5). The 3 peaks can be identified visually in the 6 isolates. In this plot, we only show the isolate ERR316404. The algorithm designed in sequana_coverage detects the main peak (with CN=5) and the secondary peaks with CN=2.5 (red segments). Note that in the 6 isolates the main peak is always detected while the secondary peaks are detected in 66% of the cases (8 peaks out of 12). CNVnator does not detect those events in none of the 6 isolates, most probably because the length of those events (irrespective of their strength) are too short. CNOGpro detect 1 event, shown here as the green area with a CN=2 for the overall event.

of samples. We have shown that sequana_coverage is competitive with dedicated tools such as CNOGpro and CNVnator. We believe that sequana_coverage could be used in a combinatorial approach with existing tools to complement and complete the toolkit of CNV detection.

Sequana_coverage is also relatively fast. Viral and bacterial genomes can be analysed in less than a minute. For larger Eukaryotic genomes (human), once the individual BED files are created for each chromosome, the analysis of the 24 human chromosome files should take less than 2 hours (1.5 hours on an HPC cluster using only one core and 1 hour on a DELL Latitude with a SSD hard disk using only one core). The longest chromosome (chr1), with 250Mb, is analysed in about 5-6 minutes. A Snakemake [33] pipeline was also recently implemented within `Sequana` [32] (named Coverage), allowing each chromosome to be analysed independently. Using 24 cores, we could analyse the 24 chromosomes in about 7-8 minutes, which is basically the time taken to analyse the longest chromosome. A graphical interface using Sequanix [40] (a Snakemake GUI) is also available, making the configuration of the parameters and execution of the analysis on a cluster accessible.

With additional features such as the ability to annotate the ROIs with genbank files and the identification of repeated regions, we believe that the standalone application sequana_coverage will help researchers in deciphering the information contained in the genome coverage.

Availability of source code

- Project name: Sequana (sequana_coverage standalone), version 0.7.0
- Project home page: http://sequana.readthedocs.org
- Operating system(s): Platform independent
- Programming language: Python 3
- Containers: Sequana is available on Bioconda channel [42, 43] and we also provide a Singularity container [44] (version 0.7.0). See http://sequana.readthedocs.org for details.
- License: BSD 3-clause Revised License

Availability of supporting data and materials

The data sets supporting the results as well as additional files used to created them are available within a Synapse project [27]. More specifically, the BED files mentioned in Section Data Description corresponding to the virus, bacteria and fungus are available under: doi:10.7303/syn10638370.1 (JB409847.filtered.bed), doi:10.7303/syn10638494.1 (JB409847.filtered.bed) and doi:10.7303/syn10638487.1 (S_pombe.filtered.bed), respectively. In addition, we provide the genome reference used in Figure 6 (doi:10.7303/syn10638477.1). The data sets are also available on a Github repository [45] together with a notebook that reproduces the figures. Finally, note that the BED files can be recreated using the original FastQ files available on doi:10.7303/syn10638358. We also provide recipes to create the BED files from the FastQ files as notebooks in [45]. All notebooks mentioned are available in [45].

Declaration

List of abbreviations

- BAM: Binary Alignment Map, the binary version of the Sequence Alignment Map (SAM) format.
- BED: Browser Extensible Data
- BOC: Breadth of Coverage
- CNV: Copy Number Variation
- DOC: Depth of Coverage
- CV: Coefficient of Variation
- EM: Expectation Maximization
- HTS: High Throughput Sequencing
- MA: Moving Average
- MLE: Maximum Likelihood Estimate
- RM: Running Median
- ROI: Regions of Interest, samples within a data set identified for a particular purpose.
- SNP: Single Nucleotide Polymorphisms

Competing Interests

All authors have no conflicts of interest to this manuscript.

Funding

This work has been supported by France Génomique consortium ANR10-INBS-09-08.

Author's Contributions

D.D. and T.C. conceived the study. D.D and T.C. implemented the software. C.B. provided the data. T.C. did the CNV studies. D.D. and T.C. contributed to the initial writing. D.D., T.C., C.B and S.K contributed to the final manuscript. All authors contributed to writing and revision and approved the submission.

Acknowledgements

We are grateful to Nicolas Escriou (Institut Pasteur) for providing the FastQ and reference of the Virus test case. We are also grateful to Benoit Arcangioli (Institut Pasteur) and Serge Gangloff (Institut Pasteur) for providing the FastQ files and reference of the S. Pombe test case. We thank Juliana Pipoli da Fonseca for her various comments on the manuscript. We are also grateful to the reviewers who suggested the CNV studies.

References

1. Goodwin, S., et al. (2016) Coming of age: ten years of next-generation sequencing technologies. Nature Reviews Genetics, 17(6), 333-351.
2. Wang, Z. et al. (2009) RNA-Seq: a revolutionary tool for transcriptomics. Nature reviews genetics, 10 (1), 57-63.
3. Meyerson, M. et al. (2010) Advances in understanding cancer genomes through second-generation sequencing. Nature Reviews Genetics, 11(10), 685-696.
4. Iorio, F. et al. (2016) A Landscape of Pharmacogenomic Interactions in Cancer. Cell, 166(3), 740-754.
5. Eid, J. et al. (2009) Real-time DNA sequencing from single polymerase molecules. Science 323, (5910) 133-138.
6. Lee, H. et al. (2004) Error correction and assembly complexity of single molecule sequencing reads. BioRxiv, 006395.
7. Eisenstein, M. (2012) Oxford Nanopore announcement sets sequencing sector abuzz Nat. Biotechnology 30(4), 295-296
8. Li, H. (2013) Aligning sequence reads, clone sequences and assembly contigs with BWA-MEM. arXiv preprint arXiv:1303.3997.
9. Bankevich, A. et al. (2012) SPAdes: a New genome assembly algorithm and its applications to single-cell sequencing. J. Comput. Biol. 19(5): 455-477.
10. Lander, E.S. and Waterman, M.S. (1988) Genomic mapping by fingerprinting random clones: a mathematical analysis. Genomics, 2(3), 231-239.
11. Wendl, M.C. and Barbazuk, W.B. (2005) Extension of Lander-

Waterman theory for sequencing filtered DNA libraries. BMC Bioinformatics, 6(1):245.

12. Ajay S.S., Parker S.C., Abaan H.O., Fajardo K.V., Margulies E.H. (2011) Accurate and comprehensive sequencing of personal genomes. Genome Res. 21(9):1498-505.
13. Mirebrahim, H. et al. (2015) De novo meta-assembly of ultra-deep sequencing data. Bioinformatics, 31(12), i9-i16.
14. Yoon S., Xuan Z., Makarov V., Ye K., Sebat J. (2009) Sensitive and accurate detection of copy number variants using read depth of coverage. Genome Research 19:1586-1592.
15. Brynildsrud, O., Snipen L.G, Bohlin J. (2015) CNOGpro: detection and quantification of CNVs in prokaryotic whole-genome sequencing data. Bioinformatics, 31(11), 2015, 1708-1715.
16. Abyzov A., Urban A.E., Snyder M., Gerstein M. (2011) CNVnator: An approach to discover, genotype, and charaterize typical and atypical CNVs from family and population genome sequencing. Genome Research 21:974-984.
17. Zhao M, Wang Q., Wang, Q, Jia P., Zhao Z. (2013) Computational tools for copy number variation (CNV) detection using next-generation sequencing data: features and perspectives. BMC Bioinformatics 2013, 14 (Suppl 11):S1.
18. Lindner, M.S. et al. (2013) Analyzing genome coverage profiles with applications to quality control in metagenomics. Bioinformatics, 29(10) 1260-1267.
19. Quinlan, A.R. and Hall, I.M., (2010). BEDTools: a flexible suite of utilities for comparing genomic features. Bioinformatics. 26, 6, pp. 841-842. http://bedtools.readthedocs.io
20. Tong, S.Y. et al. (2015) Genome sequencing defines phylogeny and spread of methicillin-resistant Staphylococcus aureus in a high transmission setting. Genome Res., 25(1), 111-118.
21. Bremer, H. Churchward, G (1977) An examination of the Cooper-Helmstetter theory of DNA replication in bacteria and its underlying assumptions. Journal of Theoretical Biology, 69(4): 645-654.
22. Prescott, D.M. and Kuempel, P.L., (1972) Bidirectional replication of the chromosome in Escherichia coli. Proceedings of the National Academy of Sciences, 69(10): 2842-2845.
23. European Nucleotide Archive (ENA). http://www.ebi.ac.uk/ENA. Accessed 8 Sept 2017.
24. Combredet, C. et al. (2003), A molecularly cloned Schwarz strain of measles virus vaccine induces strong immune responses in macaques and transgenic mice. J. Virol., 77(21): 11546-11554
25. Wood, V. et al., (2002) The genome sequence of Schizosaccharomyces pombe. Nature 415(6874), 871-880.
26. Sages's Synapse platform https://www.synapse.org. Accessed 8 Sept 2017.
27. Supporting materials on Synapse project page (BEDs, FastQs, Genome references and genbanks). http://dx.doi.org/doi:10.7303/syn10638358. Accessed 8 Sept 2017.
28. Percival, D.B. and Walden, A.T. (1993) Spectral analysis for physical applications. Cambridge University Press.
29. Balasubramanian, R. et al. (2005) GEO 600 online detector characterization system. Classical Quant. Grav., 22(23), 4973-4986.
30. McKinney, W. Data Structures for Statistical Computing in Python, Proceedings of the 9th Python in Science Conference, 51-56 (2010).
31. Dempster, A.P. and Laird,N.M., and Rubin,D.B. (1977). Maximum likelihood from incomplete data via the EM algorithm. Journal of the royal statistical society. Series B (methodological) 39(1) 1-38.
32. Cokelaer, T. and Desvillechabrol, D. and Legendre, R. and Cardon, M. (2017) Sequana: a Set of Snakemake NGS pipelines. The Journal of Open Source Software, 2, 16 https://doi.org/10.21105/joss.00352. Accessed 8 Sept 2017.
33. Köster, J., and Rahmann, S. (2012). Snakemake - a scalable bioinformatics workflow engine. Bioinformatics, 28(19), 2520-2522.
34. Cokelaer, T. et al.(2013). BioServices: a common Python package to access biological Web Services programmatically. Bioinformatics, 29(24), 3241-3242.
35. Ewels P., Magnusson M., Lundin S., Käller M (2016) MultiQC: Summarize analysis results for multiple tools and samples in a single report Bioinformatics 32, 19, 3047–3048.
36. Dohm, J.C. and Lottaz, C. and Borodina, T. and Himmelbauer, H. (2008) Substantial biases in ultra-short read data sets from high-throughput DNA sequencing. Nucleic Acids Res. 36(16): e105
37. Mohanty, S.D. (2002). Median based line tracker (MBLT): model independent and transient preserving line removal from interferometric data. Class. Quantum Grav., 19(7): 1513-1519.
38. Jones, E. and Oliphant, T. and Peterson, P. et al. (2001) SciPy: Open source scientific tools for Python.
39. Mokry, M. et al (2010) Accurate SNP and mutation detection by targeted custom microarray-based genomic enrichment of short-fragment sequencing libraries. Nucleic Acids Res. 38(10) e116
40. Desvillechabrol D., Legendre R., Rioualen, C., Bouchier C., van Helden J., Kennedy Sean, Cokelaer, T. (2017) Sequanix: a dynamic graphical interface for Snakemake workflows. Bioinformatics, 10.1093/bioinformatics/bty034.
41. Sims, D. et al. (2014) Sequencing depth and coverage: key considerations in genomic analyses. Nature Reviews Genetics, 15(2), 121-132.
42. Conda: Package, dependency and environment management for any language. https://conda.io/docs. Accessed 8 Sept 2017.
43. Grüning, B., Dale, R., Sjödin, A., Chapman, B.A, Rowe, J., Tomkins-Tinch, C., Valieris, R., Köster J., The Bioconda Team. (2018) Bioconda: sustainable and comprehensive software distribution for the life sciences. Nature Methods 15, 475-476.
44. Kurtzer, G.M and Sochat, V. and Bauer, M.W. (2017) Singularity: Scientific containers for mobility of compute. PLoS One. 12(5).
45. The Sequana resources GitHub repository. https://github.com/sequana/resources/coverage. Accessed 23d Feb 2018.

Appendix

Running median implementation

The mean is a measure of the central tendency of a population. It is not a robust estimator in the presence of large extraneous outliers in the population. In such a situation, it is preferable to consider a truncated mean or a median estimator. The median is the middle point of a sample set in which half the numbers are above the median and half are below. More formally, let us consider a sample $s[i], i = 1, .., n$ and $S[i]$ the sequence obtained by sorting $s[i]$ in ascending order (ordering of equal elements is not important here). Then, the median is defined as

$$\nu = \text{median}(\{s[1], s[2], .., s[n]\}) = \begin{cases} S\left[\frac{n+1}{2}\right] & n \text{ odd}, \\ \frac{S[n/2]+S[n/2+1]}{2} & n \text{ even}. \end{cases} \tag{8}$$

Let us now consider a series $X(k)$ where $k = 1, .., N$. Then, the running median of $X(k)$ is defined as the sequence $\nu(k) = \text{median}(\{X(k), X(k+1), .., X(k+W)\})$, $k = W/2, .., N - W/2$ where W is a window size defined by the user and the application. The first $W/2$ and last $W/2$ values are undefined so we should have $W \ll N$.

Since we perform a sorting of an array of W elements at N positions, the complexity of the running median is N times the complex-

ity of the sorting algorithm. If W and N are small (e.g., removal of narrow lines in power spectral density in addition to the overall smoothing of time or frequency series [29]), a naive quick-sort algorithm ($\mathcal{O}(W^2)$ in the worst case scenario) may be used. However, better algorithms do exist and can be decreased to $\mathcal{O}(\sqrt{W})$ in the worst case as implemented in [37]. Yet, in NGS applications, N could easily reach several millions and W may need to be set to large values up to 50,000 (e.g., to identify long deleted regions).

Instead of computing the median at each position, k, a more efficient solution consists in re-using the sorted block at $k-1$, and to maintain the block sorted as new elements are added. Indeed, one only needs to insert the next sample into the sorted block and delete the earliest sample from the sorted block. A standard Python module named bisect provides an efficient insertion in sorted data (keeping the data sorted). The complexity of this sorting algorithm is $\mathcal{O}(\log W)$.

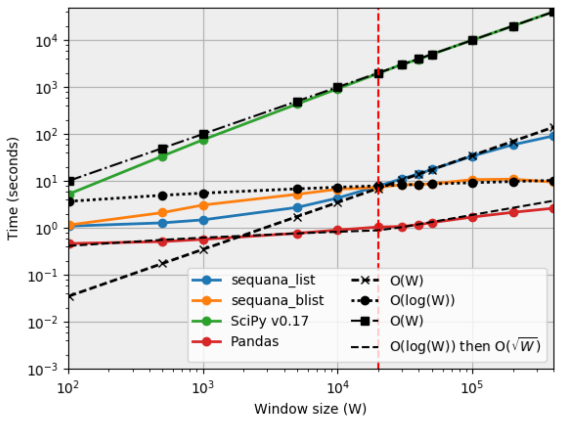

Figure 12. Computational cost of running median algorithms as a function of the window size parameter W (for N = 1e6). Four variants are considered: `SciPy` [38] implementation (function medfilt v0.17), Pandas [30] and 2 Python variants available in `Sequana` based on a `list` or `blist` data containers (see text for details). The `SciPy` variant has a $\mathcal{O}(W)$ complexity irrespective of the W value. For low W values ($W < 20\ 000$), the two Python variants have $\mathcal{O}(\log(W))$ complexity. For larger W values, the blist keeps its $\mathcal{O}(\log(W))$ complexity while the list container follows a $\mathcal{O}(W)$ complexity. Pandas complexity is less clear with a O(W) for $W < 20\ 000$ and $\mathcal{O}(\log(W))$ otherwise. The fastest implementation is clearly the Pandas one even for large W values.

So far, we have neglected the cost of the insertion and deletion steps, which is not negligible. For instance, in Python language, one of the most common data structure is the list. It is a dynamically-sized array (i.e., insertion and deletion of an item from the beginning or middle of the list requires to move most of the list in memory) and the look-up, insertion and deletion have a $\mathcal{O}(n)$ complexity. So the running median is actually dominated by the slow O(n) insertion and deletion steps. A better data structure is available thanks to the blist package; it is based on a so-called B-tree, which is a self-balancing tree data structure that keeps data sorted. The blist allows searches, sequential access, insertions, and deletions in $\mathcal{O}(\log n)$ (see https://pypi.python.org/pypi/blist/ for details).

Based on materials from http://code.activestate.com/recipes/576930/, we have implemented these two variants of running median functions in Python available in Sequana [32] library. We also considered established numerical analysis tools from the `SciPy` [38] and Pandas [30] libraries. We finally compare the four implementations in terms of computation time and complexity as shown in Figure 12. It appears that the Pandas implementation is the fastest. For $W > 20,000$ up to 200,000, our implementation is 2-3 order of magnitude faster than the `SciPy` version but 4-5 times slower than Pandas. We should emphasize the fact that the `SciPy` function has additional features since it is available for N-dimensional data sets whereas we restrict ourselves to 1-D data sets. In `Sequana`, the two variants only differ in the data structure being used to hold the data (list versus `blist`). The Figure 12 shows the difference between the list and blist data structures that is marginal for low W values while for large values asymptotic behaviours are reached showing the interest of the blist over the list choice. We also see that our implementation with blist has a lower complexity than the Pandas implementation. However, for the range considered Pandas is always the fastest choice.
