# Supplementary material for: Sequana coverage: detection and characterization of genomic variations using running median and mixture models
Source: Gigascience. 2018 Sep 6;7(12):giy110. doi: 10.1093/gigascience/giy110 (PMC6275460; doi:10.1093/gigascience/giy110)
Supplement: Response_to_Reviewer_Comments_Original_Submission.pdf [file giy110_response_to_reviewer_comments_original_submission.pdf]

Author's Response To Reviewer Comments

Close

Dear editor,

Please find here below the changes made to the manuscript and supplementary data as well as the answers to the reviewers' comments. In the submission, we also provide a file called main_annotated.pdf with changes highlighted.

The main differences concern the section “Application” that was rewritten to include a discussion on CNV detection. We also added a sub section about the “impact of the running median window parameter”, figure 2 was replaced with 2 different panels and a different example. Other changes are shown in the main_annotated.pdf file. Note also that we changed the title from

Sequana Coverage: Automatic Detection and Characterization of Low and High Genome Coverage Regions.

To

Sequana Coverage: Detection and Characterization of Genomic Variations using Running Median and Mixture Models.

Best regards

Thomas Cokelaer on behalf of the authors

REVIEWER 1
==

QUESTION

The authors presented `sequana_coverage` as a tool that can be used to automatically detect low/high-coverage regions of interest (ROI) on genome sequencing data. The main reason for detecting these regions (parsing from the submission) is to detect > potentially interesting biological features and also to assess the quality of mapping to a reference genome. The submission is quite clear in its scope and the authors should be commended for making the source code open and installation of the too
available via a publicly-accessible repository. The code, though not explicitly shown here, is readable and not difficult to understand. It is also continuously tested, which puts it above a large number of published scientific tools in terms of code quality.
However, there are some concerns and questions that I came across while reviewing the manuscript, which I hope can be clarified and/or explained by the authors in order to improve the quality of the submission.

Major concerns:

The authors highlighted the dangers of using static coverage boundary across the genome and uses it as the rationale of developing the methods described in the submission (last 4 paragraphs of the background section). However, there are two important use cases here that should be better distinguished: the use of coverage for assessing quality metrics or for detecting biologically interesting features. Since the authors mentioned Type 1 and 2 errors, one has the impression that it is the latter use of coverage that is being discussed. Unfortunately, this does not make for a convincing rationale for developing the tools since published methods that do use coverage for detecting features (for example, copy number variation detection or simple structural variation detection) do not employ fixed coverage boundary to do so but instead rely on complex statistical methods tailor-made for the problem at hand [for example, see Brynildsrud et al. 2015 [https://www.ncbi.nlm.nih.gov/pubmed/25644268]). Could the authors comment on this?

ANSWER:

We originally designed sequana_coverage to quickly access standard metrics such as depth of coverage (DOC) or breadth of coverage (BOC) and also visualise the coverage for viral and bacterial genomes. We also wanted a quick and efficient means to visualise and detect atypical significant events that occur in the depth of coverage signal. The goal here was to detect potentially interesting biological features including deleted genes but also much shorter features.

"one has the impression that it is the latter use of coverage that is being discussed." : Indeed the manuscript, we focused on the second aspect, which should be clear from the background section that has been edited accordingly.

"this does not make for a convincing rationale for developing the tools since published methods that do use coverage for detecting features ... do not employ fixed coverage boundary…. " We use fixed threshold as an example to emphasize the DOC biases that exist in replicating bacteria genomes. We then provide a solution (running median). We then complement our approach with robust statistical method.

QUESTION

One application I was hoping to see explained (or at least compared to) is the detection of copy number variation. There, the detection of features are similarly based on sequencing coverage and no pre-set coverage values need to be set for detection. Zare, et al. (2017) [https://bmcbioinformatics.biomedcentral.com/articles/10.1186/s12859-017-1705-x] calls this the read-count based method for detecting CNVs. Seeing that CNVs also represent interesting biological features, an omission of this discussion makes the submission feel incomplete.

ANWSER:

Our tool was not designed to specifically detect CNVs, however the reviewer's comments make it clear that this would improve our tool and broaden its interest. To that end, we added a section addressing CNV detection. sequana_coverage is sown to be competitive with existing tools such as in Brynildsrud et al., 2015 or Abyzov et al 2011 (CNVnator). This was not

mentioned in the original manuscript and we have updated the code and the manuscript to emphasize this capability.

In particular, we have compared sequana_coverage with results found in the supplementary results from Brynildsrud et al.
Our comparison shows that we can retrieve the properties of the CNVs (position, length, copy number); we can detect the same CNVs (>1000 bp) and could also detect short features (1 to 1000 bp), which were not reported in Brynildsrud et al.

As an example, we show in the following image that the CNV at position 2,874,000 is detected with the exact position and length (+- 1 base; horizontal colored segments) whereas CNOGpro split the event into 5 parts and reports starting and ending position with large offset. See for example figure here:

https://tinyurl.com/y7jy5agt

The intention of the manuscript was not, and is not, to claim that sequana_coverage is better or worst than CNOGpro or other such tools. This use of sequana_coverage illustrates that this potentially interesting biological feature, CNVs, can be detected and brought to the attention of the biologist. As such, sequana_coverage is competitive with respect to CNOGpro.

We have also looked at another tool called CNVnator. On the same species and test example as above, we found that CNVnator and sequana_coverage identify the same CNVs. In terms of calculation time, sequana_coverage was as fast (faster for viral and bacterial) as CNVnator.

We also compared sequana_coverage and cnvnator on a human genome (NA12878 from the 1000 genomes project) as described in the notebook available here:

https://github.com/sequana/resources/blob/master/coverage/comparison_cnvnator_human/human_case.ipynb

A full comparison of the two tools is not within the scope of this manuscript given the complexity of the human genome. Yet, recognizing the potential interest of the community,

we updated sequana_coverage and demonstrate we can analyse such data with a reasonable memory footprint (3Go; CNVnator requires 6.5Go). We subsequently analyzed the 24 human chromosomes with both tools. CNVnator took 5.5 hours on a single CPU and could analyse the same data in a an hour on a dual-core. We found that sequana_coverage required about 20 hours to complete. We decided to provide a simple Snakemake (https://snakemake.readthedocs.io/en/stable/) pipeline to analyse the chromosome in parallel on a cluster (24 chromosome files using 24 CPUs). With this configuration, the analysis took 1 hour.

Additional features are complement existing CNV detection tools because:
- It can also detect short features thanks to the running median.
- The underlying algorithm is simple (running median + normalisation, +

mixture model) but is nevertheless
based on solid statistical considerations
- HTML reports are provided, which is very convenient in the context of viral and bacterial genomes
- Multiqc report are now available to compare different contig statistics
- Snakemake pipeline is provided to analyse several chromosomes in parallel
- The entire code is in Python and researchers can built on top of it as demonstrated n the numerous notebooks that have been provided in this open resource page on github: https://github.com/sequana/resources/tree/master/coverage

In summary, we have improved the manuscript (and sequana_coverage) significantly (application section) by adding a dedicated section on CNVs detection.

QUESTION

It would make for a stronger submission if the features detected using `sequana_coverage` and how they would look like using a naive setting of coverage depths is discussed. The bacteria test case explained further in Figure 7, while interesting, does not > really convince the readers on the need of this tool as the changes in coverages seem to be visible even without using the tool (i.e. just by plotting the raw coverages). In figures 8-9 it seems to be the case as well that detection of the ROI can be done by > using only the mapping quality track.

ANWSER:

The bacteria test case example of the figure 7 (previous version) has been removed. Instead, the new figure 2 (a virus example) is more convincing:

https://tinyurl.com/yc6a75js
https://tinyurl.com/y884rjwl

In the region shown, we have a deleted event (15,000 bases) followed by a depleted event (5,000 bases) with a copy number of 0.5 separated by 5,000 bases. This better emphasizes the inability of a moving average to detect the depleted event. The red/green dots indicate detected events: there are many false positives in the left panel, while the running median (right) has a much better performance.

Figure 8 and 9 have been removed and replaced with examples related to CNV detections.

We are convinced of the utility of using a running median. As space in the text is limited, we provide a notebook that explains the negative impact of the constant threshold in the presence of replication or non-constant depth of coverage along the genome (it also contains examples that motivates the use of a running median instead of moving average):

https://github.com/sequana/resources/blob/master/coverage/running_median_motivation/running_median.ipynb

QUESTION

What are the characteristics of the features that can be detected using this tool. For example, are they between a specific size range (in relation to, for example, the window size and/or the read length). This is not completely clear from the submission, and while some methods of correlating the detected features to known biological features are presented, one wonders if such detection can be made with simpler methods.

ANWSER

Sequana_coverage can detect any events from 1 base to W/2 bases. There was some confusion as to how W is set, and it's optimal value. We have clarified the text to explain the impact of the window length parameter, W, adding a section called 'Impact of the running median parameter'. We have also provided a notebook with examples and figures:

https://github.com/sequana/resources/tree/master/coverage/window_impact

In summary, in order to detect (and avoid the impact) of a deleted or duplicated event of length N, one should use a window parameter W > 2N . This is independent of the genome size. However, if you have a small genome (e.g. virus), then the window size must be less than this genome size, of course. In such situation, we would recommend a fifth of the genome size.

The impact of the W parameter on z-score is marginal. One should not set W to large values (e.g., 500,000) otherwise the trend will not be correctly estimated. We also recommend a W>20,000 ; below this there is a slight increase of false positives (see image below and notebooks https://github.com/sequana/resources/tree/master/coverage/window_impact).

https://tinyurl.com/y888n8jd

So, we can detect events from 1 base to W / 2 bases. The drawback of using larger windows are (i) slower computation, although the implementation in place is very efficient so this is marginal, (ii) too much smoothing will decrease the detection power if the signal is very noisy.

We have changed the text to mention those different aspects.

One final point concerns the reviewer's allusion to 'simpler methods.' We feel that sequana_converage takes into account the biases and variability inherent in both biological systems as well as HTS data. Indeed part of our motivation is to accurately account for significant variations in DOC in sequencing data that may or may not be biologically relevant. These are not simple systems and thus require analysis with tools adapted to the task.

Minor concerns:

QUESTION

Although the tool here is presented as a standalone tool, installing it requires installation of other tools and pipelines in the authors' sequana` toolkit. It would be more suitable if the tool is also packaged as its own, truly standalone package.

ANSWER

The easiest and most robust way to use sequana_coverage is to use the singularity solution. Singularity tool (http://singularity.lbl.gov/) can be installed in minutes.

We provide sequana (version 0.7.0) as an image on Singularity hub https://www.singularity-hub.org/collections/114) .
Although the image is 1.7Gb it contains all the tools required. It does not require any compilation and is reproducible. We are not aware of a simpler solution that would provide an executable that would work locally and on a cluster.

We also provide sequana version 0.7.0 on bioconda (https://www.biorxiv.org/content/early/2017/10/21/207092) , in review in Nature Method. and on Pypi website for python developers.

Everything is documented on sequana.readthedocs.io in particular, please see http://sequana.readthedocs.io/en/master/installation.html#singularity

The reason we do not provide sequana_coverage as a standalone is because of manpower: we decided to keep the coverage tool within the sequana library to ease the development and debugging of the tool and allows us to be more responsive. We have every intention of maintaining this tool in the future, and this structure is most conducive with this goal.

QUESTION
The version of the `sequana_coverage` tool used for producing the plots is unfortunately not mentioned clearly. Could the authors clarify this?

ANWSER
The version used in the manuscript and notebooks is 0.7.0, which is now also written in the manuscript.

QUESTION
The EM algorithm to which the author refers in the statistical section, should be expanded as Expectation Maximization instead of Expectation Minimization. This is how it was presented in the referred Dempster et al. (1977) paper.

ANSWER
Thank you for catching this oversight. It has been corrected.

QUESTION

The rationale on choosing the running median window size is not completely clear. Are there any relations between this value and, for example, the read length or the reference genome size?

ANSWER

As mentioned above, in order to detect a deleted or duplicated event of length N, one should use a window parameter W such that W > 2N , which is independent of the the genome size, coverage or read length.

Could the author comment on the suitability of using `sequana_coverage` for other species such as humans, mice, and/or some plant species?

ANSWER

We have used sequana_coverage for viruses, bacteria and fungi. However, in the notebook

https://github.com/sequana/resources/tree/master/coverage/comparison_cnvnator_human

we also demonstrate that it can be used for humans. We have now updated the code that allows the analysis of a human genome in an hour (+1 hour to convert BAM to BED files), which is competitive with dedicated tools such as CNVnator (5-6 hours).

QUESTION

Page 2, line 27-30. The authors wrote: "For instance, to detect human genome mutations, single-nucleotide polymorphisms (SNPs), and rearrangements, a 50 X depth is recommended [1] to be able to distinguish between sequencing errors and true SNPs." Could the authors clarify which table and/or statements present in the cited article that contains this recommendation?

ANSWER

This refers to reference 15 (Ajay et al., 2011) of the reference 1. We changed the text to add the missing reference. We now refer to a range 30-50X instead of 50X since this really depends on the application (SNPs, SNV) and technologies.

REVIEWER2
===

In this manuscript, Desvillechabrol et al. introduce seqana_coverage, a method and tool for estimating statistically significant deviations in genome coverage data and providing an automated method to detect genomic regions of interest (ROI). The overall algorithm method seems sound, and consists of de-trending the data using a running-median filter, followed by learning foreground (center) and background (outlier) distributions, as well as their maximum likelihood mixture proportions in the sample. Finally, given the Gaussianity assumptions of the model, they define a z-score that can be computed for each base in the genome, which can aid in discovering loci that exhibit statistically surprising high or low coverage. I generally find the manuscript convincing, and the tool looks as though it will be useful. However, I have a couple of high-level questions about the proposed solution and the

manuscript.

Questions / comments ----

There is not much discussion in the current manuscript on the effect of W, the window length used in the running-median de-trending algorithm. Do the authors have a recommended procedure for setting W? It would seem that the choice of W will set a "scale" for outliers, and while the approach would generally be robust to small changes in W, larger changes could highlight different regions of interest (this, of course, can be viewed as a feature rather than a problem). However, for someone wishing to use the tool, a recommendation on how to set W, or what values make sense for discovering different types of regions of interest, would be useful.

ANSWER

We have clarified the text to explain the impact of the window length parameter, W, adding a section called ‘Impact of the running median parameter’. We have also provided a notebook with examples and figures:

https://github.com/sequana/resources/tree/master/coverage/window_impact

In summary, in order to detect (and avoid the impact) of a deleted or duplicated event of length N, one should use a window parameter W > 2N . This is independent of the genome size. However, if you have a small genome (e.g. virus), then the window size must be less than this genome size of course. In such situation, we would recommend a fifth of the genome size.

The impact of the W parameter on z-score is marginal. One should not set W to large values (e.g., 500,000) otherwise we may not estimate the trend correctly. We also recommend a W>20,000 ; below, there is a slight increase of false positives (see image below and notebooks https://github.com/sequana/resources/tree/master/coverage/window_impact).

https://tinyurl.com/y888n8jd

So, we can detect events from 1 base to W / 2 bases. The drawbacks of using larger windows are (i) slower computation, although the implementation in place is very efficient so this is marginal, (ii) too much smoothing which decreases detection power if the signal is very noisy.

We have changed the text to clarify those different aspects.

QUESTION

Related to the above, one potential effect of the de-trending that is performed in seqana_coverage is that outliers appear to generally be high-frequency effects. Would the proposed approach be applicable if one wished to discover low-frequency effects (i.e. very large ROIs)? For example, one might expect a dip in coverage in difficult-to-sequence regions or in regions with highly-repetitive arrays of elements. Would it be possible to detect such large-scale regions using the proposed approach, or do they not classify as

outliers under the assumed definition?

ANWSER

Sequana_coverage can detect any events from 1 base to W/2 bases. We generally set W to 20,000 but it can be increased safely to 50,000, or even larger values. For instance, when the goal is really on the detection of large events, such as those to be found in human genome, we used values of 250,000 or even 500,000. We then compared to CNVnator, a CNV dedicated tool, as shown in:

https://github.com/sequana/resources/tree/master/coverage/comparison_cnvnator_human

Certainly a much more detailed investigation would be needed to fully characterize and compare the behaviour of sequana_coverage algorithm on this kind of data. This type of analysis of human CNV detection is out of scope with the current manuscript; we indeed focused on bacterial genome at first. Nevertheless, it looks promising both in terms of computational time and overlap with CNVnator. Larger values for W would not be recommended since the running median would not follow the trend closely, especially in the presence of long deleted regions, which are common in human genomes

QUESTION

I'm not certain I understand, or completely agree with, the argument that the outlier distribution can safely be assumed as Gaussian. Specifically, for the CLT to apply, we must be taking the (normalized) sum of a large number of *independent* random variables. However, it's not immediately clear to me why the outlier samples are independent --- e.g., changes due to repeated elements may be be correlated. Also, given the definition of the outlier samples (those with considerably higher or lower coverage than their local, de-trended window), it's not completely clear why this distribution may not be e.g., bimodal. Perhaps the assumption of normality is simply a computational convenience, as it allows closed-form update rules for the mixture model being applied. But perhaps this assumption could be addressed empirically (e.g., by looking at a goodness-of-fit test of the outlier samples to a normal distribution with the inferred mean and standard deviation).

ANSWER

Our assumption of normality of the distributions that compose the normalised coverage is empirical. Moreover, as the reviewer stated in his comment/question, this is also a convenient choice to allow us to fit a mixture of Gaussian models. However, low depth of coverage does not exhibit a Gaussian distribution, and we do not recommend to use sequana_coverage for coverage below 10X.

As for the possible bimodal distributions of outliers on each side of the central distribution; this is indeed a possibility. However we are not concerned by the outliers at this stage, as the most important aspect is the statistical properties of the central distribution. This is why we use k=2 in the mixture models and why we assume the central distribution to be predominant.

Our hypotheses is that the central distribution is Gaussian, which looks reasonable

in the cases considered. For instance, in the case of a simulated data set, the mapped data is normalised and exhibits a central distribution that is gaussian (see link to figure below).

https://tinyurl.com/y9ues5t4

QUESTION

It would be nice if the authors could provide a couple of examples demonstrating the underlying biological cause of some of the outliers identified by their algorithm. Specifically, the algorithm is demonstrated on 3 real datasets, and outliers are predicted. It would be a nice addition to the manuscript to select a few extreme outliers and discuss what gave rise to them.

ANSWER

We have updated the manuscript, and now show examples from data from CNOGpro (6 strains of Staphylococcus) to emphasize the ability of sequana_coverage to identify CNVs with perfect location and size, in itself an ‘extreme’ case when deal with human data. However, because the manuscript now has a dedicated section on CNVs, we are lacking place for extra examples demonstrating the underlying biological cause of some of the outliers.

Figure 9 also demonstrates a potentially extreme event, where a duplication of 10X has no impact on the detection of the two flanking events of lesser intensity. Even were a region to have 100X or 1000X coverage, it would have no impact on surrounding events as long as the size is less that W / 2. A strength of the running median is that extreme events are handled exceptionally well.

Minor concerns -----
The second sub-section of the methods section is titled "Building a statistics", which sounds awkward and should probably be reworded.

Changed to “Parameter estimation of the central distribution and adaptive thresholds in the original space ”, which is less concise but is more explicit.

In the same section, when justifying the Gaussianity assumption for the outlier distribution, the authors claim "we can consider that the outliers population is a mix of samples and that we are in the limit of the central theorem". The wording here is a bit strange --- I would, instead, suggest something like "if we consider the outlier population as arising from a mix of different independent samples, then the central limit theorem applies, implying that $\tilde{C}_b^1$ can be treated as Gaussian."

The wording is now that suggested by the reviewer. Thank you.

REVIEWER3
==

This manuscript presents a novel method for detecting regions of anomalous mapped read depth. As I understand it from the manuscript, the expected uses of this appear to be to detect problems with either a genome or metagenome assembly or sequencing sample, and to assess the GC bias in a sample. The authors do really address the other application that might seem obvious: the calling of CNVs. The following review will be broken down into two pieces: the manuscript and the software. I will conclude with a summary of my recommendations.

I have two major concerns:
Any new piece of software aiming to solve a problem where other pieces of software attack the same problem should be compared to these other tools. While the authors do mention a couple of other approaches, mostly from the field of metagenomics, there exist a range of tools that use read depth to investigate CNVs. See for example Yao et al Molecular Cytogenetics 2017 10:30 doi:10.118/s13039-017-0333-5, Zhao et al BMC Bioinformatics 2013 14(Suppl 11):S1 doi:10.1186/1471-2105-14-511-S1. The authors should set their method in the context of these methods and how how it differs in its aims/approach/performance. If applicable a comparison between their method and others on the same dataset should be presented. At a minimum I would like to see a comparison to CNVnator (Abyzov et al, Genome Research, 2011 21:974-948, doi: 10.1101/gr.114876.110) or an explanation of why such a comparison is not appropriate.

ANWSER

Indeed, the original version of our manuscript did not mention CNVs or make reference to existing CNV detection tools. Thanks to the reviewers comments and feedbacks, we significantly changed the application section, adding specific reference to CNV detection. We have addressed this issue in significant depth by comparing sequana_coverage with
CNOGpro, which is primarily aimed at bacterial genome. For comparison we re-used their data sets, as the authors also selected Staphylococcus as a test case (6 strains). We also compared CNVnator, which is dedicated to whole eukaryotic genomes (human).

We have several notebooks where we compare CNOGpro results with the sequana_coverage (also CNVnator). :

https://github.com/sequana/resources/tree/master/coverage/08-comp_CNOGpro_cnvnator_sequana_bacteria

https://github.com/sequana/resources/tree/master/coverage/09-comparison_cnvnator_bacteria

https://github.com/sequana/resources/tree/master/coverage/10-comparison_cnvnator_virus

https://github.com/sequana/resources/tree/master/coverage/11-comparison_cnvnator_human

In the following figure, there is an obvious CNV event with copy number of 3.5. Coverage data for one strain (black curve) is shown with the corresponding sequana_coverage event detection (horizontal black line). The other 5 horizontal

coloredline show the reported event in the 5 other strains. This demonstrates a few important points: (1) all strains exhibit the same event (2) sequana_coverage called all 6 cases (3) the precision of the position and length of the events is at the same position (precise). The reported mean coverage of the events allows us to determine the average copy number. In comparison, CNOGpro identifies the events, but splits them into several events (colored green and red areas) because it uses a different paradigm (split of the genome into intergenic segments; here the green and red areas). CNVnator, by comparison, is also very precise and consistent in identifying this event.

https://tinyurl.com/y7jy5agt

This is just one example (more are provided in the notebook). The main conclusions are that sequana_coverage identifies the same events as CNOGpro and CNVnator and is often more precise. We tested sequana_coverage for human genomes and found that it is competitive with CNVnator in terms of computational time even though a full comparison on human is evidently more difficult than for bacteria, which could be checked by hand.

As reported in Zhao et al., existing CNV tools (48 tested !) do not agree and should be used together rather than independently. Thus, sequana_coverage can be considered as a good complement to the existing tools. Moreover, one additional strength is that it can detect short events that are missed by CNOGpro and CNVnator as explained in the notebook:

https://github.com/sequana/resources/tree/master/coverage/comparison_cnvnator_bacteria

Here is an example of such an event where sequana_coverage detects 4 events (orange and blue areas) while CNVnator detects only the first and last, completely missing the short strong event and the first long but weak depleted region:

https://tinyurl.com/yaycvxzf

Of course, the different results depend on parameters in the different tools,. Nevertheless, this kind of result is observed with CNOGpro as well, and the underlying mean-shift tool is the cause of the missed events.

We have now added a 2-page section in the application section with further details in response to the reviewer’s comments and suggestions.

QUESTION

The algorithm has a single tuning parameter, W. The authors state that the value of this setting has little effect on the parameters learnt by their model. The authors should expand on this further: presumably the choice of W does have an effect on the z score obtained For example I believe any region of elevated or depressed coverage larger than W would clearly be missed. The authors should include a discussion of how changing W changes the results and how to choose a good value for W.

ANSWER

We have clarified the text to explain the impact of the window length parameter, W, adding a section called ‘Impact of the running median parameter’. We have also provided a notebook with examples and figures:

https://github.com/sequana/resources/tree/master/coverage/window_impact

In summary, in order to detect (and avoid the impact) of a deleted or duplicated event of length N, one should use a window parameter W > 2N . This is independent of the genome size. However, if you have a small genome (e.g. virus), then the window size must be less than this genome size of course. In such situation, we would recommend a fifth of the genome size.

The impact of the W parameter on z-score is marginal. One should not set W to large values (e.g., 500,000) otherwise we may not estimate the trend correctly. We also recommend a W>20,000 ; below, there is a slight increase of false positives (see image below and notebooks https://github.com/sequana/resources/tree/master/coverage/window_impact).

https://tinyurl.com/y888n8jd

So, we can detect events from 1 base to W / 2 bases. The drawback of using larger windows are (i) slower computation, although the implementation in place is very efficient so this is marginal, (ii) too much smoothing will decrease the detection power if the signal is very noisy.

We have changed the text to mention those different aspects.

QUESTION
There are also a number of smaller concerns or comments. Under "building a statistic" the authors state that the per-base coverage should theoretically follow a poisson distribution. Is it not actually the case that it is the number of reads that start at any given base that should follow a poisson distribution, and the non-independent nature of depths and nearby bases may account for some of the over-dispersion?

ANSWER

We have added a reference (Lindner et al.) concerning the Poisson distribution . We have not found any reference regarding a theoretical reason for the over-dispersion.

QUESTION

It is not clear to me why the gaussian approximation of the negative binomial becomes valid at 10X coverage, rather than 5 or 20.

This is an empirical choice. One can analyse data with coverage of 5X, as we did for the human genome. However, we can see (visually) that the distribution is skewed to large depth, meaning the gaussian approximation is not valid.
One can still change the threshold to reduce the number of reported events.

Practically, we propose 10X to be a level of coverage where the statistical assumptions upon which event detect is based become valid. (Ajay et al., 2011), cited in the manuscript, suggests 50X coverage for the detection of certain events and the biologist must assume some responsibility for the amount of sequencing necessary to answer a specific question..

QUESTION

The authors state that bases of 0 coverage are not included in their fitting, but would these values not be important in finding the mixing parameters?

ANSWER
No, we are interested in fitting a distribution on the central part of the distribution. The zeros are zeros and belong to the population of outliers. Yet, if we include them, they may have a negative impact of the EM algorithm. So, we decided to ignore them.

QUESTION

The authors state that one of the benefits of their approach is that a statistically meaningful value is attached to each base and that they can control the false positive rate this way. However, they then go on to use a two step threshold to identify regions. It seems to me that that the above benefit does not transfer over to the regions and it is difficult to assess the statistical properties of the regions. Perhaps the authors should comment on this in their discussion.

ANSWER
With the algorithm in place, a z-score on a per-base is computed. The 2-step threshold allows us to cluster events. In order to report a dedicated z-score (probability to see N bases crossing a threshold), we should assume that two consecutive bases are independent, which is not the case. So, we report the mean-zscore and max z-score of the events. A robust statistical property for such events would require more investigations.

QUESTION
The authors comment on how the method scales to viral, bacterial and yeast datasets. How would the performance scale if applied to mammalian or plant genomes?

ANSWER

Since the first version of the manuscript, we have improved the code to handle eukaryote data sets. We use the same data set from the 1000 genomes project as in CNVnator (3.5Gb). We found that performance scales well and that we can analyse the data in a couple of hours, compared with CNVnator that took 5-6 hours. To be fair, CNVnator takes as input a BAM file whereas sequana_coverage still needs the BED file as input. So, if we take into account the BAM to BED conversion, this needs an extra hour but is done once for all. Finally, to reach a 2-hours computational time, we also use the same binning of 100 used by CNVnator.

Software
Of the three available installation routes I was able to install the software without an error message at install time using only one route - via the sequana conda package. I was a little perturbed to be made to download and install several Gb of dependencies just to use what one would image would be a fairly dependency-lite piece of software. Indeed I several times ran out of disk space on both the Ubuntu virtual machine and institutional HPC accounts I attempted the installation on. This was due to having to download an install the whole of the sequana collection, which has many dependencies way beyond the scope of the tool under review here. I obtained the viral dataset used in the manuscript from the synergy and attempted to run the tool. The tool seemed to get though most of its processes, unfortunately I was met with an error I was unable to solve in what I assume was the reporting phase. Unfortunately I don't feel that I can provide a full review of the software until I am able to run it fully with out error. My personal suggestion for a tool as self contained as this it would be better it to could be installed on a stand alone basis with a minimal number of dependencies. This would minimise the chance for things to go wrong and also minimise the footprint of the tool on the users system. To be useful to many bioinformaticians, who do most of their work on institutional clusters, it needs to be possible to successfully install without root permissions. Conda should allow this to be possible.

ANWSER

The easiest and most robust way to use sequana_coverage is to use the singularity solution. Singularity tool (http://singularity.lbl.gov/) can be installed in a couple of minutes. We provide sequana (version 0.7.0) as an image on Singularity hub (https://www.singularity-hub.org/collections/114) .

Although the image is 1.7Gb it contains all the tools required. It does not require any compilation and is reproducible. We are not aware of a simpler solution that would provide an executable that would work locally and on a cluster.

We also provide sequana version 0.7.0 on bioconda (https://www.biorxiv.org/content/early/2017/10/21/207092) , in review in Nature Method. and on Pypi website for python developers. For conda, these commands installed sequana_coverage in about 5-10 minutes on a Fedora box:

```
conda create --name sequana_0_7 python=3.5
source activate sequana_0_7
conda install sequana==0.7.0
```

Everything is documented on sequana.readthedocs.io in particular, please see http://sequana.readthedocs.io/en/master/installation.html#singularity

Recommendations

QUESTION
I believe that the work here is a valuable contribution to the field and could be suitable for publication if the authors addressed the comments outlined. Of particular importance:

Comparisons of the method to published methods using read-depth to call CNVs (for example CNVnator).

ANWSER

We have compared sequana_coverage tool with CNVnator and CNOGpro on bacterial genome including 6 isolates of staphylococcus.
Moreover, we compared CNVnator and sequana_coverage on (i) a viral genome, (ii) a human genome. We included a 2 pages section in the new version of the manuscript

QUESTION
A discussion of the effect of changing the W parameter and how to choose a good value

ANWSER

We have added a subsection "Impact of the running median parameter" to discuss this aspect.

QUESTION

I need to be able to install the software flawlessly on, for example, a freshly minted Ubuntu virtual machine or similar (X and conda or pip installed) and run without error. Ideally there should be a non-root requiring way to install.

ANWSER

We recommend the singularity solution as described here:
http://sequana.readthedocs.io/en/master/installation.html#singularity

First install singularity 2.4.2:
Second, download the sequana singularity 0.7.0 image:

singularity pull --name sequana_0_7_0.img shub://sequana/sequana:0_7_0

Third use the standalone:

Singularity exec sequana_0_7_0.img sequana_coverage --input test.bed

Close
