# Supplementary material for: Sequana coverage: detection and characterization of genomic variations using running median and mixture models
Source: Gigascience. 2018 Sep 6;7(12):giy110. doi: 10.1093/gigascience/giy110 (PMC6275460; doi:10.1093/gigascience/giy110)
Supplement: Response_to_Reviewer_Comments_Revision_1.pdf [file giy110_response_to_reviewer_comments_revision_1.pdf]

Author's Response To Reviewer Comments

Close

Dear editor,

The main changes are as followed:

We created a new sub section called Example: viral genome characterisation before the section CNV detection. In this section, we moved the discussion about the viral genome (previously in the CNV detection section), added a Figure and a Table and answered to the first point of the reviewer concerning the false positive (a new image Fig 8 was added to complement the text, one of the reviewer's concern) . Then, in the CNV section, we cleaned up the text with respect to english style by editing the different paragraphs. Finally, we rephrased the last paragraphs of the conclusions; in particular, we clarify the information with respect to the performance of the sequana_coverage tool to analyse the whole human genome (last reviewer's point).

Best regards,

Thomas Cokelaer on behalf of the authors

Reviewer #3:

The authors present a thoroughly updated revision of the manuscript that makes a serious attempt at updating many of my previous points. Following the authors advice, I am now able to install the software on a clean virtual machine through the singularity route. This route would still not present though for users without the necessary privileges to install the singularity software (e.g. institutional HPC), but I guess it is better than many packages. I was able to run the software on the provided example files. In the case of the viral genome I successfully identified, 8 depleted regions and one enriched region (see below).

ANSWER: Given the numbers, we presume that the W parameter was set to 2000 and -o option (circularity) was used. If so, indeed, as shown in the notebook 10, there are 8 depleted regions and one enriched region detected by sequana_coverage. See hereafter for more comments following the next point/question

--

The enriched region is clearly a false positive as its depth is almost exactly at the long term trend for the genome, but is surrounded by two strong depleted regions that are pulling the average down.

ANSWER: Indeed, the enriched region is a false positive caused by the presence of two depleted regions around it. Playing with the W parameter, one can see that for W>4,500 the running median is smoother and that the false positive is not detected anymore. Note that the strength of this False Positive is weak: the mean z-score is around 5.

In the previous version of the manuscript, there was a small discussion in the CNV section concerning the viral case. We have moved and improved that section into a subsection

called “Example: viral genome characterisation”.

The 8 depleted events (2 long and strong, 3 weak events, 3 strong but few bases long) are all detected by sequana_coverage. This number remains stable for various values of the window parameter.

To give a comparison, we shown that CNVnator detects the two large events, do not show a False positive for the enriched region but misses the 3 weak events as well as the 3 short events. We are aware that CNVnator was designed to detect long CNVs so this result is not surprising.

We have also added in the notebook 10 a comparison using CNOGpro, which was designed for eukaryotic genomes. Only the two large depleted events are detected but with poor precision of the actual length. All short events are missed.

So despite the detection of a False Positive with sequana_coverage, the overall behaviour seems more robust and allows the detection of very short events in addition to the longer ones.

We have added a Figure (8) and a Table (2) to illustrate this point in the new section Example: viral genome characterisation

----

The largest update to the manuscript concerns an investigation into the use of sequana_coverage for detecting CNVs. This study begins with a simulation experiment. The authors do not report in the main text the number of false positives discovered in simulations with not simulated CNVs saying only "the number of ROIs detected with sequana_coverage varies from one simulation to the other". Examination of the referenced notebook reveals that their representative example has 14 regions.

ANSWER: Indeed in the notebook, a simulated data set with a coverage of 100X for a bacterial genome of about 3Mb led to 14 detected events but is not reported in the manuscript. We have now performed more simulations as explained hereafter.

The authors state that these false positives "have short lengths (below 50) and low mean z-scores (below 5)." All methods produce false positive; using a threshold of 4, one would expect to find around 200 bases beyond the threshold if each base was independent (which it clearly isn't). Does this mean that ROIs shorter than 50bp and with z-scores below 5 should be ignored in CNV or other analyses, or are these bases identifying genuine features in the genome (such as unmappable sequence)?

ANSWER: Using simulated data, the number and strength of the detected events tell us the level of False Positives that are expected. Indeed, if the data were purely gaussian and independent, we would expect for such a genome (3Mbp) about 200 bases beyond a

threshold of 4. Here we get only about 17 events (we repeated the simulated data 100 times to get a good statistics) probably due to the nature of the mapping process. Those events are False Positives since we have only random reads covering uniformly the genome. By repeating the simulation 100 times (compare to only one simulation in the previous manuscript), we could get a better idea of the distribution of the False Positives. More importantly we can confirm that the detected events are not genuine features in the genome (e.g. due to GC biases, repeats, etc). Indeed, if this was the case, from one simulation to the other we would possibly detect events at the same location (where there is a genuine feature) and this does not happen across the 100 independent simulated data sets: an event of 50 bases is not seen at the same location from one simulation to the other.

In the simulated data, none of the 1800 ROIs have a length above 80 and a mean z-score (absolute value) above 5. So, in our opinion, with this algorithm, short CNVs below 50 or mean z-score below 5 should be considered as noise.

Clearly what might be considered a false positive for one analysis would not be for another, while so me regions are genuinely false. This is a point that could perhaps be made in the discussion.

ANWSER: we have added an image in the manuscript with the distribution of the ROIs on simulated data and include a paragraph in the CNV section to emphasize the fact that events with zscore below 5 and length below 50 should be considered as noise.

The authors then compare their tool to other tools, and find roughly comparable performance, although it is unfortunately that the same level of analysis doesn't seem to have been applied to the sequana_coverage only ROIs as has been applied to the CNVnator and CNOGpro only events. In particular the authors do not mention any false positives in the viral genome. There are several, as I found when I ran the viral genome analysis myself, including one an enrichment that appears to be caused by normal depth sequence sitting between two depleted regions, which could point to the necessity of further W value tuning. The authors briefly touch on performance of the tool on the human genome as requested, although I am somewhat confused as to the results. In the paper they mention that the tools runs as quickly as 30 minutes on a dual core machine for the human genome.

However, in response to another author they say that running on a human bed takes 1 hour on 24 cores, or 20 hours on one core.

ANSWER: There was probably a confusion or an error in our responses due to executions on different machines. We double checked the number. It takes 1 hour to 1 hour and a half tto analyse the 24 chromosomes (sequentially) on one core. To be conservative, we specify less than 2 hours in the text (on a single core). However, there are 24 chromosomes. So, we implemented a Snakemake pipeline that allows the analysis of the 24 chromosomes in parallel. So, it can actually go down to only 7 minutes when using 24 cores, which is the time required to analyse the longest chromosome. In the conclusion, we have added

“the analysis of the 24 human chromosome files should take less than 2 hours (1.5 hours on

an HPC cluster using only one core and 1 hour on a DELL Latitude with a SSD hard disk using only one core)”

And

“The longest chromosome (chr1) with 250Mb is analysed in about 5-6 minutes. A Snakemake pipeline was also recently implemented within sequana (called coverage) allowing each chromosome to be analysed independently. Using 24 cores, we could analyse the 24 chromosomes in about 7-8 minutes, which is basically the time taken to analyse the longest chromosome.”

In their response the authors repeated refer to leaving things out because of limited space, but I was under the impression that GIGA-science did not impose length limits on articles. In particular the revised manuscript contains a large number of references to notebooks stored on the sequana github. I feel like many of these would make useful figures or supplementary figures for those not conversant with the python. One might also wonder if the notebooks would be better as supplementary files rather than links to a potentially volatile github site.

ANSWER: We decided to put the notebooks under a “neutral” organization project on github (rather than a username) so that it can be updated in the future. We can provide a snapshot of the notebook within an archive that can be deposited on giga science web site if required.

Some of the lanuage is less polished than in the original submission.

ANSWER: We believe we have now checked the new sections more thoroughly

---

The authors have address many of my points. While I would appreciate some
further discussion, particularly on the nature of the false-positives, I do not
think that this should prevent publication.

ANSWER: We would like to thank again the reviewer for his suggestions and useful comments that made the final manuscript and the sequana_coverage tool even more useful to the community.

Close
