# Supplementary material for: Sequana coverage: detection and characterization of genomic variations using running median and mixture models
Source: Gigascience. 2018 Sep 6;7(12):giy110. doi: 10.1093/gigascience/giy110 (PMC6275460; doi:10.1093/gigascience/giy110)
Supplement: Response_to_Reviewer_Comments_Revision_2.pdf [file giy110_response_to_reviewer_comments_revision_2.pdf]

Author's Response To Reviewer Comments

Close

I have added the sentence and added the reference in the bibliography as requested.
Regards
Thomas Cokelaer on behalf of the authors

Close
