# Supplementary material for: Sequana coverage: detection and characterization of genomic variations using running median and mixture models
Source: Gigascience. 2018 Sep 6;7(12):giy110. doi: 10.1093/gigascience/giy110 (PMC6275460; doi:10.1093/gigascience/giy110)
Supplement: Reviewer_1_Report_(Original_Submission) -- Wibowo Arindrarto, M.Sc. — 10/16/2018 Reviewed [file giy110_reviewer_1_report_(original_submission).pdf]

Reviewer Report

Title: **Sequana Coverage: Detection and Characterization of Genomic Variations using Running Median and Mixture Models**

Version: **Original Submission** **Date:** 10/16/2017

Reviewer name: **Wibowo Arindrarto**

Reviewer Comments to Author:

The authors presented `sequana_coverage` as a tool that can be used to automatically detect low/high-coverage regions of interest (ROI) on genome sequencing data. The main reason for detecting these regions (parsing from the submission) is to detect potentially interesting biological features and also to assess the quality of mapping to a reference genome.The submission is quite clear in its scope and the authors should be commended for making the source code open and installation of the tool available via a publicly-accessible repository. The code, though not explicitly shown here, is readable and not difficult to understand. It is also continuously tested, which puts it above a large number of published scientific tools in terms of code quality.However, there are some concerns and questions that I came across while reviewing the manuscript, which I hope can be clarified and/or explained by the authors in order to improve the quality of the submission.Major concerns:* The authors highlighted the dangers of using static coverage boundary across the genome and uses it as the rationale of developing the methods described in the submission (last 4 paragraphs of the background section). However, there are two important use cases here that should be better distinguished: the use of coverage for assessing quality metrics or for detecting biologically interesting features. Since the authors mentioned Type 1 and 2 errors, one has the impression that it is the latter use of coverage that is being discussed. Unfortunately, this does not make for a convincing rationale for developing the tools since published methods that do use coverage for detecting features (for example, copy number variation detection or simple structural variation detection) do not employ fixed coverage boundary to do so but instead rely on complex statistical methods tailor-made for the problem at hand [for example, see Brynildsrud et al. 2015 [https://www.ncbi.nlm.nih.gov/pubmed/25644268]). Could the authors comment on this?* One application I was hoping to see explained (or at least compared to) is the detection of copy number variation. There, the detection of features are similarly based on sequencing coverage and no pre-set coverage values need to be set for detection. Zare, et al. (2017) [https://bmcbioinformatics.biomedcentral.com/articles/10.1186/s12859-017-1705-x] calls this the read-count based method for detecting CNVs. Seeing that CNVs also represent interesting biological features, an omission of this discussion makes the submission feel incomplete.* It would make for a stronger submission if the features detected using `sequana_coverage` and how they would look like using a naive setting of coverage depths is discussed. The bacteria test case explained further in Figure 7, while interesting, does not really convince the readers on the need of this tool as the changes in coverages seem to be visible even without using the tool (i.e. just by plotting the raw coverages). In figures 8-9 it seems to be the case as well that detection of the ROI can be done by using only the mapping quality track.* What are the characteristics of the features that can be detected using this tool. For example, are they between a specific size range (in relation to, for example, the window size and/or the read length). This is not completely clear from the submission, and while some methods of correlating the detected features to known biological features are presented, one wonders if such detection can be made with simpler methods.Minor concerns:* Although the tool here is presented as a standalone tool, installing it requires installation of other tools and pipelines in the authors' `sequana` toolkit. It would be more suitable if the tool is also packaged as its own, truly standalone package.* The version of the `sequana_coverage` tool used for producing the plots is unfortunately not mentioned clearly. Could the authors clarify this?* The EM algorithm to which the author refers in the statistical section, should be expanded as Expectation Maximization instead of Expectation Minimization. This is how it was presented in the referred Dempster et al. (1977) paper.* The rationale on choosing the running median window size is not completely clear. Are there any relations between this value

and, for example, the read length or the reference genome size? * Could the author comment on the suitability of using `sequana_coverage` for other species such as humans, mice, and/or some plant species?* Page 2, line 27-30. The authors wrote: "For instance, to detect human genome mutations, single-nucleotide polymorphisms (SNPs), and rearrangements, a 50 X depth is recommended [1] to be able to distinguish between sequencing errors and true SNPs." Could the authors clarify which table and/or statements present in the cited article that contains this recommendation?

Level of Interest

Please indicate how interesting you found the manuscript: An article of limited interest

Quality of Written English

Please indicate the quality of language in the manuscript: Acceptable

Declaration of Competing Interests

Please complete a declaration of competing interests, considering the following questions:

- Have you in the past five years received reimbursements, fees, funding, or salary from an organisation that may in any way gain or lose financially from the publication of this manuscript, either now or in the future?
- Do you hold any stocks or shares in an organisation that may in any way gain or lose financially from the publication of this manuscript, either now or in the future?
- Do you hold or are you currently applying for any patents relating to the content of the manuscript?
- Have you received reimbursements, fees, funding, or salary from an organization that holds or has applied for patents relating to the content of the manuscript?
- Do you have any other financial competing interests?
- Do you have any non-financial competing interests in relation to this paper?

If you can answer no to all of the above, write 'I declare that I have no competing interests' below. If your reply is yes to any, please give details below.

I declare that I have no competing interests

I agree to the open peer review policy of the journal. I understand that my name will be included on my report to the authors and, if the manuscript is accepted for publication, my named report including any attachments I upload will be posted on the website along with the authors' responses. I agree for my report to be made available under an Open Access Creative Commons CC-BY license (http://creativecommons.org/licenses/by/4.0/). I understand that any comments which I do not wish to be included in my named report can be included as confidential comments to the editors, which will not be published.

I agree to the open peer review policy of the journal

To further support our reviewers, we have joined with Publons, where you can gain additional credit to further highlight your hard work (see: https://publons.com/journal/530/gigascience). On publication of this paper, your review will be automatically added to Publons, you can then choose whether or not to claim your Publons credit. I understand this statement. Yes
