# Supplementary material for: Sequana coverage: detection and characterization of genomic variations using running median and mixture models
Source: Gigascience. 2018 Sep 6;7(12):giy110. doi: 10.1093/gigascience/giy110 (PMC6275460; doi:10.1093/gigascience/giy110)
Supplement: Reviewer_2_Report_(Original_Submission) -- Rob Patro — 10/18/2018 Reviewed [file giy110_reviewer_2_report_(original_submission).pdf]

Reviewer Report

Title: Sequana Coverage: Detection and Characterization of Genomic Variations using Running Median and Mixture Models

Version: Original Submission **Date:** 10/18/2017

Reviewer name: Rob Patro

Reviewer Comments to Author:

In this manuscript, Desvillechabrol et al. introduce seqana_coverage, a method and tool for estimating statistically significant deviations in genome coverage data and providing an automated method to detect genomic regions of interest (ROI). The overall algorithm method seems sound, and consists of de-trending the data using a running-median filter, followed by learning foreground (center) and background (outlier) distributions, as well as their maximum likelihood mixture proportions in the sample. Finally, given the Gaussianity assumptions of the model, they define a z-score that can be computed for each base in the genome, which can aid in discovering loci that exhibit statistically surprising high or low coverage. I generally find the manuscript convincing, and the tool looks as though it will be useful. However, I have a couple of high-level questions about the proposed solution and the manuscript. Questions / comments----* There is not much discussion in the current manuscript on the effect of W, the window length used in the running-median de-trending algorithm. Do the authors have a recommended procedure for setting W? It would seem that the choice of W will set a "scale" for outliers, and while the approach would generally be robust to small changes in W, larger changes could highlight different regions of interest (this, of course, can be viewed as a feature rather than a problem). However, for someone wishing to use the tool, a recommendation on how to set W, or what values make sense for discovering different types of regions of interest, would be useful.* Related to the above, one potential effect of the de-trending that is performed in seqana_coverage is that outliers appear to generally be high-frequency effects. Would the proposed approach be applicable if one wished to discover low-frequency effects (i.e. very large ROIs)? For example, one might expect a dip in coverage in difficult-to-sequence regions or in regions with highly-repetitive arrays of elements. Would it be possible to detect such large-scale regions using the proposed approach, or do they not classify as outliers under the assumed definition?* I'm not certain I understand, or completely agree with, the argument that the outlier distribution can safely be assumed as Gaussian. Specifically, for the CLT to apply, we must be taking the (normalized) sum of a large number of *independent* random variables. However, it's not immediately clear to me why the outlier samples are independent --- e.g., changes due to repeated elements may be be correlated. Also, given the definition of the outlier samples (those with considerably higher or lower coverage than their local, de-trended window), it's not completely clear why this distribution may not be e.g., bimodal. Perhaps the assumption of normality is simply a computational convenience, as it allows closed-form update rules for the mixture model being applied. But perhaps this assumption could be addressed empirically (e.g., by looking at a goodness-of-fit test of the outlier samples to a normal distribution with the inferred mean and standard deviation).* It would be nice if the authors could provide a couple of examples demonstrating the underlying biological cause of some of the outliers identified by their algorithm. Specifically, the algorithm is demonstrated on 3 real datasets, and outliers are predicted. It would be a nice addition to the manuscript to select a few extreme outliers and discuss what gave rise to them.Minor concerns-----* The second sub-section of the methods section is titled "Building a statistics", which sounds awkward and should probably be reworded.* In the same section, when justifying the Gaussianity assumption for the outlier distribution, the authors claim "we can consider that the outliers population is a mix of samples and that we are in the limit of the central theorem". The wording here is a bit strange --- I would, instead, suggest something like "if we consider the outlier population as arising from a mix of different independent samples, then the central limit theorem applies, implying that $\tilde{C}_b^1$ can be treated as Gaussian."

Level of Interest

Please indicate how interesting you found the manuscript: An article of importance in its field

Quality of Written English

Please indicate the quality of language in the manuscript: Acceptable

Declaration of Competing Interests

Please complete a declaration of competing interests, considering the following questions:

- Have you in the past five years received reimbursements, fees, funding, or salary from an organisation that may in any way gain or lose financially from the publication of this manuscript, either now or in the future?
- Do you hold any stocks or shares in an organisation that may in any way gain or lose financially from the publication of this manuscript, either now or in the future?
- Do you hold or are you currently applying for any patents relating to the content of the manuscript?
- Have you received reimbursements, fees, funding, or salary from an organization that holds or has applied for patents relating to the content of the manuscript?
- Do you have any other financial competing interests?
- Do you have any non-financial competing interests in relation to this paper?

If you can answer no to all of the above, write 'I declare that I have no competing interests' below. If your reply is yes to any, please give details below.

I declare that I have no competing interests

I agree to the open peer review policy of the journal. I understand that my name will be included on my report to the authors and, if the manuscript is accepted for publication, my named report including any attachments I upload will be posted on the website along with the authors' responses. I agree for my report to be made available under an Open Access Creative Commons CC-BY license (http://creativecommons.org/licenses/by/4.0/). I understand that any comments which I do not wish to be included in my named report can be included as confidential comments to the editors, which will not be published.

I agree to the open peer review policy of the journal

o further support our reviewers, we have joined with Publons, where you can gain additional credit to further highlight your hard work (see: https://publons.com/journal/530/gigascience). On publication of this paper, your review will be automatically added to Publons, you can then choose whether or not to claim your Publons credit. I understand this statement. Yes
