# Supplementary material for: Sequana coverage: detection and characterization of genomic variations using running median and mixture models
Source: Gigascience. 2018 Sep 6;7(12):giy110. doi: 10.1093/gigascience/giy110 (PMC6275460; doi:10.1093/gigascience/giy110)
Supplement: Reviewer_3_Report_(Original_Submission) -- Ian Sudbery — 10/25/2017 Reviewed [file giy110_reviewer_3_report_(original_submission).pdf]

Reviewer Report

Title: Sequana Coverage: Detection and Characterization of Genomic Variations using Running Median and Mixture Models

Version: Original Submission **Date:** 10/25/2017

Reviewer name: Ian Sudbery

Reviewer Comments to Author:

Click here to enter text.

This manuscript presents a novel method for detecting regions of anomalous mapped read depth. As I understand it from the manuscript, the expected uses of this appear to be to detect problems with either a genome or metagenome assembly or sequencing sample, and to assess the GC bias in a sample. The authors do really address the other application that might seem obvious: the calling of CNVs. The following review will be broken down into two pieces: the manuscript and the software. I will conclude with a summary of my recommendations.

Manuscript

I have two major concerns:

Any new piece of software aiming to solve a problem where other pieces of software attack the same problem should be compared to these other tools. While the authors do mention a couple of other approaches, mostly from the field of metagenomics, there exist a range of tools that use read depth to investigate CNVs. See for example Yao et al Molecular Cytogenetics 2017 10:30 doi:10.118/s13039-017-0333-5, Zhao et al BMC Bioinformatics 2013 14(Suppl 11):S1 doi:10.1186/1471-2105-14-511-S1. The authors should set their method in the context of these methods and how how it differs in its aims/approach/performance. If applicable a comparison between their method and others on the same dataset should be presented. At a minimum I would like to see a comparison to CNVnator (Abyzov et al, Genome Research, 2011 21:974-948, doi: 10.1101/gr.114876.110) or an explanation of why such a comparison is not appropriate.

The algorithm has a single tuning parameter, W. The authors state that the value of this setting has little effect on the parameters learnt by their model. The authors should expand on this further: presumably the choice of W does have an effect on the z score obtained For example I believe any region of elevated or depressed coverage larger than W would clearly be missed. The authors should include a discussion of how changing W changes the results and how to choose a good value for W.

There are also a number of smaller concerns or comments.

Under "building a statistic" the authors state that the per-base coverage should theoretically follow a poisson distribution. Is it not actually the case that it is the number of reads that start at any given base that should follow a poisson distribution, and the non-independent nature of depths and nearby bases may account for some of the over-dispersion?

It is not clear to me why the gaussian approximation of the negative binomial becomes valid at 10X coverage, rather than 5 or 20.

The authors state that bases of 0 coverage are not included in their fitting, but would these values not be

important in finding the mixing parameters?

The authors state that one of the benefits of their approach is that a statistically meaningful value is attached to each base and that they can control the false positive rate this way. However, they then go on to use a two step threshold to identify regions. It seems to me that that the above benefit does not transfer over to the regions and it is difficult to assess the statistical properties of the regions. Perhaps the authors should comment on this in their discussion.

The authors comment on how the method scales to viral, bacterial and yeast datasets. How would the performance scale if applied to mammalian or plant genomes?

Software

Of the three available installation routes I was able to install the software without an error message at install time using only one route - via the sequana conda package.

I was a little perturbed to be made to download and install several Gb of dependencies just to use what one would image would be a fairly dependency-lite piece of software. Indeed I several times ran out of disk space on both the Ubuntu virtual machine and institutional HPC accounts I attempted the installation on. This was due to having to download an install the whole of the sequana collection, which has many dependencies way beyond the scope of the tool under review here.

I obtained the viral dataset used in the manuscript from the synergy and attempted to run the tool. The tool seemed to get though most of its processes, unfortunately I was met with an error I was unable to solve in what I assume was the reporting phase. Unfortunately I don't feel that I can provide a full review of the software until I am able to run it fully with out error.

My personal suggestion for a tool as self contained as this it would be better it to could be installed on a stand alone basis with a minimal number of dependencies. This would minimise the chance for things to go wrong and also minimise the footprint of the tool on the users system.

To be useful to many bioinformaticians, who do most of their work on institutional clusters, it needs to be possible to successfully install without root permissions. Conda should allow this to be possible.

Recommendations

I believe that the work here is a valuable contribution to the field and could be suitable for publication if the authors addressed the comments outlined. Of particular importance:

* Comparisons of the method to published methods using read-depth to call CNVs (for example CNVnator).
* A discussion of the effect of changing the W parameter and how to choose a good value
* I need to be able to install the software flawlessly on, for example, a freshly minted Ubuntu virtual machine or similar (X and conda or pip installed) and run without error. Ideally there should be a non-root requiring way to install.

Level of Interest

Please indicate how interesting you found the manuscript: An article whose findings are important to those with closely related research interests

Quality of Written English

Please indicate the quality of language in the manuscript: Acceptable

Declaration of Competing Interests

Please complete a declaration of competing interests, considering the following questions:

- Have you in the past five years received reimbursements, fees, funding, or salary from an organisation that may in any way gain or lose financially from the publication of this manuscript, either now or in the future?
- Do you hold any stocks or shares in an organisation that may in any way gain or lose financially from the publication of this manuscript, either now or in the future?
- Do you hold or are you currently applying for any patents relating to the content of the manuscript?
- Have you received reimbursements, fees, funding, or salary from an organization that holds or has applied for patents relating to the content of the manuscript?
- Do you have any other financial competing interests?
- Do you have any non-financial competing interests in relation to this paper?

If you can answer no to all of the above, write 'I declare that I have no competing interests' below. If your reply is yes to any, please give details below.

I declare that I have no competing interests

I agree to the open peer review policy of the journal. I understand that my name will be included on my report to the authors and, if the manuscript is accepted for publication, my named report including any attachments I upload will be posted on the website along with the authors' responses. I agree for my report to be made available under an Open Access Creative Commons CC-BY license (http://creativecommons.org/licenses/by/4.0/). I understand that any comments which I do not wish to be included in my named report can be included as confidential comments to the editors, which will not be published.

I agree to the open peer review policy of the journal

To further support our reviewers, we have joined with Publons, where you can gain additional credit to further highlight your hard work (see: https://publons.com/journal/530/gigascience). On publication of this paper, your review will be automatically added to Publons, you can then choose whether or not to claim your Publons credit. I understand this statement. Yes
