# Supplementary material for: Sequana coverage: detection and characterization of genomic variations using running median and mixture models
Source: Gigascience. 2018 Sep 6;7(12):giy110. doi: 10.1093/gigascience/giy110 (PMC6275460; doi:10.1093/gigascience/giy110)
Supplement: Reviewer_3_Report_Revision_1 -- Ian Sudbery — 3/27/2018 Reviewed [file giy110_reviewer_3_report_revision_1.pdf]

Reviewer Report

Title: **Sequana Coverage: Detection and Characterization of Genomic Variations using Running Median and Mixture Models**

Version: **Revision 1** **Date:** 3/27/2018

Reviewer name: **Ian Sudbery**

Reviewer Comments to Author:

The authors present a thoroughly updated revision of the manuscript that makes a serious attempt at updating many of my previous points. Following the authors advice, I am now able to install the software on a clean virtual machine through the singularity route. This route would still not present though for users without the necessary privileges to install the singularity software (e.g. institutional HPC), but I guess it is better than many packages. I was able to run the software on the provided example files. In the case of the viral genome I successfully identified, 8 depleted regions and one enriched region (see below). The enriched region is clearly a false positive as its depth is almost exactly at the long term trend for the genome, but is surrounded by two strong depleted regions that are pulling the average down. The largest update to the manuscript concerns an investigation into the use of sequana_coverage for detecting CNVs. This study begins with a simulation experiment. The authors do not report in the main text the number of false positives discovered in simulations with not simulated CNVs saying only "the number of ROIs detected with sequana_coverage varies from one simulation to the other". Examination of the referenced notebook reveals that their representative example has 14 regions. The authors state that these false positives "have short lengths (below 50) and low mean z-scores (below 5)." All methods produce false positive; using a threshold of 4, one would expect to find around 200 bases beyond the threshold if each base was independent (which it clearly isn't). Does this mean that ROIs shorter than 50bp and with z-scores below 5 should be ignored in CNV or other analyses, or are these bases identifying genuine features in the genome (such as unmappable sequence)? Clearly what might be considered a false positive for one analysis would not be for another, while some regions are genuinely false. This is a point that could perhaps be made in the discussion. The authors then compare their tool to other tools, and find roughly comparable performance, although it is unfortunately that the same level of analysis doesn't seem to have been applied to the sequana_coverage only ROIs as has been applied to the CNVnator and CNOGpro only events. In particular the authors do not mention any false positives in the viral genome. There are several, as I found when I ran the viral genome analysis myself, including one an enrichment that appears to be caused by normal depth sequence sitting between two depleted regions, which could point to the necessity of further W value tuning. The authors briefly touch on performance of the tool on the human genome as requested, although I am somewhat confused as to the results. In the paper they mention that the tools runs as quickly as 30 minutes on a dual core machine for the human genome. However, in response to another author they say that running on a human bed takes 1 hour on 24 cores, or 20 hours on one core. In their response the authors repeated refer to leaving things out because of limited space, but I was under the impression that GIGA-science did not impose length limits on articles. In particular the revised manuscript contains a large number of references to notebooks stored on the sequana github. I feel like many of these would make useful figures or supplementary figures for those not conversant with the python. One might also wonder if the notebooks would be better as supplementary files rather than links to a potentially volatile github site. Some of the lanuage is less polished than in the original submission. The authors have address many of my points. While I would appreciate some further discussion, particularly on the nature of the false-positives, I do not think that this should prevent publication.

Level of Interest

Please indicate how interesting you found the manuscript: An article whose findings are important to those with closely related research interests

Quality of Written English

Please indicate the quality of language in the manuscript: Needs some language corrections before being published

Declaration of Competing Interests

Please complete a declaration of competing interests, considering the following questions:

- Have you in the past five years received reimbursements, fees, funding, or salary from an organisation that may in any way gain or lose financially from the publication of this manuscript, either now or in the future?
- Do you hold any stocks or shares in an organisation that may in any way gain or lose financially from the publication of this manuscript, either now or in the future?
- Do you hold or are you currently applying for any patents relating to the content of the manuscript?
- Have you received reimbursements, fees, funding, or salary from an organization that holds or has applied for patents relating to the content of the manuscript?
- Do you have any other financial competing interests?
- Do you have any non-financial competing interests in relation to this paper?

If you can answer no to all of the above, write 'I declare that I have no competing interests' below. If your reply is yes to any, please give details below.

I declare that I have no competing interests

I agree to the open peer review policy of the journal. I understand that my name will be included on my report to the authors and, if the manuscript is accepted for publication, my named report including any attachments I upload will be posted on the website along with the authors' responses. I agree for my report to be made available under an Open Access Creative Commons CC-BY license (http://creativecommons.org/licenses/by/4.0/). I understand that any comments which I do not wish to be included in my named report can be included as confidential comments to the editors, which will not be published.

I agree to the open peer review policy of the journal

To further support our reviewers, we have joined with Publons, where you can gain additional credit to further highlight your hard work (see: https://publons.com/journal/530/gigascience). On publication of this paper, your review will be automatically added to Publons, you can then choose whether or not to claim your Publons credit. I understand this statement. Yes
